# Orchestrating photoswitch thermal backconversion using hydrogen bonds to reshape energy landscapes

**DOI:** 10.1038/s41467-026-76898-2

**Published:** 2026-08-22

**Authors:** Liang Fei, Zacharias Liasi, Jacob L. Elholm, Helen Hölzel, D. Flemming Hansen, Kurt V. Mikkelsen, Kasper Moth-Poulsen

**Affiliations:** 1 Department of Chemical Engineering, Universitat Politècnica de Catalunya, EEBE, Barcelona, Spain; 2 Department of Chemistry, University of Copenhagen, Copenhagen, Denmark; 3 Institute of Organic Chemistry, Justus-Liebig-University Giessen, Giessen, Germany; 4 Division of Biosciences, Department of Structural and Molecular Biology, University College London, London, UK; 5 Department of Chemistry and Chemical Engineering, Chalmers University of Technology, Gothenburg, Sweden; 6 The Institute of Materials Science of Barcelona, ICMAB-CSIC, Barcelona, Spain; 7 Catalan Institution for Research & Advanced Studies, ICREA, Barcelona, Spain

**Keywords:** Optical materials, Light harvesting

## Abstract

Thermodynamic control of molecular photoswitches remains a fundamental challenge, centered on the stabilization of high-energy metastable states. Inspired by concepts of selective kinetic regulation found in Maxwell’s demon-like systems, we explore hydrogen bonding as a molecular-level selector for modulating the thermal back-conversion of quadricyclane (QC) to norbornadiene (NBD). By strategically incorporating hydrogen-bond donors and acceptors, we show that these interactions build a water “bridge” that restricts rotational degrees of freedom. This bridge significantly elevates the kinetic barrier and redirects the isomerization along an alternative reaction coordinate. In this manner, the hydrogen-bonding system selectively regulates the thermal back-conversion dynamics and enables directional control over the isomerization kinetics, achieving up to a 5000-fold extension of the thermal half-life. This work illustrates how hydrogen bonds can facilitate precise control over back-conversion dynamics and molecular thermal stability in molecular photoswitches.

## Abstract (web summary)

It is challenging to thermodynamically control molecular photoswitches, due to the high energy metastable states. Here the authors explore using hydrogen bonding groups to regulate the dynamics between quadricyclane to norbornadiene, controlling isomerization kinetics.

## Introduction

**Fig. 1:**
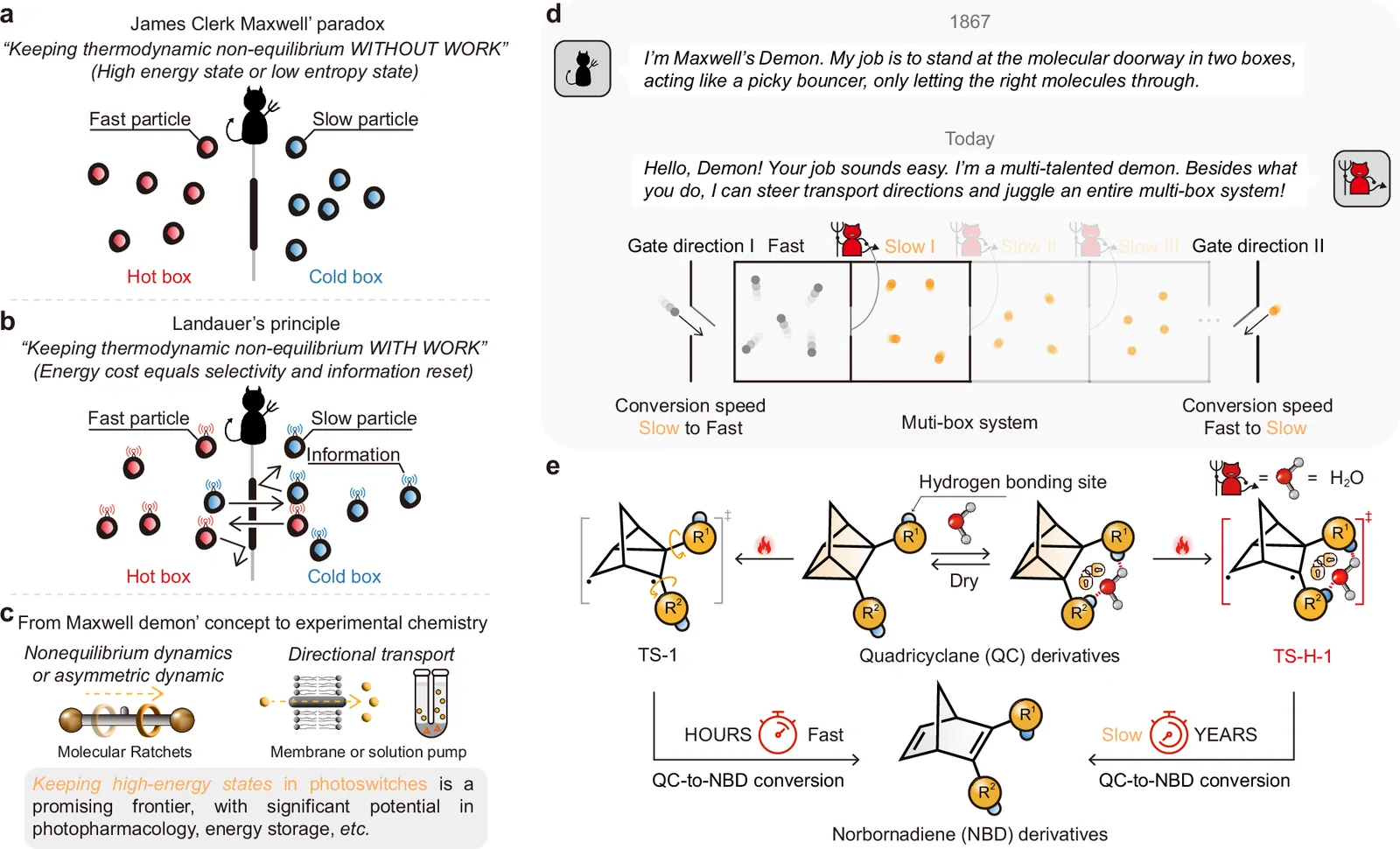
Concept of hydrogen bonds play Maxwell’s demon in norbornadiene/quadricyclane pair system. **a** James Clerk Maxwell’s paradox: a demon operates a frictionless gate to sort particles without performing work. **b** Landauer’s principle: the thermodynamic necessity of an energy cost associated with selective control and information reset. **c** Recent experimental chemistry from Maxwell demon’s concept^6,8,11,62,63^, and the promising frontier for high-energy photoswitches investigated in this study. **d** A comparison of Maxwell demon in 1867 and this work. **e** This work on hydrogen-bond supermolecular NBD system for broadly-tuneable thermal backconversion dynamics.

The spontaneous decay of high-energy states toward thermodynamic equilibrium is a fundamental law of nature. Yet, the pursuit of strategies to sustain non-equilibrium states has remained a cross-disciplinary challenge for over a century. This inquiry traces its intellectual lineage to the seminal thought experiments of Maxwell’s paradox and Landauer’s principle (Fig. 1a, b)^1,2^, which conceptualize a “demon” capable of selectively partitioning particles to reduce a system’s entropy^3–5^. In current molecular science, Maxwell’s demon-inspired systems are generally not viewed as strict realizations of the original entropy-reduction thought experiment or as attempts to violate the second law of thermodynamics. Rather, the concept has evolved into a broader phenomenological and information-guided framework for describing selective kinetic regulation, directional transport, and nonequilibrium molecular processes, including molecular machines and membrane transport systems (Fig. 1c)^6–11^. However, while significant progress has been made in driving molecular systems away from equilibrium, the challenge of stabilizing these transient states through autonomous, selective regulation remains virtually unexplored.

At the molecular level, photoswitches provide a unique platform to investigate thermodynamic principles, as their reversible isomerization inherently facilitates controlled transitions between thermodynamically stable low-energy and metastable high-energy states^12^. Beyond their fundamental interest, these systems have been applied into diverse applications, including molecular machines^9,13–15^, photopharmacology^16–18^, and molecular solar thermal energy storage^19–22^. Among these, the norbornadiene (NBD)/quadricyclane (QC) pair stands out as one of the most extensively studied photoswitching systems^23–25^. Its significant energy storage capacity (up to ~1 MJ kg^−1^) and inherent susceptibility to thermal reversion make it an ideal molecular laboratory for emulating Maxwellian dynamics, where the challenge lies in selectively arresting the spontaneous decay of the high-energy QC isomer in a controlled way. The catalytic and photochemical methods have been reported to accelerate the QC-to-NBD back-isomerization, driving the system toward thermodynamic equilibrium^26–28^. Short thermal half-lives (*t*_1/2_,describing the kinetic), however, remain a major obstacle to practical applications. Although prolonging *t*_1/2_ by raising the thermal back-conversion barrier through substituent engineering is possible^29–31^, such strategies require significant synthetic effort and often compromise intrinsic molecular properties^25,32,33^. Moreover, a controllable *t*_1/2_ is highly desirable, as different applications demand different timescales: photopharmacology often requires thermal relaxation within minutes to hours^34,35^, whereas solar thermal storage systems benefit from months to years of stability^19,20,36^. The search for a reversible “demon” to selectively control over metastable high-energy states thus represents a major frontier in molecular engineering.

Here we introduce a supramolecular strategy, inspired by the hydrogen-bonding motifs that underpin molecular recognition in biological systems and supramolecular assemblies^37–41^, to regulate the thermal dynamics of photoswitchable molecules. In our approach, water molecules act as dynamic information carriers encoded through reversible supramolecular interactions, modulating molecular energy landscapes. Although individual hydrogen bonds are weak (tens of kJ mol^−1^)^42,43^, their collective action can dramatically enhance binding strength and provide precise dynamic control^44–47^. We demonstrate that strategically positioned hydrogen bonds in NBD derivatives can selectively stabilize transition-state geometries, reversibly raise activation barriers, and even reroute thermal isomerization reaction pathways. This supramolecular design elevates hydrogen bonding from a passive structural motif to an active regulator of entropy and enthalpy and by that the thermal reactivity, enabling tunable back-conversion dynamics that address the key limitations of current photoswitch systems.

## Results

### Molecular design for a Maxwell’s demon-inspired system

A Maxwell’s demon-inspired framework is used to describe a selective molecular gating behavior that biases transport directionality within a multi-box system (Fig. 1d). Our system comprises two compartments (the Slow Box and Fast Box), representing distinct thermal back-conversion rates from QC to NBD, separated by dynamic hydrogen-bonding networks. These hydrogen-bonding interactions stabilize the designed QC derivatives and increase the thermal back-conversion barrier. Importantly, water molecules can modulate these interactions by selectively interacting with the QC derivatives, thereby acting as a dynamic regulatory mediator of the local hydrogen-bonding environment. Initially, QCs distribute between the Slow and Fast Boxes depending on the presence of hydrogen bonds. The gating direction is tunable: introducing water promotes hydrogen bonding, shifting QCs from the Fast Box to the Slow Box, whereas removing water reverses this flow (see Supplementary Section 2.5, Figs. S1 and S5).

The structural design of our system is critical; however, when developing molecular systems for specific applications, improving one property often comes at the expense of another. In this study, we address a well-known trade-off between thermal back-conversion dynamics and absorption wavelengths, which can be mitigated through our hydrogen-bonding strategy. While red-shifted absorption in donor-acceptor molecules enhances light-harvesting efficiency, it drastically reduces *t*_1/2_ from months to minutes^25,32,33,48,49^. This phenomenon arises from the reduced S_0_-S_1_ energy gap in NBD, which simultaneously lowers the thermal back-conversion barrier, which is a challenge not only for NBD-QC pairs but for most types of photoswitches^50–52^. In our previous work, we introduced bulky substituents to enhance steric repulsion, thereby restricting the rotational motion of side groups and hindering the back-conversion pathway (Fig. S24)^31^. While this approach partially decouples the absorption spectrum from back-conversion rates, *t*_1/2_ remains difficult to control, and the increased molecular weight from bulky groups can reduce the energy storage density.

A hydrogen-bonding supramolecular strategy is demonstrated to effectively tune backconversion dynamics by incorporating hydrogen-bond donor (D) and acceptor (A) building blocks at the 2- and 3-positions of unsubstituted NBDs (Fig. 1e). We present a series of target compounds, **NBD1-6**, each featuring distinct hydrogen-bonding sites (Figs. 2a, b, and S1–23). To explore the most effective hydrogen-bonding supramolecular NBD system, i.e., the molecular structure most sensitive to hydrogen bonding, a family of six NBD derivatives was investigated. These derivatives include hydrogen bond acceptors (cyano group, –CN; ether group, –O–; ester group, –C(=O)O) and a donor (secondary amino group, –NHR). The nature of the hydrogen-bonding sites, along with the strength of the acceptor and donor groups, can significantly influence the hydrogen-bonding interactions between substitutes and water molecules.

**Fig. 2:**
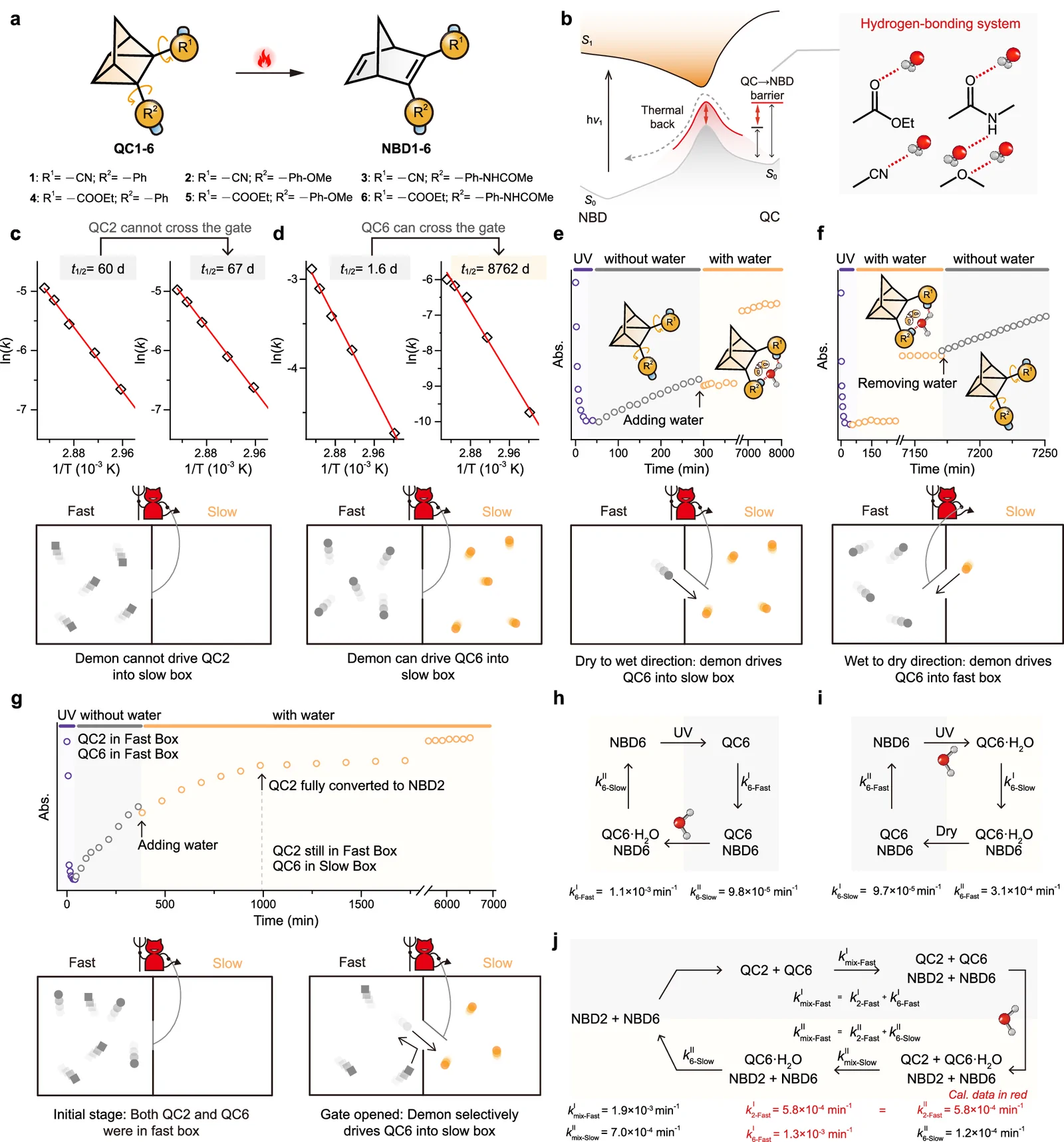
Demon plays the backconversion dynamics. **a** Molecular structures of NBD/QC1-6. **b** Energy diagram of a backconversion process between NBD and QC, and their hydrogen bonding sites. Arrhenius plots of **c** **QC2** and **d** **QC6** in MeCN without water (left) and with water at a molar ratio of NBD:water = 1:20 (right). Cartoon of Maxwell’s demon system illustrates that **QC2** is unable to pass through the gate, whereas **QC6** can. Backconversion plots of **QC6** at 60 °C in MeCN **e** from dry to wet and **f** from wet to dry. Cartoon of Maxwell’s demon system represents the directional gate-opening control. **g** Backcoversion plots of **QC2** and **QC6** mixture at 60 °C in MeCN. Cartoon of Maxwell’s demon system represents the selective molecular transport. Kinetic model of **h** dry to wet direction of **QC6** solution, **i** wet to dry direction of **QC6** solution, and **j** selective transport of **QC2** and **QC6** mixture solution.

The photoisomerization properties of **NBD1-6** were studied using UV-Vis absorption spectroscopy in toluene (Fig. S25) to prevent hydrogen bonding with solvent molecules. The NBD derivatives exhibited strong absorption above 300 nm, attributed to the donor-acceptor groups introduced on the double bond. Upon exposure to 340 nm light, all compounds underwent nearly complete photoisomerization (>97%) to their corresponding QC forms (Figs. S26–31). The loss of conjugation within the push-pull system resulted in a blue shift in the absorption wavelengths of the QC isomers.

The back-isomerization from QC to NBD was found to be thermally induced, and all NBD derivatives were subjected to a cycling test in solution, involving repeated photoisomerization and subsequent thermal back-conversion at 80 °C. **NBD1-3**, which contain cyano groups, remained stable over 20 cycles with negligible degradation, whereas **NBD4-6**, featuring ester groups, exhibited slight degradation. UV irradiation of the NBD derivatives can lead to photoinduced degradation, resulting in species that cannot undergo back-isomerization to the NBD form. This effect is particularly pronounced in NBD derivatives containing a weakly electron-withdrawing ester group in combination with a strongly electron-donating substituent^21^. Moreover, some NBD units have been reported to undergo photoinduced polymerization, and cannot be back-converted to the initial state under any circumstances^22,26,53,54^.

### Thermal backconversion dynamics control

To investigate the thermal backconversion dynamics of QCs, we examined the effect of varying molar ratios of water molecules to QC in acetonitrile (Figs. S32–67 and Tables S1–19). At the start of the experiment, we measured the backconversion rates (represented by the rate constant, *k*) and fit the data to the Arrhenius plots for all QCs, both with and without hydrogen-bonding systems, to assess the influence of hydrogen bonding on the backconversion dynamics. The *k* values of **QC1** and **QC2** were not affected by the presence of water; therefore, both were categorized to be in the Fast Box (Figs. 2c and S68a). In contrast, for **QC3-6**, which contain hydrogen-bonding sites, the *k* values decreased significantly, indicating slower backconversion rates. In these cases, QCs without water and those with water were classified in the Fast Box and Slow Box, respectively (Figs. 2d and S68b–d). Notably, the *t*_1/2_ value of **QC6** in the hydrogen-bonding system increased by approximately 5000-fold (from 1.6 to 8762 days, Table S19), demonstrating that multiple hydrogen bonds can have a pronounced impact on backconversion dynamics.

To further verify the direct control of backconversion dynamics in this selective gating system, two-way experiments of **QC6** between the Fast Box and Slow Box were performed. In the initial state, when the **QC6** solution contained no water, approximately 30% of **QC6** converted into **NBD6** within 250 minutes, with a *k*^I 6-Fast^ value of 1.1 × 10^−3 ^min^-1^ (Fig. 2d, h). Upon the addition of water, the backconversion rate of **QC6** decreased dramatically, with the *k*^II^_6-Slow_ value reducing to 9.8 × 10^−5^ min^−1^. When the water ratio reached 20, the back-conversion rate remained nearly constant, even upon further increasing the water ratio to 200 (Figs. S66, 67 and Table S18). In this scenario, the system enables a preferential redistribution of species from the Fast Box to the Slow Box. Conversely, when the **QC6** solution initially contained water, the backconversion rate accelerated again after the water was removed, corresponding to a transition from the Slow Box back to the Fast Box (Fig. 2e, f). To confirm the selectivity of this gating behavior, a control experiment was performed using **QC2** (Fig. S69). The backconversion dynamics of **QC2** remained unchanged upon adding or removing water from the system, indicating that **QC2** cannot cross the gate. These results demonstrate that the demon can reversibly switch the kinetics between the two states for the **NBD6**/**QC6** pair, suggesting that the observed motion arises from the hydrogen-bonding system.

Beyond the directional transport of backconversion dynamics, the demon can selectively control that some of QC cross the gate, while others cannot cross. In the initial state, the mixture solution of **QC2** and **QC6** contained no water and the *k*^I^_mix-Fast_ was integrated by the *k*^I^_6-Fast_ and *k*^I^_2-Fast_, where both of QCs were in the Fast Box (Fig. 2g, j). Upon the addition of water, only **QC6** can cross the gate, resulting from the slower *k*^II^_6-Slow_, while the *k*^II^_2-Fast_ kept same values. Due to that *k*^II^_6-Slow_ is significantly slower than *k*^II^_2-Fast_, nearly all of **QC2** converted into **NBD2** in 1000 min, while the **QC6** remained unconverted. This selective conversion of QCs can provide a great potential for backconversion control in multi photoswitching systems.

**Fig. 3:**
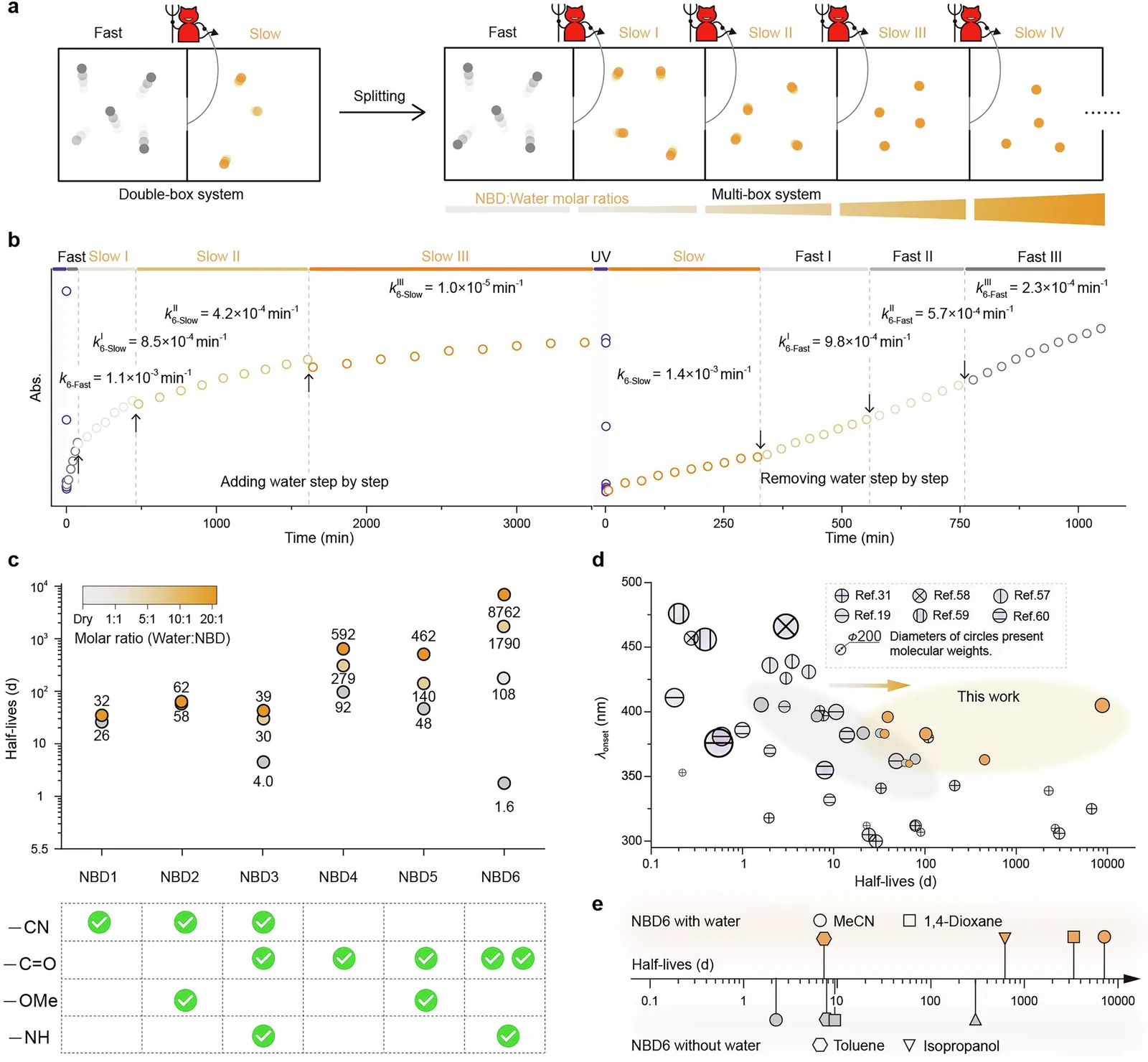
Thermal backconversion dynamics of QCs. **a** Cartoon representation of the Maxwell’s demon system, showing how the demon gauges multi-box system. **b** Backconversion plots of **QC6** at 60 °C in MeCN, obtained by stepwise addition and removal of water. For water addition, the molar ratios of NBD to water were 1:3 (Slow I), 1:5 (Slow II), and 1:20 (Slow III). For water removal, the ratios were 1:5 (Fast I), 1:2.5 (Fast II), and 1:0 (Fast III). **c** Half-lives of QCs in MeCN with various molar ratios between water molecules and QC at 25 °C in MeCN, which are calculated by Arrhenius plots (the detailed spectroscopic data in Supplementary Information Section 3.2.2). **d** Half-lives, onset absorption wavelengths (*λ*_onset_), and molecular weights of QCs in reported literature and this study. **e** Half-lives of **QC6** in various solvents (the detailed spectroscopic data in Supplementary Information Section 3.2.3).

Compared to traditional Maxwell’s demon-inspired systems where molecular transport is typically considered between two compartments, here we demonstrate a multi-state system regulated through hydrogen-bonding interactions. Changes in the NBD-to-water molar ratio provide a direct way to control the backconversion dynamics, which can be regarded as analogous to infinitely splitting the boxes (Fig. 3a). Initially, **QC6** was in the anhydrous state with a *k*_6-Fast_ of 1.1 × 10^−3 ^min^−1^ (Fig. 3b). During water addition, the backconversion rate decreased stepwise to 8.5 × 10^−4^, 4.2 × 10^−4^, and 1.0 × 10^−5 ^min^−1^. Upon water removal, the rate gradually increased from 1.4 × 10^−5^ to 2.3 × 10^−4^ min^−1^. The final dried rate is lower than the initial rate because water removal under reduced pressure could not achieve complete dryness (see “Methods”). This process can thus be viewed as the demon kinetic gating molecular passage through each “box”.

The thermal half-life (*t*_1/2_) is a critical parameter for evaluating thermal backconversion dynamics, which directly influences practical applications such as energy storage duration in molecular solar thermal systems, data retention and writing in information storage, and response time in photochromic devices^18,29,41,55,56^. For **QC1** and **QC2**, the *t*_1/2_ values remained largely unaffected by the hydrogen bonding system. In contrast, **QC3-6** exhibited a gradual increase in *t*_1/2_ with increasing water:NBD molar ratios (Fig. 3c). These findings are consistent with the corresponding changes observed in the thermal backconversion rate constants (Tables S1–18). Additionally, even at the highest NBD-to-water molar ratio (1:20), the water content in acetonitrile remains as low as 20 mM (0.0046 wt%), such that changes in bulk solvent properties are negligible (see Supplementary Information Section 2.4.4). Compared to previously reported NBD/QC pairs^19,31,57–60^, **QC3**-**6** demonstrated significantly extended *t*_1/2_ values, attributed to the formation of hydrogen bonding networks, without compromising blue-shifted absorption or introducing excessive steric bulk (Fig. 3d). In addition, the magnitude of half-life modulation achieved through this hydrogen-bonding strategy exceeds that reported for conventional solvent-effect-based approaches (see Table S27), with the thermal half-life tunable over a remarkably broad timescale, from hours to years.

The influence of solvent polarity and water miscibility on *t*_1/2_ was further investigated for **QC6** in five different solvents: MeCN, 1,4-dioxane, isopropanol, toluene, and hexane (Figs. 3e, S70–81, and Tables S20–26). In nonpolar solvents such as toluene and hexane, *t*_1/2_ remained unchanged upon water addition due to poor miscibility and the inability to establish effective hydrogen bonding interactions. In contrast, significantly prolonged *t*_1/2_ values were observed in MeCN and 1,4-dioxane, while isopropanol showed only a slight effect, due to the favorable solvation of water molecules over **QC6** interactions, hindering hydrogen bond formation. Notably, *t*_1/2_ values in nonpolar solvents were generally higher than those in polar solvents. This trend can be attributed to the ability of polar solvents to stabilize the transition state and lower the activation energy barrier (Table S26), thereby accelerating the thermal backconversion process.

### Evidence of reversible hydrogen bonding systems

**Fig. 4:**
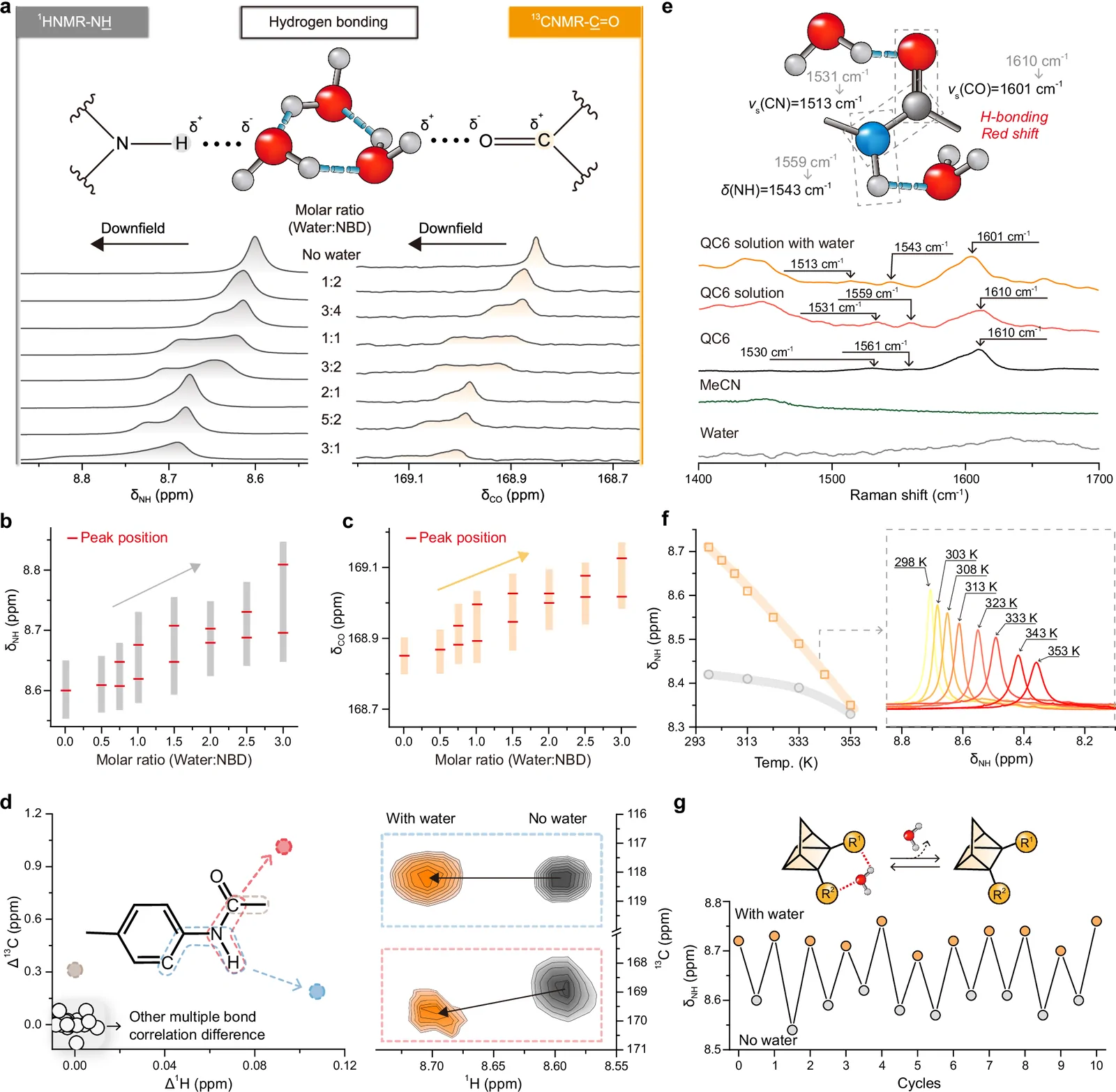
Evidence for the hydrogen bonding system. **a** ^1^H NMR (400 MHz) and ^13^C NMR (101 MHz), **b** peaks of NH, and **c** peaks of CO of **NBD6/QC6** pair with different ratios of water in CD_3_CN at 25 °C. **d** Chemical shifts of protons and carbons of **NBD6** in ^1^H-^13^C HMBC (400 MHz) spectra with and without water in CD_3_CN at 25 °C. **e** Raman spectra of **QC6** solution, MeCN, water. **f** ^1^H NMR (400 MHz) of **NBD6/QC6** with and without water under various temperatures in CD_3_CN at 25 °C. **g** Cycles of hydrogen bonding forming in **QC6** CD_3_CN solution.

As in the Maxwell’s thought experiment above, our initial system is driven by hydrogen bonding interactions to generate a shifted kinetic dynamic upon the introduction of water molecules. In the following paragraphs we will explore the system further using a combination of experimental and computational methods in order to elucidate the underlying mechanisms of the pronounced selectivity and acceleration effects. The chemical shifts (δ) of the amidic proton (^1^H) and carbonyl (^13^C) carbon in amides are commonly monitored to probe local environmental factors such as hydrogen bond strength, microsolvation, electrostatics, and polarity^43,61^. Accordingly, we investigated the proton (–CONH) and carbon (–CONH and –COOEt) signals of the **NBD6/QC6** pair under varying molar ratios of water to NBD/QC (Fig. 4a–c). The ^1^H nuclei of -CONH exhibited a deshielding (downfield) shift due to N–H···O–H hydrogen bonding, which results from a reduction in electron density around the hydrogen nuclei^39,40,56^. The δ_NH_ values progressively shifted downfield with increasing water content, indicating the formation of stronger hydrogen bonds, with a maximum shift of 0.2 ppm. Similarly, the ^13^C_CO_ nuclei involved in hydrogen bonding showed deshielding behavior, with a maximum shift of 0.3 ppm. Interestingly, at low water concentrations, the –CONH signals split into two peaks, due to partial hydrogen bonding of these groups with water molecules. In the parent NBD and NBD2 systems, all proton and carbon NMR signals displayed only minor negative shifts (<0.1 ppm) as the amount of added water increased (Figs. S82–85).

To reveal where hydrogen bonding occurs in **QC6**, we followed the structural evolution using ^1^H-^13^C HMBC spectra (Fig. 4d). Compared to the anhydrous solution, both the proton and carbon cross-peaks of H–N–C=O shifted significantly downfield, with Δδ_NH_ and Δδ_CO_ values of 0.1 and 0.9 ppm, respectively, due to deshielding by hydrogen bonding with water molecules. In contrast, water molecules had only a negligible effect on the aromatic carbons, resulting in a much smaller shift (0.15 ppm) in the H–N–C–C fragment. Besides, the cross-peaks of O=C–C–H only exhibited a ^13^C shift (0.3 ppm) but no ^1^H nuclei shift, suggesting that hydrogen bonds form with the ester group but not with the C–H group. Other protons and carbons in **QC6** were negligibly changed, indicating the absence of hydrogen-bonding interactions with those groups.

To further confirm the hydrogen bond forming, we now turn our attention to the Raman spectra of **QC6**, in solutions, and pure solvents (Fig. 4e). The symmetric stretching (*ν*_s_) modes of C=O and C–N, as well as the bending vibration (*δ*) of the N–H group, were observed in pure **QC6** at 1610, 1531, and 1559 cm^−1^, respectively. These bands remained almost unchanged upon dissolution in MeCN (0.1 M), but shifted to lower frequencies upon hydrogen-bond formation. Taken together, the NMR and Raman spectra indicate that the hydrogen-bonding sites are located in the amide and ester groups of **QC6**.

The in situ ^1^H NMR patterns are performed to recorded to monitor the changes in hydrogen bonding over the temperature range of 298–353 K (Fig. 4f). As the temperature increased, the N–H peak shifted upfield, which is higher than that in the anhydrous solution This shift arises because higher temperatures weaken the hydrogen-bonding interactions, thereby reducing the deshielding effect. We also evaluated the reversibility of the hydrogen-bonding and dehydrogenation processes (Fig. 4g). The chemical shift of the N–H was found to change reversibly over 10 cycles, demonstrating excellent durability and potential for practical applications.

### Simulations for backconversion pathway

**Fig. 5:**
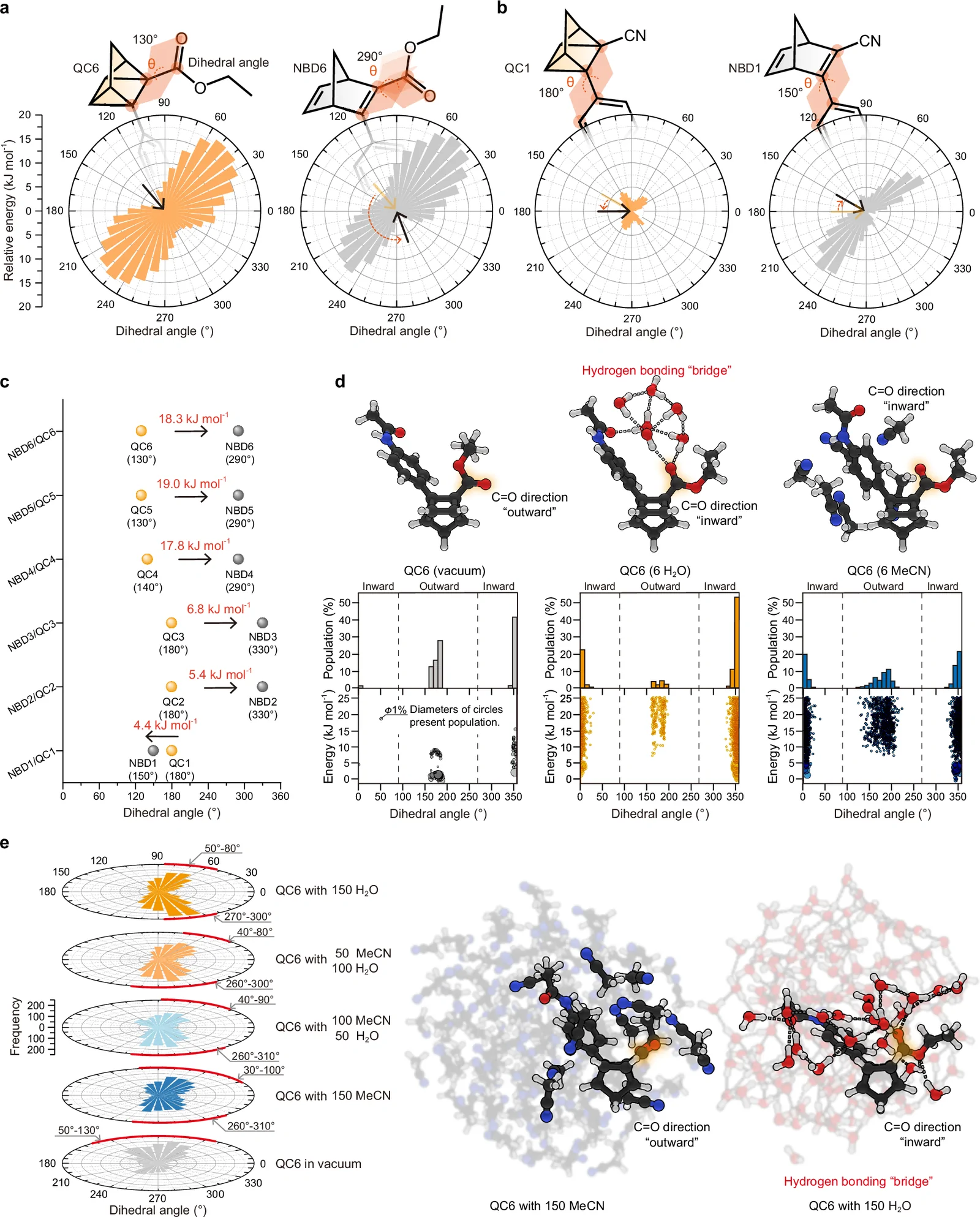
Simulations of NBD/QC pairs. Dihedral angles and energy landscapes in **a** **NBD6/QC6** and **b** **NBD1/QC1** pairs. **c** Rotational angles and the corresponding energies along the dihedral angle scans. The annotated angles and energy values correspond to the minimum- and maximum-energy points identified during the dihedral angle scanning process, respectively. **d** Inward and outward orientation population and energy landscapes of **QC6** in vacuum, with six H_2_O molecules, and with six MeCN molecules. **e** Molecular dynamics and relative structures of **QC6** at 298.15 K. The carbonyl dihedral angle was monitored across 2.5 ns trajectories under five solvent conditions: pure vacuum, 150 explicit MeCN molecules, mixed H_2_O/MeCN solutions (50 H_2_O/100 MeCN and 100 H_2_O/50 MeCN), and 150 explicit H_2_O molecules.

To elucidate the molecular mechanism underlying water-mediated control of thermal backconversion, we performed comprehensive density functional theory calculations combined with molecular dynamics simulations (Fig. 5). Global conformer searches and dihedral angle scans provide complementary insight into the rotational energy profiles (Figs. S86–91, and Movie S1).

The rotamer minima of **QC5** and **QC6** occur at identical dihedral angles as their NBD counterparts, but their relative energies invert between isomeric states (Figs. 5a, b, S94–98, and Movies S2, 3). Consequently, QC-to-NBD isomerization necessitates rotation of the carbonyls toward outward orientations through intermediate angles (~90–270°). Electrostatic potential maps reveal that the **NBD6**/**QC6** pair localizes stronger negative charge on the ester group and stronger positive charge on the amide group (Figs. S99, 100), creating favorable sites for water-bridge formation. Water selectively stabilizes inward conformers (~350° and 0–30°), such that escape from this water-locked geometry requires rotational reorganization of up to 180° prior to reaching the transition state. (Figs. 5c, 98, Table S28, Movie S2). This water-mediated conformational inversion is absent in **QC1-3** (Figs. S86–88), which lack suitable hydrogen-bonding sites, underscoring the selectivity of the supramolecular interaction.

QC derivatives exhibited pronounced solvent-dependent conformational shifts, with response magnitude increasing systematically from **QC4** to **QC6** (Figs. 5d and S100–102). **QC6** showed the strongest solvent response, nearly fully inverting its conformational preference. In vacuum, populations were bimodal (~80% at 170–210°, ~16% at 350°). In acetonitrile, ~64% occupied inward regions (>270°), while the lower-energy outward region (170–230°) accounted for ~13%. In water, ~92% populated inward conformations (~72% at 350°, ~20% at 10°), effectively eliminating outward orientations. Conformer searches produced substantially larger ensembles in solvent (acetonitrile: 549–3291 conformers; water: 321–4095) than in vacuum (16–80), with corresponding energy ranges of ~25 kJ mol^−1^ versus 6.9–19.4 kJ mol^−1^. **QC5** in water exhibited the lowest dominant conformer population fraction (63.8–81.6%), indicating that water stabilizes multiple rotameric states, yielding high conformational heterogeneity with both inward and vacuum-preferred orientations similarly populated.

Molecular dynamics simulations validated these conformational predictions and characterized dynamic sampling at 298.15 K (Figs. 5e, and S103–109). NBD derivatives displayed broad dihedral distributions (standard deviation, SD = 77–130°) largely unaffected by solvent, indicating minimal hydrogen-bond sensitivity. In contrast, QC counterparts experienced strong water-induced conformational restriction. QC6 showed intermediate flexibility (SD = 90–105°) with clear outward carbonyl preference, though inward rotamers remained populated. In water, the bimodal distribution narrows substantially, reflecting stabilization of two distinct water-locked rotameric states.

These findings provide insight into the mechanism underlying the selective regulation of thermal backconversion. Water channels the solute into preferred conformations, reducing rotational entropy in exchange for enthalpic stabilization through hydrogen bonding. By stabilizing the higher-energy inward rotamer, water displaces QC molecules from their intrinsic conformational ground states into configurations more distant from transition-state geometries. Beyond conformational narrowing, simulations reveal water molecules bridging the side groups and forming clusters around hydrophilic sites, with both the degree and prevalence of this structuring increasing systematically across the NBD/QC derivative series. Thermal backconversion thus requires overcoming not only the intrinsic activation barrier but also the additional energetic penalty of conformational reorganization-escaping from water-locked geometries to access transition-state-competent structures.

## Discussion

Using a hydrogen-bonding NBD/QC photoswitch system, and a combined experimental and theoretical framework, we have shown how Maxwell’s demon can conceptually be realized at the molecular scale. In the studied system, the passage through a thermal gate is regulated by hydrogen-bond interactions embedded within the NBD scaffold. The demon selectively controls transport directions and pathway selection within a multi-box setup. Here, hydrogen-bond donors and acceptors act as molecular selectors, reshaping the rotational energy landscape of QC-to-NBD interconversion, raising water-locked geometry barriers, and diverting the backisomerization pathway. The system exploits the distinct supramolecular environments of QC and QC-water complexes to establish a tunable kinetic gradient, adjustable via the NBD-to-water ratio. Remarkably, **QC6**-water interactions prolong the thermal half-life by over 5000-fold compared to the anhydrous form, an effect attributed to multiple hydrogen bonds imposing steric and enthalpic constraints at the transition state. This strategy could be extended beyond intramolecular hydrogen bonding to other switchable interactions, such as small-anion coordination or reversible covalent motifs, offering new ways to tune the energy landscape. Complementary to such ground-state stabilization strategies, excited-state approaches, including triplet sensitization or vibronic-coupling pathways^24^, may provide alternative strategies to enhance photoisomerization efficiency and further optimize photoswitch performance. Beyond solution-phase systems, embedding such photoswitches in solid matrices or supramolecular frameworks could further enhance thermal stability and enable cooperative effects.

## Methods

### Synthesis

The synthesis and characterization of NBD/QC derivatives (Figs. S2–4) are provided in Supplementary Information.

### Directional transport of backconversion dynamics

#### Fast Box to Slow Box

Solutions of NBD/QC pairs were prepared in anhydrous MeCN (0.1 mM) and heated to 60 °C. The absorbance at the maximum wavelength of NBD was monitored using a home-built UV-Vis spectroscopic setup. Samples were irradiated under UV light (*λ* = 315 nm, mounted LED, minimum output 53 mW, 700 mA, Thorlabs) until PSS was reached, after which irradiation was stopped to initiate the thermal back-conversion process. After 200 min of monitoring, water was added to each solution at an NBD-to-water molar ratio of 1:20, and kinetic measurements of the thermal back-conversion continued. The values of *t*_1/2_, *E*_a_, Δ*H*^‡^ and Δ*S*^‡^ for each stage were determined according to Equations S1-S4.

#### Slow Box to Fast Box

Solutions of NBD/QC pairs were first prepared in anhydrous MeCN (0.1 mM), and water was then introduced to achieve an NBD-to-water molar ratio of 1:20. The mixtures were heated to 60 °C, and the absorbance of NBD at its maximum wavelength was recorded using the same UV-Vis spectroscopic setup. The samples were irradiated under UV light (*λ* = 315 nm, mounted LED, minimum output 53 mW, 700 mA, Thorlabs) until PSS was reached, followed by irradiation removal to initiate thermal back-conversion. After 7100 min, the solutions were taken out of the holder and rapidly cooled to room temperature. MeCN and water were removed under reduced pressure at 30 °C for approximately 10 min (Biotage V10 Touch). The dried residues were re-dissolved in anhydrous MeCN of the same volume, and thermal back-conversion kinetics were measured again. The values of *t*_1/2_, *E*_a_, Δ*H*^‡^ and Δ*S*^‡^ for each stage were determined according to Equations S1-S4.

### Selective transport of backconversion dynamics

In order to demonstrate selective transport behavior, we mixed two types of NBD/QC pairs in one solution. Solutions of **NBD6**/**QC6** and NBD2/QC2 pairs were prepared in anhydrous MeCN (0.1 mM) and heated to 60 °C. The absorbance at 315 nm was monitored using a home-built UV-Vis spectroscopic setup. Samples were irradiated under UV light (*λ* = 315 nm, mounted LED, minimum output 53 mW, 700 mA, Thorlabs) until PSS was reached, after which irradiation was stopped to initiate the thermal back-conversion process. After 250 min of monitoring, water was added to each solution at an NBD-to-water molar ratio of 1:20, and kinetic measurements of the thermal back-conversion continued. The values of *t*_1/2_, *E*_a_, Δ*H*^‡^ and Δ*S*^‡^ of **NBD6**/**QC6** and **NBD2**/**QC2** pairs for each stage were determined according to Equations S1-S4.

### Multi-box control of backconversion dynamics

Compared to traditional selective transport systems involving two compartments, we demonstrate a multi-compartment system regulated by hydrogen-bonding interactions. The NBD-to-water molar ratio change is a direct way to control the backconversion dynamics, which can be seen as like an infinitely box splitting. To illustrate this, we designed an experiment with various NBD-to-water molar ratios as described below.

#### Multi-box experiment from Fast Box to Slow Box

Solutions of **NBD6**/**QC6** were prepared in anhydrous MeCN (0.1 mM) and heated to 60 °C. The absorbance at the maximum wavelength of NBD was monitored using a home-built UV-Vis spectroscopic setup. Samples were irradiated under UV light (*λ* = 315 nm, mounted LED, minimum output 53 mW, 700 mA, Thorlabs) until PSS was reached, after which irradiation was stopped to initiate the thermal back-conversion process. After 78 min of monitoring, water was added to solution at an NBD-to-water molar ratio of 1:3, and kinetic measurements of the thermal back-conversion continued. Then at 480 and 1642 min, water was added to solution at an NBD-to-water molar ratio of 1:5 and 1:20, respectively. The values of *t*_1/2_, *E*_a_, Δ*H*^‡^ and Δ*S*^‡^ for each stage were determined according to Equations S1-S4.

### Multi-box experiment from Slow Box to Fast Box

Solutions of **NBD6**/**QC6** were reused following above experiment and heated to 60 °C. The absorbance at the maximum wavelength of NBD was monitored using a home-built UV-Vis spectroscopic setup. Samples were irradiated under UV light (*λ* = 315 nm, mounted LED, minimum output 53 mW, 700 mA, Thorlabs) until PSS was reached, after which irradiation was stopped to initiate the thermal back-conversion process. After 340 min of monitoring, 3/4 of the solution was taken out of the holder and rapidly cooled to room temperature. MeCN and water were removed under reduced pressure at 30 °C for approximately 10 min (Biotage V10 Touch). The dried residues were re-dissolved in anhydrous MeCN of the same volume, and thermal back-conversion kinetics were measured again. Then at 580 and 780 min, 3/4 and all solutions were taken out of the holder and rapidly cooled to room temperature, respectively. The dried residues were re-dissolved in anhydrous MeCN of the same volume, and thermal back-conversion kinetics were measured again. The values of *t*_1/2_, *E*_a_, Δ*H*^‡^ and Δ*S*^‡^ for each stage were determined according to Equations S1-S4.

#### Half-life testing

Solutions of NBD/QC pairs were first prepared in anhydrous MeCN (0.1 mM), and water was then introduced to achieve various NBD-to-water molar ratios (e.g., 1:3, 1:5, 1:20). The samples were irradiated until they reached PSS under UV light (*λ* = 315 nm, minimum 53 mW Mounted LED, 700 mA, Thorlabs), and then they followed the thermal back-conversion kinetics method (see in Supplementary Section 2.4.4). The values of *t*_1/2_, *E*_a_, Δ*H*^‡^ and Δ*S*^‡^ for each stage were determined according to Eqs. S1–S4.

## Supplementary information

Supplementary Information

Description of Additional Supplementary Files

Supplementary Movie 1

Supplementary Movie 2

Supplementary Movie 3

Peer Review File

## Data Availability

The main data generated in this study are provided in the article and Supplementary Information. The raw data and Cartesian coordinates of all optimized structures generated in this study have been deposited in the Zenodo database, https://zenodo.org/records/21409447. All data are available from the corresponding author upon request.
