# Supplementary material for: Orchestrating photoswitch thermal backconversion using hydrogen bonds to reshape energy landscapes
Source: Nat Commun. 2026 Aug 22;17:10063. doi: 10.1038/s41467-026-76898-2 (PMC13594139; doi:10.1038/s41467-026-76898-2)
Supplement: Supplementary file 1 [file 41467_2026_76898_MOESM1_ESM.pdf]

Supplementary Information

Orchestrating Photoswitch Thermal Backconversion using Hydrogen Bonds to Reshape Energy Landscapes

Liang Fei[1], Zacharias Liasi[2], Jacob L. Elholm[1], Helen Hölzel[1,3], D. Flemming Hansen[4], Kurt V. Mikkelsen[2]* and Kasper Moth-Poulsen[1,5,6,7]*

1 Department of Chemical Engineering, Universitat Politècnica de Catalunya, EEBE, Eduard Maristany 10-14, 08019 Barcelona, Spain
2 Department of Chemistry, University of Copenhagen, Universitetsparken 5, 2100 Copenhagen, Denmark
3 Institute of Organic Chemistry, Justus-Liebig-University Giessen, Heinrich-Buff-Ring 17, 35392 Giessen, Germany
4 Division of Biosciences, Department of Structural and Molecular Biology, University College London, London WC1E 6BT, United Kingdom
5 Department of Chemistry and Chemical Engineering, Chalmers University of Technology, SE-412 96 Gothenburg, Sweden
6 The Institute of Materials Science of Barcelona, ICMAB-CSIC, Bellaterra, 08193 Barcelona, Spain
7 Catalan Institution for Research & Advanced Studies, ICREA, Pg. Lluís Companys 23, 08010 Barcelona, Spain
*E-mail: kmi@chem.ku.dk; kasper.moth-poulsen@upc.edu

Table of Contents

1. Maxwell's demon-inspired system....3

1.1 What is Maxwell's Demon (Suppl. Note 1)....3

1.2 How does it inspire in this study (Suppl. Note 2)....4

2. Experimental Procedures....5

2.1 Materials (Suppl. Note 3)....5

2.2 Molecular synthesis (Suppl. Note 4)....5

2.3 Computational Modelling Approach (Suppl. Note 5)....10

2.4 Characterization and Methods (Suppl. Note 6)....11

2.4.1 Structural characterization....11

2.4.2 Photostationary state measurement....11

2.4.3 Hydrogen bonds characterization....12

2.4.4 Thermal back-conversion kinetics....12

2.5 Maxwell's demon experiments (Suppl. Note 7)....15

2.6 NMR spectra....16

2.6.1 NMR of NBD-CN,Br....16

2.6.2 NMR of NBD1....17

2.6.3 NMR of NBD2....18

2.6.4 NMR of NBD3....19

2.6.5 NMR of ethyl 3-bromopropiolate....20

2.6.6 NMR of NBD-Ester,Br....21

2.6.7 NMR of NBD4....22

2.6.8 NMR of NBD5....23

2.6.9 NMR of NBD6....24

3. Results and Discussion....25

3.1 Molecular design (Suppl. Note 8)....25

3.2 Thermal backconversion dynamics..30

3.2.1 Thermal back conversion of NBD/QC pairs in MeCN..30

3.2.2 Selectively control thermal backconversion...49

3.2.4 Thermal back conversion of NBD6/QC6 pair in various solvents.............................51

3.3 The reversible hydrogen bonding systems 59

4. Theoretical calculation...63

4.1 Global conformer searches (Suppl. Note 8)...63

4.2 Dihedral angle scan and energy landscape (Suppl. Note 9)..68

4.3 Molecular dynamics simulations (Suppl. Note 10) 76

References 81

1. Maxwell's demon-inspired system

1.1 What is Maxwell's Demon (Suppl. Note 1)

Maxwell's demon is a thought experiment introduced by James Clerk Maxwell in 1867 to probe the foundations of the Second Law of Thermodynamics. The scenario considers a container of gas divided into two chambers by a wall with a small door controlled by an intelligent "demon" (**Fig. S1**). By selectively allowing only fast-moving molecules to pass in one direction and slow-moving molecules in the other, the demon could, in principle, create a temperature gradient between the two chambers without performing any macroscopic work. This apparent decrease in entropy challenged the conventional understanding that the entropy of an isolated system never spontaneously decreases, leading to a long-standing paradox in thermodynamics.

The paradox was resolved through the recognition that the demon itself must acquire, store, and process information about individual molecules. Szilard (1929) first formalized the connection between information and thermodynamic entropy, and later, Landauer (1961) demonstrated that the act of erasing information incurs a minimum thermodynamic cost in the form of heat dissipation. Accounting for this information-processing cost restores compliance with the Second Law, revealing that information is fundamentally a physical quantity with energetic consequences.

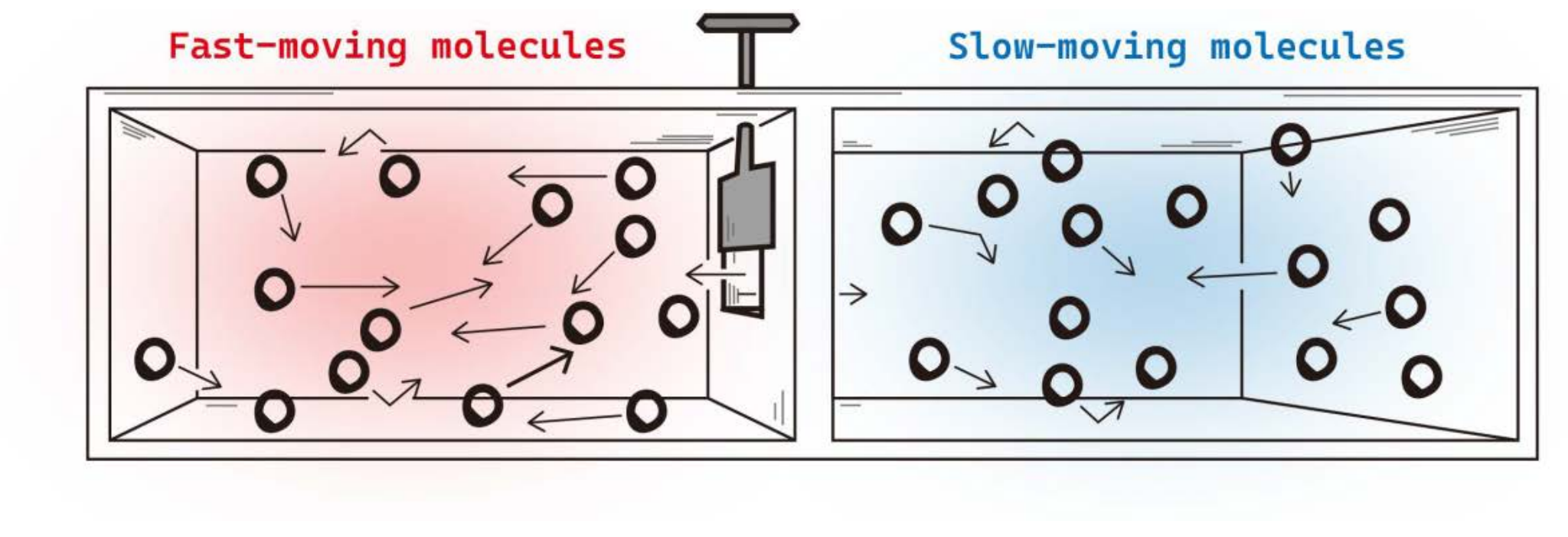

Fig. S1. Concept of Maxwell's Demon system.

In recent decades, Maxwell's demon has inspired experimental and theoretical research at the intersection of thermodynamics, information theory, and nanoscience.[1-9] Nanoscale "information engines" have been constructed, for example, by using optical traps to manipulate colloidal particles, where feedback based on measurements can extract work from thermal fluctuations. Stochastic thermodynamics provides a theoretical framework for quantifying such processes, particularly in small systems dominated by thermal noise. Extending the concept into the quantum regime, "quantum demons" exploit measurement, feedback, and entanglement to manipulate quantum states, demonstrating that information can serve as a resource for work even in quantum systems. Beyond fundamental studies, these insights have practical implications for the design of energy-efficient computation approaching

Landauer's limit, the operation of molecular machines, and nanoscale energy harvesting technologies. Maxwell's demon thus serves as a bridge connecting classical thermodynamics, information theory, and modern nanoscience, illustrating that the physical processing of information is inherently tied to energetic and entropic costs.

1.2 How does it inspire in this study (Suppl. Note 2)

This concept offers a powerful analogy for understanding and designing photoresponsive molecular systems where energy is stored in metastable isomers and released through thermal back-conversion. The norbornadiene (NBD)/quadricyclane (QC) photoisomerization system provides an archetypal platform for studying such principles: light excitation induces the conversion of NBD to QC, while QC's thermally activated reversion to NBD allows back-conversion structural changes. The tunability of thermal back-conversion kinetics in NBD derivatives demonstrates how molecular-level information can be encoded to control energy landscapes. This concept also can be broadly applicable to diverse photoswitches such as azobenzenes, diarylethenes, spiropyrans, and related chromophores used in photopharmacology, molecular actuation, and data storage.

In this framework, chemical substituents and supramolecular environments act as "molecular demons," encoding structural information that selectively modulates activation barriers and reaction kinetics. For example, substituents with electronic or steric effects, and supramolecular system (*i.e.* hydrogen-bonding networks) tune the enthalpic and entropic components of the QC-to-NBD isomerization, allowing fine control over storage half-lives ranging from hours to years. Furthermore, water in hydrogen-bonding networks can also act as environmental "feedback mechanisms", effectively "opening" or "closing" thermodynamic gates to regulate molecular energy flow. This active control echoes Maxwell's demon's selective sorting of particles, underscoring the role of chemical design as a form of information processing at the molecular level.

By positioning NBD photoisomerization chemistry within the broader paradigm of information thermodynamics, this analogy provides a unifying framework for molecular design: supramolecular hydrogen-bonding gates encode environmental information and direct reaction kinetics, allowing precise spatiotemporal control of back-conversion dynamics across multiple application spaces. While NBD/QC chemistry has been central to molecular solar energy research, the same design principles, including the strategic encoding of information through hydrogen bonds, and substituent effects, extend naturally to other classes of photoswitches. This perspective highlights how hydrogen-bonding-mediated regulation can be integrated with modern supramolecular chemistry, opening pathways toward adaptive and programmable photoresponsive materials that combine energy storage, actuation, and sensing within a single molecular platform.

2. Experimental Procedures

2.1 Materials (Suppl. Note 3)

Solvents of acetonitrile (MeCN, extra dry, 99.9%, over molecular sieve, AcroSeal®), toluene (extra dry, anhydrous, 99.8%, over molecular sieve, packaged under Argon in resealable ChemSeal™ bottle), 1,4-dioxane (extra dry, 99.5%, over molecular sieve, stabilized, AcroSeal®), n-hexane (97%, extra dry over molecular sieve, AcroSeal®), isopropanol (99.5%, extra dry over molecular sieve, AcroSeal®), N,N-Dimethylformamide (DMF, 99.8%, extra dry over molecular sieve, AcroSeal®), acetone (99.8%), ethyl acetate (EtOAc, 99%), petroleum spirit (40-60 °C), n-hexane (99.8%), dichloromethane (DCM, 99.8%), chlorobenzene (99.8%), tetrahydrofuran (99.8%), acetonitrile-*d*3 (CD_3CN, 99.8 atom % D), and Chloroform-*d* ($CDCl_3$, 99.8 atom % D) were bought from Thermo Fisher Scientific. The phenylboronic acid (98%), 4-methoxyphenylboronic acid (98%), 4-acetamidophenylboronic acid (95%), Copper(I) cyanide (CuCN, 99%), Iron(III) chloride ($FeCl_3$, 98%), sodium thiosulfate (Na_2SO_4, 99+%), N-Bromosuccinimide (NBS, 99%), potassium carbonate (99%), silver nitrate ($AgNO_3$, 99+%), 2-dicyclohexylphosphino-2',6'-diisopropoxy-1,1'-biphenyl (RuPhos, 98%), palladium(II) acetate ($[Pd(OAc)_2]_3$, 98%), ammonium chloride (99.5%), and magnesium sulfate ($MgSO_4$, anhydrous, 99.5%) were used as received from Sigma-Aldrich. Deionized water was obtained from a Milli-Q purification system (Millipore) and used in all experiments.

2.2 Molecular synthesis (Suppl. Note 4)

All final norbornadiene (NBD) derivatives were synthesized by the Suzuki-Miyaura coupling reaction[10] (Fig. S2). The organohalides were 3-bromobicyclo[2.2.1]hepta-2,5-diene-2-carbonitrile (**NBD-CN,Br**) and ethyl-3-bromobicyclo[2.2.1]hepta-2,5-diene-2-carboxylate (**NBD-Ester,Br**), which were homemade. The boronic acids were phenylboronic acid, 4-methoxyphenylboronic acid, and 4-acetamidophenylboronic acid from Sigma-Aldrich.

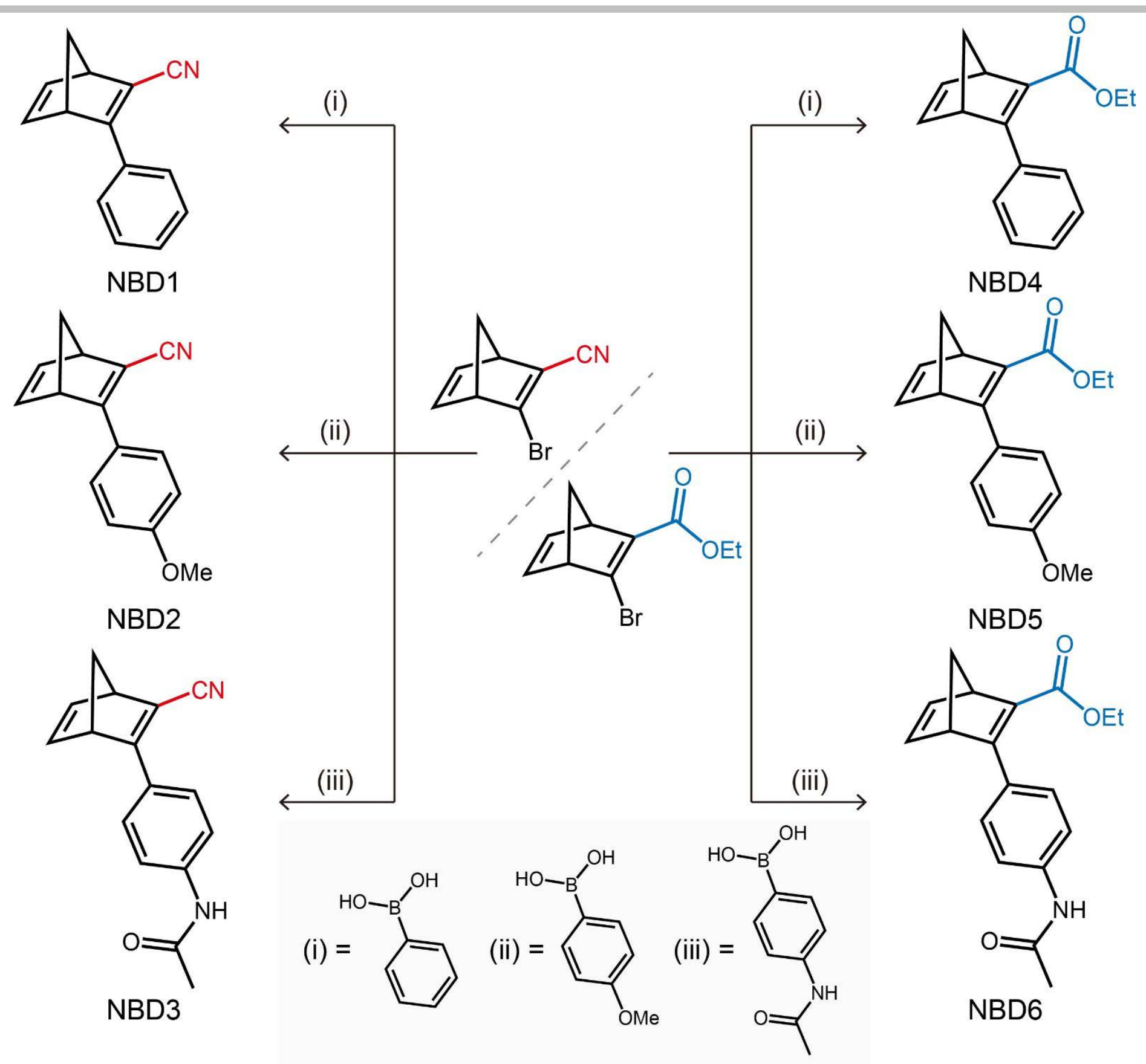

Fig. S2. Synthesis of NBDs by the Suzuki reaction.

NBD-CN,Br was synthesized as following:

A suspension of dibromo NBD (1.25 g, 5.0 mmol, 1.0 equiv.) and CuCN (0.49 g, 5.5 mmol, 1.1 equiv.) in anhydrous DMF (20 mL) was prepared. The reaction mixture was heated to 115 °C and stirred for 1 hour. Following this, the mixture was quenched with a 10% aqueous $FeCl_3$ solution and extracted using a 1:9 mixture of EtOAc and hexane. The combined organic layers were dried over Na_2SO_4, filtered, and concentrated under reduced pressure to remove the solvent. The reaction mixture was purified using a BUCHI Pure C-815 Flash system equipped with a BUCHI FlashPure EcoFlex Silica 12 g column, employing a 1:9 mixture of EtOAc and hexane as the eluent. This process afforded NBD-CN,Br with a yield of 15% (0.15 g). ^{1}H NMR (400 MHz, $CDCl_3$) δ 6.93 – 6.89 (m, 1H), 6.89 – 6.84 (m, 1H), 3.92 – 3.86 (m, 1H), 3.75 (dddt, *J* = 3.8, 3.0, 2.2, 1.0 Hz, 1H), 2.41 (dt, *J* = 7.0, 1.7 Hz, 1H), 2.24 (dt, *J* = 6.9, 1.7 Hz, 1H). ^{13}C NMR (101 MHz, $CDCl_3$) δ 154.10, 142.40, 140.50, 124.83, 115.39, 73.10, 60.00, 54.82. HRMS (ESI) m/z: $[M + H]^+$ calcd for C_8H_7BrN, 196.9663; found, 196.9661.

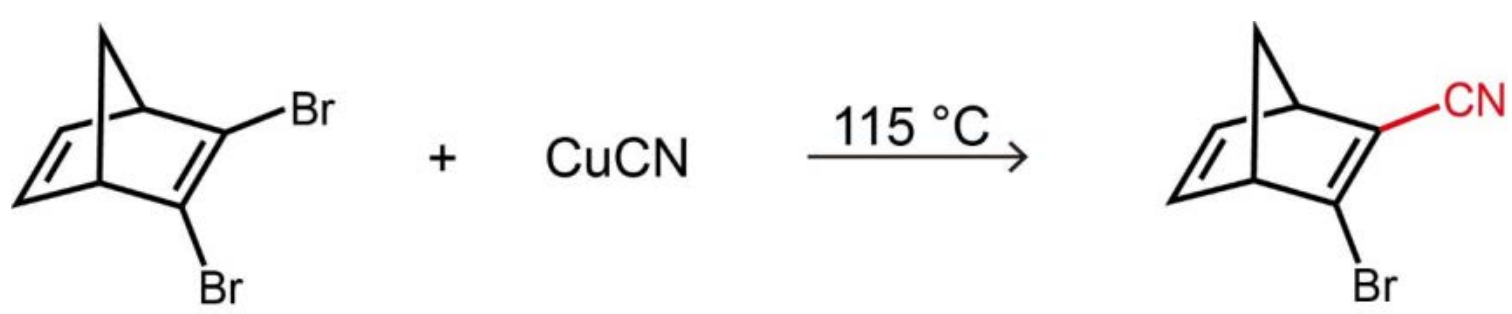

Fig. S3. Synthesis of **NBD-CN,Br**.

NBD-Ester,Br was synthesized as following:

A solution of ethyl propiolate (0.49 g, 5.0 mmol, 1.0 equiv.) in acetone (5 mL) was reacted with NBS (0.98 g, 5.5 mmol, 1.1 equiv.) and $AgNO_3$ (0.084 g, 10 mol%) at room temperature under magnetic stirring. After 2 hours, the reaction mixture was diluted with hexane (100 mL), and the precipitated crystals were removed by filtration. The filtrate was concentrated under reduced pressure and purified by passing through a silica gel column using hexanes as the eluent. The product, **ethyl 3-bromopropiolate**, was obtained as a pure, colorless oil in 79% yield (0.70 g). ^{1}H NMR (400 MHz, $CDCl_3$) δ 4.27 (q, *J* = 7.1 Hz, 2H), 1.34 (t, *J* = 7.1 Hz, 3H). ^{13}C NMR (101 MHz, Chloroform-*d*) δ 152.45, 72.83, 62.45, 52.35, 13.94. HRMS (ESI) m/z: $[M + H]^+$ calcd for $C_5H_5BrO_2$, 176.9545; found, 176.9534.

Dicyclopentadiene was placed in a round-bottom flask equipped with a short distillation column and condenser, and the system was purged with a gentle flow of nitrogen to maintain an inert atmosphere. The flask was immersed in an oil bath preheated to 185 °C, and the mixture was allowed to reflux, promoting retro-Diels–Alder cleavage to generate **cyclopentadiene** monomer. The liberated cyclopentadiene was distilled as it formed and collected in an ice-cooled receiver. The product, cyclopentadiene was obtained as a white crystal in 90% yield, and stored into freezer to avoid repolymerization.

Cyclopentadiene (0.59 g, 9.0 mmol, 3.0 equiv.), ethyl 3-bromopropiolate (0.53 g, 3.0 mmol, 1.0 equiv.), and chlorobenzene were added to a flask. The flask was sealed and heated at 40 °C for 24 hours. Upon completion of the reaction, the mixture was purified using a BUCHI Pure C-815 Flash system equipped with a BUCHI FlashPure EcoFlex Silica 12 g column, employing a 1:8 mixture of a DCM and petroleum spirit (1:1) as the eluent. The product, **NBD-Ester,Br**, was obtained as a pure, light yellow liquid in 78% yield (0.57 g). ^{1}H NMR (400 MHz, $CDCl_3$) δ 6.91 (dd, *J* = 5.1, 3.0 Hz, 1H), 6.85 (dd, *J* = 4.9, 3.2 Hz,1H), 4.22 (dd, *J* = 7.2, 6.0 Hz, 2H), 4.00 (dp, *J* = 3.0, 1.5 Hz, 1H), 3.68 (td, *J* = 3.0, 2.4, 1.4 Hz, 1H), 2.32 (dt, *J* = 6.7, 1.7 Hz, 1H), 2.12 (dt, *J* = 6.7, 1.7 Hz, 1H), 1.32 (t, *J* = 7.1 Hz, 3H). ^{13}C NMR (101 MHz, $CDCl_3$) δ 163.83, 148.44, 143.09, 142.15, 140.45, 71.76, 61.71, 60.52, 52.11, 14.27. HRMS (ESI) m/z: $[M + H]^+$ calcd for $C_{10}H_{11}BrO_2$, 243.0015; found, 243.0006.

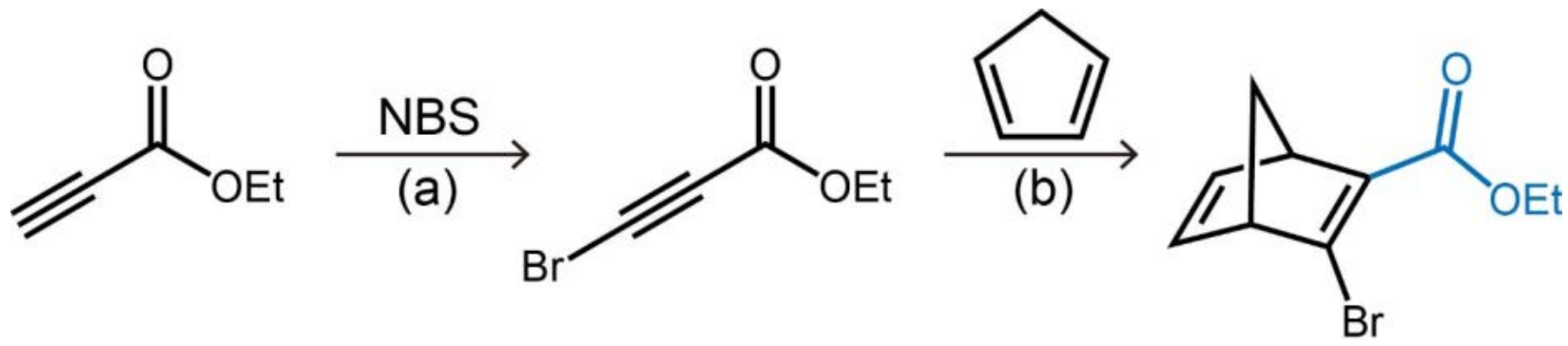

Fig. S4. Synthesis of **NBD-Ester,Br**.

NBD1 was synthesized as following:

A nitrogen-degassed solution of **NBD-CN,Br** (0.20 g, 1.0 mmol, 1.0 equiv.) in toluene (15 mL) was added to a pre-degassed reaction flask containing phenylboronic acid (0.15 g, 1.2 mmol, 1.2 equiv.), potassium carbonate (K_2CO_3, 0.41 g, 3.0 mmol, 3.0 equiv.), RuPhos (0.047 g, 10 mol%), and $[Pd(OAc)_2]_3$ (0.011 g, 4.8 mol%). Degassed

water (10 mL) was then added, creating a biphasic mixture. The reaction was stirred at 100 °C and monitored by TLC. Once complete, the mixture was allowed to cool to room temperature, quenched with saturated aqueous ammonium chloride (NH_4Cl, 20 mL), and diluted with an additional 20 mL of water. The resulting mixture was extracted with toluene (3 × 20 mL), and the combined organic layers were dried over $MgSO_4$, filtered, and concentrated under reduced pressure. The reaction mixture was purified using a BUCHI Pure C-815 Flash system equipped with a BUCHI FlashPure EcoFlex Silica 12 g column, employing a 1:7 mixture of EtOAc and hexane as the eluent. The product, **NBD1**, was obtained as a pure, colorless oil in 43% yield (0.083 g). ^{1}H NMR (400 MHz, $CDCl_3$) δ 7.76 – 7.70 (m, 2H), 7.56 – 7.35 (m, 3H), 6.94 (dd, *J* = 5.1, 3.0 Hz, 1H), 6.87 (dd, *J* = 5.2, 3.2 Hz, 1H), 4.13 (s, 1H), 3.94 (s, 1H), 2.29 (dt, *J* = 6.9, 1.7 Hz, 1H), 2.20 (dt, *J* = 6.9, 1.7 Hz, 1H). ^{13}C NMR (101 MHz, $CDCl_3$) δ 170.85, 143.11, 140.35, 133.10, 130.14, 128.92, 126.46, 118.44, 116.93, 71.25, 55.00, 54.20. HRMS (ESI) m/z: $[M + H]^+$ calcd for $C_{14}H_{11}N$, 194.0925; found, 194.0913.

NBD2 was synthesized as following:

A nitrogen-degassed solution of **NBD-CN,Br** (0.20 g, 1.0 mmol, 1.0 equiv.) in toluene (15 mL) was added to a pre-degassed reaction flask containing 4-methoxyphenylboronic acid (0.18 g, 1.2 mmol, 1.2 equiv.), K_2CO_3 (0.41 g, 3.0 mmol, 3.0 equiv.), RuPhos (0.047 g, 10 mol%), and $[Pd(OAc)_2]_3$ (0.011 g, 4.8 mol%). Degassed water (10 mL) was then added, creating a biphasic mixture. The reaction was stirred at 100 °C and monitored by TLC. Once complete, the mixture was allowed to cool to room temperature, quenched with saturated aqueous ammonium chloride (NH_4Cl, 20 mL), and diluted with an additional 20 mL of water. The resulting mixture was extracted with toluene (3 × 20 mL), and the combined organic layers were dried over $MgSO_4$, filtered, and concentrated under reduced pressure. The reaction mixture was purified using a BUCHI Pure C-815 Flash system equipped with a BUCHI FlashPure EcoFlex Silica 12 g column, employing a 1:8 mixture of EtOAc and hexane as the eluent. The product, **NBD2**, was obtained as a pure, light yellow solid in 65% yield (0.14 g). ^{1}H NMR (400 MHz, CD_3CN) δ 7.81 – 7.68 (m, 2H), 7.09 – 7.02 (m, 2H), 6.98 (dd, *J* = 2.9, 0.8 Hz, 1H), 6.93 (dd, *J* = 3.1, 1.0 Hz, 1H), 4.22 – 4.15 (m, 1H), 3.96 – 3.90 (m, 1H), 3.86 (s, 3H), 2.25 (dt, *J* = 6.8, 1.7 Hz, 1H), 2.14 (dt, *J* = 6.8, 1.7 Hz, 1H). ^{13}C NMR (101 MHz, CD_3CN) δ 170.71, 161.23, 143.10, 140.43, 128.14, 125.91, 118.67, 114.34, 113.84, 70.57, 55.19, 54.75, 54.19. HRMS (ESI) m/z: $[M + H]^+$ calcd for $C_{15}H_{13}NO$, 224.1070; found, 224.1061.

NBD3 was synthesized as following:

A nitrogen-degassed solution of **NBD-CN,Br** (0.20 g, 1.0 mmol, 1.0 equiv.) in tetrahydrofuran (10 mL) was added to a pre-degassed reaction flask containing 4-acetaminophenylboronic acid (0.21 g, 1.2 mmol, 1.2 equiv.), K_2CO_3 (0.41 g, 3.0 mmol, 3.0 equiv.), RuPhos (0.047 g, 10 mol%), and $[Pd(OAc)_2]_3$ (0.011 g, 4.8 mol%). Degassed water (6 mL) was then added, creating a biphasic mixture. The reaction was stirred at 100 °C and monitored by TLC.

Once complete, the mixture was allowed to cool to room temperature, quenched with saturated aqueous ammonium chloride (NH_4Cl, 20 mL), and diluted with an additional 20 mL of water. The resulting mixture was extracted with tetrahydrofuran (3 × 20 mL), and the combined organic layers were dried over $MgSO_4$, filtered, and concentrated under reduced pressure. The reaction mixture was purified using a BUCHI Pure C-815 Flash system equipped with a BUCHI FlashPure EcoFlex Silica 12 g column, employing a 1:3 mixture of EtOAc and hexane as the eluent. The product, **NBD3**, was obtained as a pure, yellow solid in 23% yield. ^{1}H NMR (400 MHz, $CDCl_3$) δ 7.74 (d, *J* = 8.8 Hz, 2H), 7.63 (d, *J* = 8.8 Hz, 2H), 7.53 (s, 1H), 6.96 (dd, *J* = 5.2, 2.9 Hz, 1H), 6.86 (dd, *J* = 5.4, 2.8 Hz, 1H), 4.14 (s, 1H), 3.95 (s, 1H), 2.33 – 2.26 (m, 1H), 2.26 – 2.17 (m, 4H). ^{13}C NMR (101 MHz, $CDCl_3$) δ 170.28, 168.45, 143.17, 140.13, 139.69, 128.83, 127.48, 119.51, 115.18, 114.71, 70.97, 54.85, 54.00, 29.72. HRMS (ESI) m/z: $[M + H]^+$ calcd for $C_{16}H_{15}N_2O$, 251.1184; found, 251.1083. $[M + Na]^+$ calcd for $C_{16}H_{14}N_2ONa$, 273.1004; found, 273.1003.

NBD4 was synthesized as following:

A nitrogen-degassed solution of **NBD-Ester,Br** (0.24 g, 1.0 mmol, 1.0 equiv.) in toluene (15 mL) was added to a pre-degassed reaction flask containing phenylboronic acid (0.15 g, 1.2 mmol, 1.2 equiv.), K_2CO_3 (0.41 g, 3.0 mmol, 3.0 equiv.), RuPhos (0.047 g, 10 mol%), and $[Pd(OAc)_2]_3$ (0.011 g, 4.8 mol%). Degassed water (10 mL) was then added, creating a biphasic mixture. The reaction was stirred at 100 °C and monitored by TLC. Once complete, the mixture was allowed to cool to room temperature, quenched with saturated aqueous ammonium chloride (NH_4Cl, 20 mL), and diluted with an additional 20 mL of water. The resulting mixture was extracted with toluene (3 × 20 mL), and the combined organic layers were dried over $MgSO_4$, filtered, and concentrated under reduced pressure. The reaction mixture was purified using a BUCHI Pure C-815 Flash system equipped with a BUCHI FlashPure EcoFlex Silica 12 g column, employing a 1:8 mixture of EtOAc and hexane as the eluent. The product, **NBD4**, was obtained as a pure, light yellow solid in 51% yield (0.12 g). ^{1}H NMR (400 MHz, $CDCl_3$) δ 7.60 (dd, *J* = 6.8, 1.7 Hz, 2H), 7.45 – 7.32 (m, 3H), 7.06 (dd, *J* = 5.1, 2.9 Hz, 1H), 6.98 (dd, *J* = 5.1, 2.9 Hz, 1H), 4.20 (d, *J* = 7.1 Hz, 2H), 4.14 (s, 1H), 3.91 (s, 1H), 2.32 (d, *J* = 6.6 Hz, 1H), 2.12 (d, *J* = 6.6 Hz, 1H), 1.28 (t, *J* = 7.2 Hz, 3H). ^{13}C NMR (101 MHz, $CDCl_3$) δ 166.36, 165.51, 143.75, 140.90, 139.39, 135.78, 128.54, 127.90, 70.59, 60.11, 58.57, 53.13, 14.22. HRMS (ESI) m/z: $[M + H]^+$ calcd for $C_{16}H_{16}O_2$, 241.1184; found, 241.1189.

NBD5 was synthesized as following:

A nitrogen-degassed solution of **NBD-Ester,Br** (0.24 g, 1.0 mmol, 1.0 equiv.) in toluene (15 mL) was added to a pre-degassed reaction flask containing 4-methoxyphenylboronic acid (0.18 g, 1.2 mmol, 1.2 equiv.), K_2CO_3 (0.41 g, 3.0 mmol, 3.0 equiv.), RuPhos (0.047 g, 10 mol%), and $[Pd(OAc)_2]_3$ (0.011 g, 4.8 mol%). Degassed water (10 mL) was then added, creating a biphasic mixture. The reaction was stirred at 100 °C and monitored by TLC. Once

complete, the mixture was allowed to cool to room temperature, quenched with saturated aqueous ammonium chloride (NH_4Cl, 20 mL), and diluted with an additional 20 mL of water. The resulting mixture was extracted with toluene (3 × 20 mL), and the combined organic layers were dried over $MgSO_4$, filtered, and concentrated under reduced pressure. The reaction mixture was purified using a BUCHI Pure C-815 Flash system equipped with a BUCHI FlashPure EcoFlex Silica 12 g column, employing a 1:7 mixture of EtOAc and hexane as the eluent. The product, **NBD5**, was obtained as a pure, light yellow solid in 46% yield (0.12 g). ^{1}H NMR (400 MHz, Acetonitrile-*d*$_3$) δ 7.56 (d, *J* = 8.9 Hz, 2H), 6.95 (t, *J* = 1.9 Hz, 1H), 6.92 (d, *J* = 8.9 Hz, 2H), 4.15 – 4.06 (m, 2H), 3.99 (h, *J* = 1.7 Hz, 1H), 3.88 (h, *J* = 1.7 Hz, 1H), 3.81 (s, 3H), 2.18 (t, *J* = 1.7 Hz, 1H), 2.03 – 1.96 (m, 1H), 1.20 (t, *J* = 7.1 Hz, 3H). ^{13}C NMR (101 MHz, Acetonitrile-*d*$_3$) δ 167.15, 160.74, 144.04, 141.53, 137.76, 130.35, 128.57, 113.72, 70.46, 60.44, 58.74, 55.63, 53.68, 14.18. HRMS (ESI) m/z: $[M + H]^+$ calcd for $C_{17}H_{19}O_3$, 271.1334; found, 271.1328. $[M + Na]^+$ calcd for $C_{17}H_{18}O_3Na$, 293.1154; found, 293.1154.

NBD6 was synthesized as following:

A nitrogen-degassed solution of **NBD-Ester,Br** (0.24 g, 1.0 mmol, 1.0 equiv.) in tetrahydrofuran (10 mL) was added to a pre-degassed reaction flask containing 4-acetaminophenylboronic acid (0.21 g, 1.2 mmol, 1.2 equiv.), K_2CO_3 (0.41 g, 3.0 mmol, 3.0 equiv.), RuPhos (0.047 g, 10 mol%), and $[Pd(OAc)_2]_3$ (0.011 g, 4.8 mol%).Degassed water (6 mL) was then added, creating a biphasic mixture. The reaction was stirred at 100 °C and monitored by TLC. Once complete, the mixture was allowed to cool to room temperature, quenched with saturated aqueous ammonium chloride (NH_4Cl, 20 mL), and diluted with an additional 20 mL of water. The resulting mixture was extracted with tetrahydrofuran (3 × 20 mL), and the combined organic layers were dried over $MgSO_4$, filtered, and concentrated under reduced pressure. The reaction mixture was purified using a BUCHI Pure C-815 Flash system equipped with a BUCHI FlashPure EcoFlex Silica 12 g column, employing a 1:3 mixture of EtOAc and hexane as the eluent. The product, **NBD6**, was obtained as a pure, yellow solid in 28% yield (0.083 g). ^{1}H NMR (400 MHz, $CDCl_3$) δ 7.79 (s, 1H), 7.52 (d, *J* = 3.4 Hz, 4H), 6.99 (dd, *J* = 5.2, 2.9 Hz, 1H), 6.91 (dd, *J* = 5.1, 2.9 Hz, 1H), 4.17 (qd, *J* = 7.1, 2.6 Hz, 2H), 4.08 (s, 1H), 3.86 (s, 1H), 2.24 (d, *J* = 6.6 Hz, 1H), 2.15 (s, 3H), 2.07 (d, *J* = 6.7 Hz, 1H), 1.27 (t, *J* = 7.2 Hz, 3H). ^{13}C NMR (101 MHz, $CDCl_3$) δ 168.60, 166.27, 165.78, 143.68, 140.67, 138.41, 138.35, 131.30, 128.79, 118.81, 70.34, 60.17, 58.34, 53.04, 24.58, 14.23. HRMS (ESI) m/z: $[M + H]^+$ calcd for $C_{18}H_{19}NO_3$, 298.1438; found, 298.1429.

2.3 Computational Modelling Approach (Suppl. Note 5)

All calculations were carried out using Orca 6.0.1[11,12]and xtb 6.7.1[13]. For each NBD/QC derivative, initial structures were generated in vacuum and subjected to exhaustive conformer searches using GFN2-xTB[14] global optimization

(GOAT)[15] in Orca. GFN2-xTB-based climbing image nudged elastic band (CI-NEB)[16] searches were performed using the located global minima as endpoints. Saddle point refinement of the located maxima were carried out using the "zoom" methodology in Orca, followed by GFN2-xTB optimization.

The minimum-energy conformations from the vacuum search were used as initial structures for solvent placement via the SOLVATOR tool in Orca. Conformer searches were then performed using GOAT to locate minimum solvent configurations. Ground-state and transition-state structures of the core molecules were further optimized using ωB97X-D4/cc-pVTZ.[17-19] Relaxed surface scans were conducted for the core molecules by scanning the full rotation of the side groups using GFN2-xTB, followed by ωB97X-D4/cc-pVTZ optimization under geometric constraints.

The initial structures for the molecular dynamics simulations were set up using the SOLVATOR tool. All simulations were conducted in the canonical (NVT) ensemble at 298.15 K for 2.5 ns with a time step of 2 fs. An initial 500 ps equilibrating run was carried out prior to the production run. Bond lengths were constrained using the SHAKE algorithm (tolerance level 2),[20] while hydrogen atoms were subjected to 4-fold mass scaling. The accuracy of self-consistent charge calculations was set to 2.0. A spherical logfermi potential confined the entire system within the simulation box. Atomic coordinates were recorded every 1 ps for subsequent analysis. The GFN2-xTB method was used for the simulations via the xtb program.

2.4 Characterization and Methods (Suppl. Note 6)

2.4.1 Structural characterization.

^{1}H NMR (400 MHz) and ^{13}C NMR (100 MHz) spectra were recorded on a Bruker NMR Ascend 400 using the residual solvent as the internal standard ($CDCl_3$, ^{1}H 7.26 ppm and ^{13}C 77.16 ppm or CD_3CN, ^{1}H 1.94 ppm, ^{13}C 1.32 ppm, 118.26 ppm) at 25 °C. The following abbreviations (or combinations thereof) were used to explain multiplicities: s = singlet, d = doublet, t = triplet, q = quartet, m = multiplet, dd = double doublet, ddd = double double doublet, dt = double triplet, td = triple doublet. Coupling constants, *J*, were reported in Hertz unit (Hz). The high-resolution mass spectra (HRMS) were obtained by an Agilent 1260 Infinity fitted with an Agilent 6120 quadrupole using APCI mode for ionisation.

2.4.2 Photostationary state measurement.

NBDs kept in total darkness at 60 °C overnight prior to measurement. 30 mM solutions of NBDs in $CDCl_3$ or CD_3CN were prepared. The NBD solutions were illuminated by UV lamp (λ = 315 nm, minimum 53 mW Mounted LED, 700 mA, Thorlabs LED) positioned at a distance of 10 cm at room temperature (RT). Meanwhile, the ^{1}H NMR and UV-

Vis spectra (Shimadzu UV-3600) were recorded until the spectra had no impact with prolonged light irradiation, meaning that NBD-to-QC conversions reached the photostationary state (PSS).

Cycling test of NBDs in toluene. The NBD-to-QC conversion was under UV lamp (λ = 315 nm, minimum 53 mW Mounted LED, 700 mA, Thorlabs LED) until PSS, and the QC-to-NBD conversion was accelerated by heating (80 °C) until full backconversion. The alternative irradiation and thermal backconversion processes were repeated 20 cycles.

Cycling tests of NBDs. Solutions of NBD/QC pairs were prepared in anhydrous toluene (0.1 mM). The photoisomerization of NBD to QC was carried out under UV irradiation (λ = 315 nm, mounted LED, minimum output 53 mW, 700 mA, Thorlabs) until the photostationary state (PSS) was reached. Thermal back-conversion of QC to NBD was subsequently accelerated by heating at 80 °C until complete regeneration of NBD. This irradiation–heating sequence was repeated for 20 consecutive cycles to evaluate the cycling stability of the NBD/QC system.

2.4.3 Hydrogen bonds characterization.

All solvents were dried over molecular sieves prior to use. The sieves were activated by heating at 300-350 °C under vacuum for 8 h and stored in a sealed, dry nitrogen atmosphere. They were added to solvents at 20% (w/v) and left overnight under nitrogen to ensure effective dehydration. Solutions of NBDs or QCs (100 mM) were prepared in dried $CDCl_3$ or CD_3CN. The NBD solutions were subsequently mixed with water to achieve various NBD-to-water molar ratios (e.g., 2:1, 1:1, 2:3, 1:2, 1:5, 1:10, 1:20). Meanwhile, ^{1}H NMR (400 MHz), ^{13}C NMR (100 MHz), ^{1}H-^{1}H COSY, and ^{1}H-^{13}C HMBC spectra were recorded, and spectral changes were analyzed to probe hydrogen-bonding effects. *In situ* variable-temperature ^{1}H NMR experiments were conducted to monitor hydrogen-bonding interactions over the temperature range of 298-353 K.

Confocal Raman spectra of QC6 solution, MeCN, and water were acquired using a Renishaw inVia Reflex Raman spectrometer with 532 nm polarized excitation to investigate group-specific hydrogen bonding. Peak positions were determined by curve fitting with WiRE 5.3 software.

2.4.4 Thermal back-conversion kinetics

UV-Vis absorbance spectra of the thermal back-conversion kinetics were measured using a home-built UV-Vis spectroscopic platform.[21] The automated system can, for typical molecular systems, record the back-conversion kinetics in less than 24 hours. By determining the thermal rate constant at three different temperatures, we obtain the thermodynamic parameters of the thermal back-reaction such as activation energy (E_a), enthalpy ($\Delta H^{\ddagger}$) and entropy ($\Delta S^{\ddagger}$) through Eyring and Arrhenius analysis. Temperature control was achieved using Temperature-controlled cuvette holder (Quantum Northwest qPod 3e). Measurements were taken at 1 nm intervals from 500-

300 nm. The UV-Vis absorbance spectrum in the native state was first taken at 25 °C. Spectra were then taken at various temperatures (depended on the molecular structures).

All solvents were dried over molecular sieves prior to use. The samples were irradiated until they reached PSS under UV light (λ = 315 nm, minimum 53 mW Mounted LED, 700 mA, Thorlabs LED). All the curves are fitted by first-order kinetics equation as following:

$$[A(\lambda)](t) = [A_0(\lambda)]e^{-kt} \tag{S1}$$

where

$[A(\lambda)]$ = actual concentration

$[A_0(\lambda)]$ = initial concentration

k = reaction rate constant

t = reaction time

The measurements were performed at different temperatures and an exponential fit of the Arrhenius equation and Eyring equation was applied to the data to determine the apparent E_a, $\Delta H^‡$ and $\Delta S^‡$ of activation.

Arrhenius equation

$$\ln k = \frac{-E_a}{RT} + ln\frac{k_B T}{h} \tag{S2}$$

where

k = reaction rate constant

T = absolute temperature

R= gas constant

E_a = apparent activation energy

T =absolute temperature

k_B = Boltzmann constant

h = Planck' s constant

Eyring equation

$$\ln\frac{k}{T} = \frac{-\Delta H^‡}{R}\cdot\frac{1}{T} + ln\frac{k_B}{h} + \frac{\Delta S^‡}{R} \tag{S3}$$

where:

k = reaction rate constant

T =absolute temperature

R= gas constant

k_B = Boltzmann constant

h = Planck' s constant

$\Delta H^{\ddagger}$ = Enthalpy of activation

$\Delta S^{\ddagger}$ = Entropy of activation

The Gibbs free energy of activation is calculated from enthalpy and entropy.

$$\Delta G^{\ddagger} = \Delta H^{\ddagger} - T\Delta S^{\ddagger}$$

where:

$\Delta G^{\ddagger}$ = Gibbs free energy of activation

The half-life of the QC at a specific temperature corresponds to a moment where $[A(\lambda)]$ equals $[A_1(\lambda)]/2$. From equation S1, it can then be written as:

$$t_{1/2} = \frac{ln(2)}{k} \tag{S5}$$

where:

k = reaction rate constant

$t_{1/2}$ = half-life

Solvent change effect

In our experiments, the water content is very low. Even at the highest NBD-to-water molar ratio (1:20), the water concentration in acetonitrile is only 20 mM (0.0046 wt%) for UV–Vis and 600 mM (1.38 wt%) for NMR measurements.

Literature data[22] show that such low water contents (≤2 wt%) induce only minor changes in bulk solvent properties. For example, the density increases slightly from 0.777 to 0.781 kg dm^{-3} (0-2 wt% H_2O), while the dielectric constant changes modestly from 35.9 (pure MeCN) to 39.3 even at 9.9 wt% H_2O. The viscosity and refractive index change only marginally below 10 wt% water, increasing from 0.35 to 0.40 mPa·s and from 1.342 to 1.343, respectively.[23]

Therefore, under our conditions, macroscopic solvent effects are negligible. Instead, the observed behavior is more reasonably attributed to local hydrogen-bonding interactions, such as the formation of water clusters or structured solvation shells around the solute, which are expected to dominate over small changes in the bulk dielectric constant.

2.5 Maxwell's demon experiments (Suppl. Note 7)

In this study, our system is conceptualized as comprising two distinct compartments, termed the Fast Box and the Slow Box, which correspond to environments with different thermal back-conversion rates from QC to NBD. The transition between these compartments is regulated by dynamic hydrogen-bonding system. In the initial state, all molecules are located in the Fast Box, where the QC-to-NBD back-conversion pathway remains relatively flexible. A locked state is achieved when QC units establish hydrogen bonds with surrounding water molecules, which restricts molecular motion and effectively confines the molecules within the Slow Box.

For the series NBD1-6, two types of behavior can be observed. In an anhydrous situation, all NBD/QC pairs remain exclusively in the Fast Box because hydrogen-bonding interactions are absent, and no transition to the Slow Box occurs. Upon the introduction of water molecules, only selected compounds gain the ability to access the Slow Box. As intentionally designed, NBD1 and NBD2 do not contain functional groups capable of hydrogen bonding, and therefore cannot pass through the molecular gate that separates the two compartments (**Fig. S2**a). By contrast, NBD3-6 possess hydrogen-bonding sites, which enable them to undergo a reversible transition from the Fast Box to the Slow Box (**Fig. S2**b).

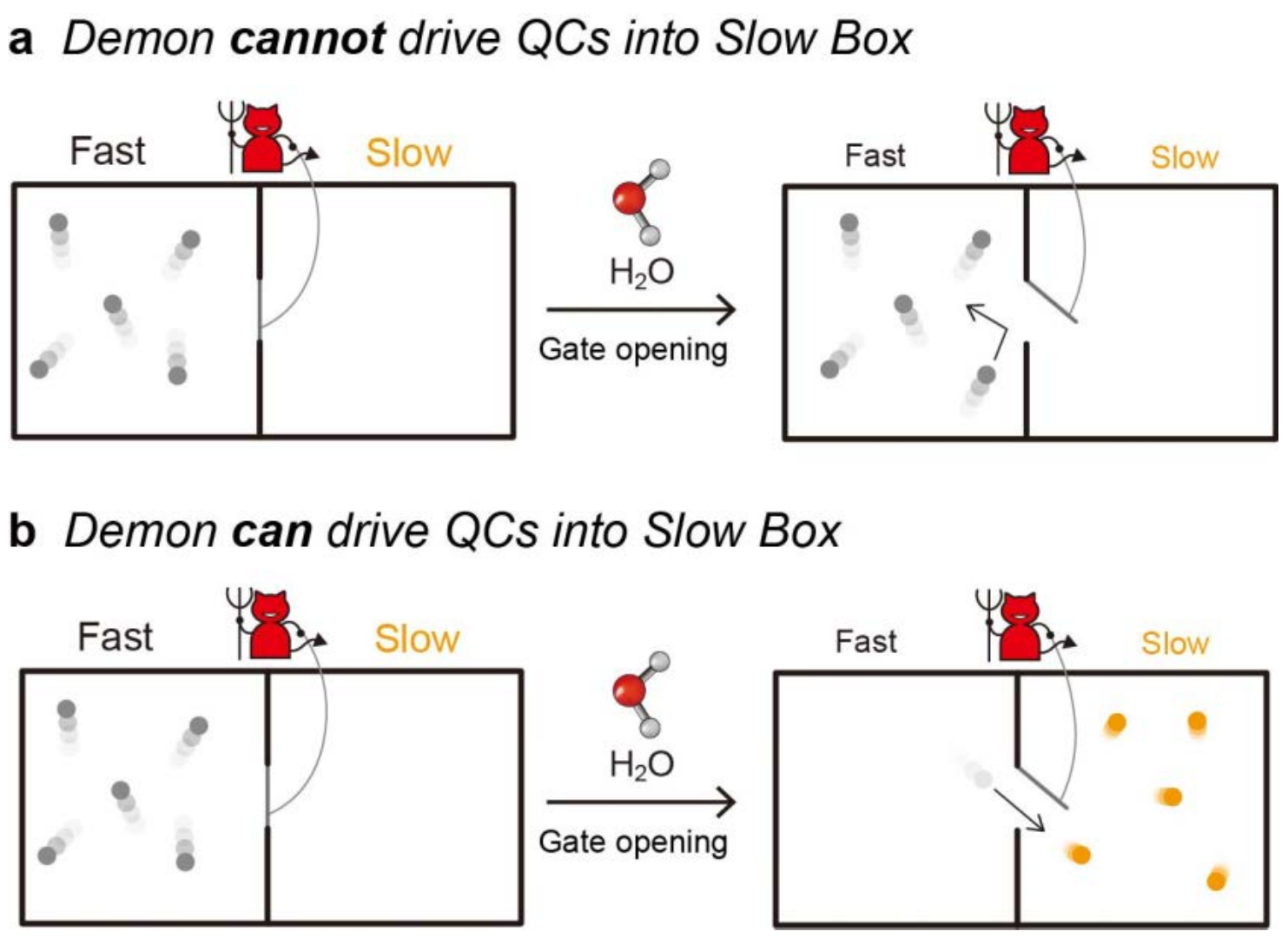

Fig. S5. Two types of behavior in this Maxwell's demon system: **a**, demon cannot drive QCs to Slow Box; **b**, demon can drive QCs to Slow Box.

Solutions of NBD/QC pairs were prepared in anhydrous MeCN (0.1 mM). All samples were irradiated until they reached PSS under UV light (λ = 315 nm, minimum 53 mW Mounted LED, 700 mA, Thorlabs LED), and then they followed the thermal back-conversion kinetics method (see in **Supplementary Section 2.4.4**). The NBD solutions were subsequently mixed with water, where the NBD-to-water molar ratio is 1:20, and then recorded the thermal back-conversion kinetics. The $t_{1/2}$, E_a, $\Delta H^‡$ and $\Delta S^‡$ values were calculated by **Equation S1-4**.

2.6 NMR spectra

2.6.1 NMR of NBD-CN,Br

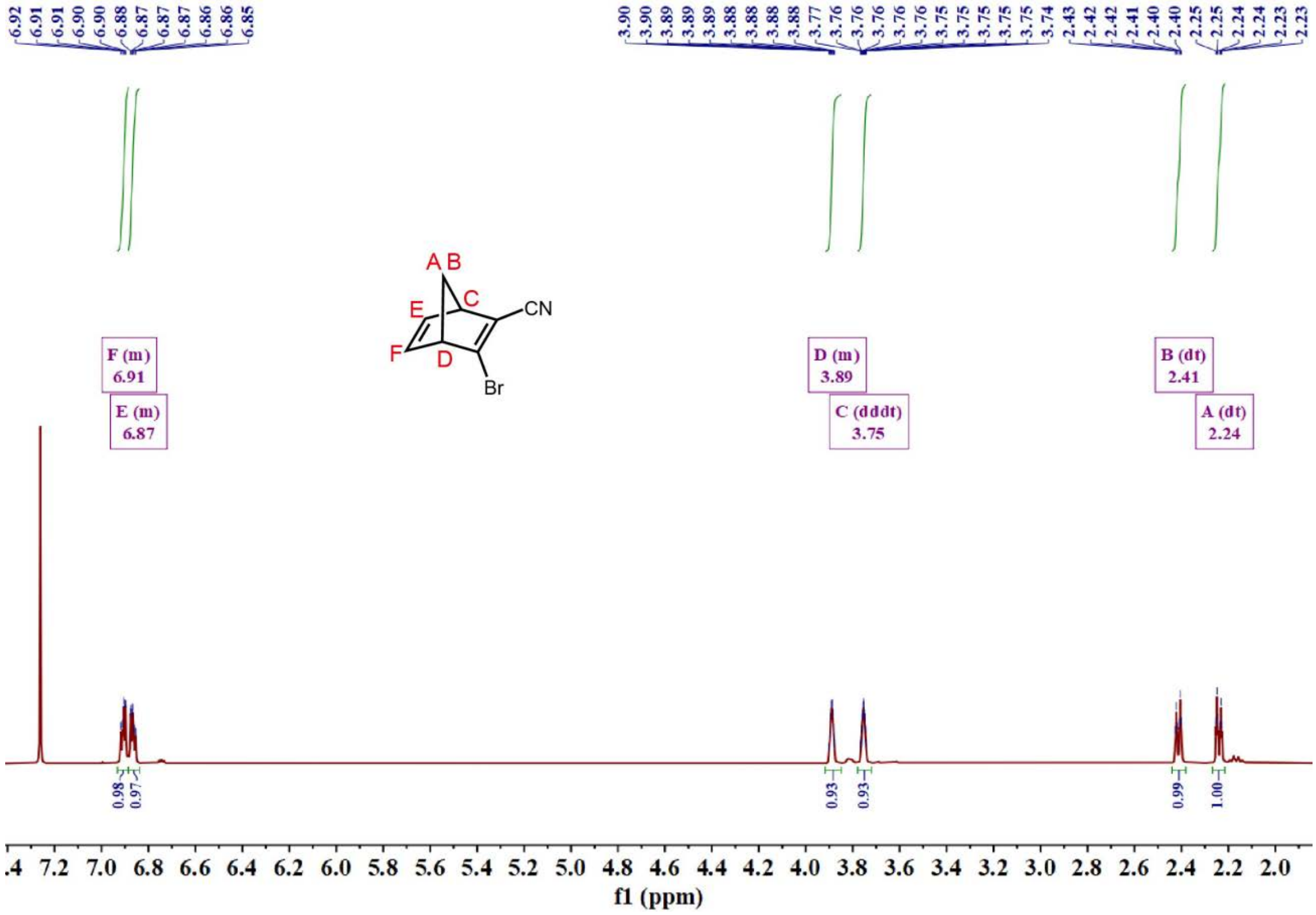

Fig. S6. ^{1}**H NMR (400 MHz) of NBD-CN,Br in $CDCl_3$ at 25 °C.**

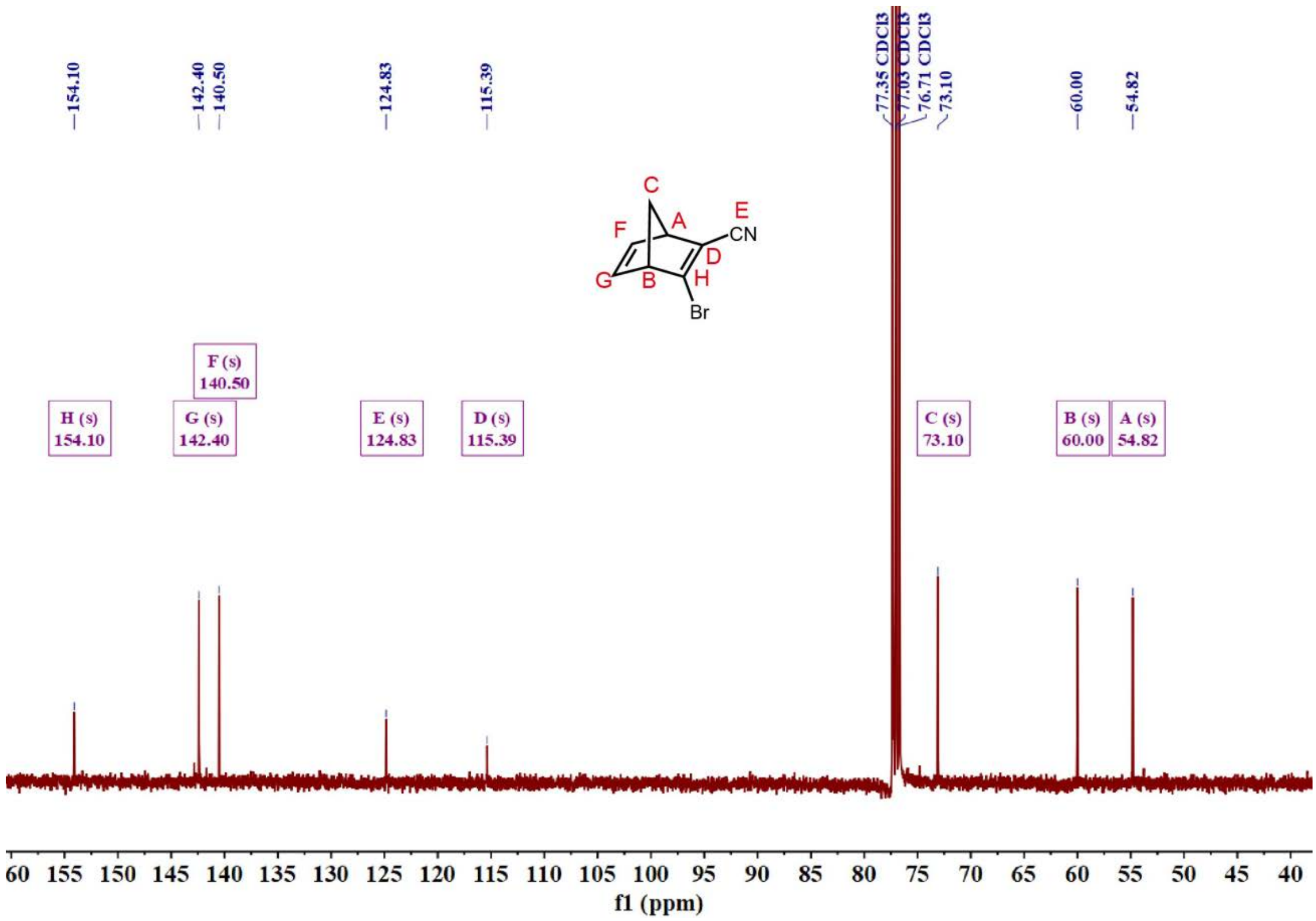

Fig. S7. ^{13}**C NMR (101 MHz) of NBD-CN,Br in $CDCl_3$ at 25 °C.**

2.6.2 NMR of NBD1

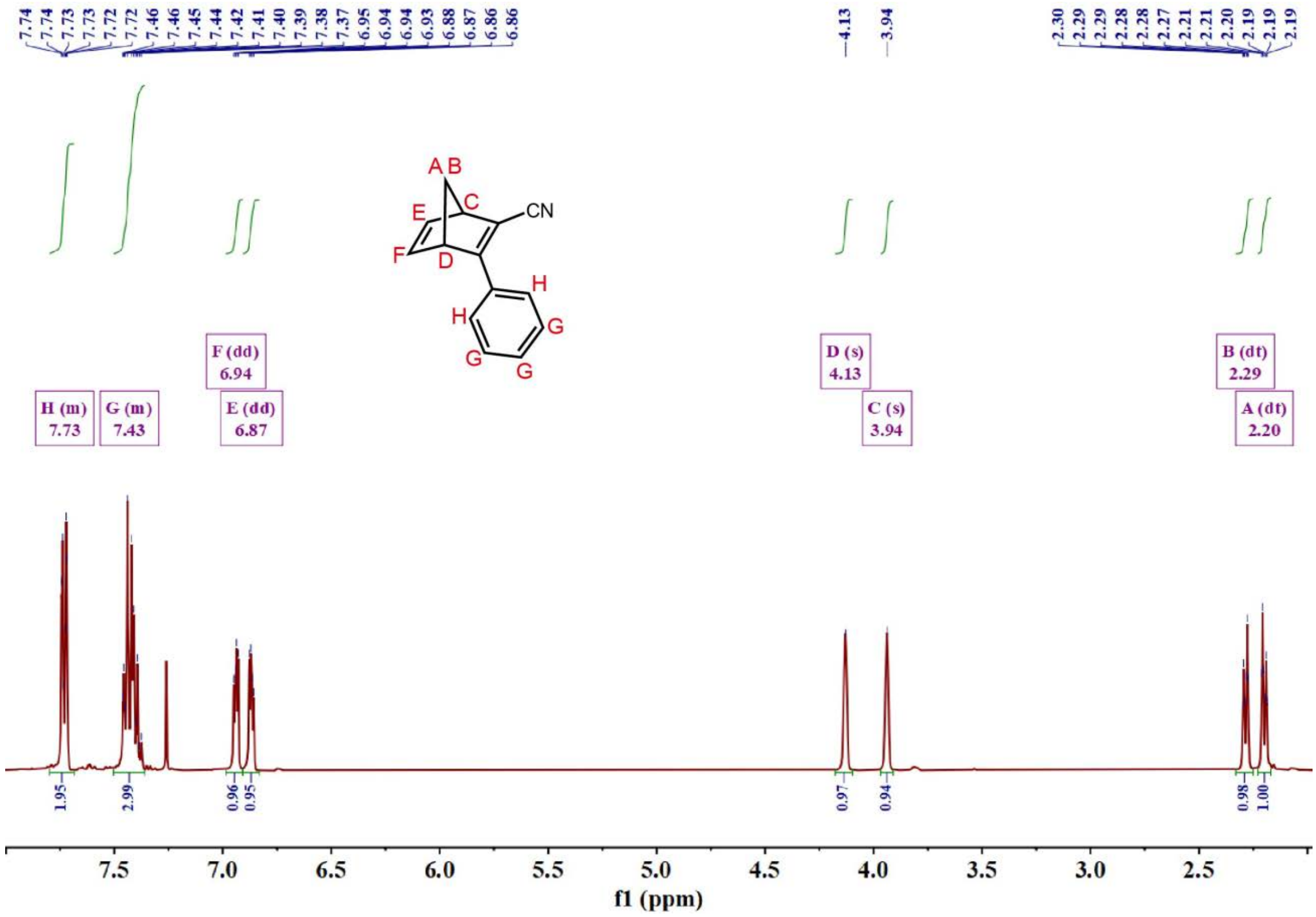

Fig. S8. **^{1}H NMR (400 MHz) of NBD1 in $CDCl_3$ at 25 °C.**

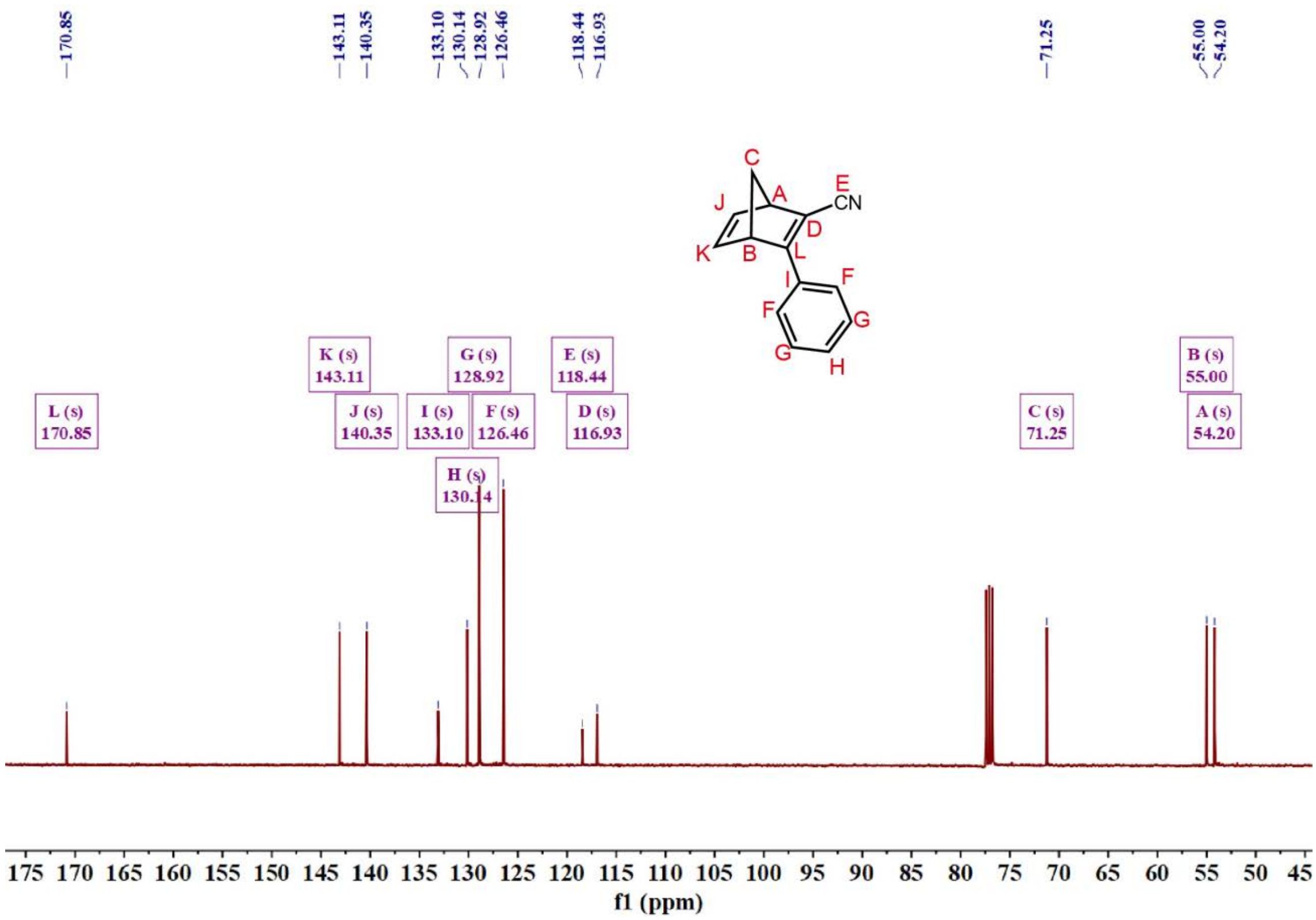

Fig. S9. **^{13}C NMR (101 MHz) of NBD1 in $CDCl_3$ at 25 °C.**

2.6.3 NMR of NBD2

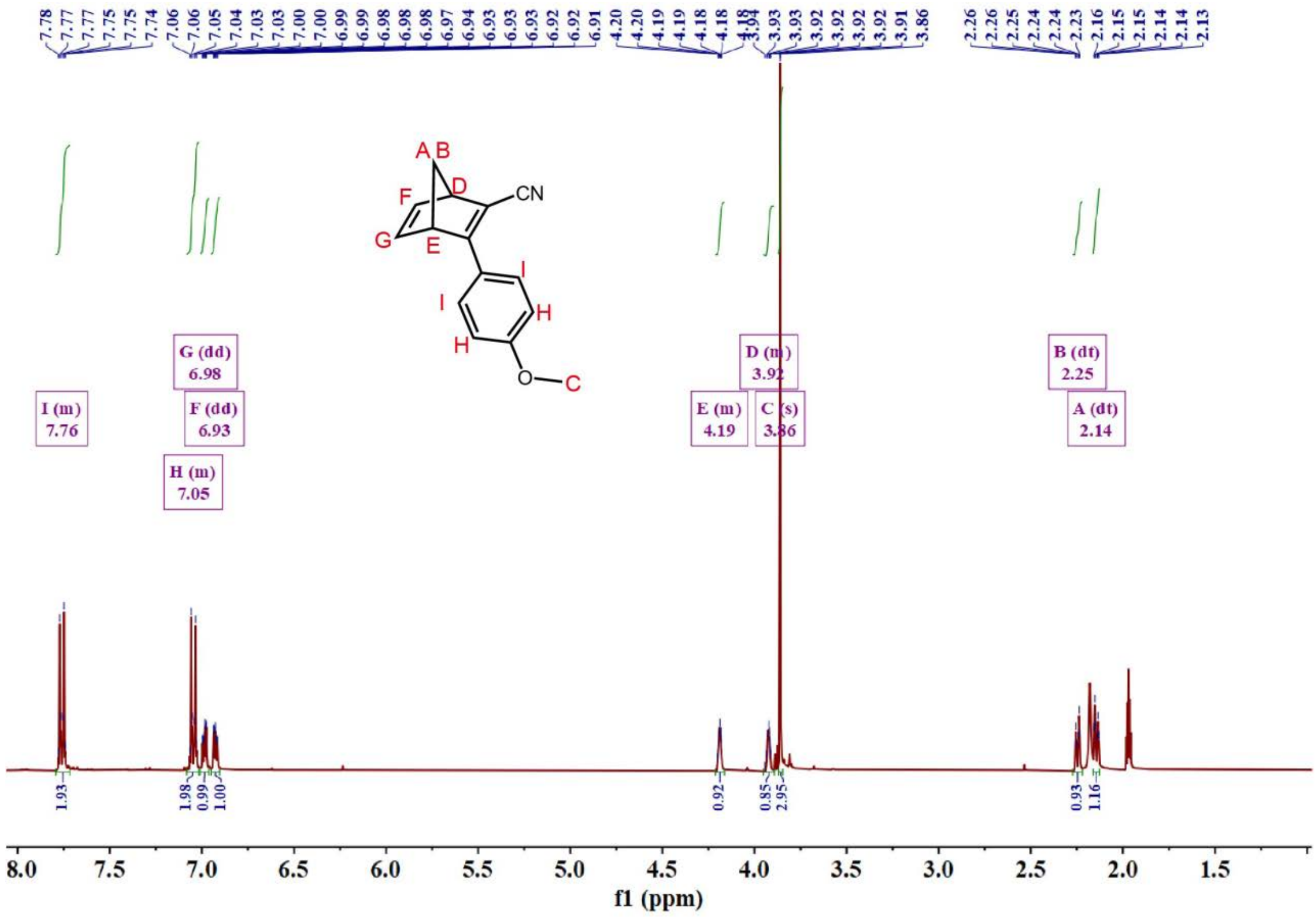

Fig. S10. **^{1}H NMR (400 MHz) of NBD2 in CD_3CN at 25 °C.**

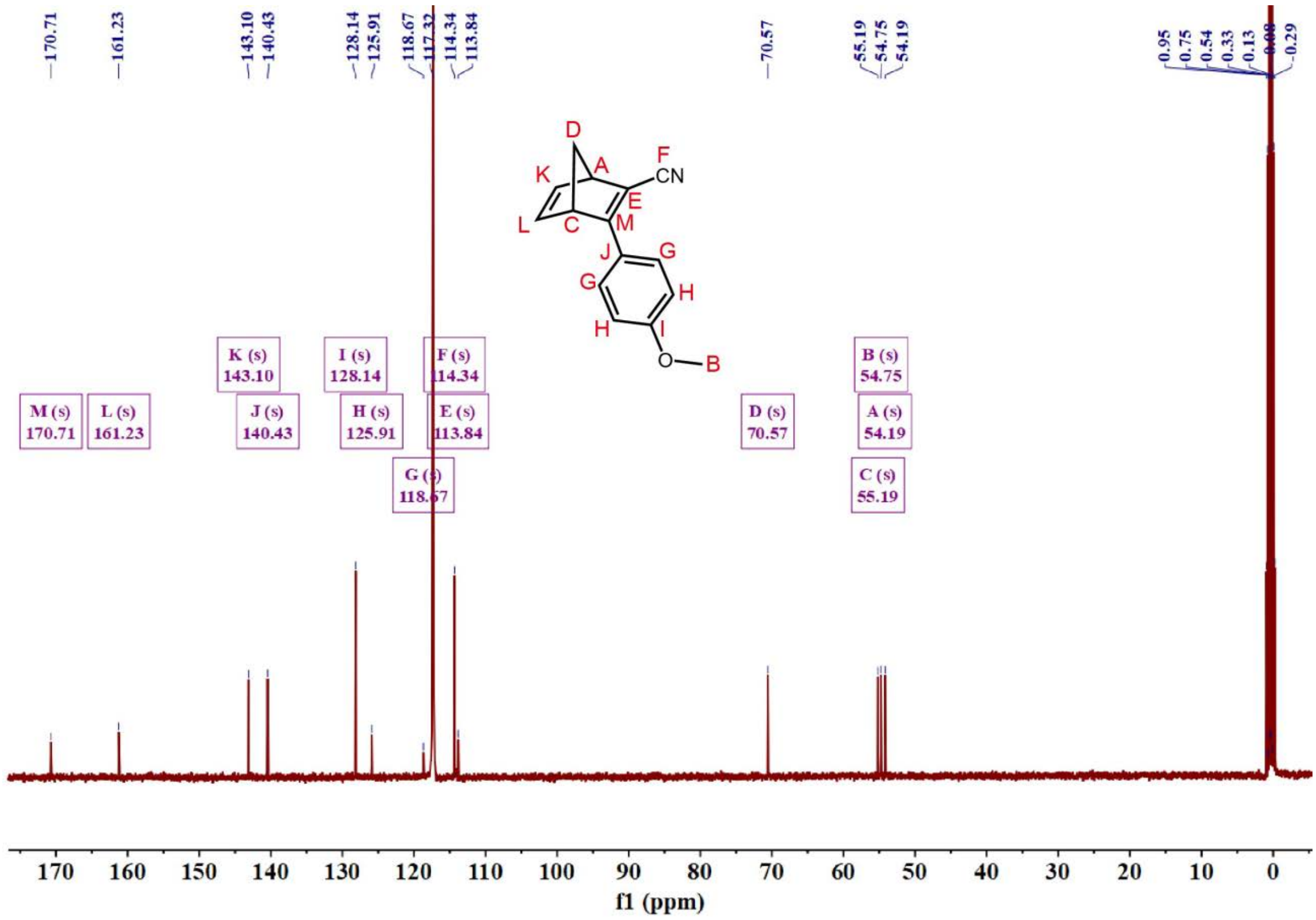

Fig. S11. **^{13}C NMR (101 MHz) of NBD2 in CD_3CN at 25 °C.**

2.6.4 NMR of NBD3

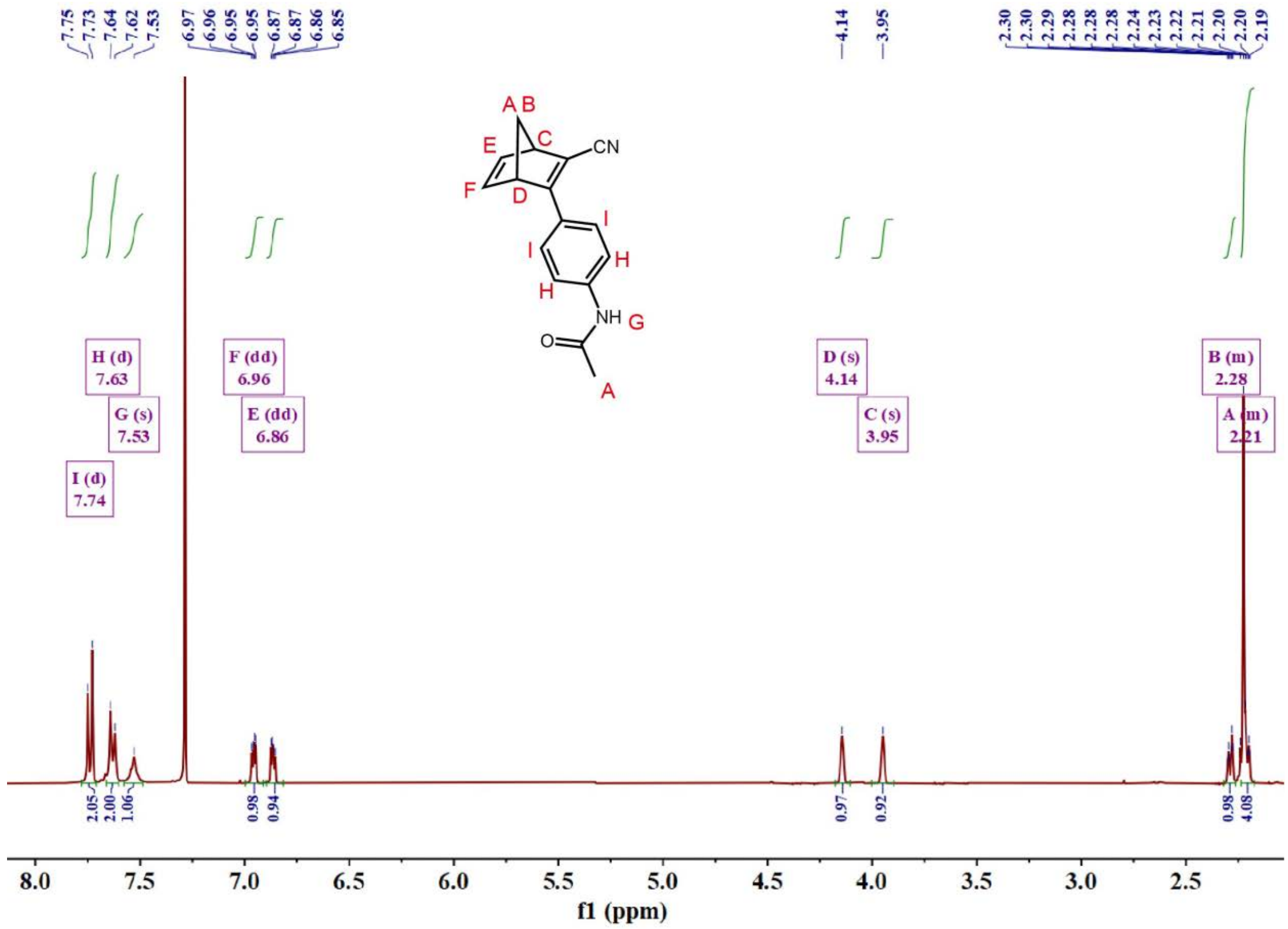

Fig. S12. ^{1}H NMR (400 MHz) of NBD3 in $CDCl_3$ at 25 °C.

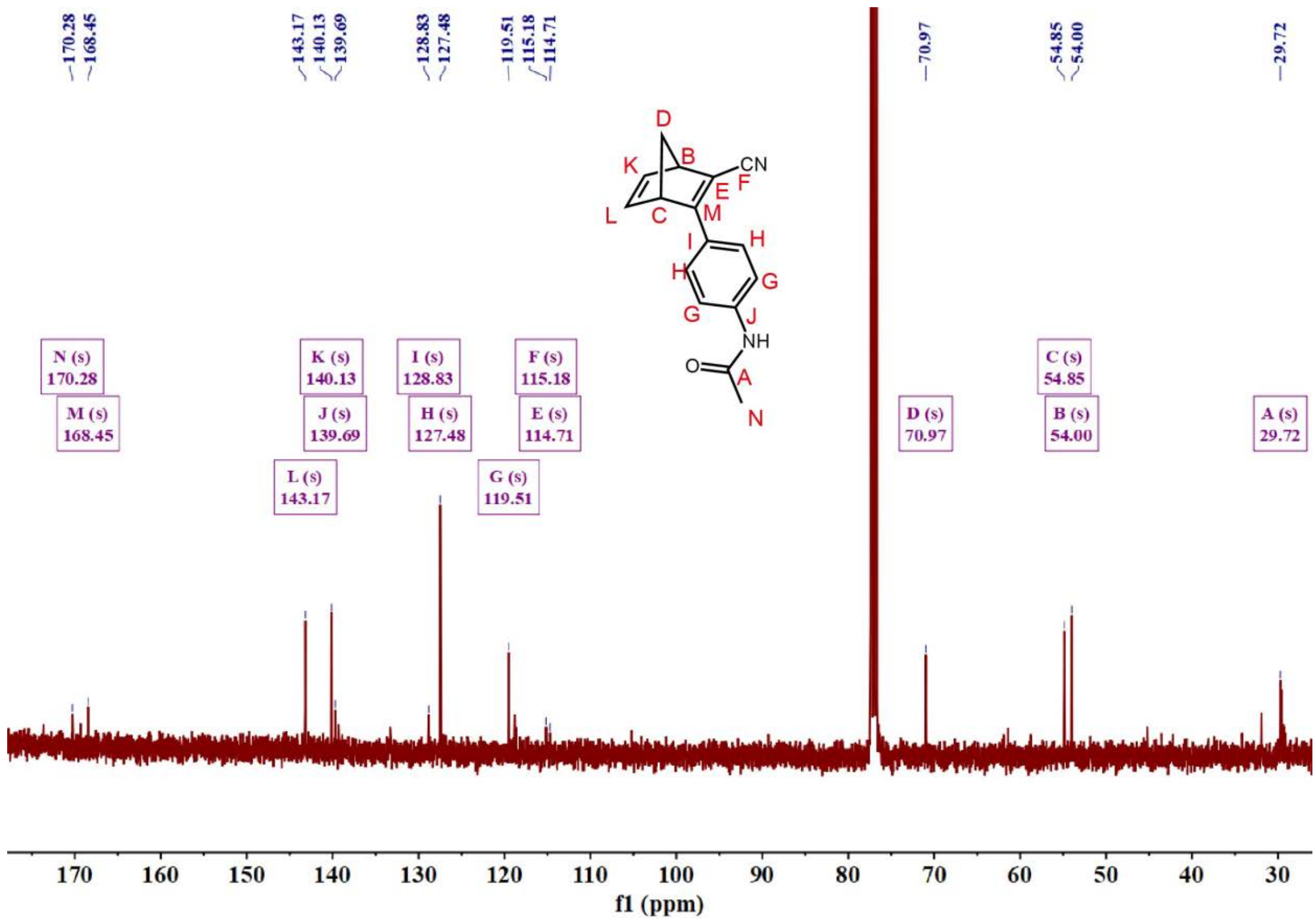

Fig. S13. ^{13}C NMR (101 MHz) of NBD3 in $CDCl_3$ at 25 °C.

2.6.5 NMR of ethyl 3-bromopropiolate

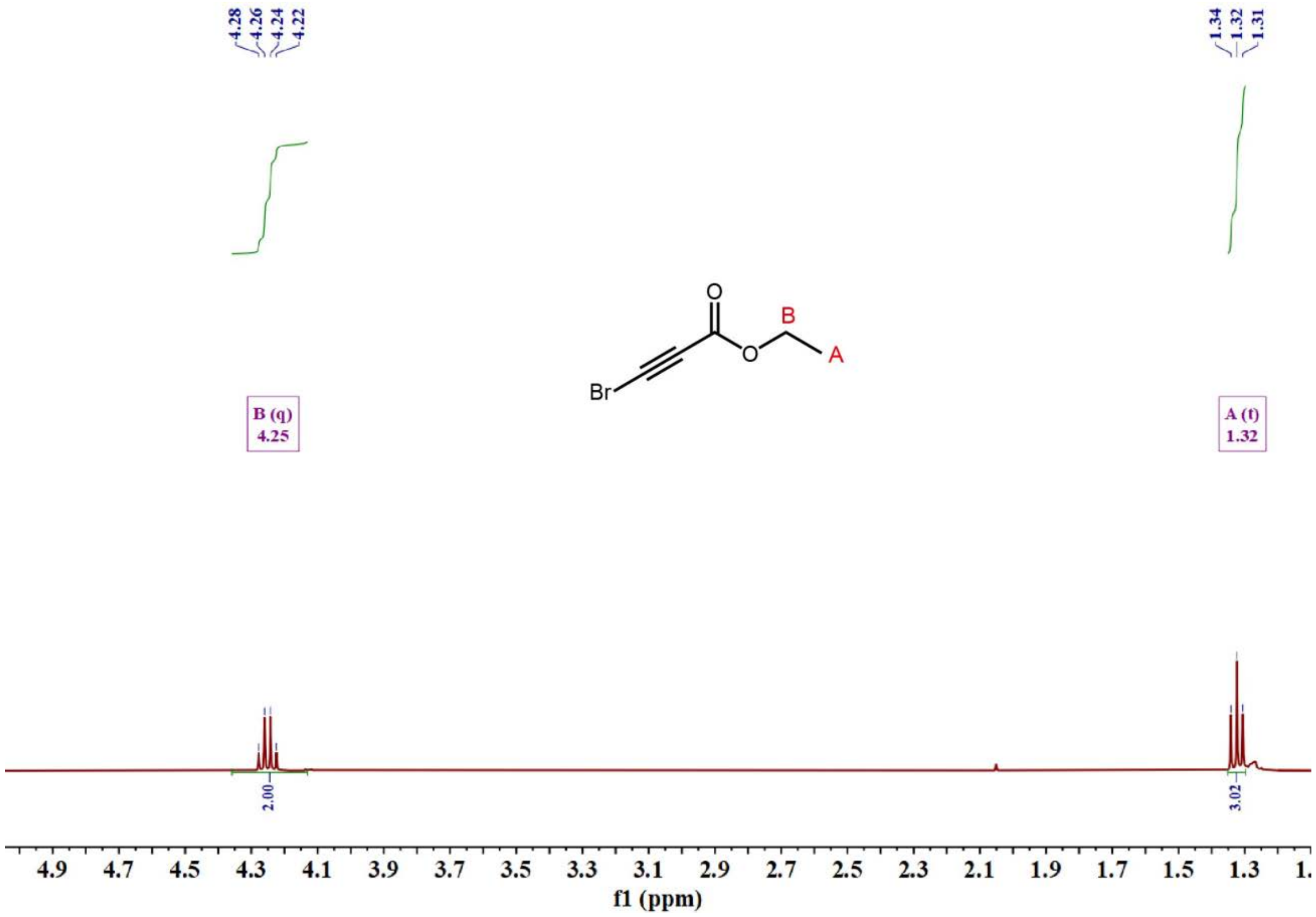

Fig. S14. **^{1}H NMR (400 MHz) of ethyl 3-bromopropiolate in $CDCl_3$ at 25 °C.**

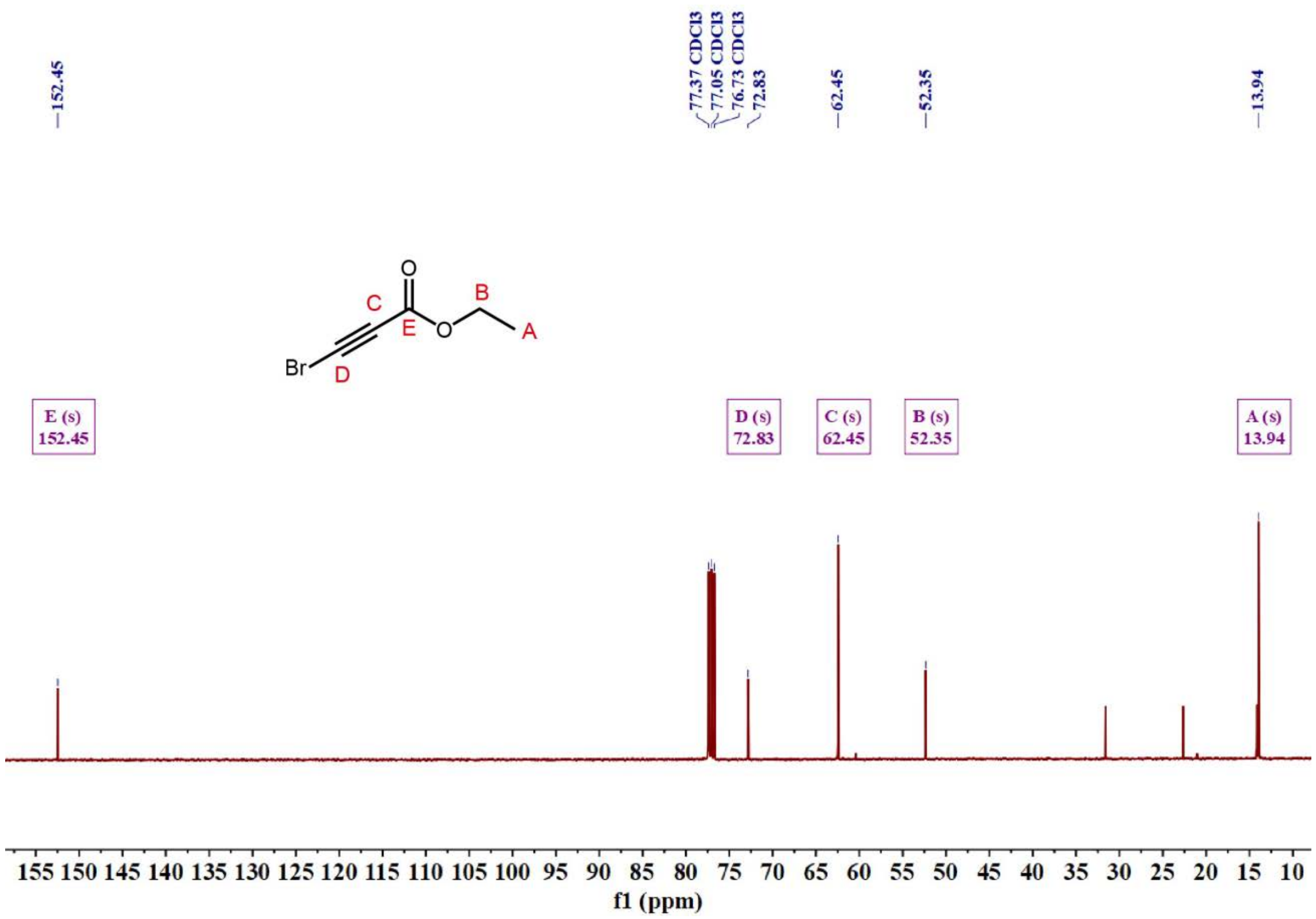

Fig. S15. **^{13}C NMR (101 MHz) of ethyl 3-bromopropiolate in $CDCl_3$ at 25 °C.**

2.6.6 NMR of NBD-Ester,Br

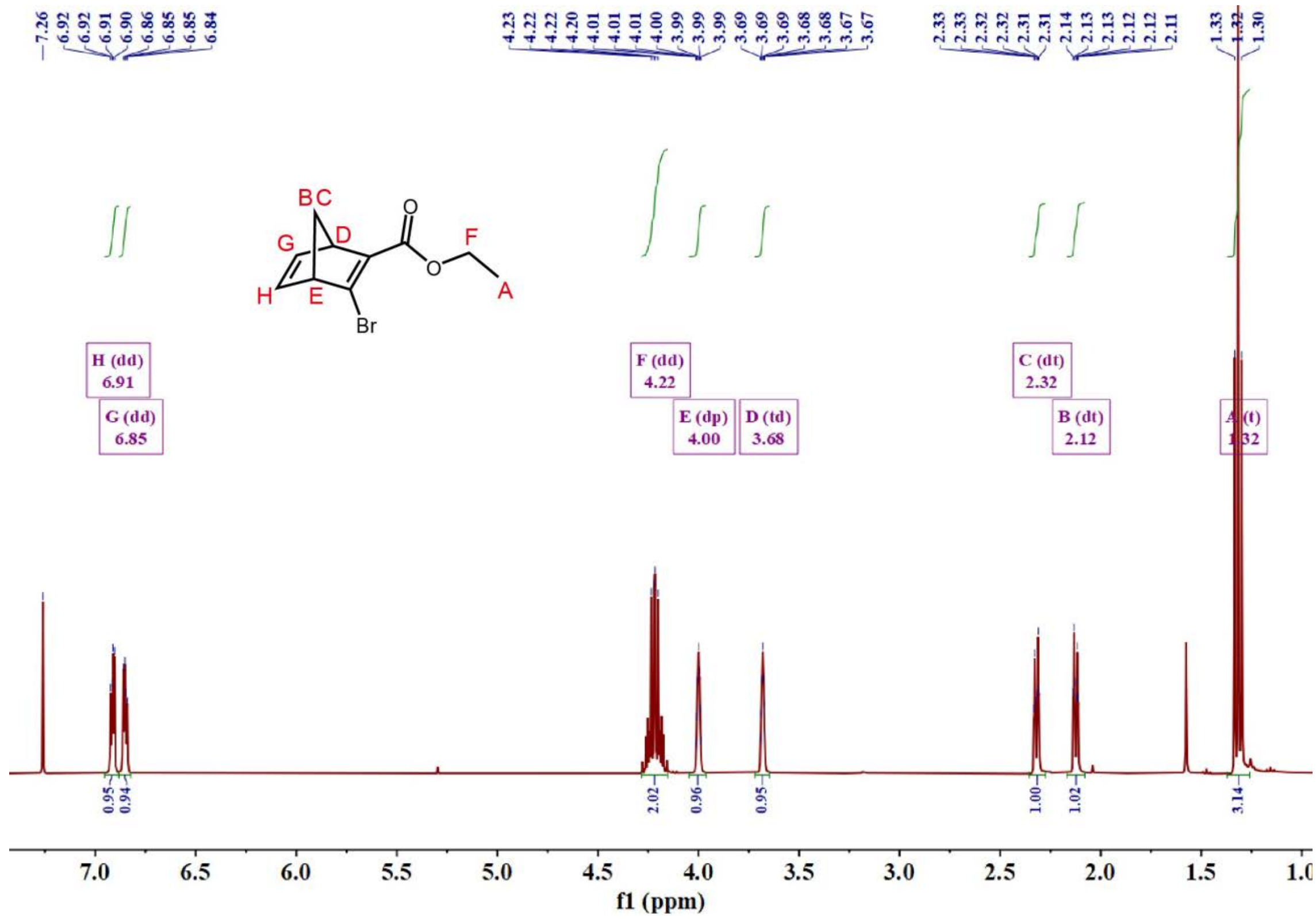

Fig. S16. **^{1}H NMR (400 MHz) of NBD-Ester,Br in $CDCl_3$ at 25 °C.**

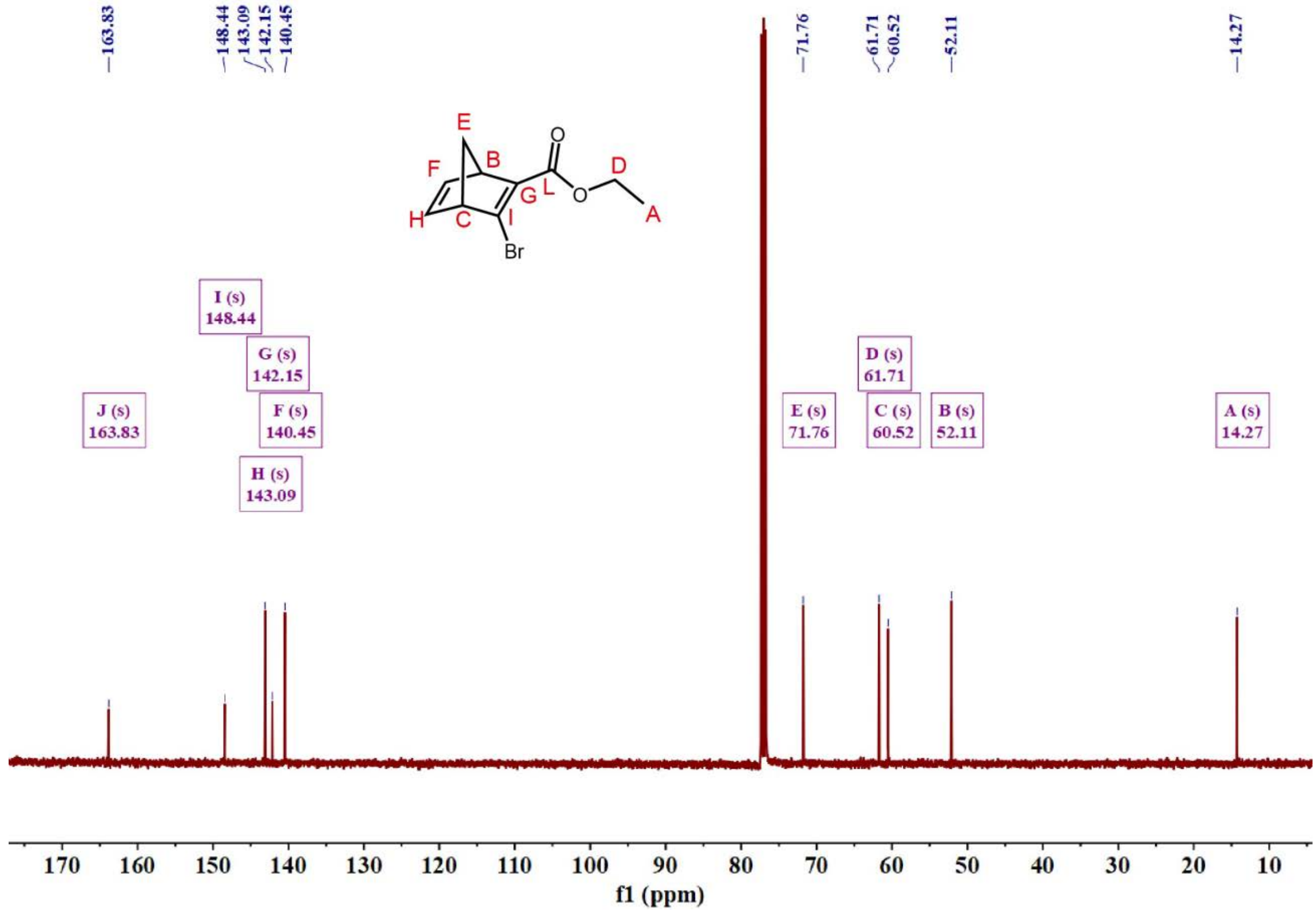

Fig. S17. **^{13}C NMR (101 MHz) of NBD-Ester,Br in $CDCl_3$ at 25 °C.**

2.6.7 NMR of NBD4

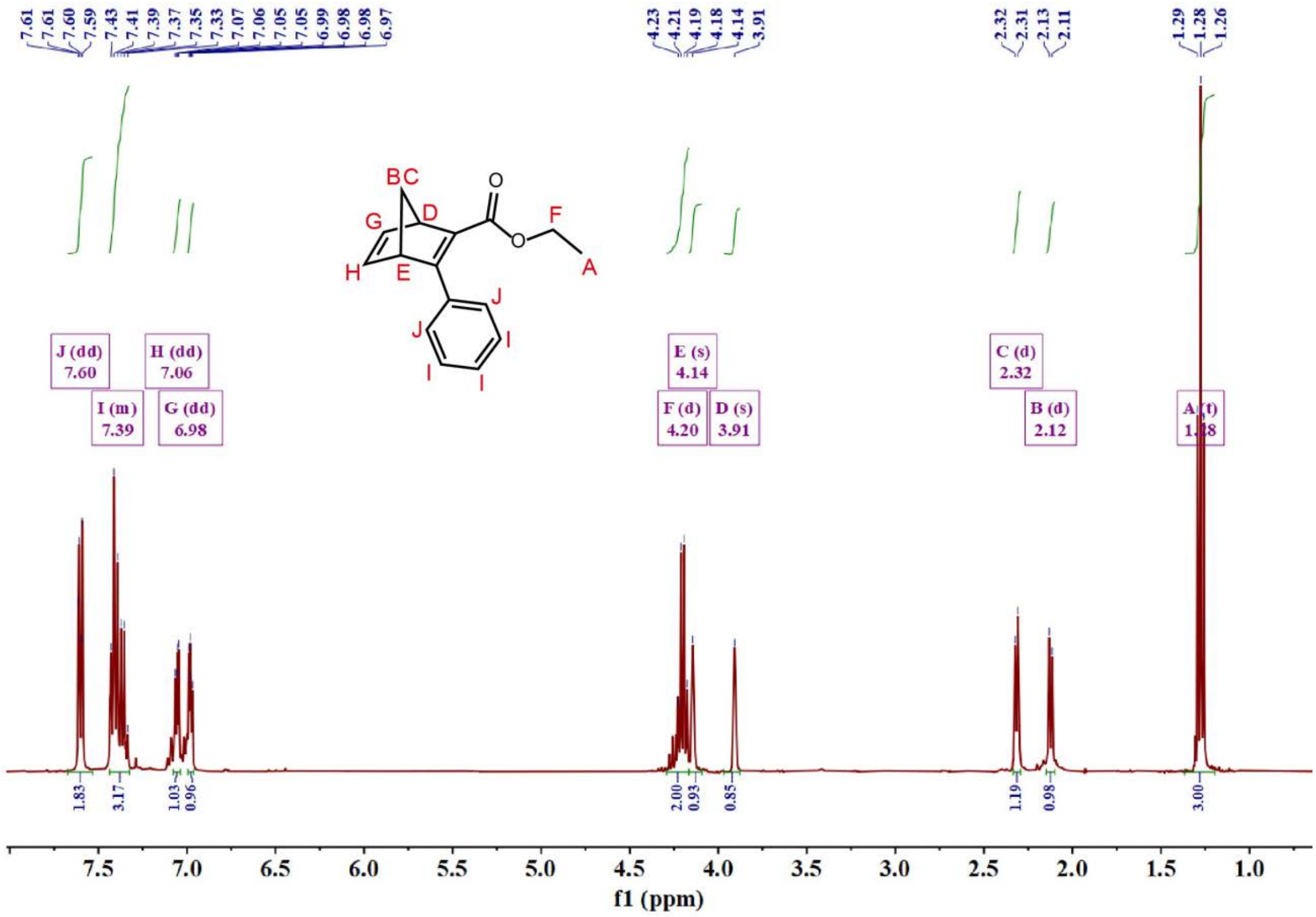

Fig. S18. **^{1}H NMR (400 MHz) of NBD4 in $CDCl_3$ at 25 °C.**

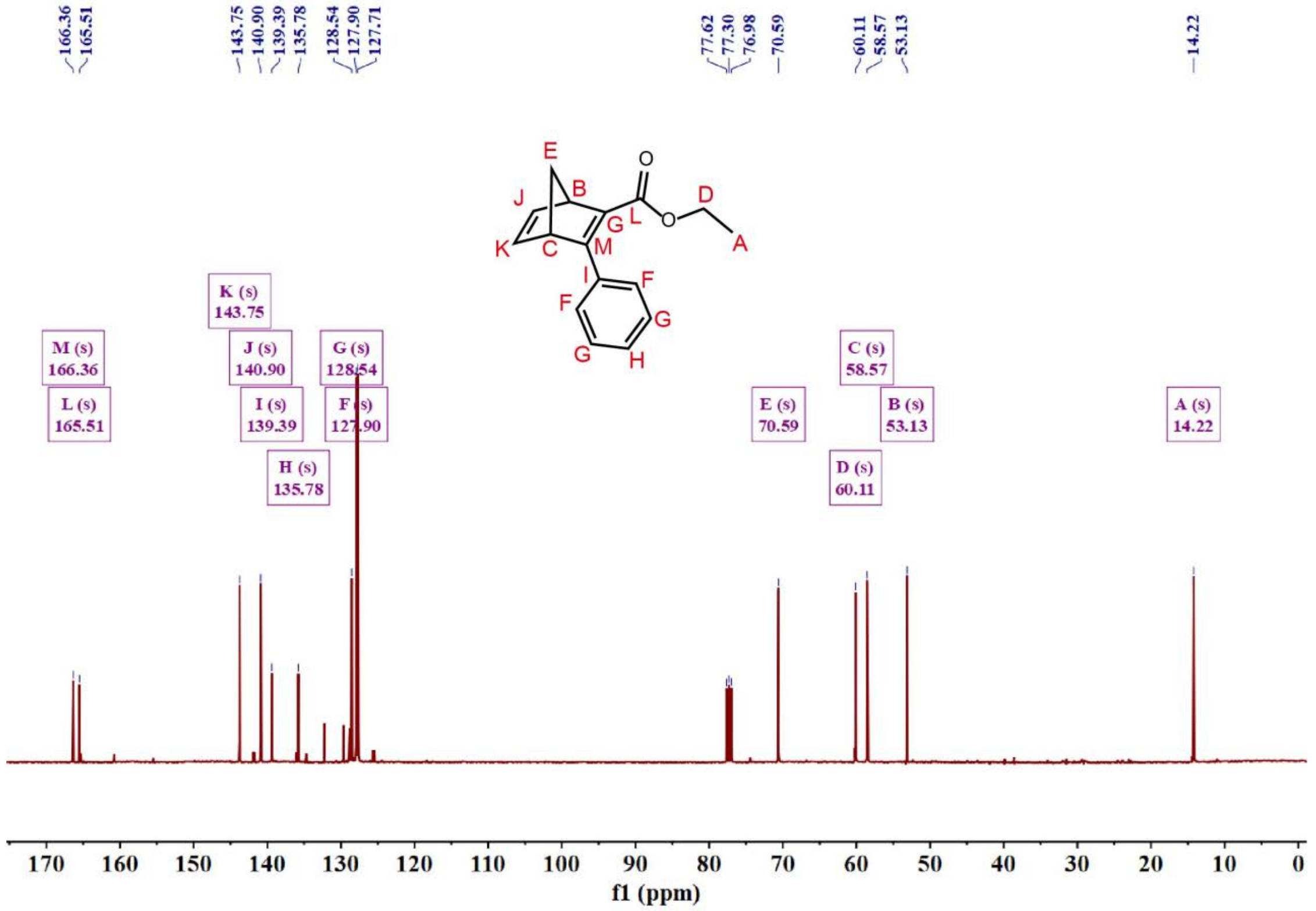

Fig. S19. **^{13}C NMR (101 MHz) of NBD4 in $CDCl_3$ at 25 °C.**

2.6.8 NMR of NBD5

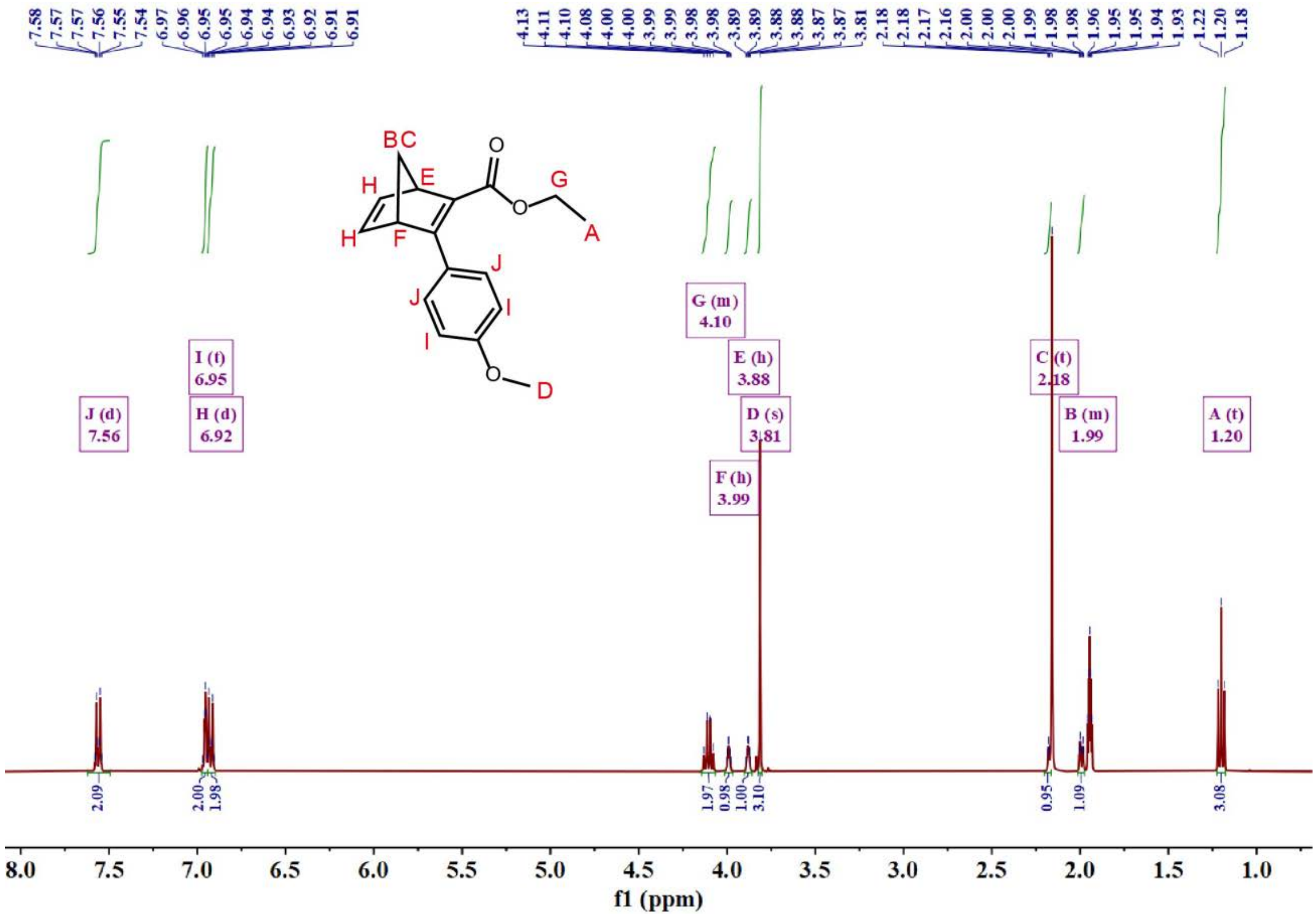

Fig. S20. **^{1}H NMR (400 MHz) of NBD5 in CD_3CN at 25 °C.**

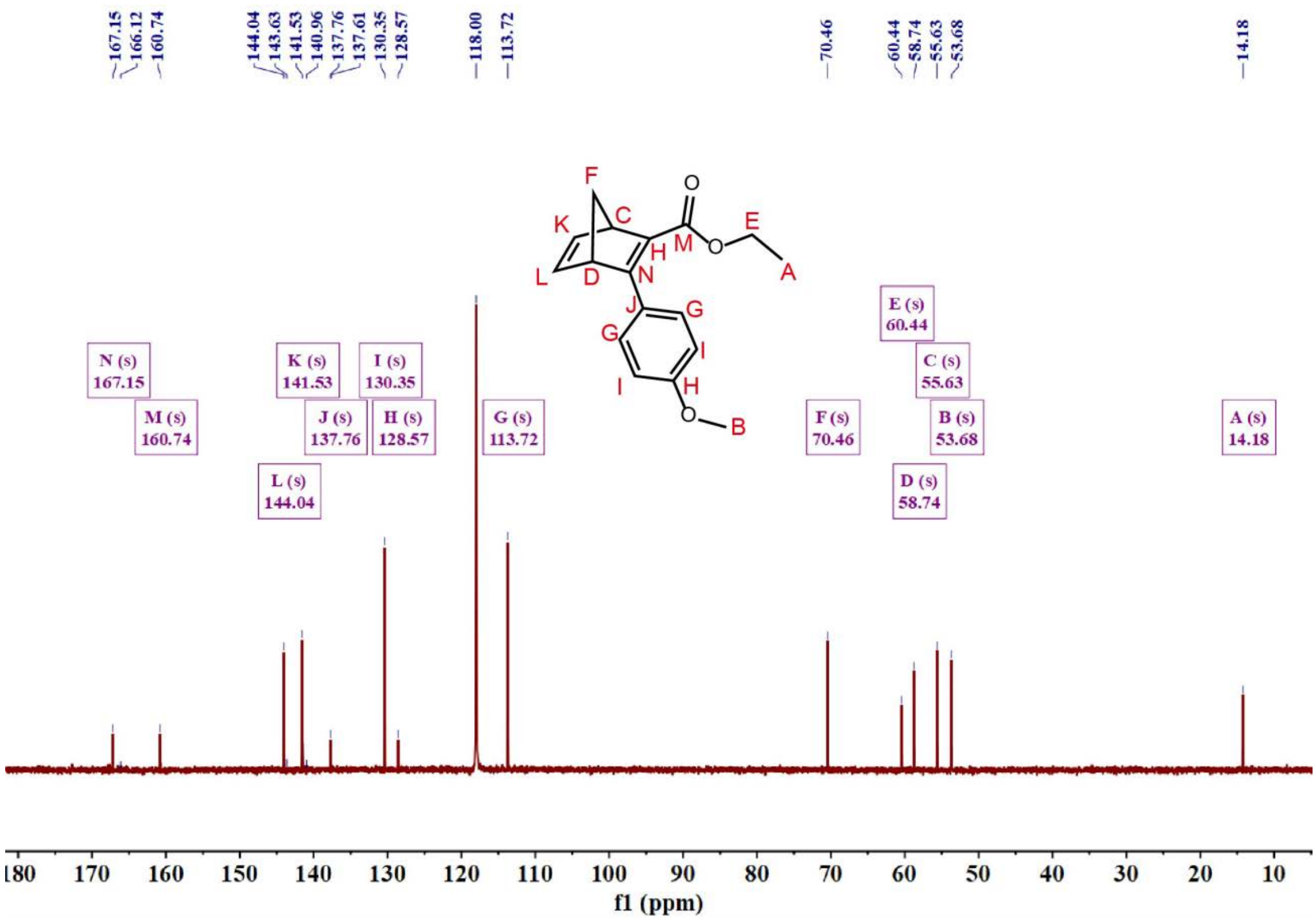

Fig. S21. **^{13}C NMR (101 MHz) of NBD5 in CD_3CN at 25 °C.**

2.6.9 NMR of NBD6

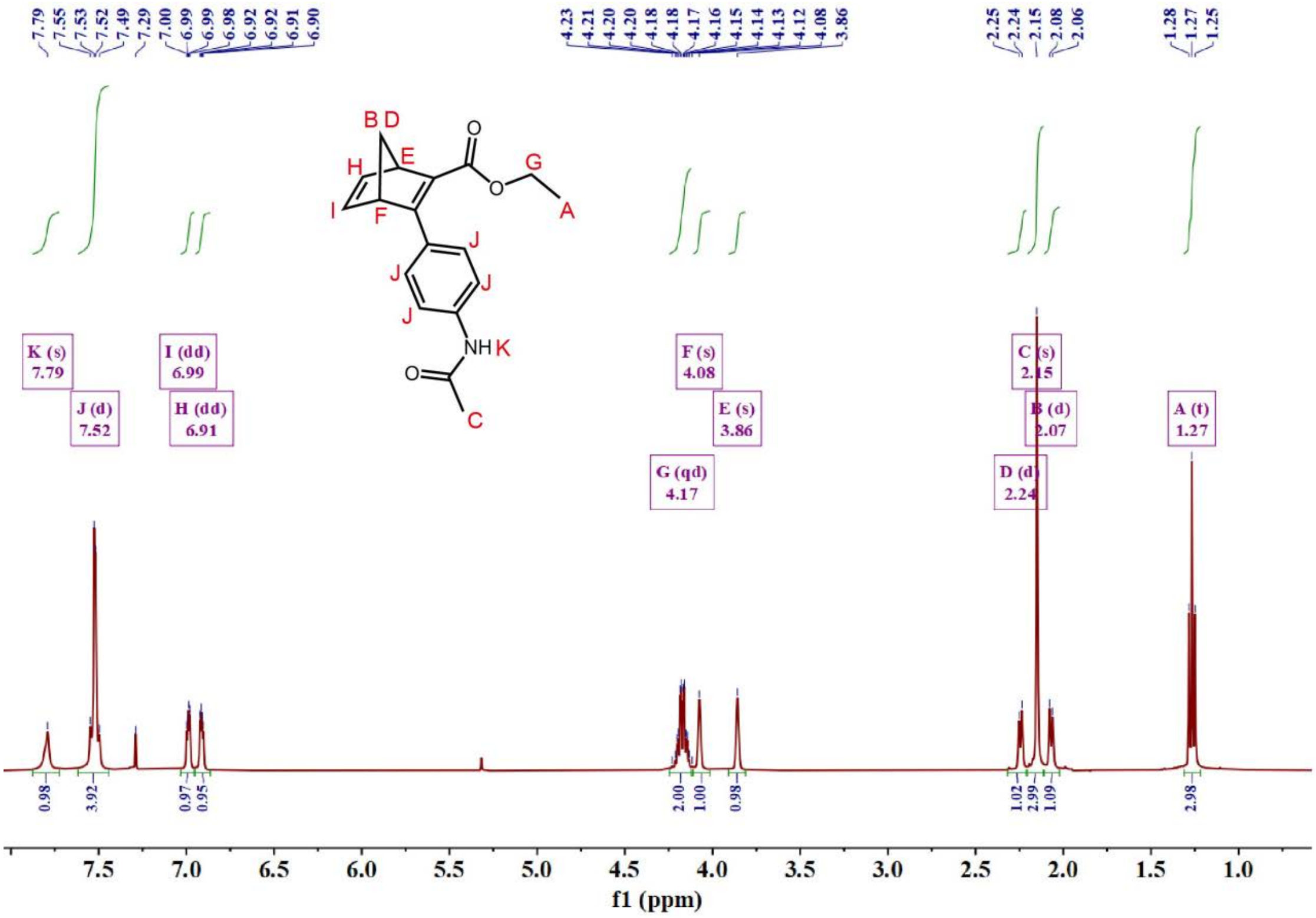

Fig. S22. **^{1}H NMR (400 MHz) of NBD6 in $CDCl_3$ at 25 °C.**

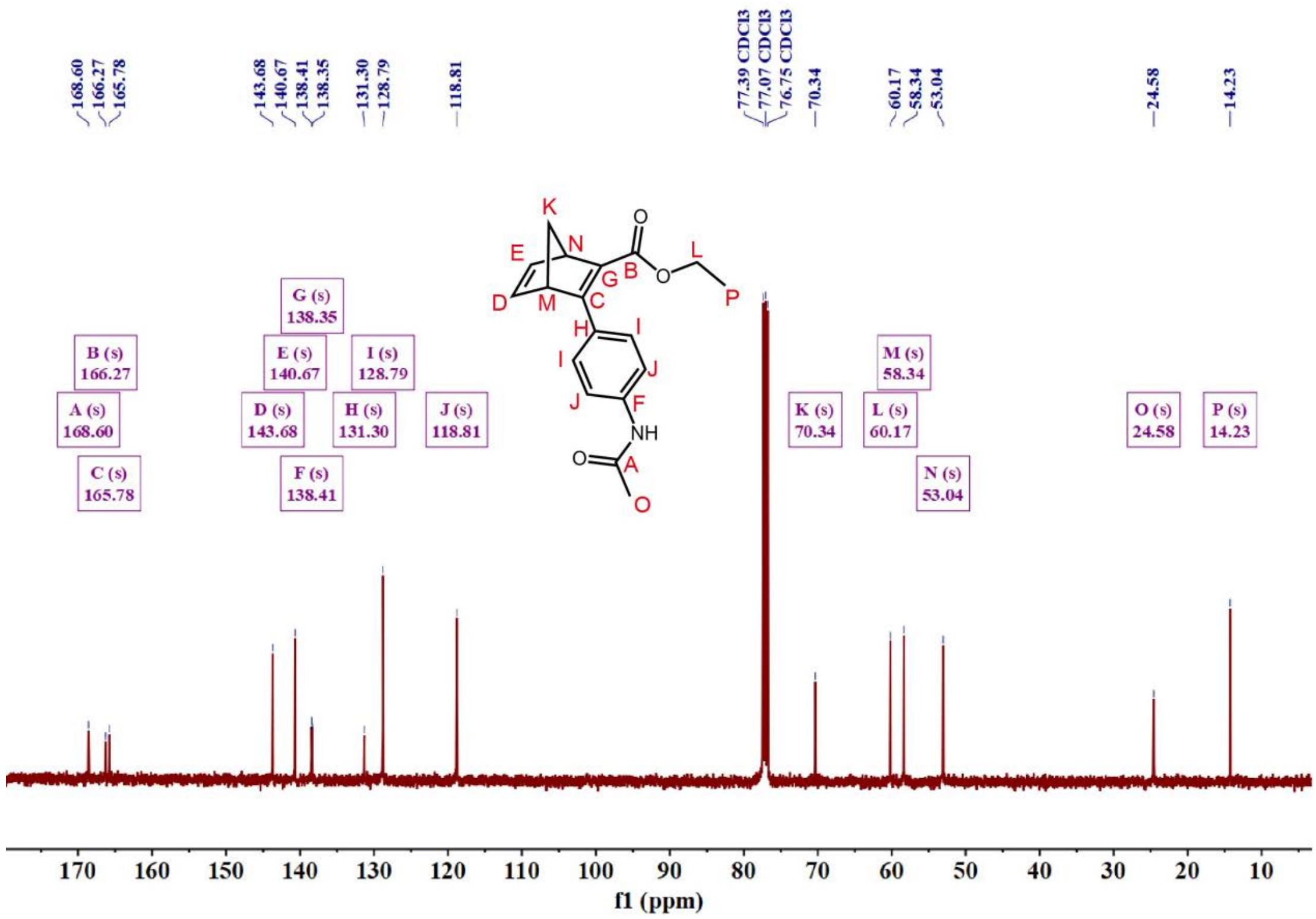

Fig. S23. **^{13}C NMR (101 MHz) of NBD6 in $CDCl_3$ at 25 °C.**

3. Results and Discussion

3.1 Molecular design (Suppl. Note 8)

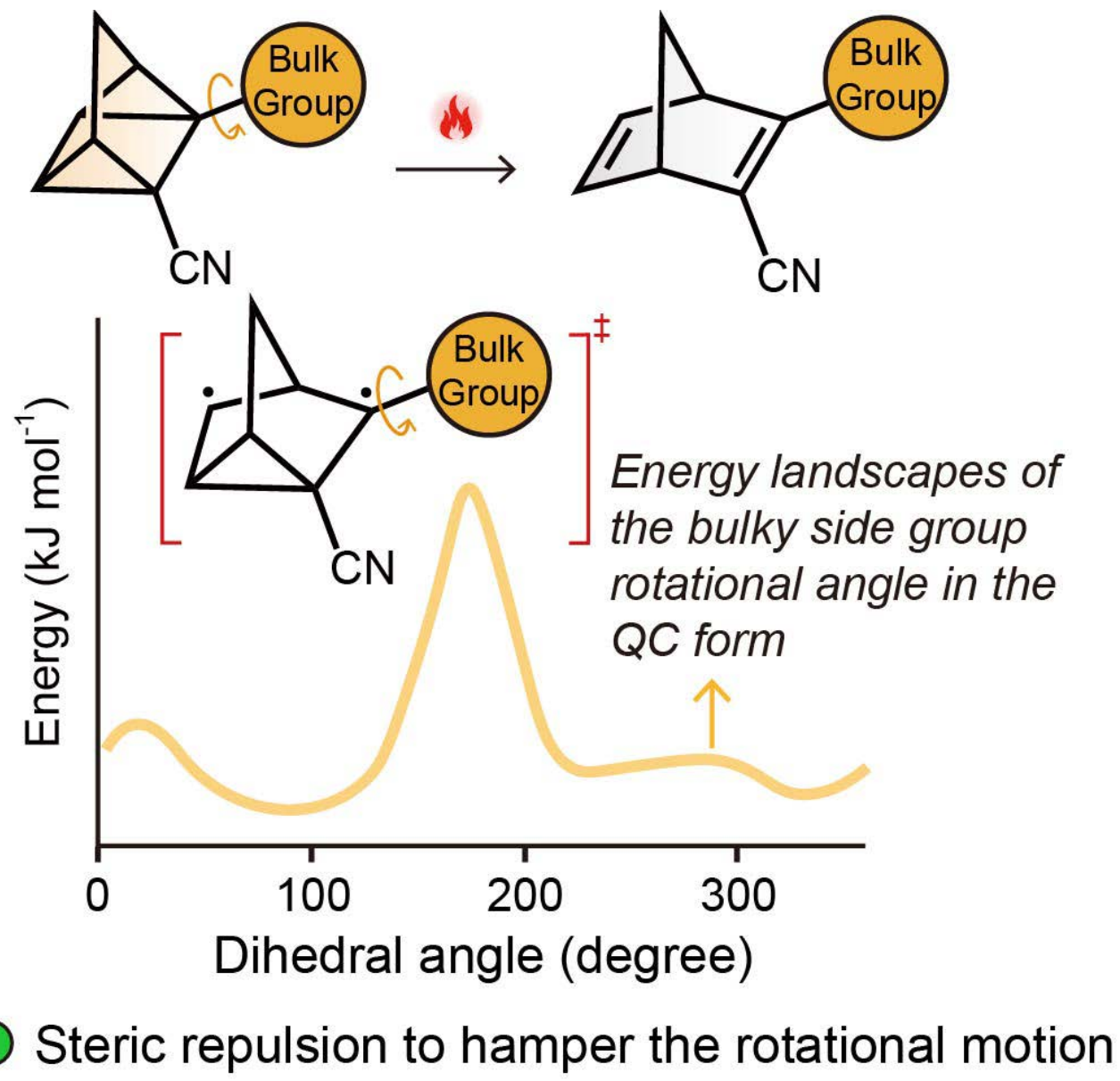

Fig. S24. Bulk groups are designed for higher backconversion entropy and long $t_{1/2}$.[24]

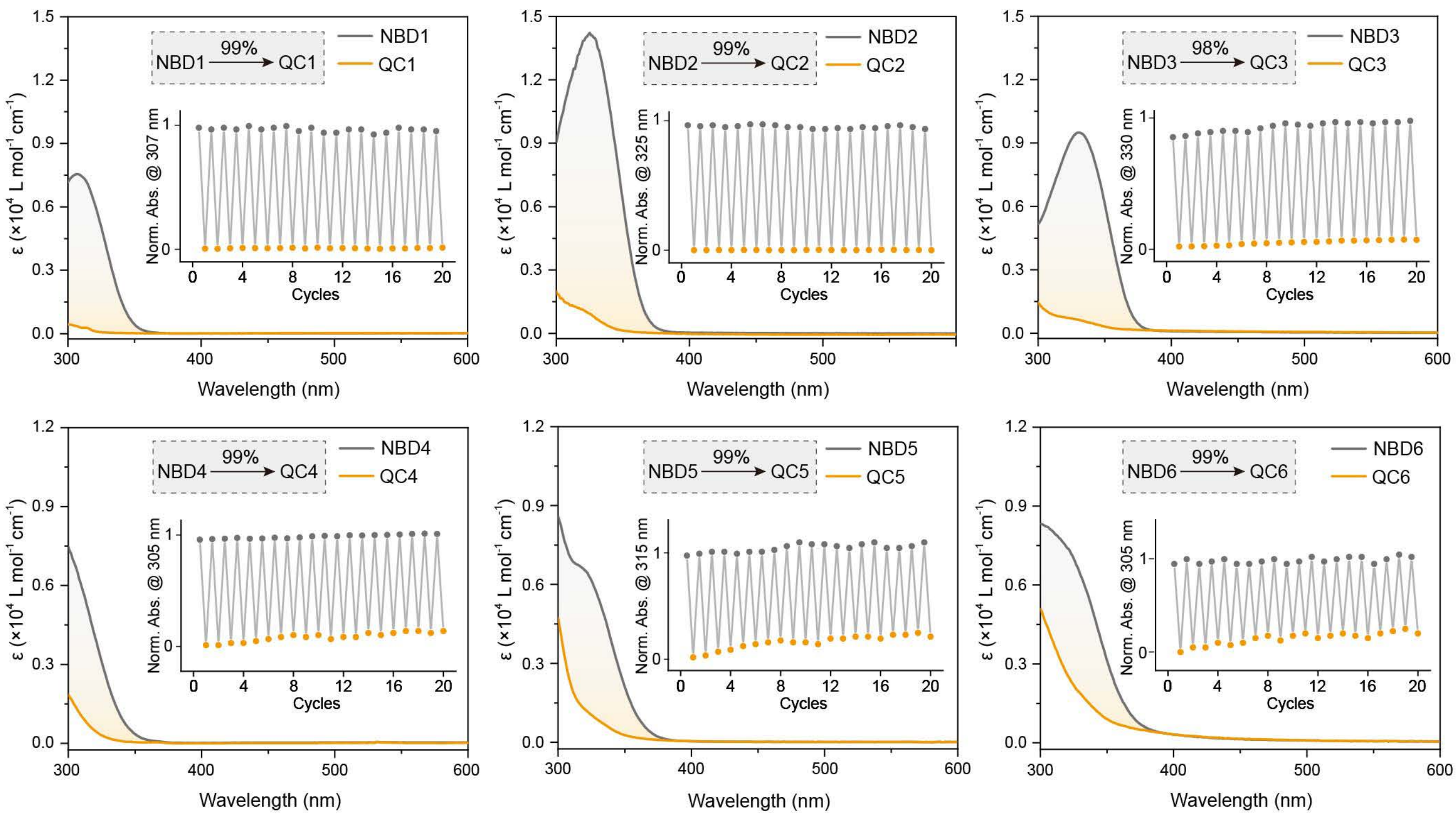

Fig. S25. Photochromism of NBDs. UV/Vis absorption spectra with molar extinction coefficients of **NBD1-6** in toluene. Insets show the photoconversion yields at photostation state by 340 nm light irradiation, and the stability of NBDs, where NBD to QC conversion by 315 nm light irradiation and subsequent QC to NBD by thermal backconversion at 80 °C, respectively.

The photoconversion yields of NBD derivatives under 340 nm irradiation were calculated in ^{1}H NMR spectra via integration of peaks representing NBD and QC isomers. After photoisomerization of NBD-to-QC, the protons presented clear shifts. For example, the proton NMR spectra of NBD6 showed characteristic peaks at 3.88 ppm when the molecule was in the norbornadiene (NBD) state. Upon photoisomerization to the corresponding quadricyclane (QC) state, these proton signals underwent a downfield shift, appearing at 3.96 ppm, indicating a change in the electronic environment of the protons associated with the structural transformation (Fig. S21). This clear shift in chemical shift confirms the successful conversion from the NBD to the QC state.

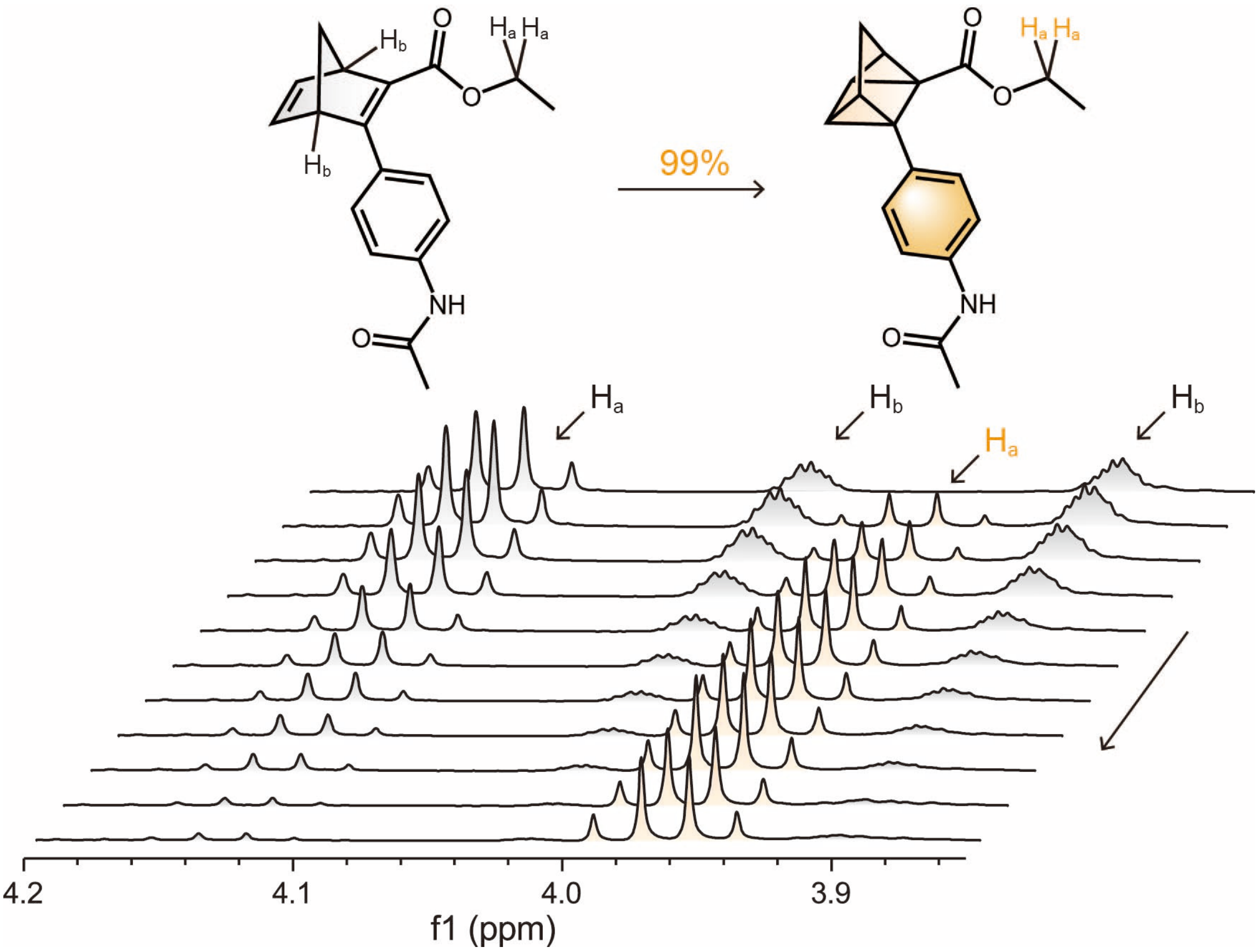

Fig. S26. ^{1}H NMR (400 MHz) spectra and photoconversion yield of NBD6 under 340 nm light irradiation (solvent: $CDCl_3$).

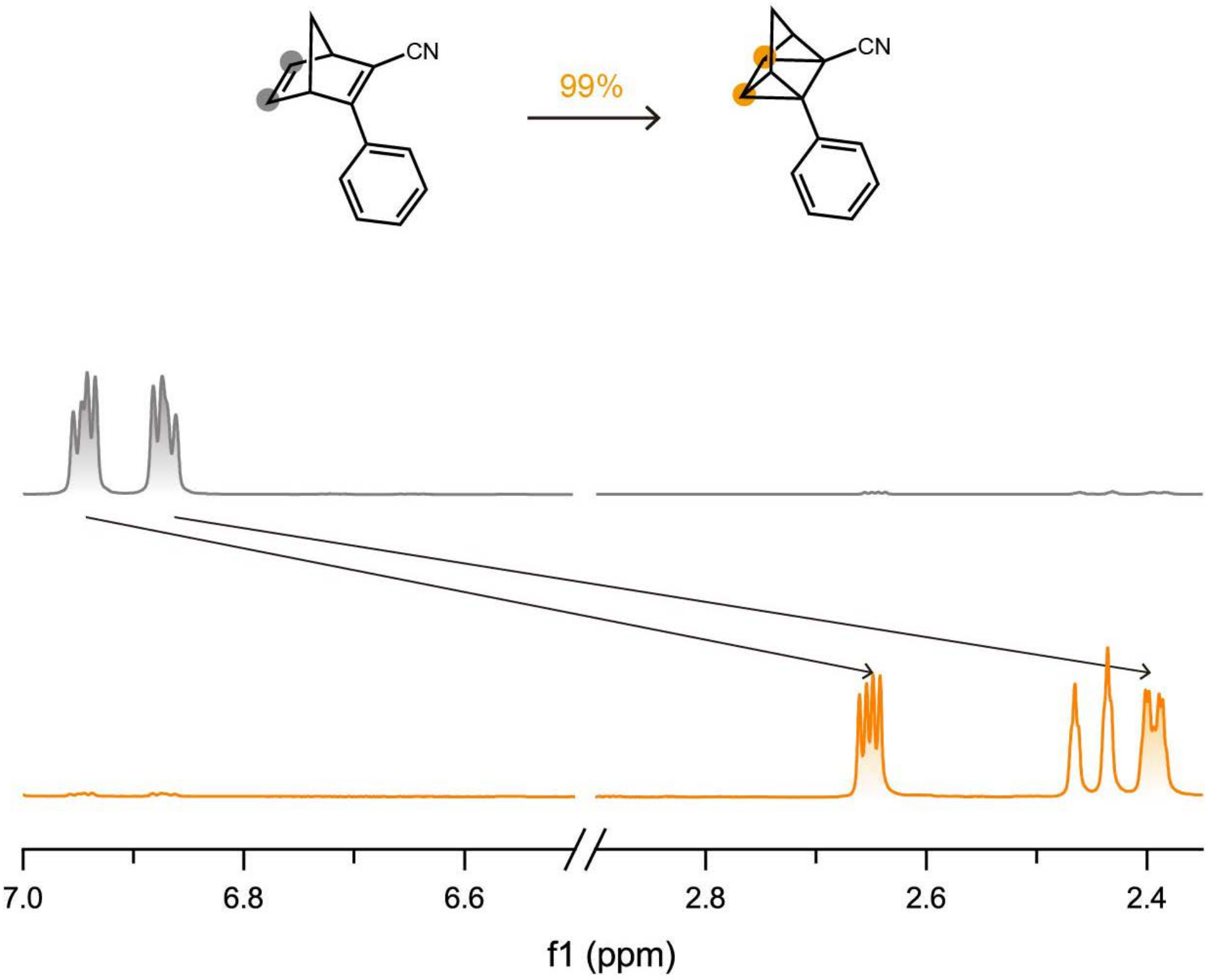

Fig. S27. ^{1}H NMR (400 MHz) spectra and photoconversion yield of NBD1 under 340 nm light irradiation (solvent: $CDCl_3$).

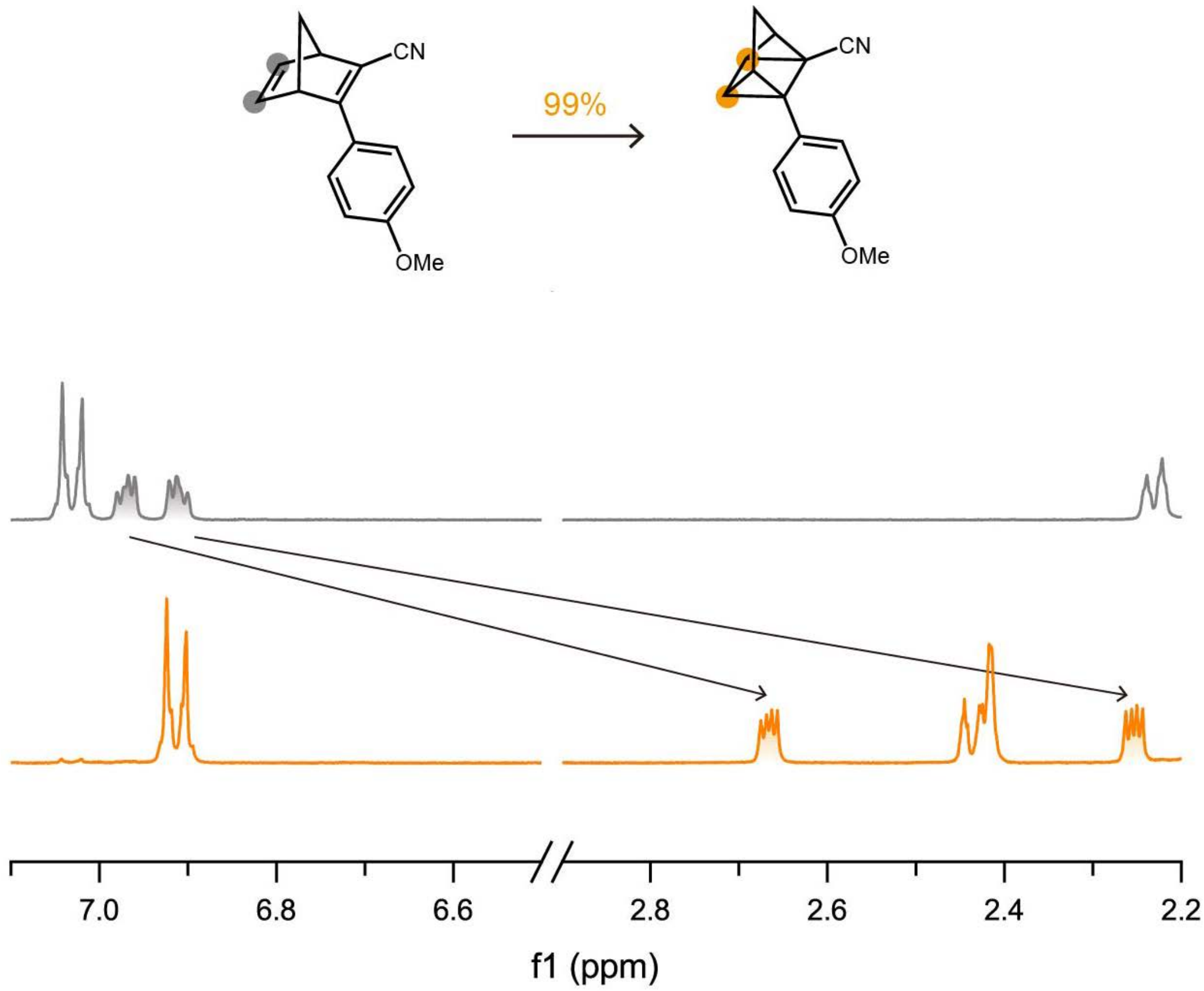

Fig. S28. ^{1}H NMR (400 MHz) spectra and photoconversion yield of NBD2 under 340 nm light irradiation (solvent: $CDCl_3$).

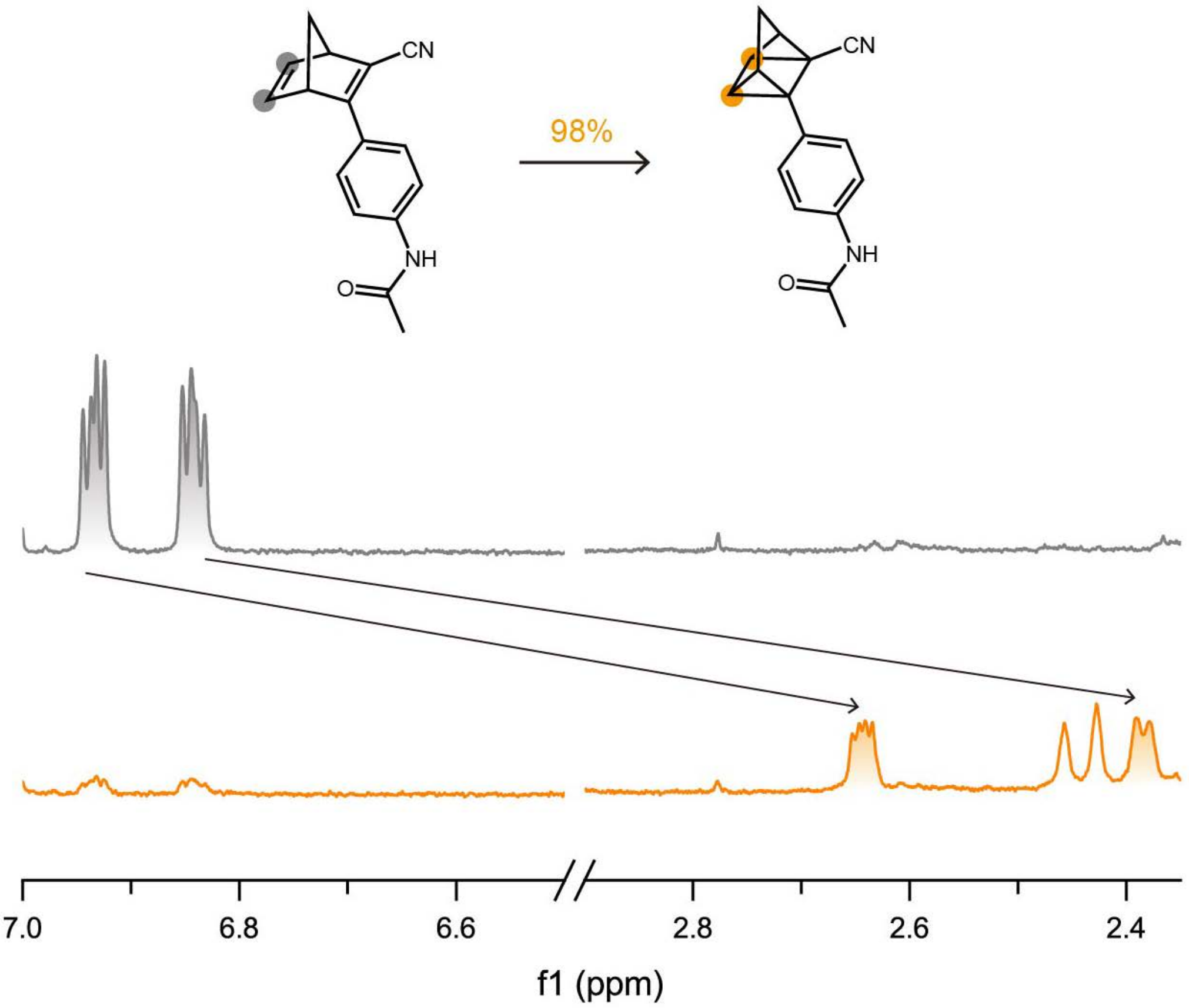

Fig. S29. ^{1}H NMR (400 MHz) spectra and photoconversion yield of NBD3 under 340 nm light irradiation (solvent: $CDCl_3$).

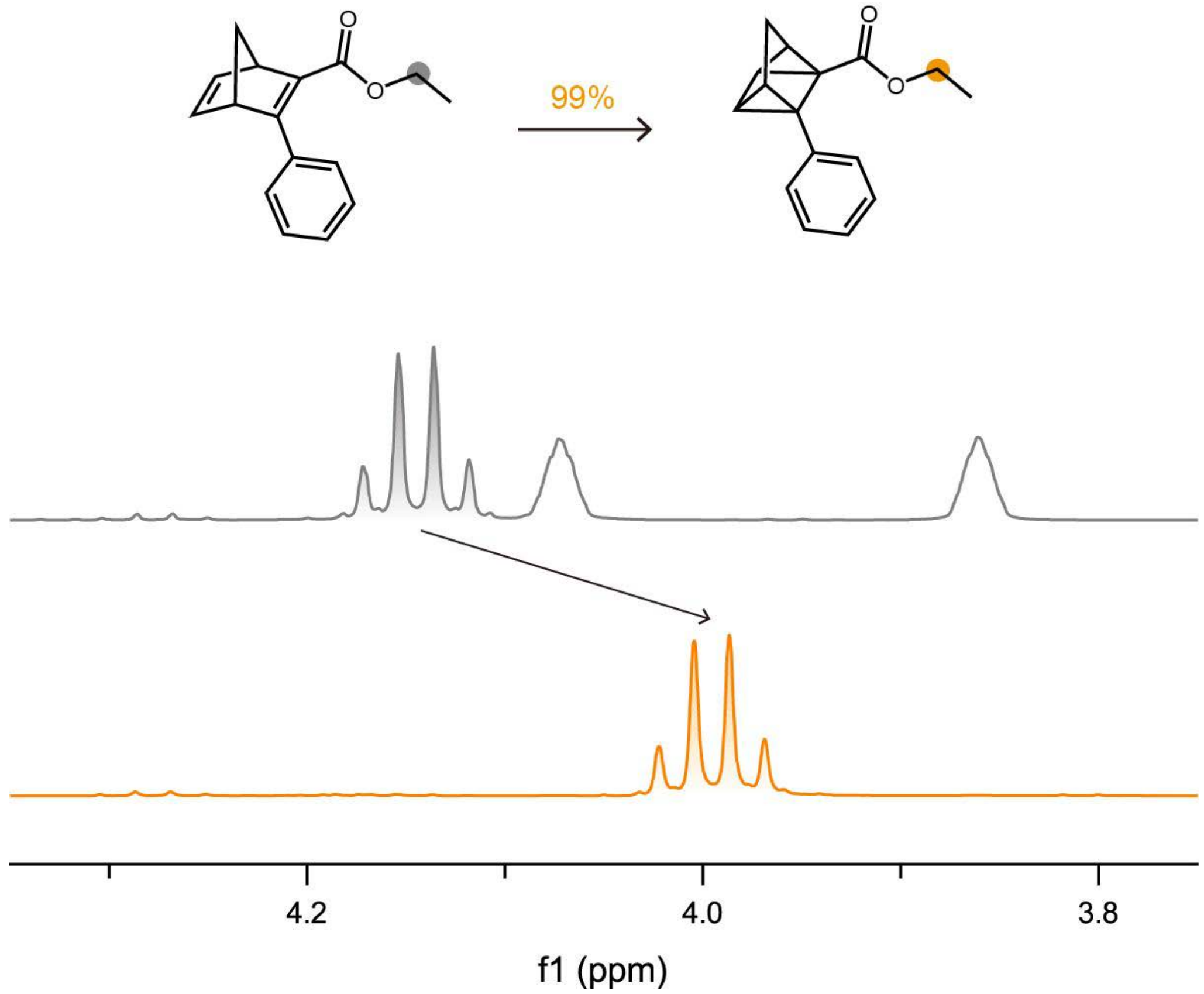

Fig. S30. ^{1}H NMR (400 MHz) spectra and photoconversion yield of NBD4 under 340 nm light irradiation (solvent: $CDCl_3$).

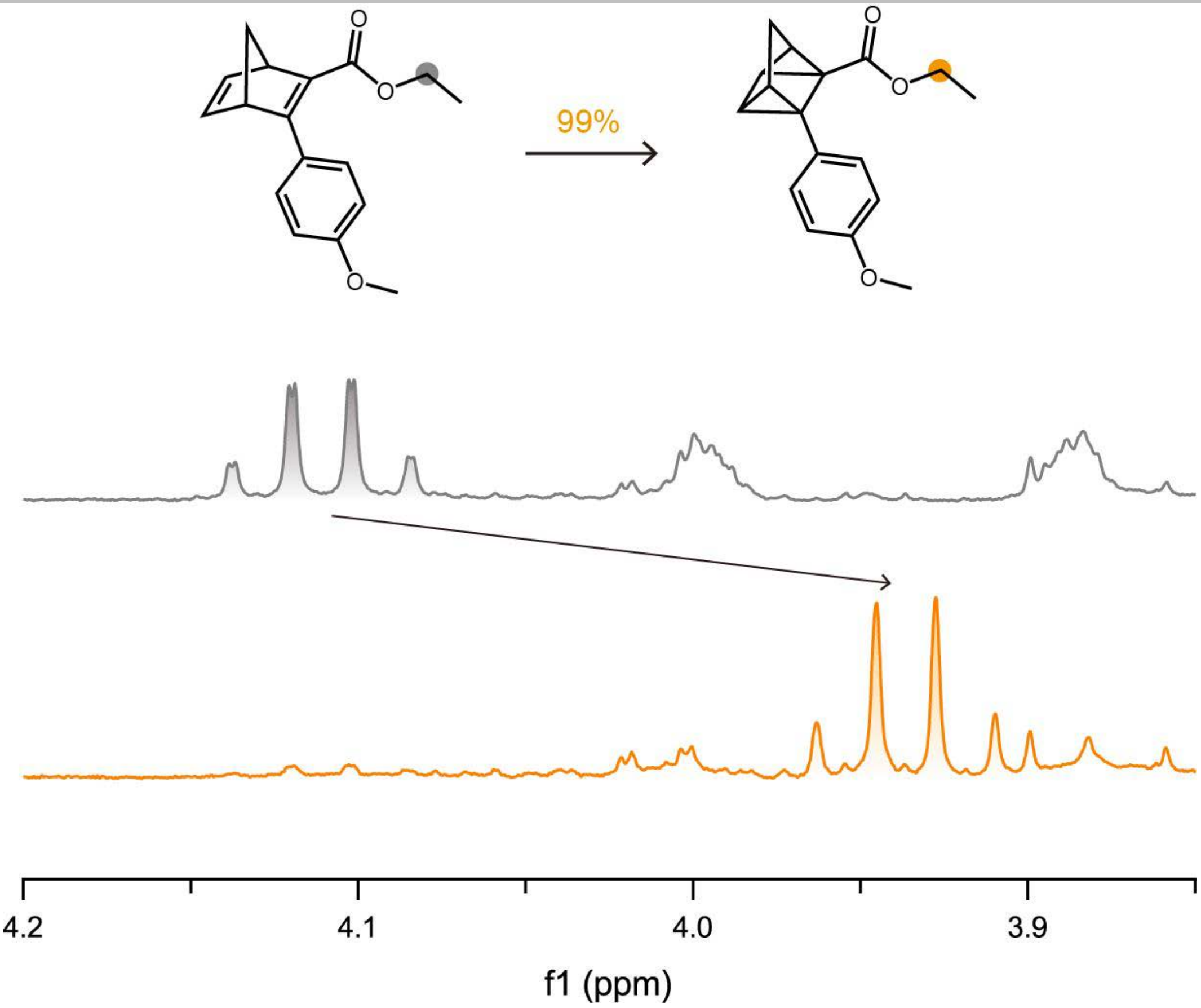

Fig. S31. ^{1}H NMR (400 MHz) spectra and photoconversion yield of NBD5 under 340 nm light irradiation (solvent: $CDCl_3$).

3.2 Thermal backconversion dynamics

3.2.1 Thermal back conversion of NBD/QC pairs in MeCN

NBD1 in anhydrous MeCN (0.1 mM)

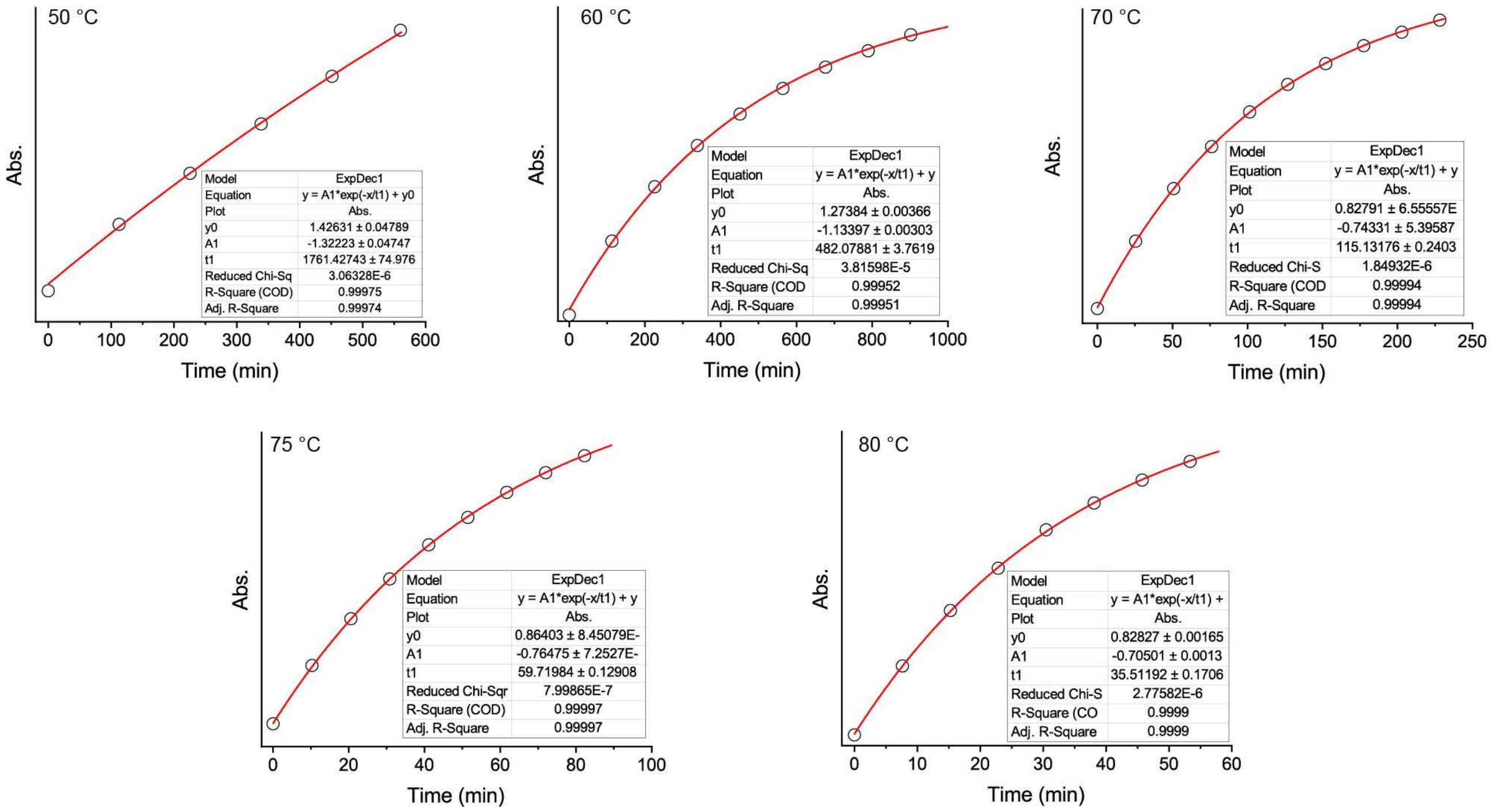

Fig. S32. Thermal back conversion of NBD1 in anhydrous MeCN (0.1 mM) at 50, 60, 70, 75, 80 °C. All the curves are fitted by first-order kinetics equation.

Table S1. Thermal backconversion parameters of NBD1 in anhydrous MeCN (0.1 mM).					
Backconversion rate	Temperature (°C)				
	50	60	70	75	80
k ($\times 10^{-3}$ min^{-1})	0.604	2.23	9.75	16.0	27.0

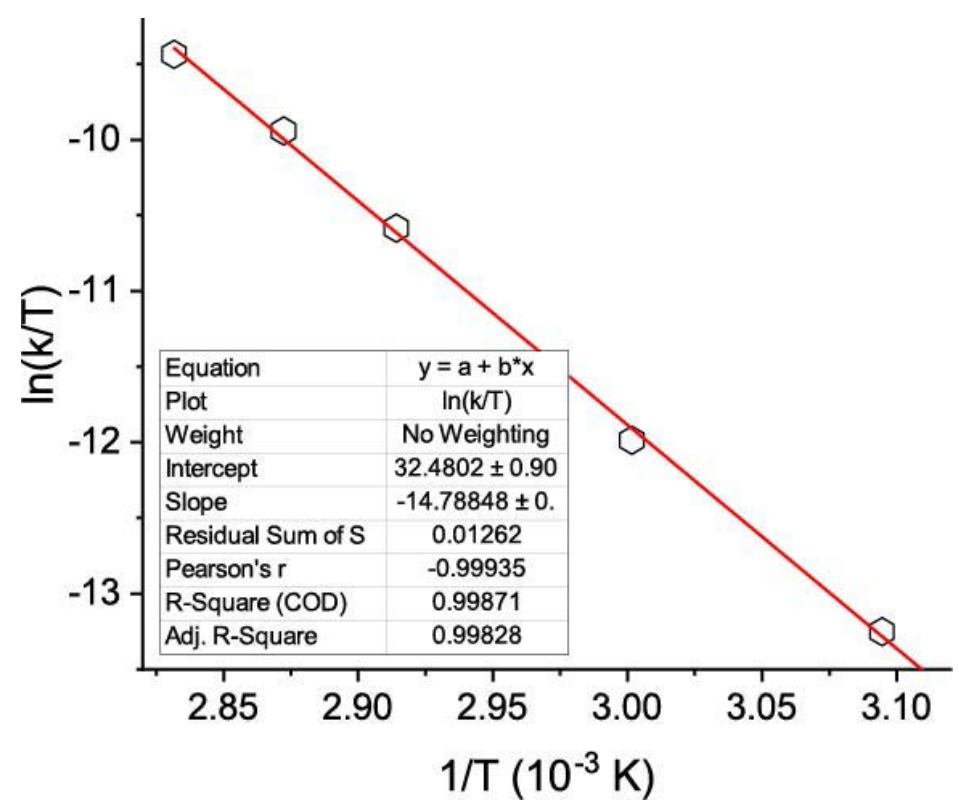

Fig. S33. Eyring plot of NBD1.

NBD1 in MeCN (0.1 mM) with NBD-to-water molar ratio of 1:20

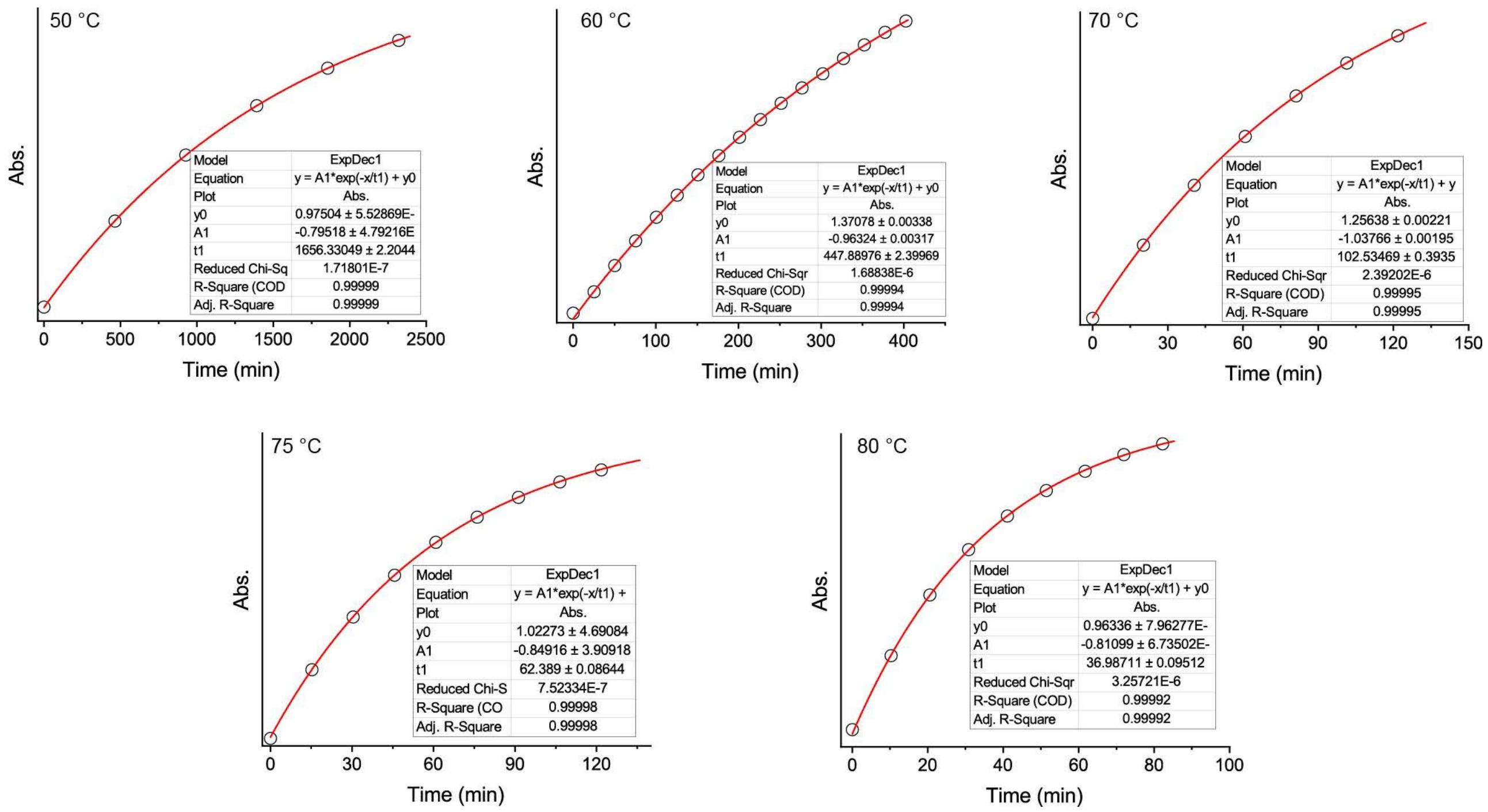

Fig. S34. Thermal back conversion of NBD1 in MeCN (0.1 mM) with NBD-to-water molar ratio of 1:20 at 50, 60, 70, 75, 80 °C. All the curves are fitted by first-order kinetics equation.

Table S2. Thermal backconversion parameters of NBD1 in MeCN (0.1 mM) with NBD-to-water molar ratio of 1:20.					
Backconversion rate	Temperature (°C)				
	50	60	70	75	80
k ($\times 10^{-3}$ min^{-1})	0.568	2.07	8.69	16.7	28.2

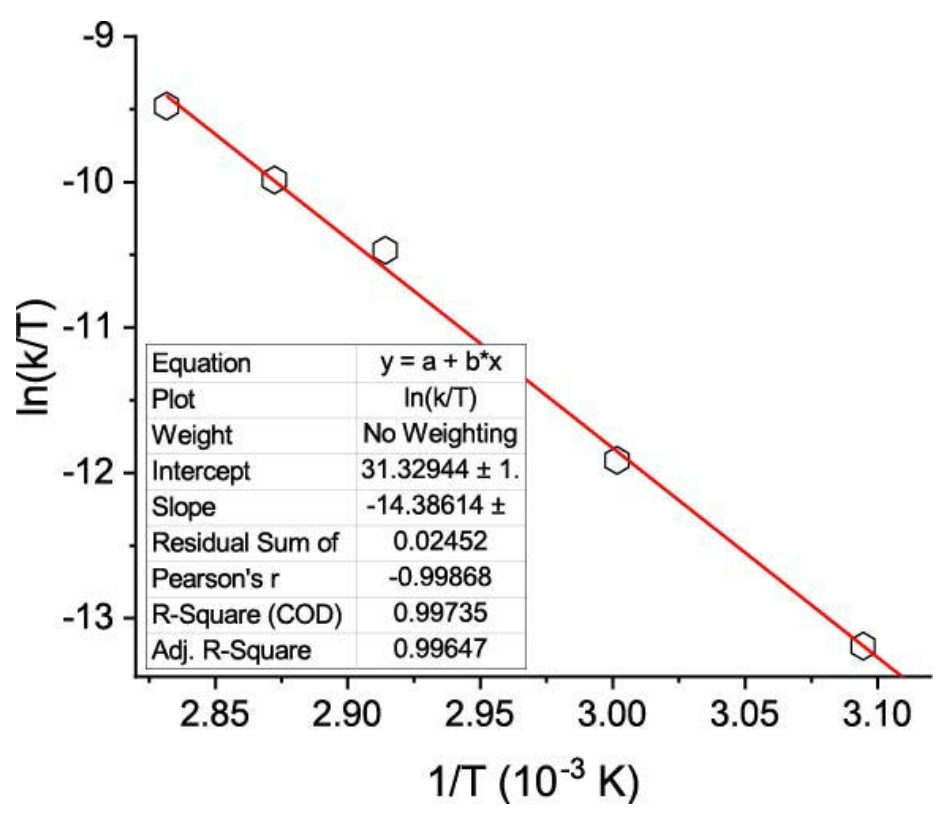

Fig. S35. Eyring plot of NBD1.

NBD2 in anhydrous MeCN (0.1 mM)

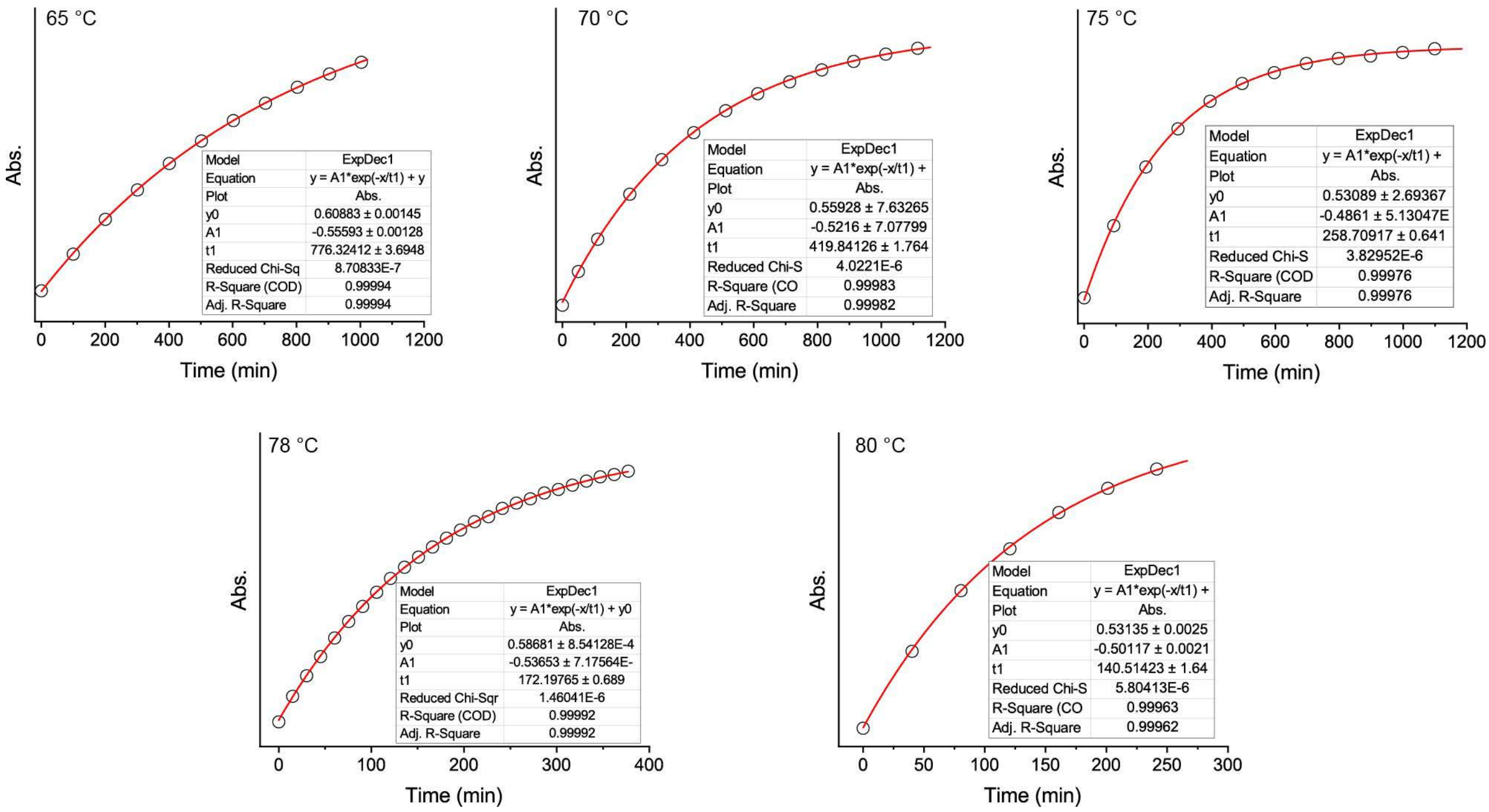

Fig. S36. Thermal back conversion of NBD2 in anhydrous MeCN (0.1 mM) at 65, 70, 75, 78, 80 °C. All the curves are fitted by first-order kinetics equation.

Table S3. Thermal backconversion parameters of NBD2 in anhydrous MeCN (0.1 mM).					
Backconversion rate	Temperature (°C)				
	65	70	75	78	80
k ($\times 10^{-3}$ min^{-1})	1.29	2.38	3.87	5.81	7.12

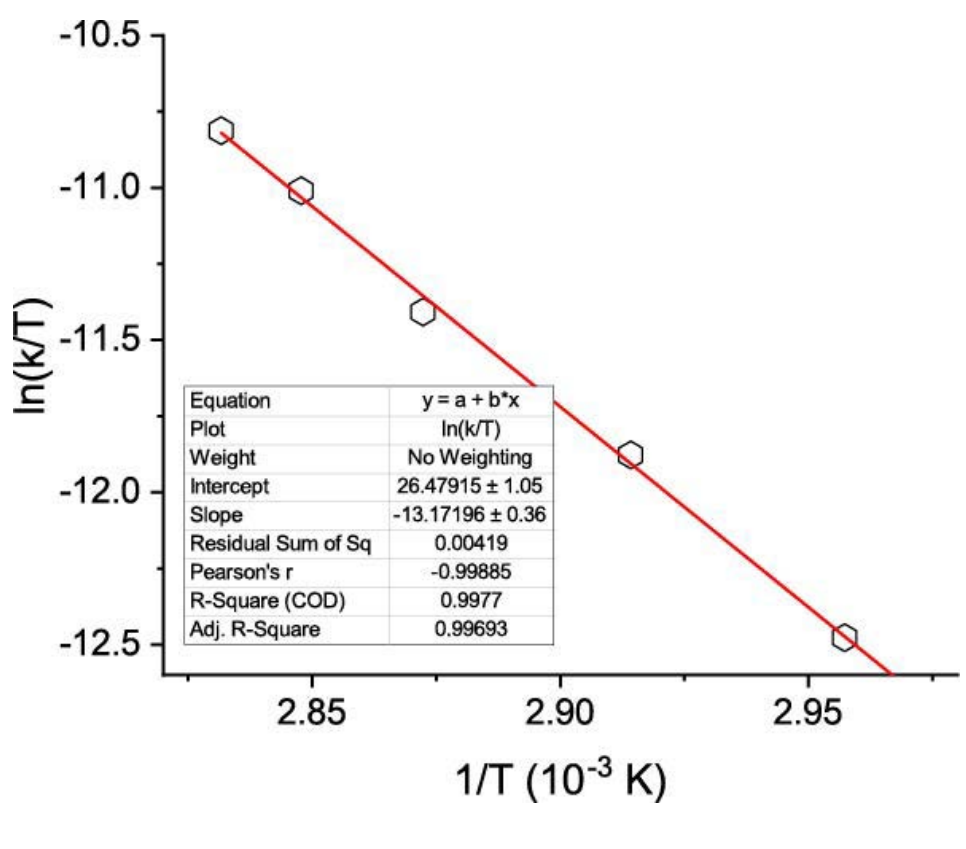

Fig. S37. Eyring plot of NBD2.

NBD2 in MeCN (0.1 mM) with NBD-to-water molar ratio of 1:20

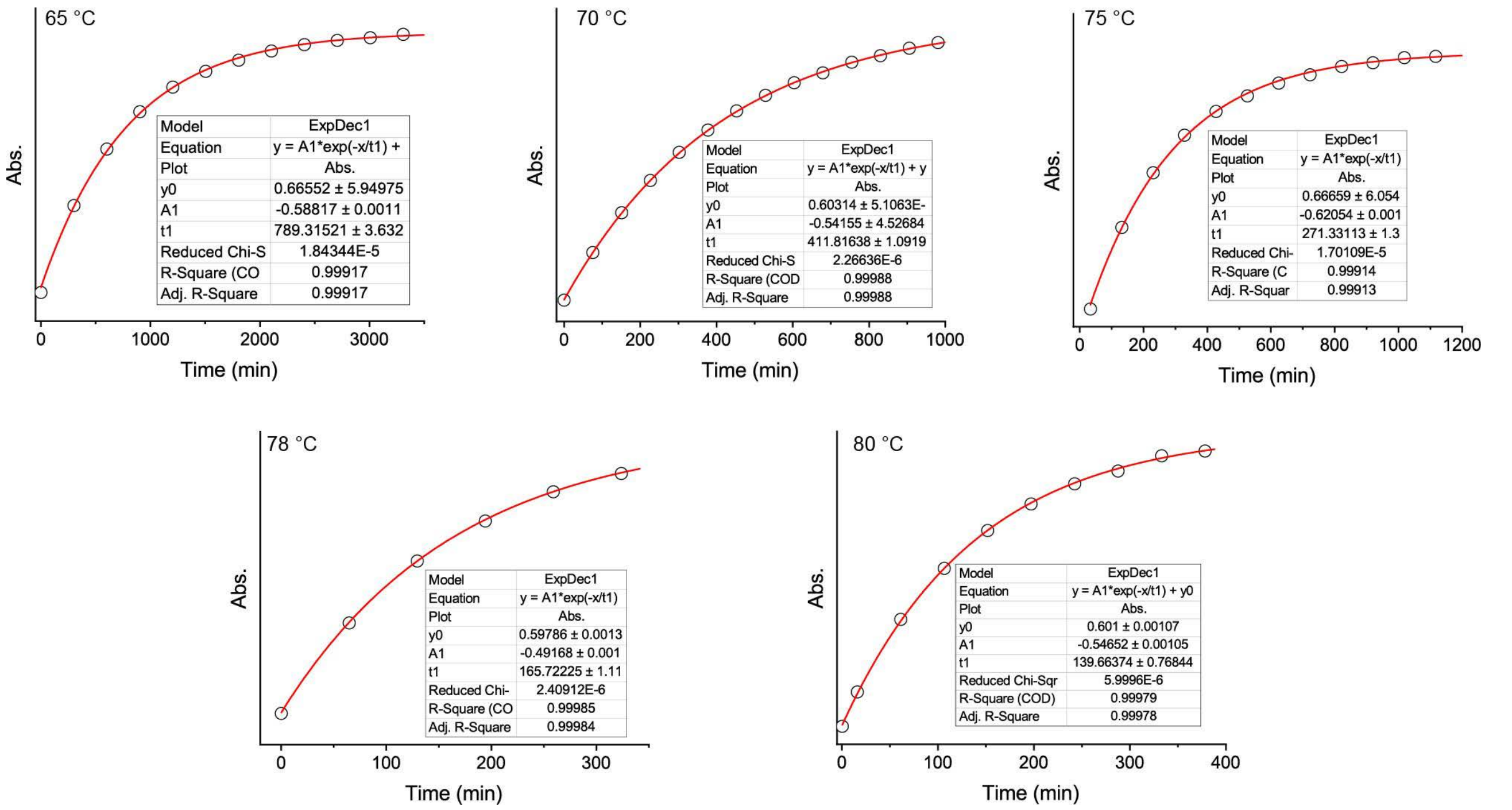

Fig. S38. Thermal back conversion of NBD2 in MeCN (0.1 mM) with NBD-to-water molar ratio of 1:20 at 65, 70, 75, 78, 80 °C. All the curves are fitted by first-order kinetics equation.

Table S4. Thermal backconversion parameters of NBD2 in MeCN (0.1 mM) with NBD-to-water molar ratio of 1:20.					
Backconversion rate	Temperature (°C)				
	65	70	75	78	80
k ($\times10^{-3}$ min^{-1})	1.27	2.43	3.69	6.03	7.16

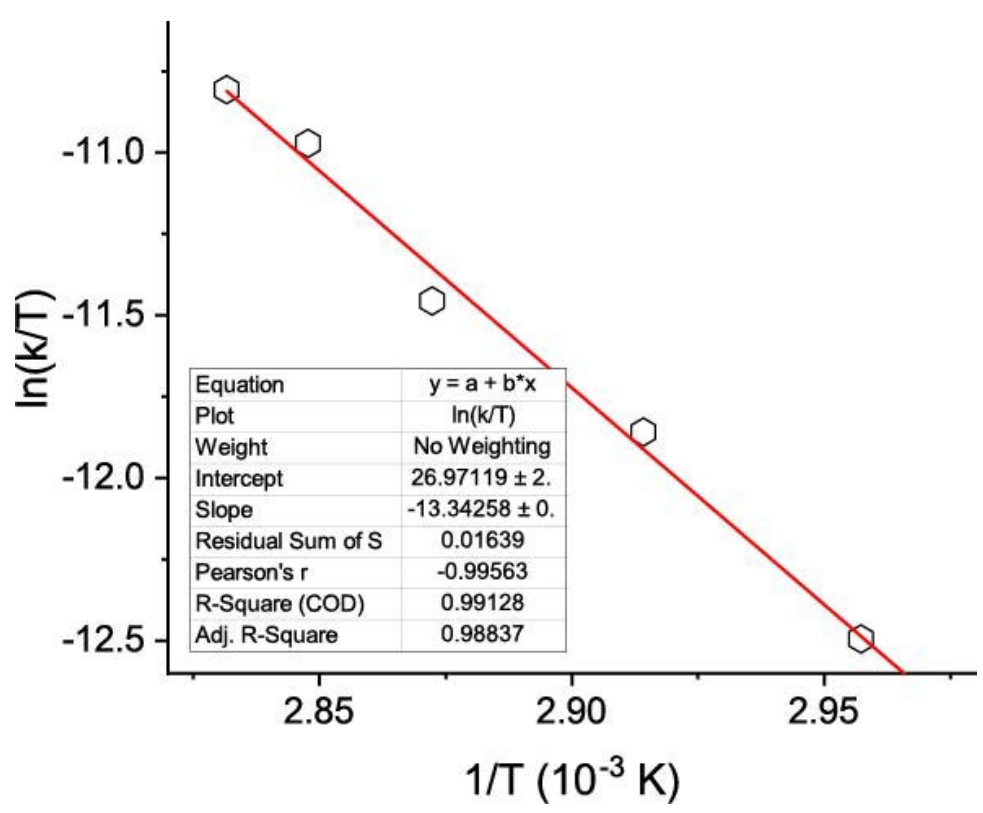

Fig. S39. Eyring plot of NBD2.

NBD3 in anhydrous MeCN (0.1 mM)

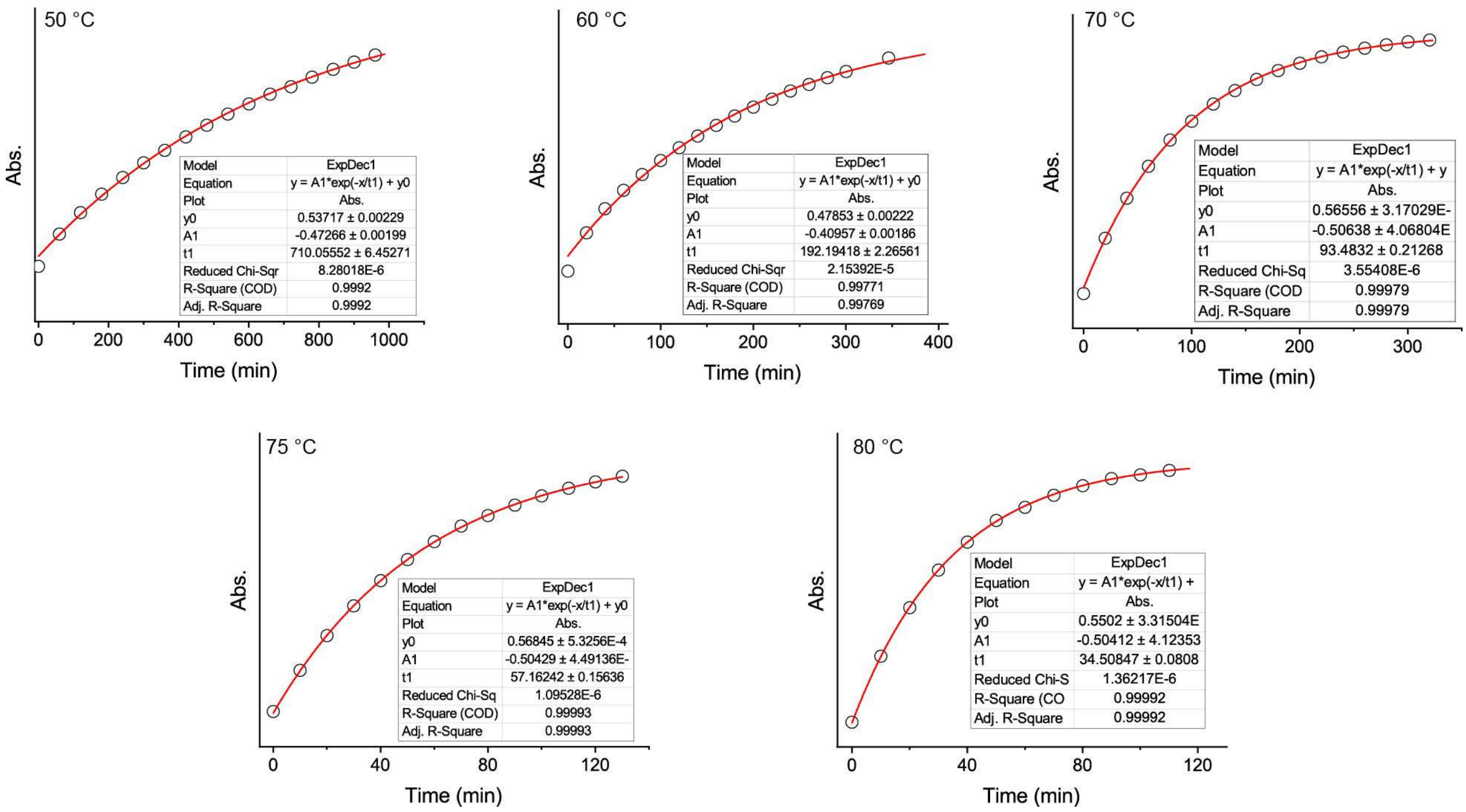

Fig. S40. Thermal back conversion of NBD3 in anhydrous MeCN (0.1 mM) at 50, 60, 70, 75, 80 °C. All the curves are fitted by first-order kinetics equation.

Table S5. Thermal backconversion parameters of NBD3 in anhydrous MeCN (0.1 mM).					
Backconversion rate	Temperature (°C)				
	50	60	70	78	80
k ($\times 10^{-3}$ min^{-1})	1.41	5.20	10.7	17.5	29.0

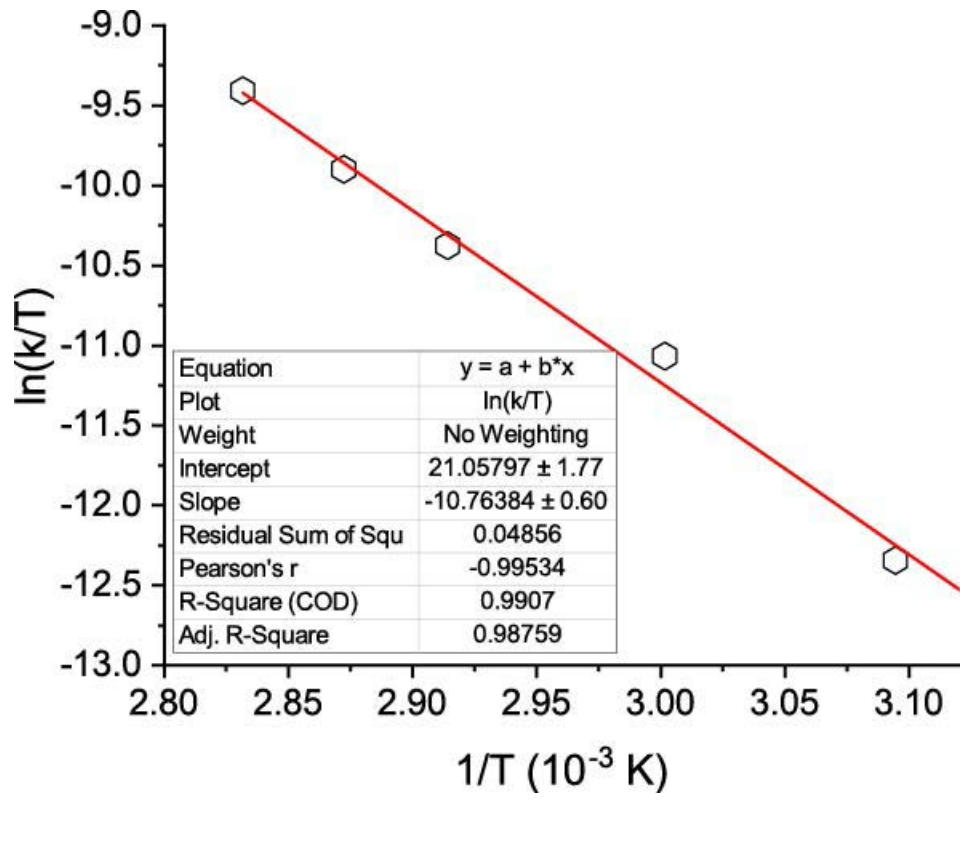

Fig. S41. Eyring plot of NBD3.

NBD3 in MeCN (0.1 mM) with NBD-to-water molar ratio of 1:5

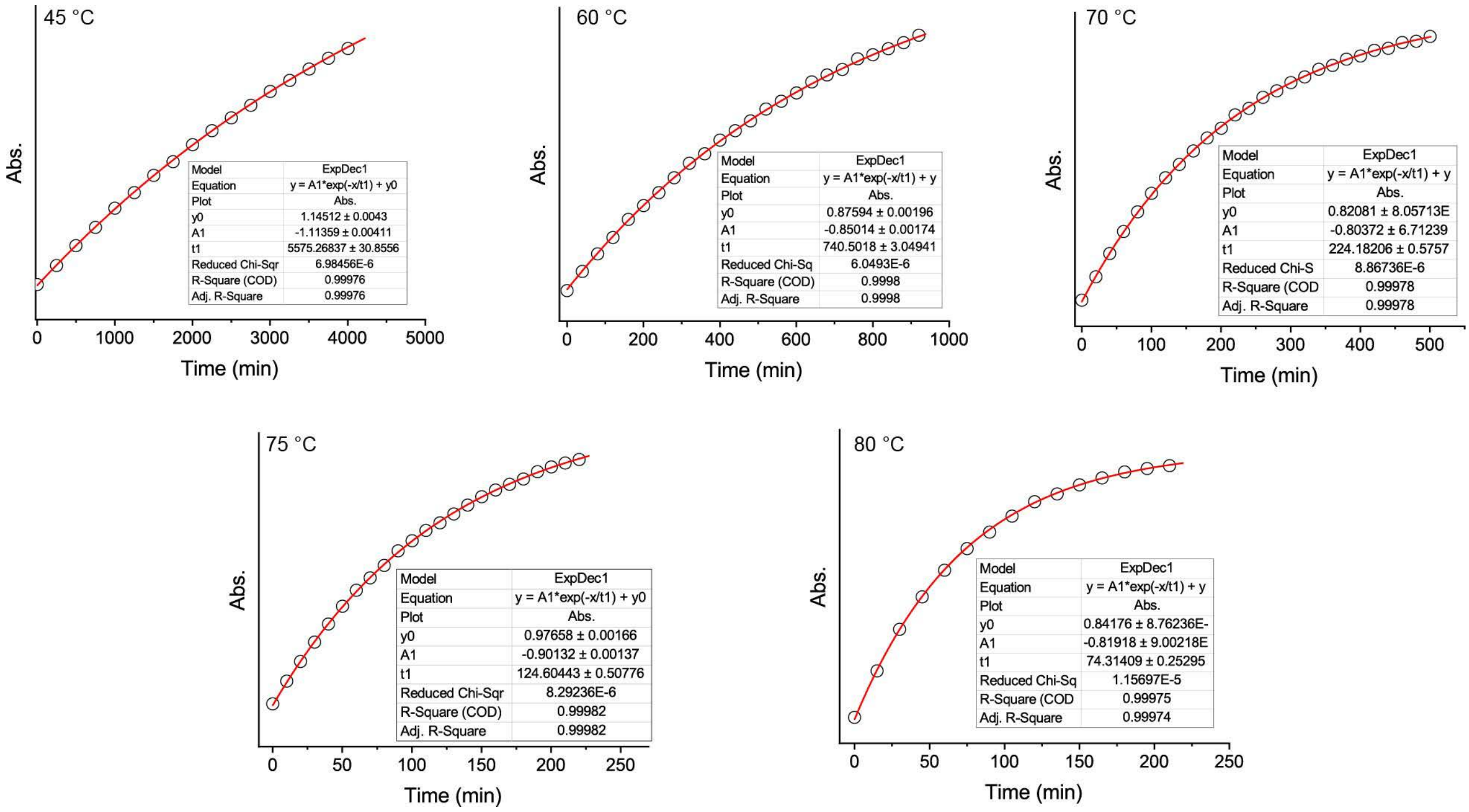

Fig. S42. Thermal back conversion of NBD3 in MeCN (0.1 mM) with NBD-to-water molar ratio of 1:5 at 45, 60, 70, 75, 80 °C. All the curves are fitted by first-order kinetics equation.

Table S6. Thermal backconversion parameters of NBD3 in MeCN (0.1 mM) with NBD-to-water molar ratio of 1:5.					
Backconversion rate	Temperature (°C)				
	45	60	70	75	80
k ($\times 10^{-3}$ min^{-1})	0.179	1.35	4.46	8.03	13.5

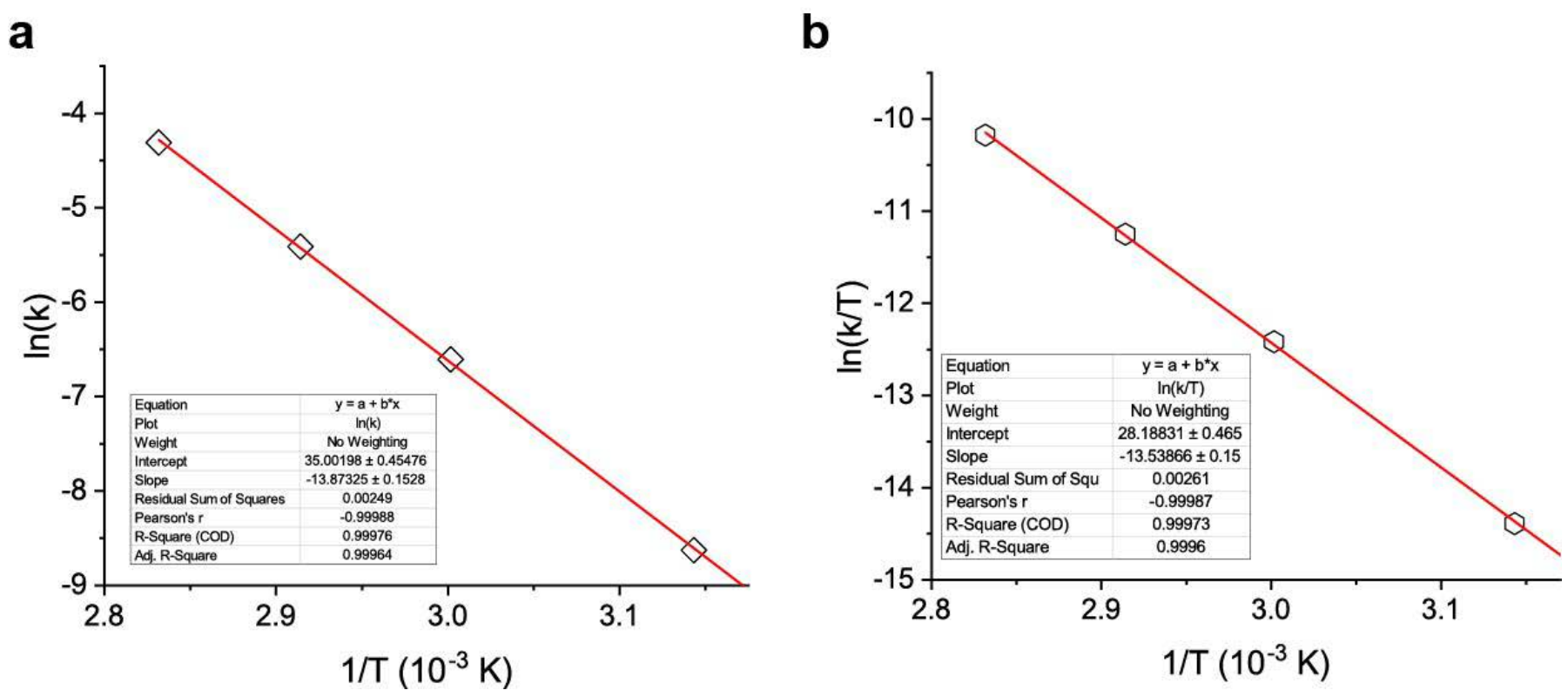

Fig. S43. **a**, Arrhenius plot of NBD3. **b**, Eyring plot of NBD3.

NBD3 in MeCN (0.1 mM) with NBD-to-water molar ratio of 1:20

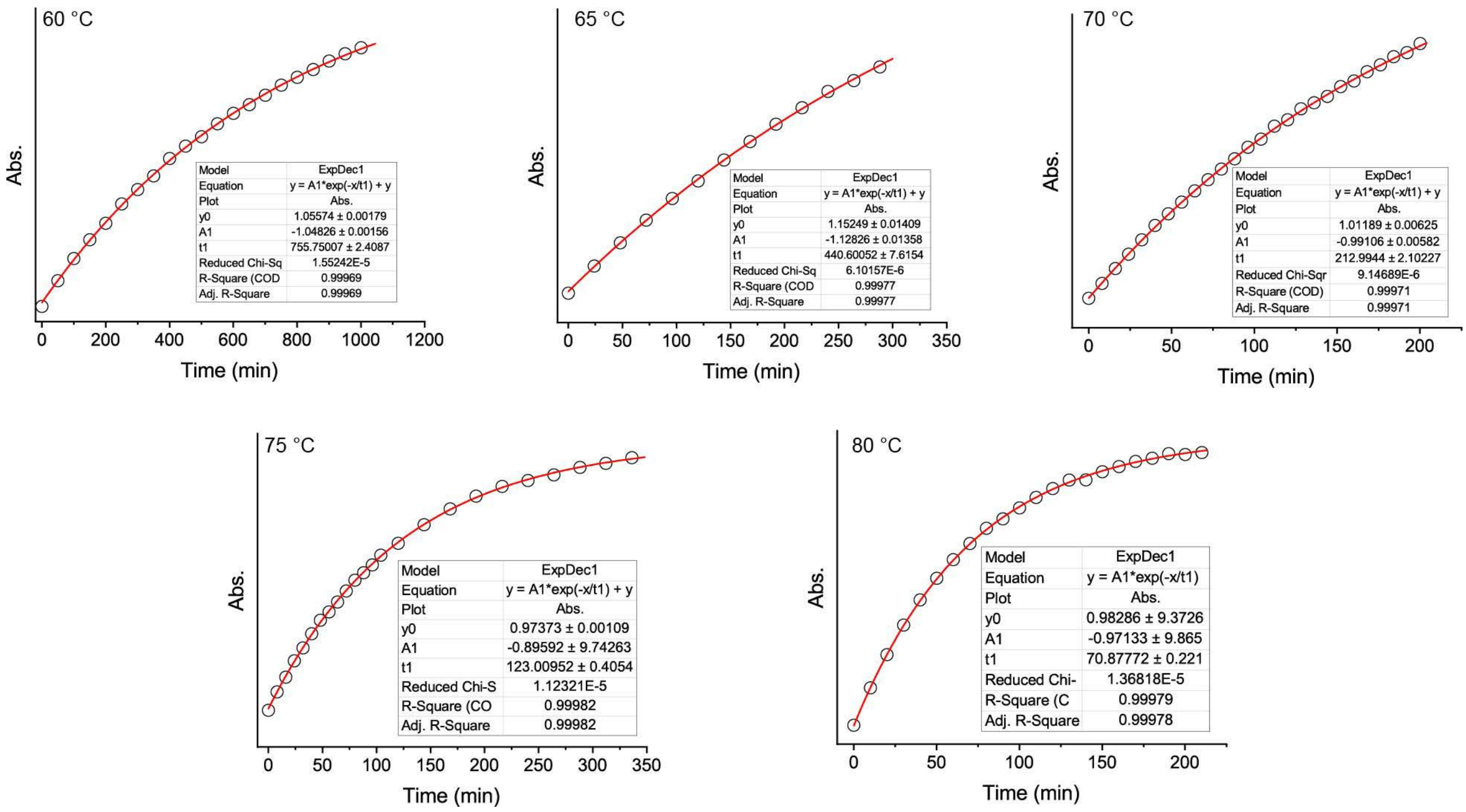

Fig. S44. Thermal back conversion of NBD3 in MeCN (0.1 mM) with NBD-to-water molar ratio of 1:20 at 60, 65, 70, 75, 80 °C. All the curves are fitted by first-order kinetics equation.

Table S7. Thermal backconversion parameters of NBD3 in MeCN (0.1 mM) with NBD-to-water molar ratio of 1:20.					
Backconversion rate	Temperature (°C)				
	60	65	70	75	80
k ($\times 10^{-3}$ min^{-1})	1.32	2.27	4.70	8.13	14.1

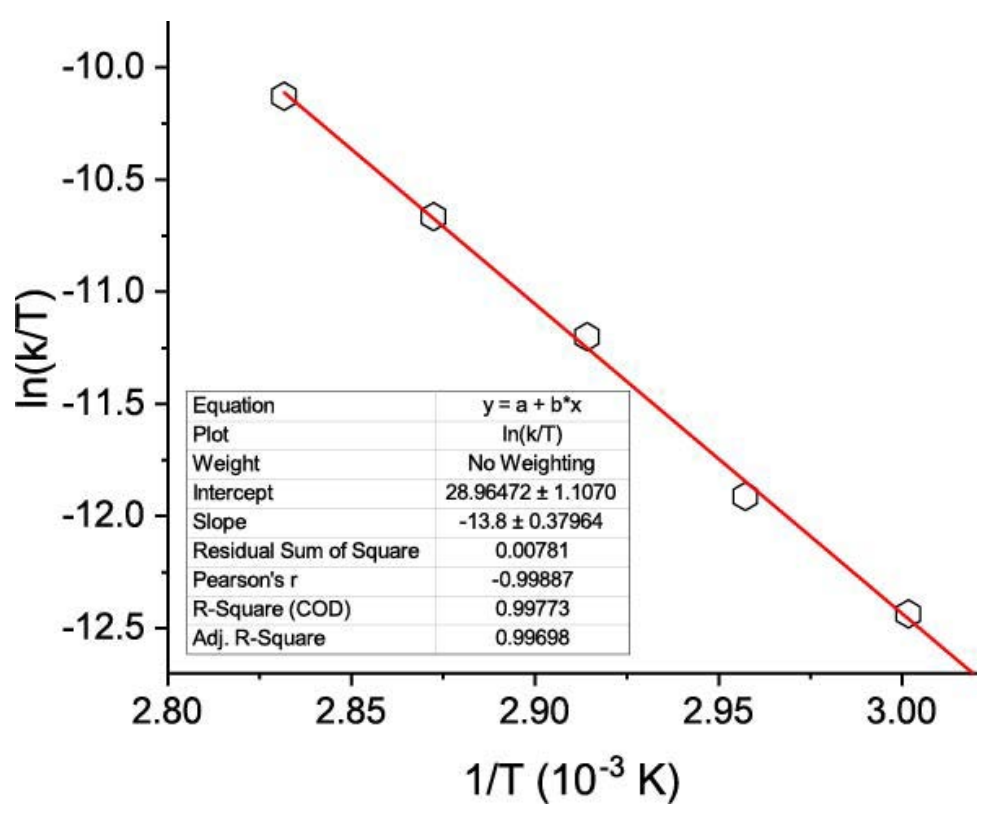

Fig. S45. Eyring plot of NBD3.

NBD4 in anhydrous MeCN (0.1 mM)

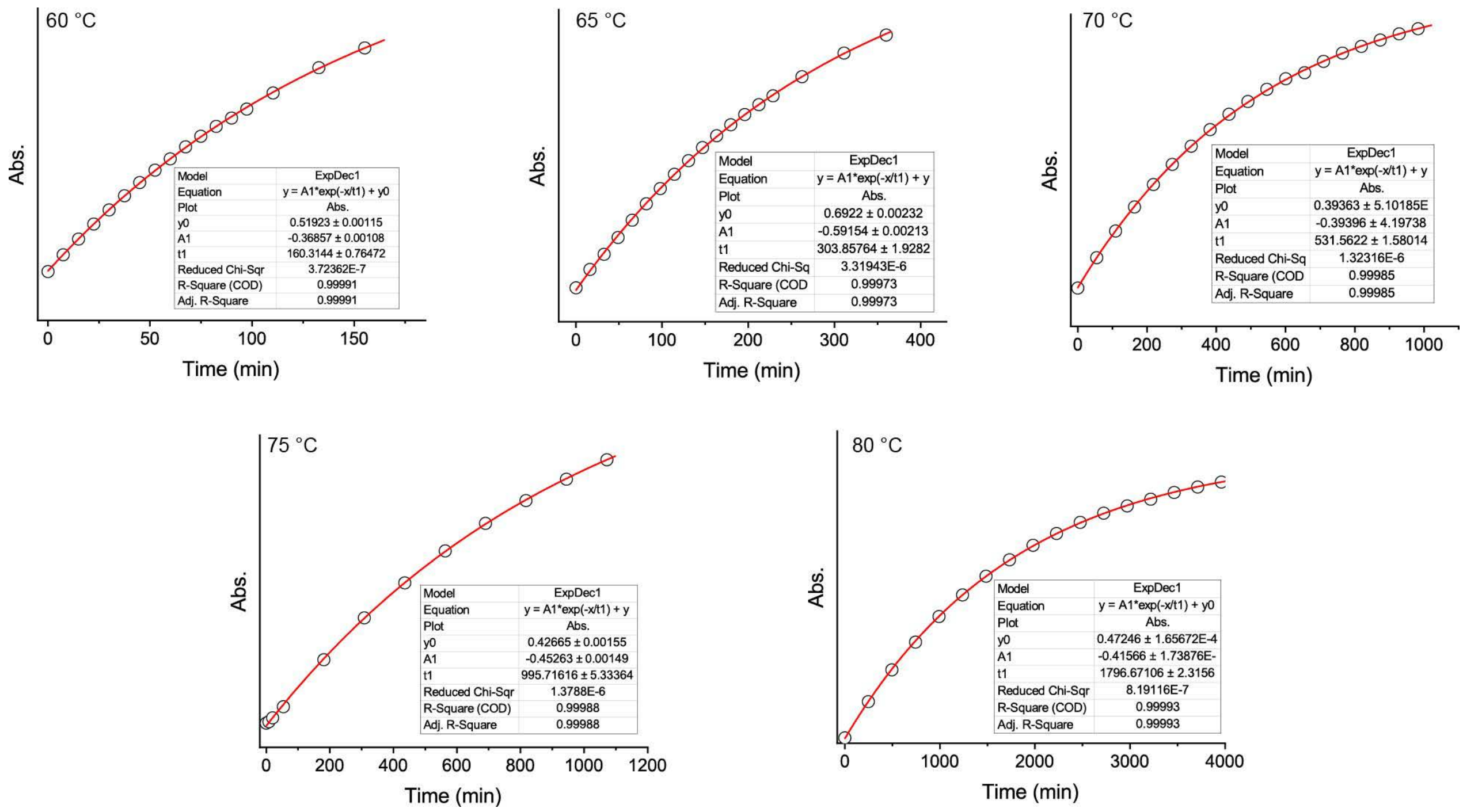

Fig. S46. Thermal back conversion of NBD4 in anhydrous MeCN (0.1 mM) at 60, 65, 70, 75, 80 °C. All the curves are fitted by first-order kinetics equation.

Table S8. Thermal backconversion parameters of NBD4 in anhydrous MeCN (0.1 mM).					
Backconversion rate	Temperature (°C)				
	60	65	70	75	80
k ($\times 10^{-3}$ min^{-1})	0.557	1.00	1.88	3.29	6.24

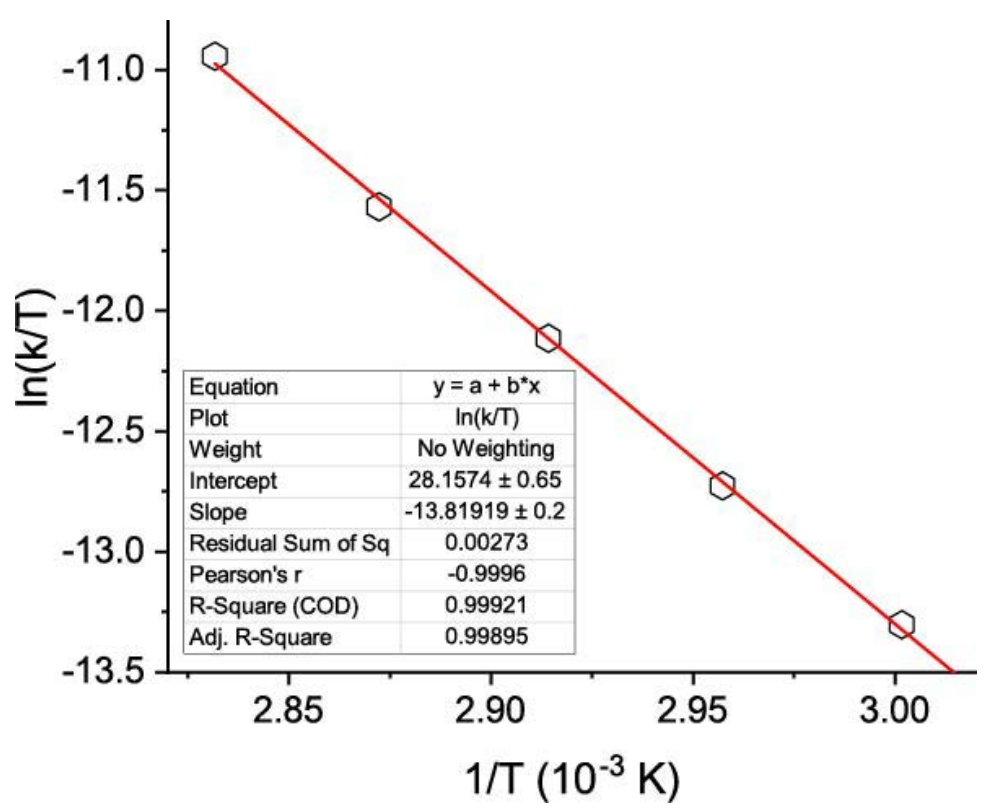

Fig. S47. Eyring plot of NBD4.

NBD4 in MeCN (0.1 mM) with NBD-to-water molar ratio of 1:5

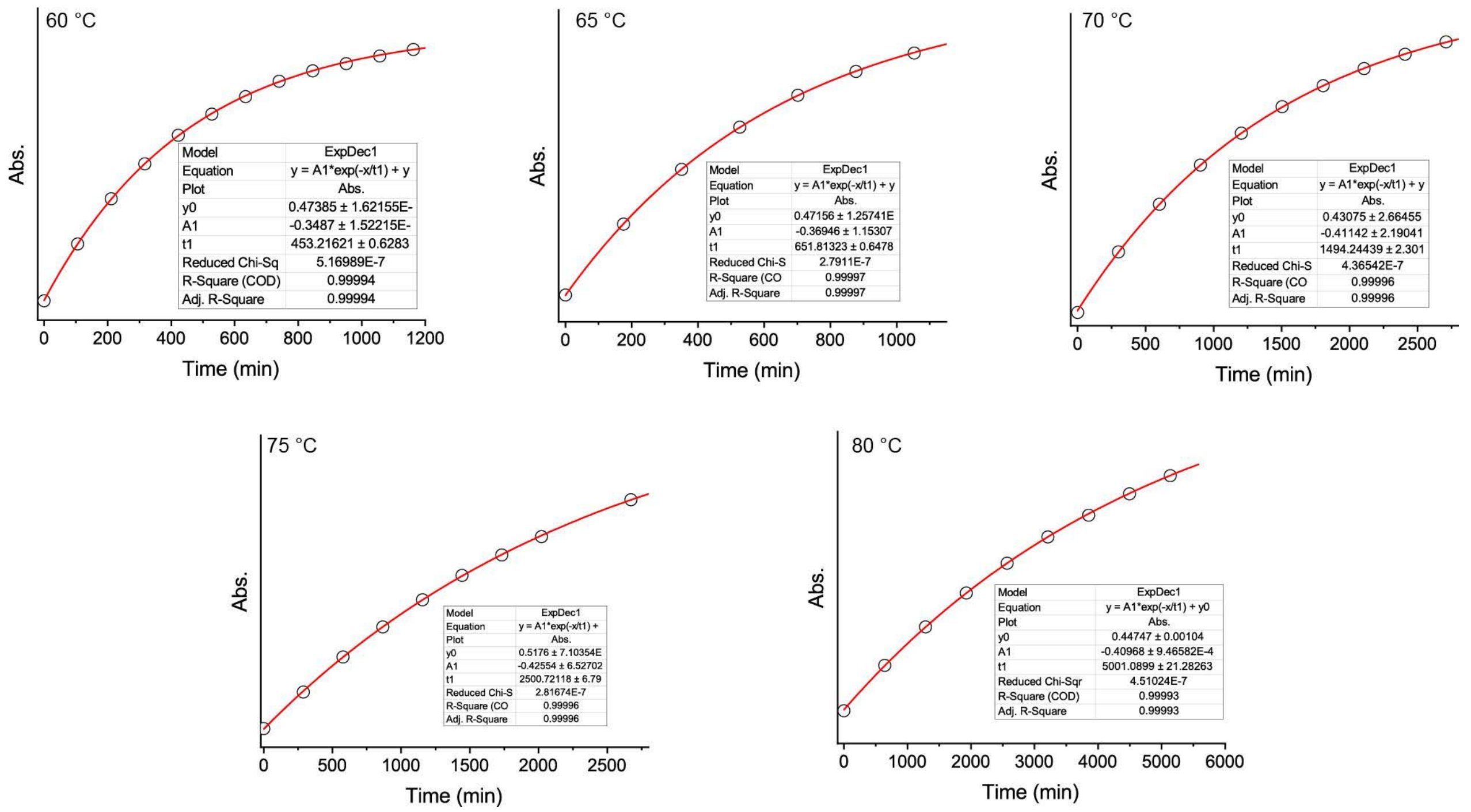

Fig. S48. Thermal back conversion of NBD4 in MeCN (0.1 mM) with NBD-to-water molar ratio of 1:5 at 60, 65, 70, 75, 80 °C. All the curves are fitted by first-order kinetics equation.

Table S9. Thermal backconversion parameters of NBD4 in MeCN (0.1 mM) with NBD-to-water molar ratio of 1:5.					
Backconversion rate	Temperature (°C)				
	60	65	70	75	80
k ($\times 10^{-3}$ min^{-1})	0.200	0.399	0.669	1.53	2.21

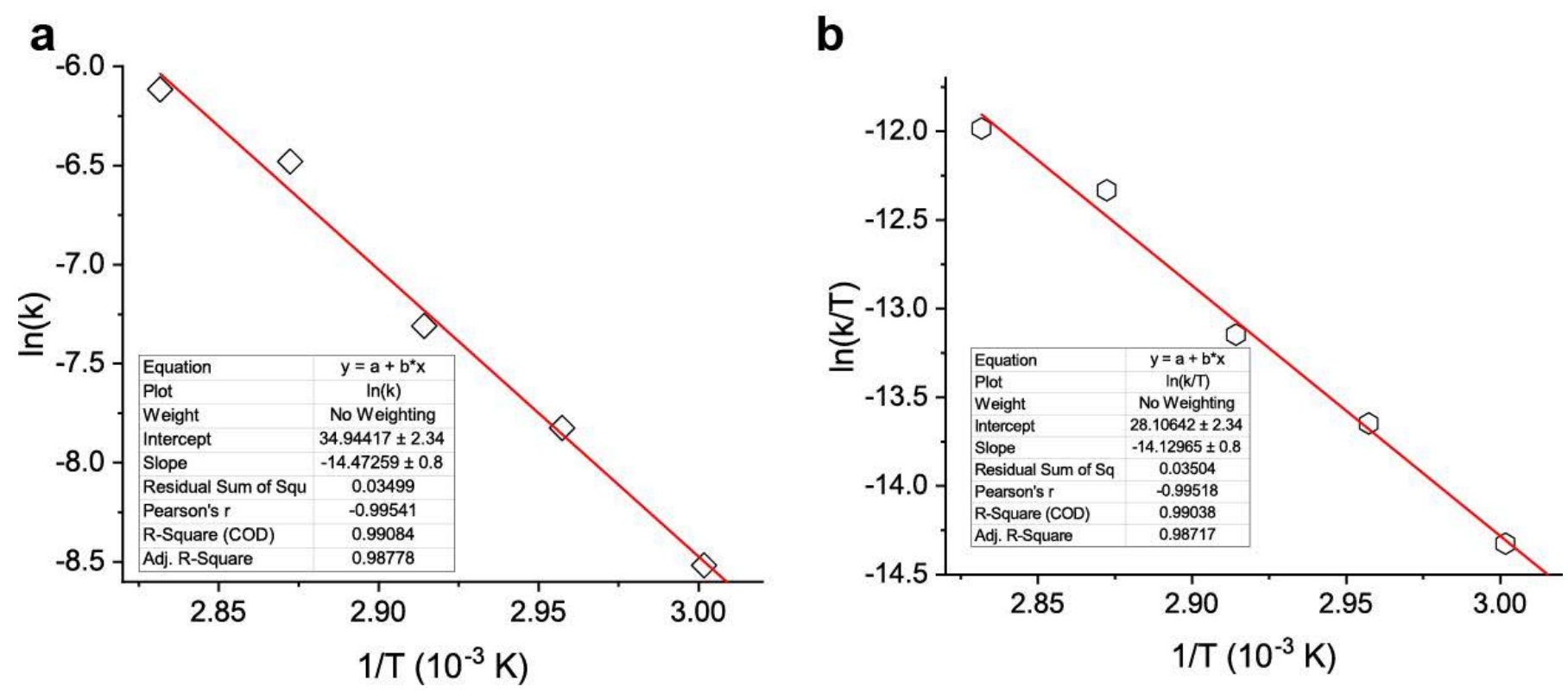

Fig. S49. **a**, Arrhenius plot of NBD4. **b**, Eyring plot of NBD4.

NBD4 in MeCN (0.1 mM) with NBD-to-water molar ratio of 1:20

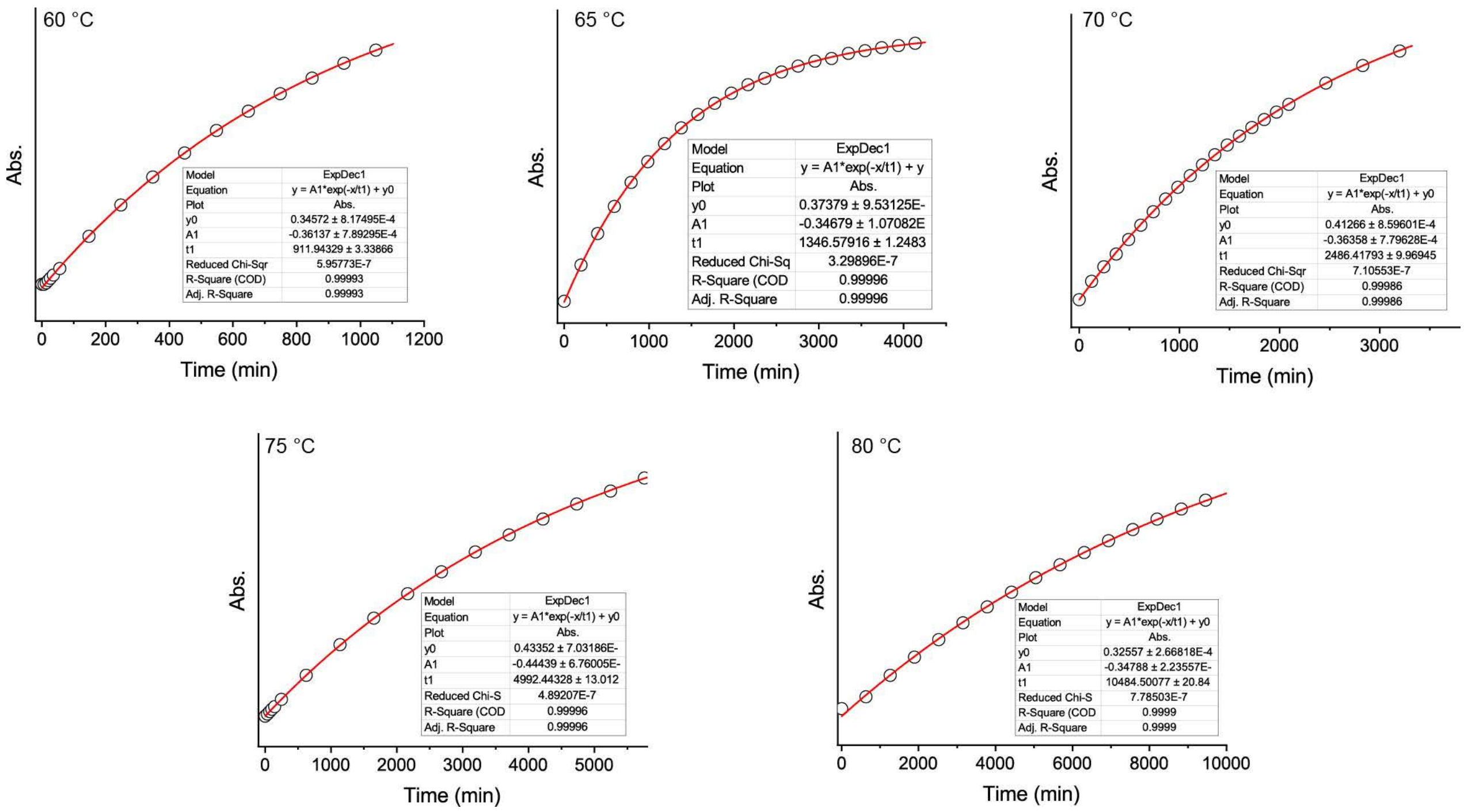

Fig. S50. Thermal back conversion of NBD4 in MeCN (0.1 mM) with NBD-to-water molar ratio of 1:20 at 60, 65, 70, 75, 80 °C. All the curves are fitted by first-order kinetics equation.

Table S10. Thermal backconversion parameters of NBD4 in MeCN (0.1 mM) with NBD-to-water molar ratio of 1:20.					
Backconversion rate	Temperature (°C)				
	60	65	70	75	80
k ($\times 10^{-4}$ min^{-1})	0.954	2.01	4.03	7.43	11.0

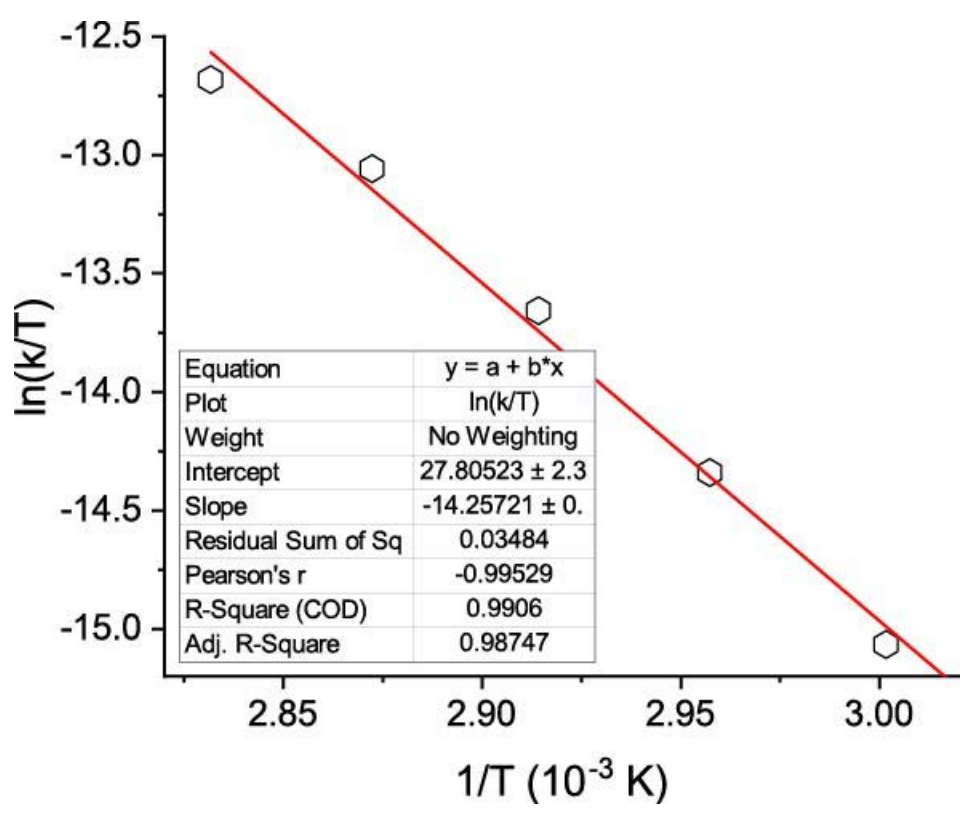

Fig. S51. Eyring plot of NBD4.

NBD5 in anhydrous MeCN (0.1 mM)

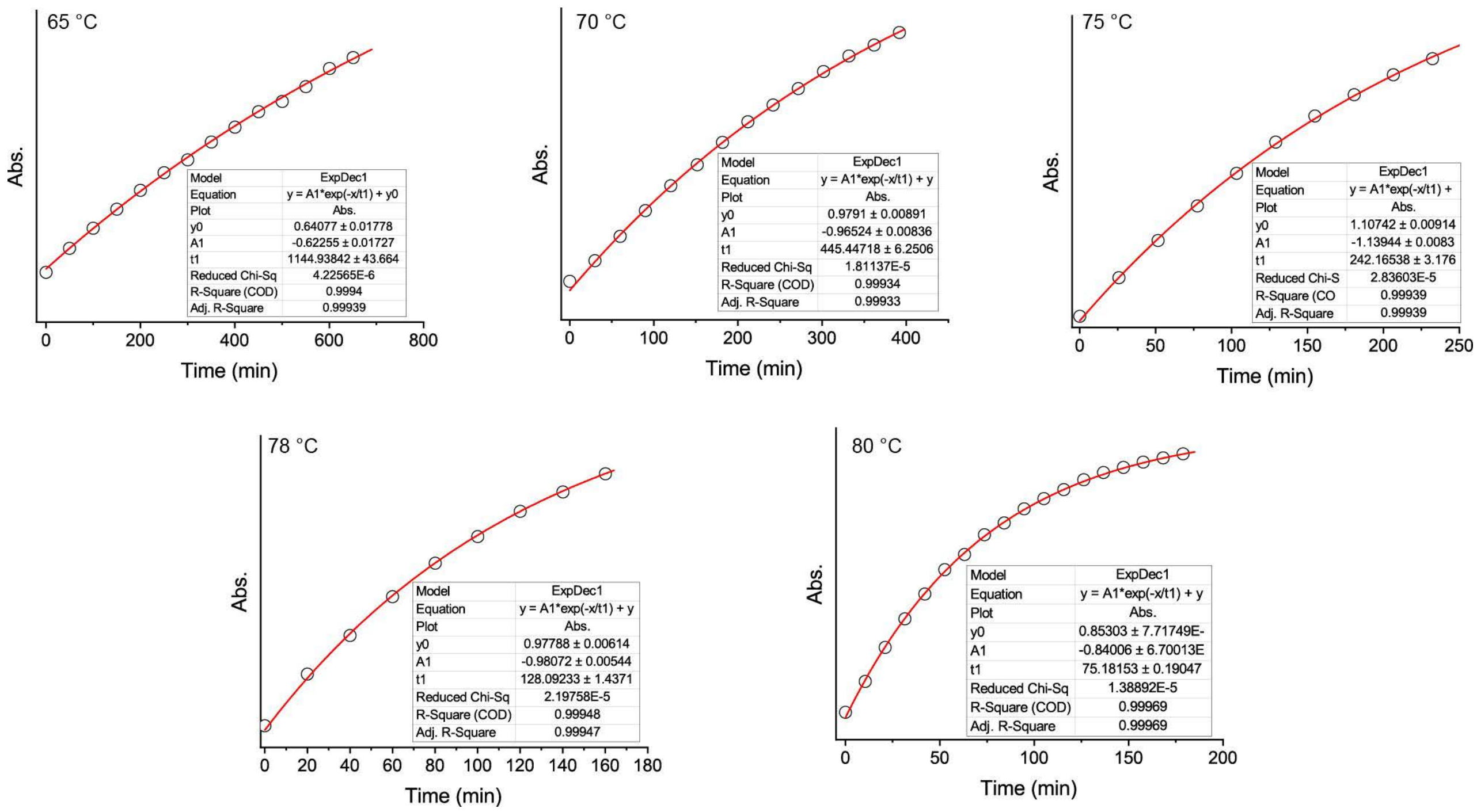

Fig. S52. Thermal back conversion of NBD5 in anhydrous MeCN (0.1 mM) at 65, 70, 75, 78, 80 °C. All the curves are fitted by first-order kinetics equation.

Table S11. Thermal backconversion parameters of NBD5 in anhydrous MeCN (0.1 mM).					
Backconversion rate	Temperature (°C)				
	65	70	75	78	80
k ($\times 10^{-3}$ min^{-1})	2.24	4.13	7.81	11.4	13.3

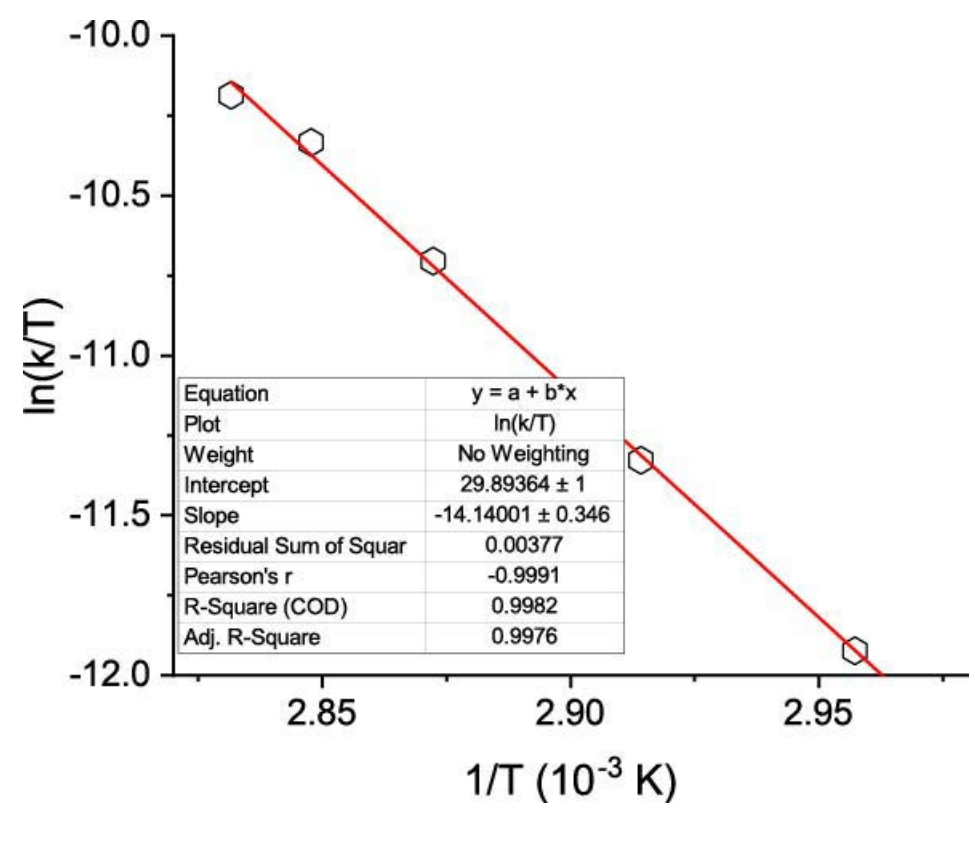

Fig. S53. Eyring plot of NBD5.

NBD5 in MeCN (0.1 mM) with NBD-to-water molar ratio of 1:5

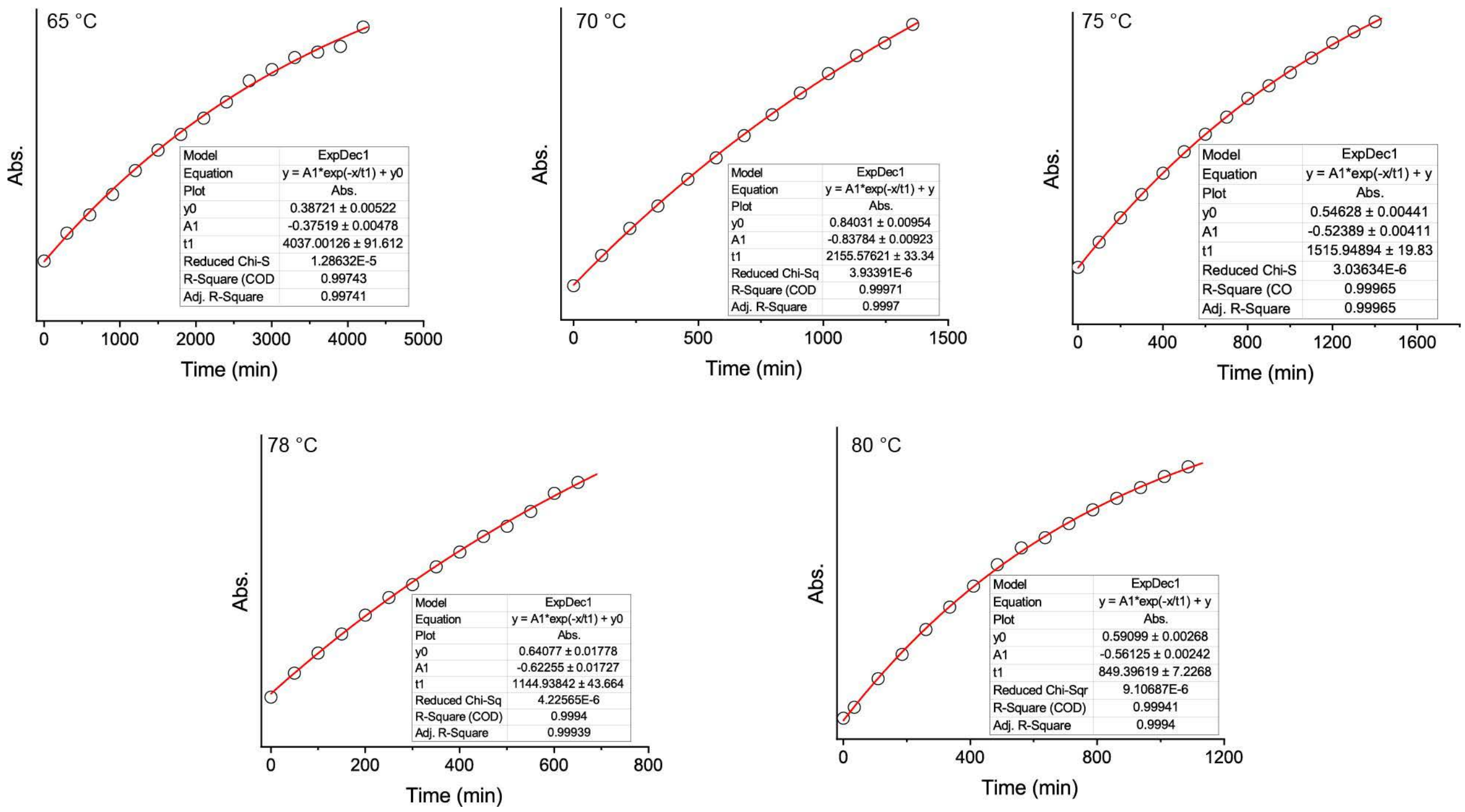

Fig. S54. Thermal back conversion of NBD5 in MeCN (0.1 mM) with NBD-to-water molar ratio of 1:5 at 65, 70, 75, 78, 80 °C. All the curves are fitted by first-order kinetics equation.

Table S12. Thermal backconversion parameters of NBD5 in MeCN (0.1 mM) with NBD-to-water molar ratio of 1:20.					
Backconversion rate	Temperature (°C)				
	65	70	75	78	80
k ($\times10^{-3}$ min^{-1})	2.48	4.64	6.60	8.73	11.7

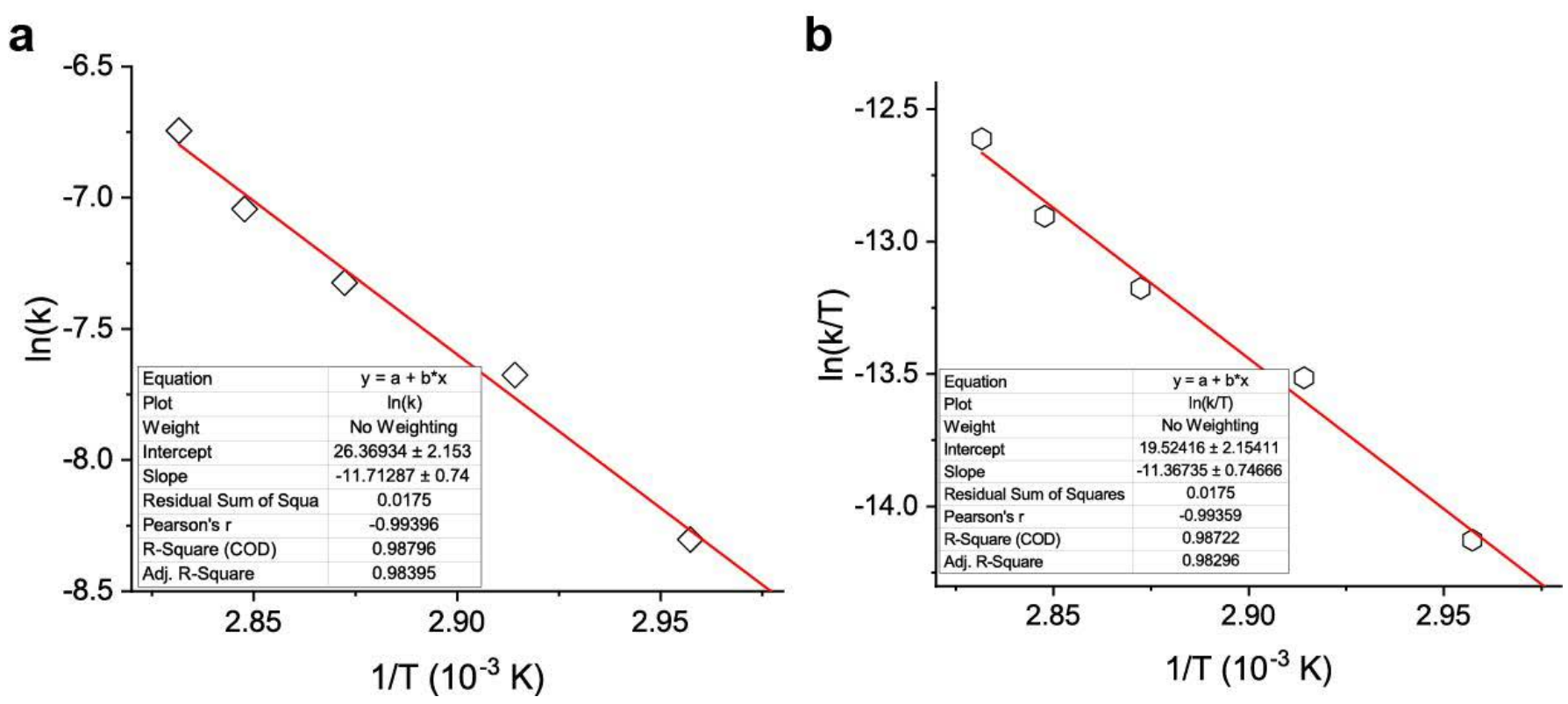

Fig. S55. **a**, Arrhenius plot of NBD5. **b**, Eyring plot of NBD5.

NBD5 in MeCN (0.1 mM) with NBD-to-water molar ratio of 1:20

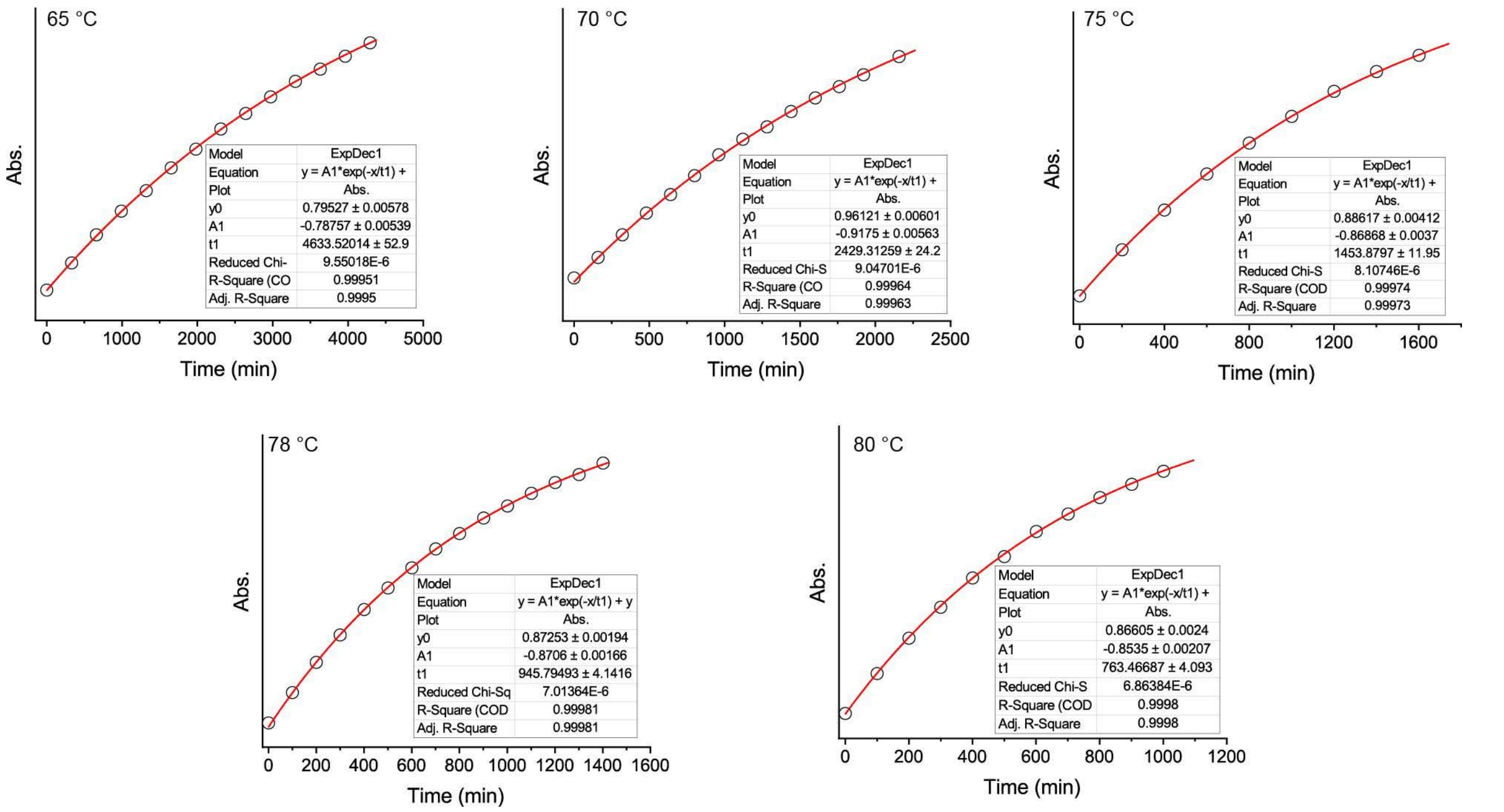

Fig. S56. Thermal back conversion of NBD5 in MeCN (0.1 mM) with NBD-to-water molar ratio of 1:20 at 65, 70, 75, 78, 80 °C. All the curves are fitted by first-order kinetics equation.

Table S13. Thermal backconversion parameters of NBD5 in MeCN (0.1 mM) with NBD-to-water molar ratio of 1:20.					
Backconversion rate	Temperature (°C)				
	65	70	75	78	80
k ($\times 10^{-3}$ min^{-1})	2.16	4.12	6.88	10.6	13.1

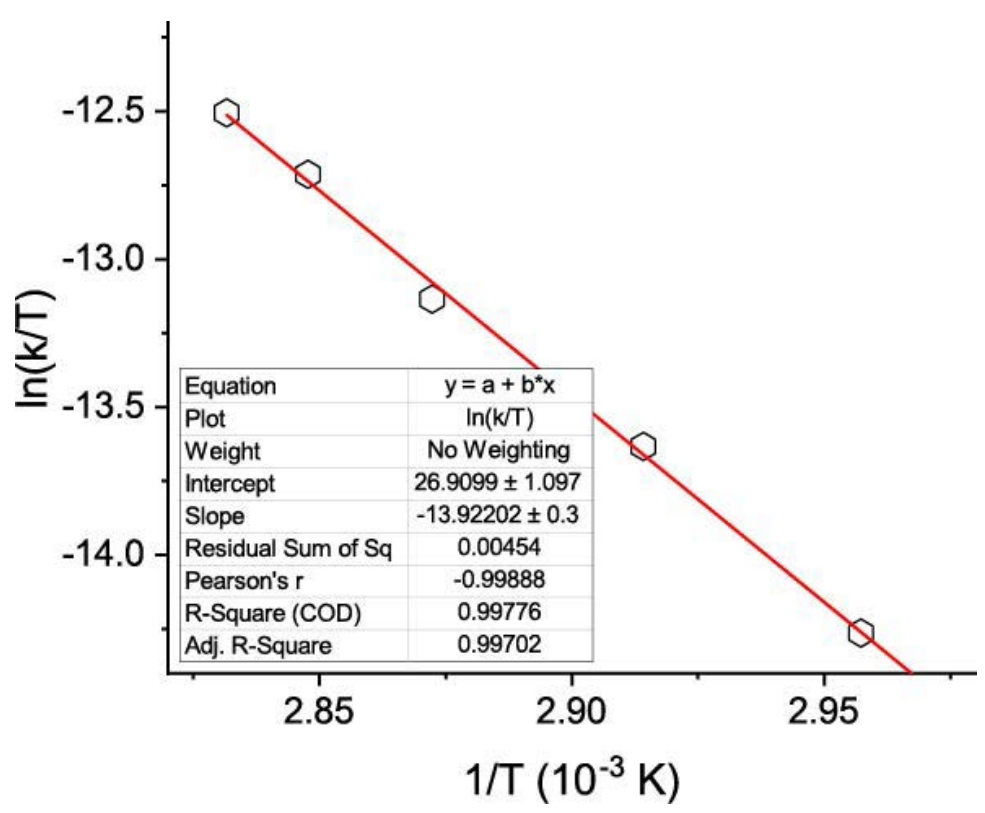

Fig. S57. Eyring plot of NBD5.

NBD6 in anhydrous MeCN (0.1 mM)

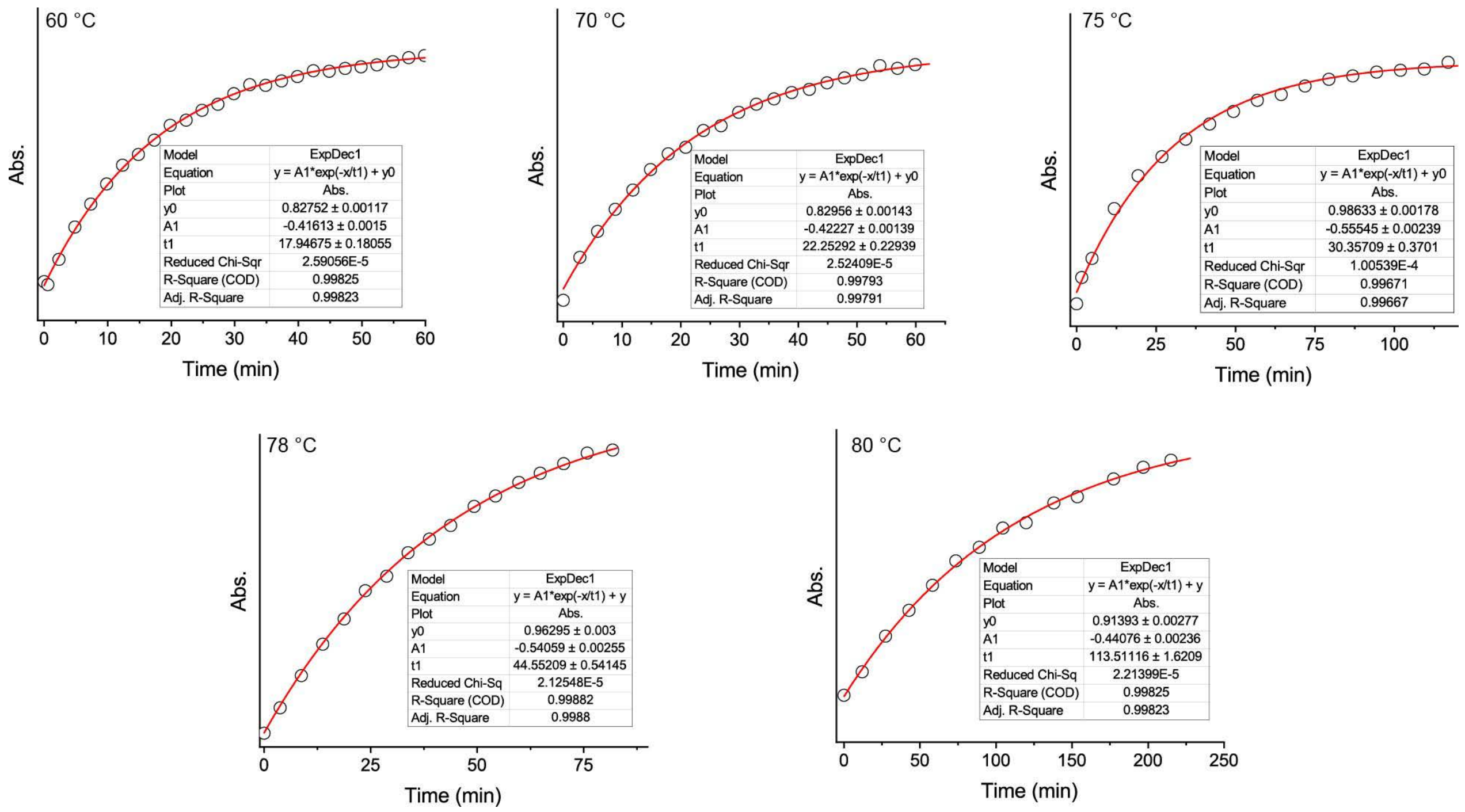

Fig. S58. Thermal back conversion of NBD6 in anhydrous MeCN (0.1 mM) at 60, 70, 75, 78, 80 °C. All the curves are fitted by first-order kinetics equation.

Table S14. Thermal backconversion parameters of NBD6 in anhydrous MeCN (0.1 mM).					
Backconversion rate	Temperature (°C)				
	60	70	75	78	80
k ($\times 10^{-2}$ min^{-1})	0.881	2.24	3.29	4.48	5.59

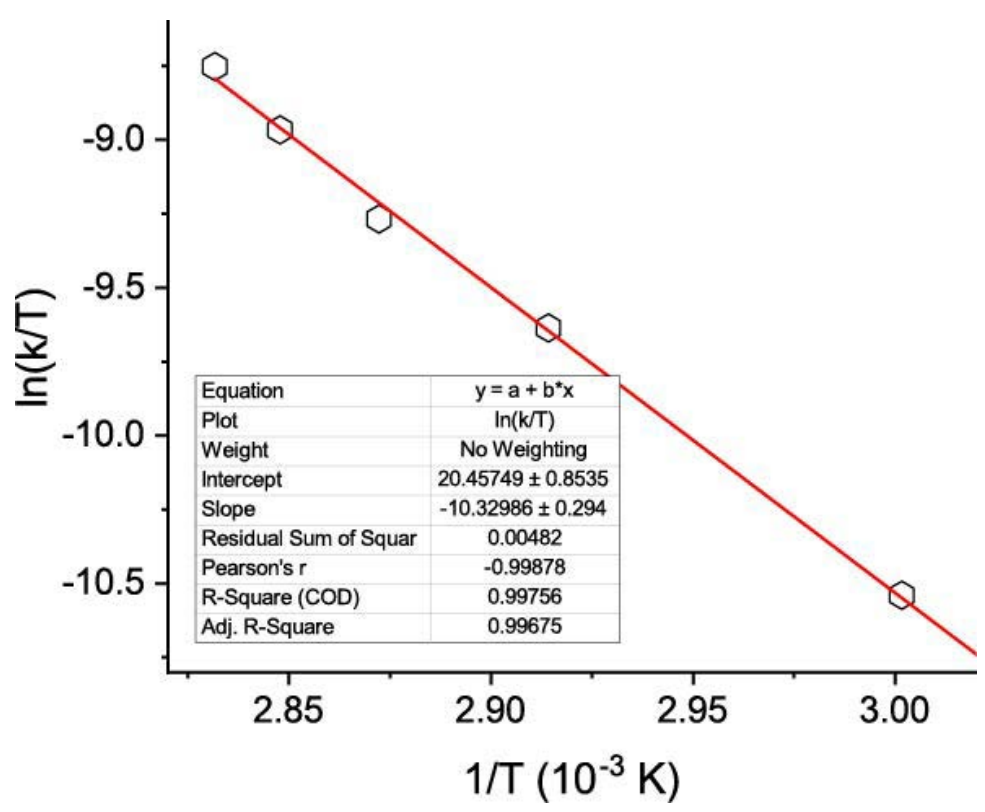

Fig. S59. Eyring plot of NBD6.

NBD6 in MeCN (0.1 mM) with NBD-to-water molar ratio of 1:5

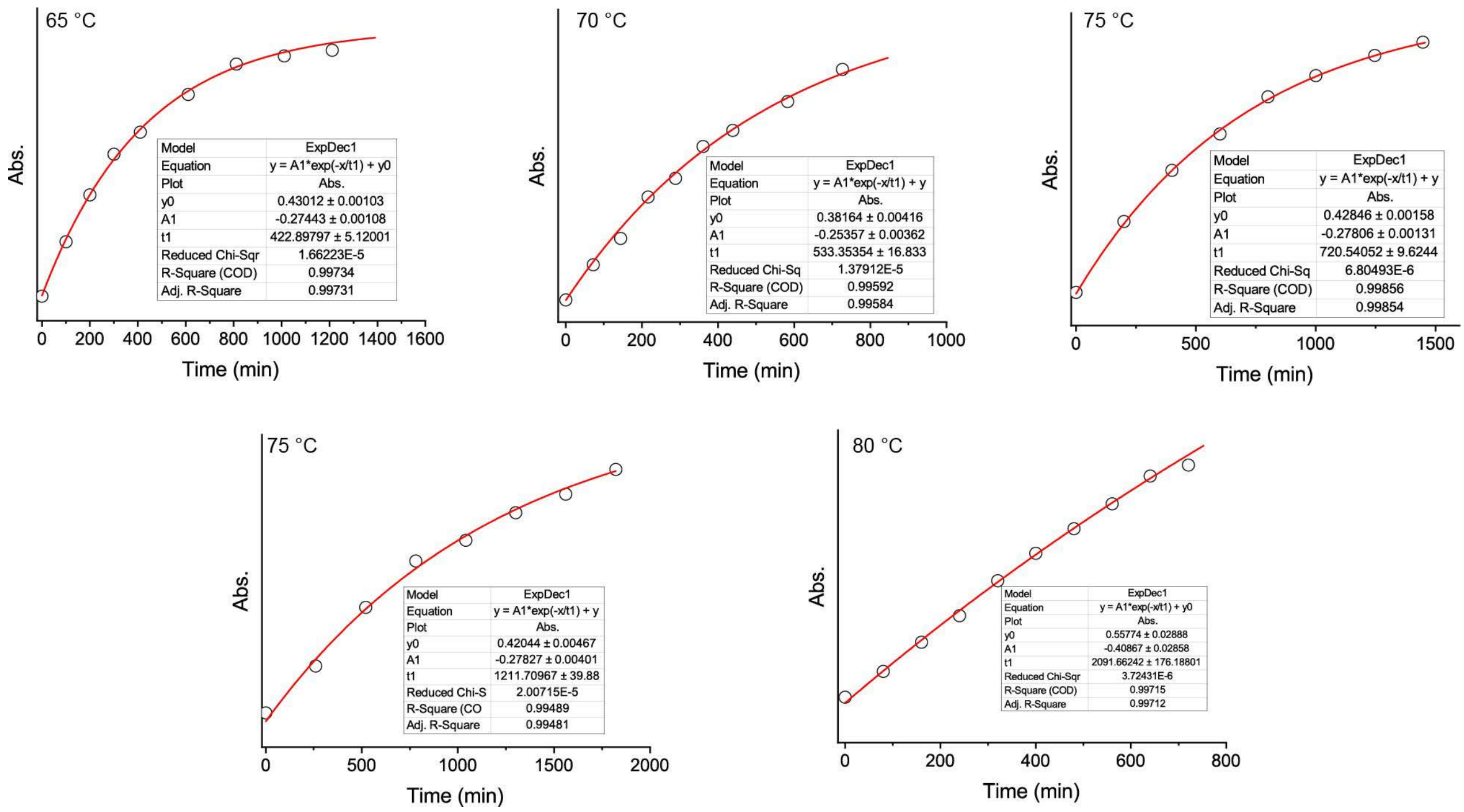

Fig. S60. Thermal back conversion of NBD6 in MeCN (0.1 mM) with NBD-to-water molar ratio of 1:5 at 65, 70, 75, 78, 80 °C. All the curves are fitted by first-order kinetics equation.

Table S15. Thermal backconversion parameters of NBD6 in MeCN (0.1 mM) with NBD-to-water molar ratio of 1:5.					
Backconversion rate	Temperature (°C)				
	65	70	75	78	80
k ($\times 10^{-3}$ min^{-1})	0.478	0.825	1.39	1.87	2.36

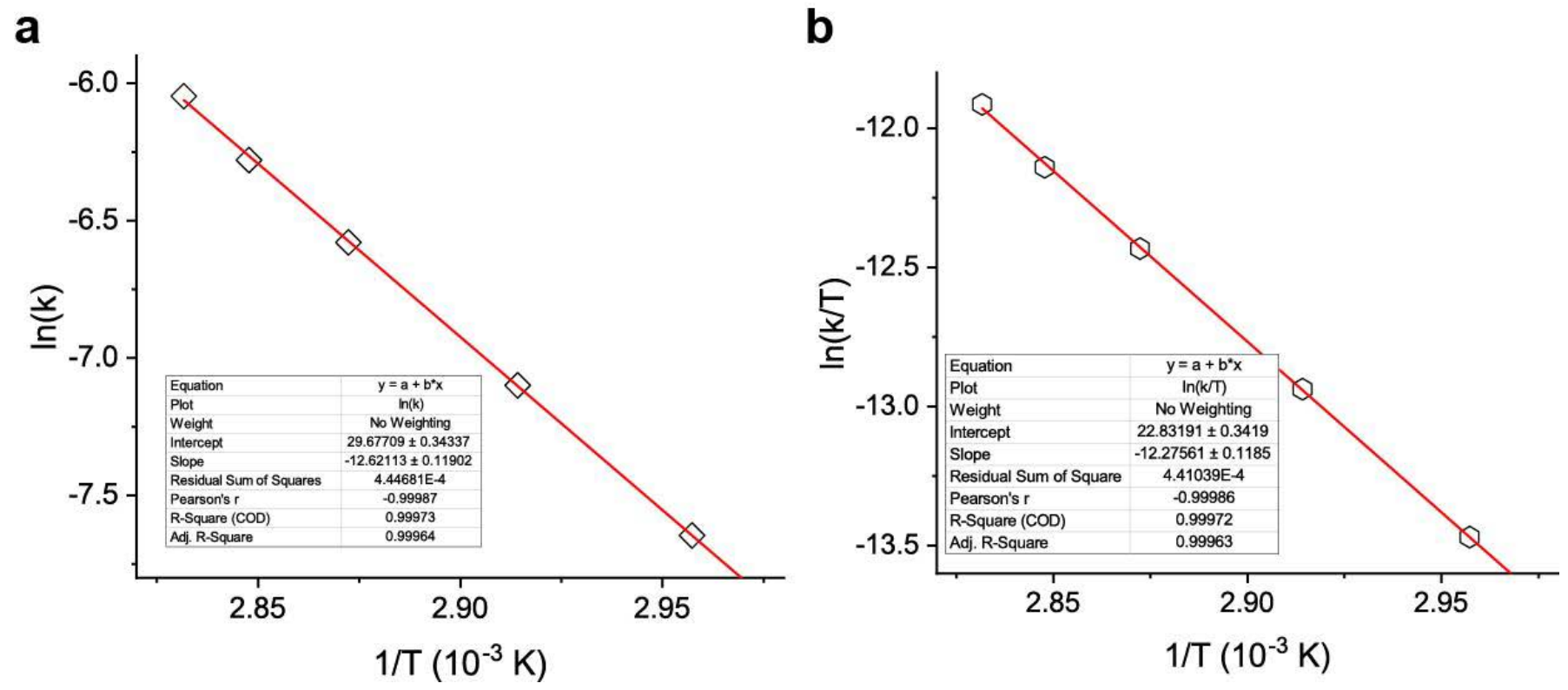

Fig. S61. **a**, Arrhenius plot of NBD6. **b**, Eyring plot of NBD6.

NBD6 in MeCN (0.1 mM) with NBD-to-water molar ratio of 1:10

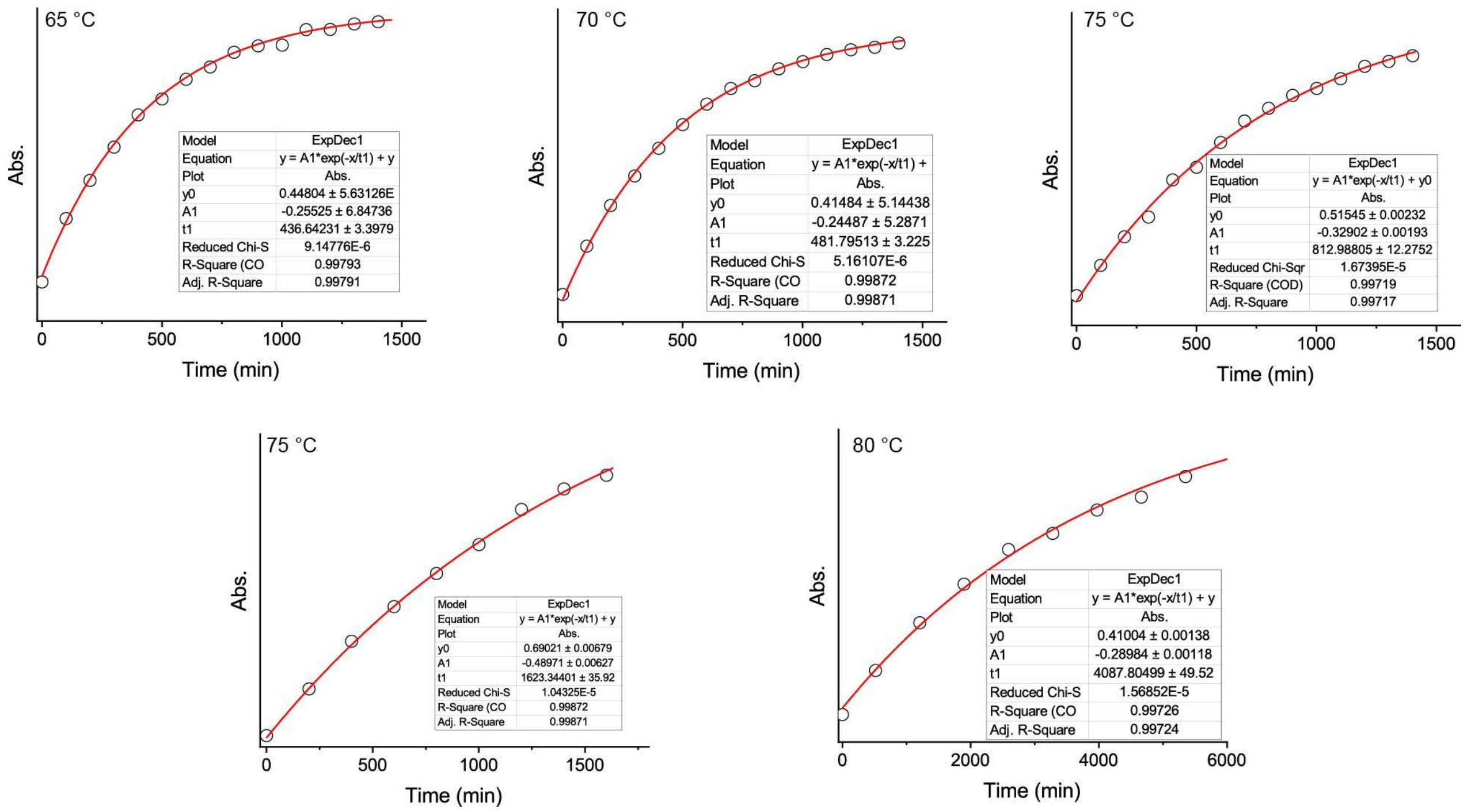

Fig. S62. Thermal back conversion of NBD6 in MeCN (0.1 mM) with NBD-to-water molar ratio of 1:10 at 65, 70, 75, 78, 80 °C. All the curves are fitted by first-order kinetics equation.

Table S16. Thermal backconversion parameters of NBD6 in MeCN (0.1 mM) with NBD-to-water molar ratio of 1:10.					
Backconversion rate	Temperature (°C)				
	65	70	75	78	80
k ($\times 10^{-3}$ min^{-1})	0.245	0.616	1.23	2.08	2.29

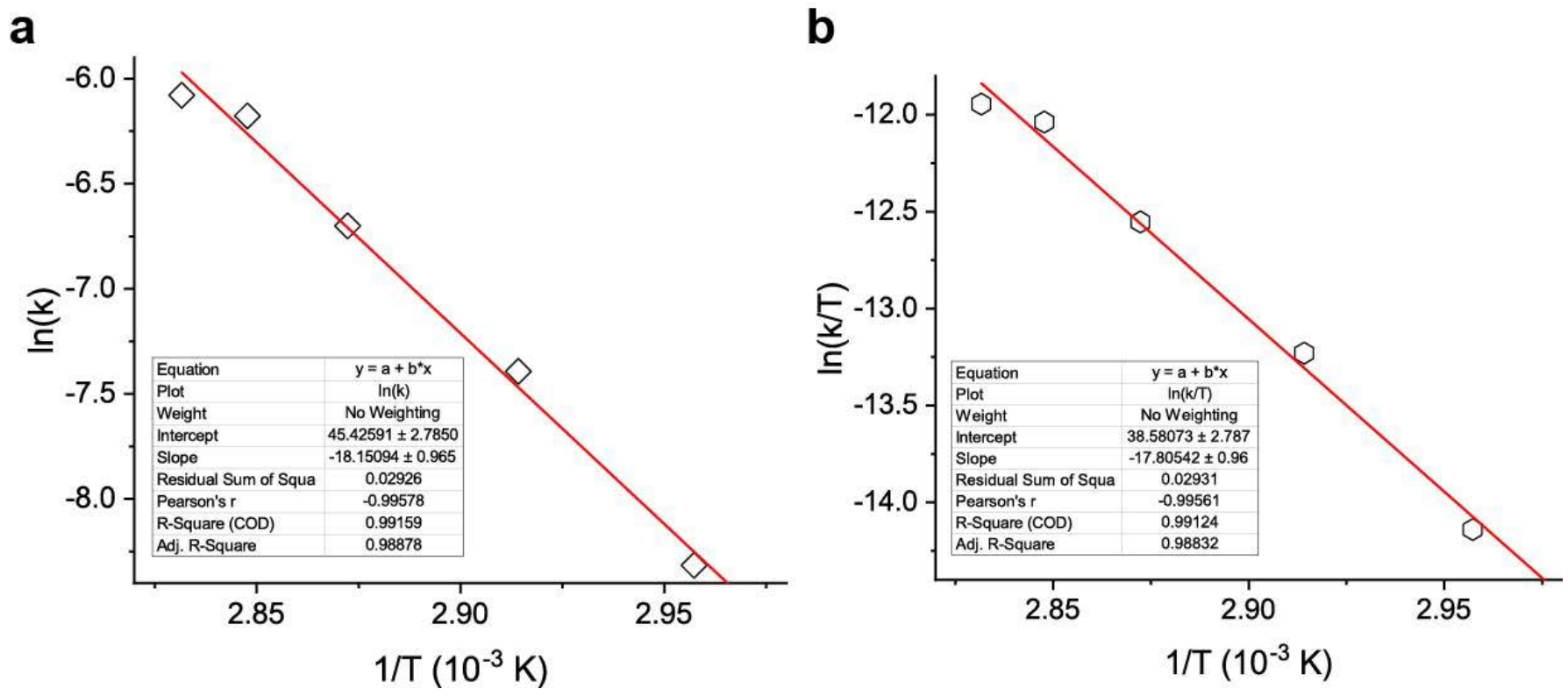

Fig. S63. **a**, Arrhenius plot of NBD6. **b**, Eyring plot of NBD6.

NBD6 in MeCN (0.1 mM) with NBD-to-water molar ratio of 1:20

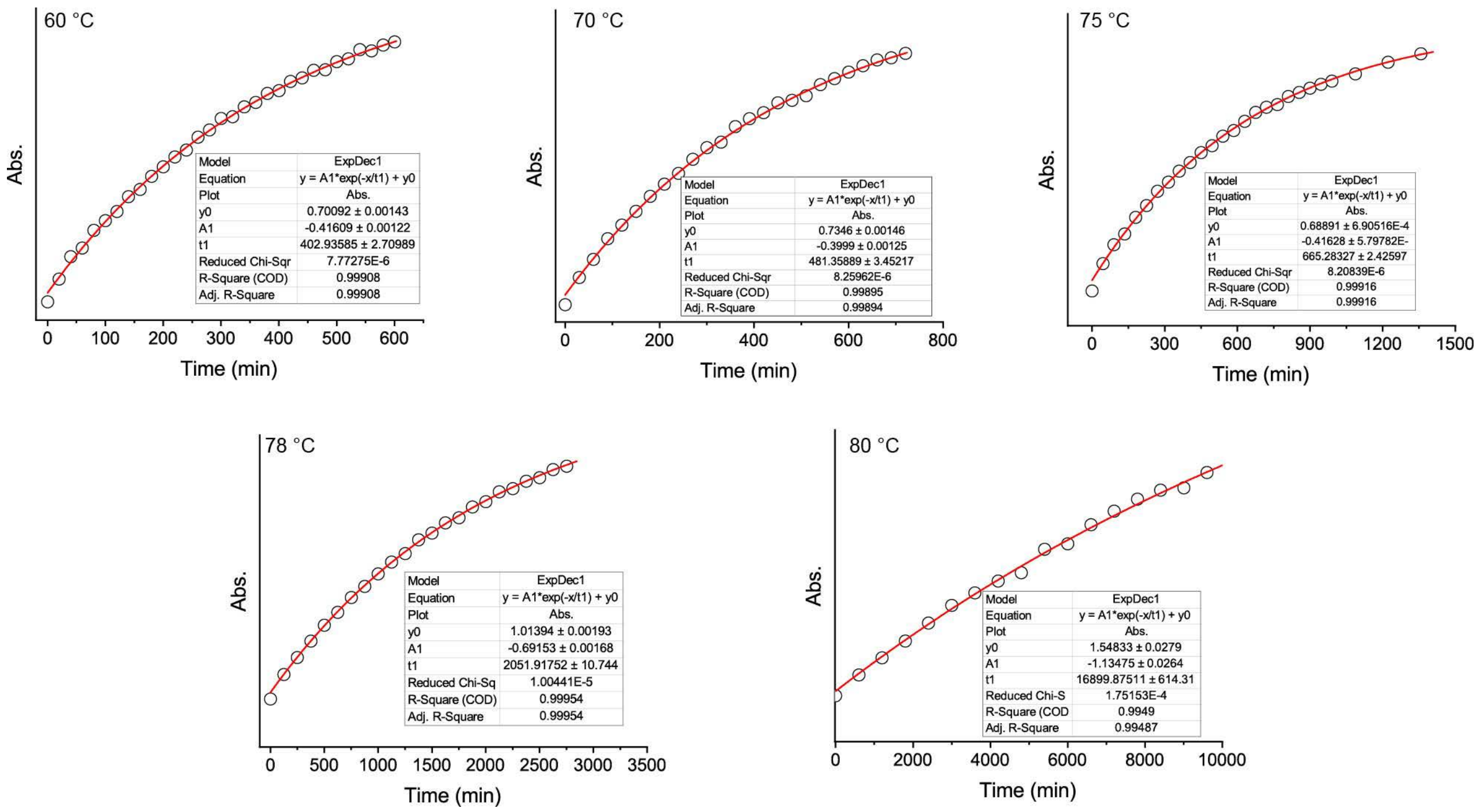

Fig. S64. Thermal back conversion of NBD6 in MeCN (0.1 mM) with NBD-to-water molar ratio of 1:20 at 60, 70, 75, 78, 80 °C. All the curves are fitted by first-order kinetics equation.

Table S17. Thermal backconversion parameters of NBD6 in MeCN (0.1 mM) with NBD-to-water molar ratio of 1:20.

Backconversion rate	Temperature (°C)				
	60	70	75	78	80
k (×10^{-3} min^{-1})	0.0592	0.487	1.50	2.08	2.48

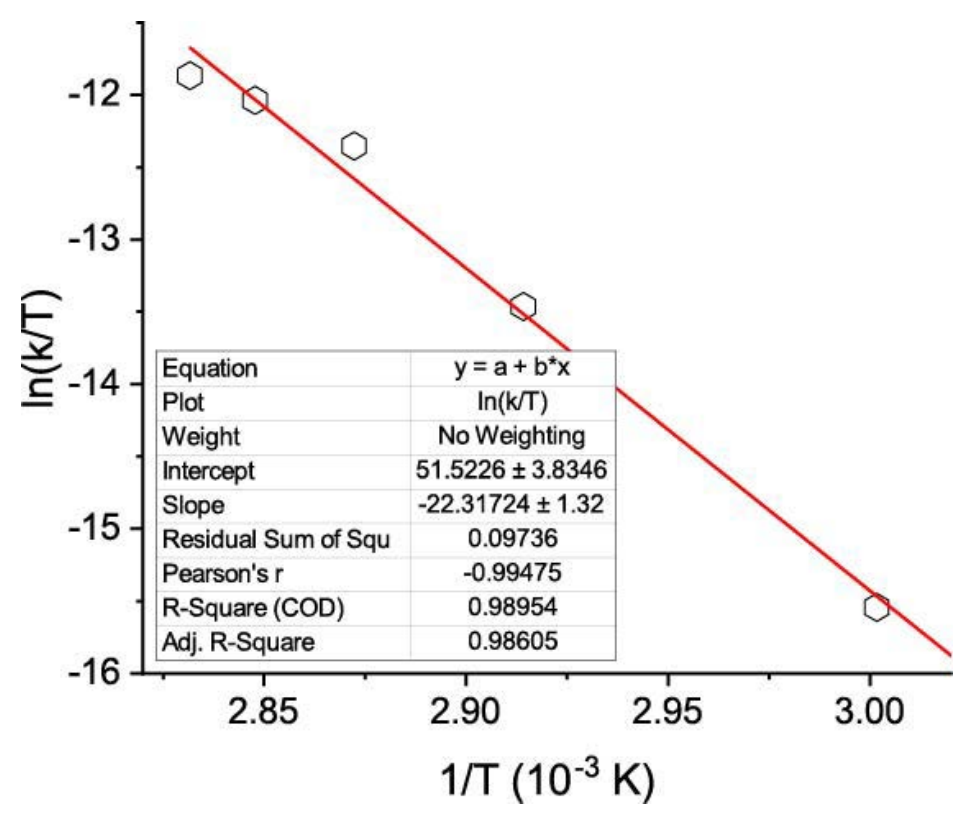

Fig. S65. Eyring plot of NBD6.

NBD6 in MeCN (0.1 mM) with NBD-to-water molar ratio of 1:200

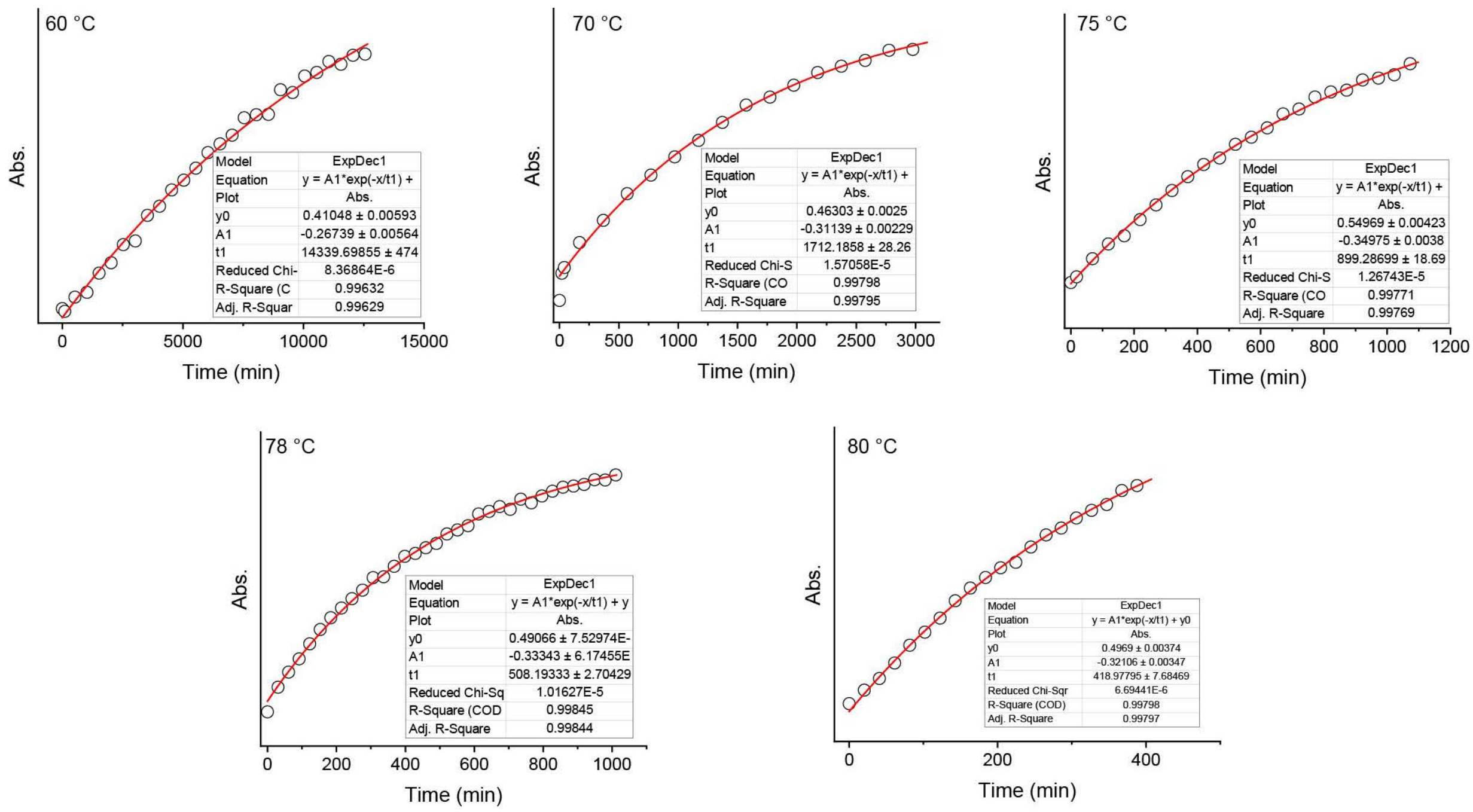

Fig. S66. Thermal back conversion of NBD6 in MeCN (0.1 mM) with NBD-to-water molar ratio of 1:200 at 60, 70, 75, 78, 80 °C. All the curves are fitted by first-order kinetics equation.

Table S18. Thermal backconversion parameters of NBD6 in MeCN (0.1 mM) with NBD-to-water molar ratio of 1:200.					
Backconversion rate	Temperature (°C)				
	60	70	75	78	80
k ($\times 10^{-3}$ min^{-1})	0.0697	0.584	1.11	1.97	2.39

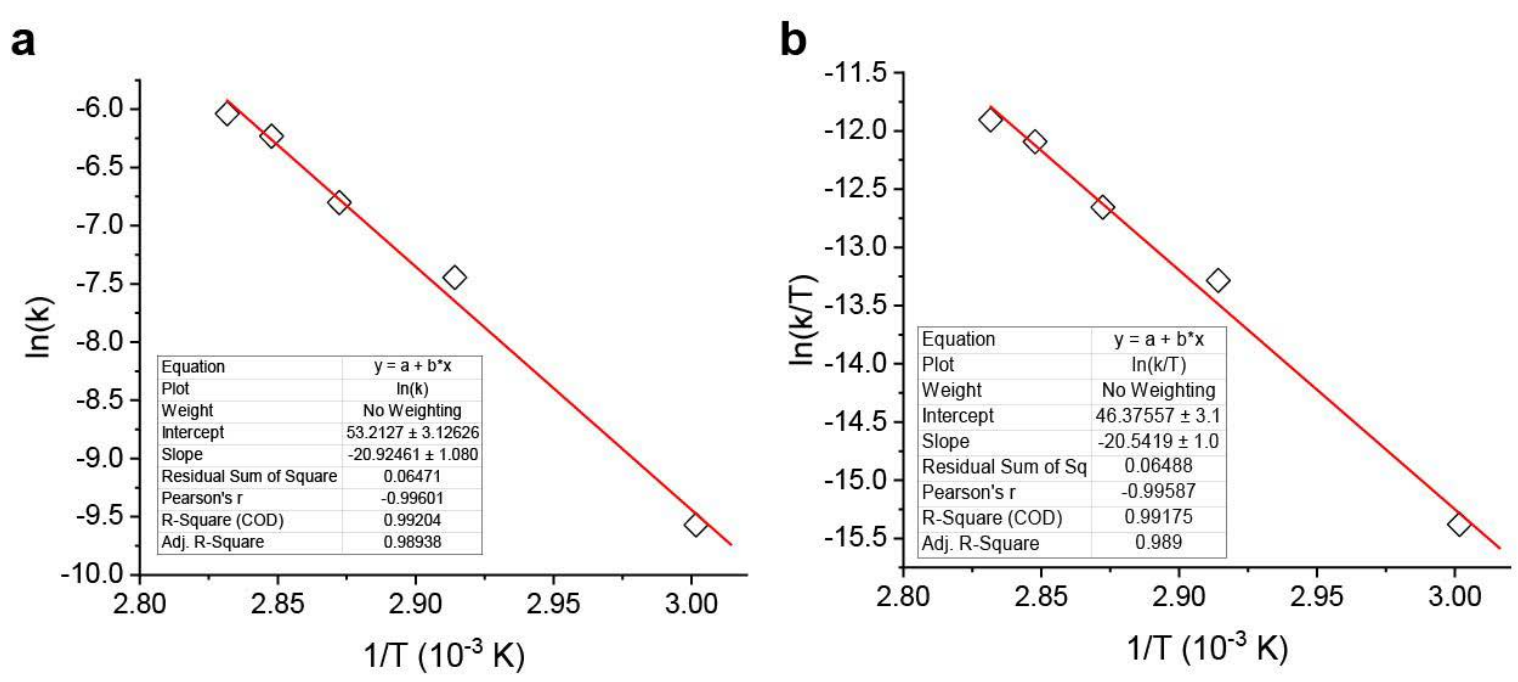

Fig. S67. **a**, Arrhenius plot of NBD6. **b**, Eyring plot of NBD6.

Table S19**.** Photoisomerization and thermal back isomerization properties of the NBD/QC pairs.

	λ_{max}[a] (nm)	λ_{onset}[b] (nm)	NBD to QC[c] (%)	Molar ratios of water:NBD	$t_{1/2}$[d] (d)	$\Delta H^{‡}$ [e] (kJ mol^{-1})	$\Delta S^{‡}$ [e] (J mol^{-1} K^{-1})	$\Delta G^{‡}$ [e] (kJ mol^{-1})
NBD1	307	372	99	No water	26	119.6	62.9	100.9
				20:1	32	122.9	71.9	101.5
NBD2	325	382	99	No water	58	109.5	22.6	102.7
				20:1	62	110.9	26.7	102.9
NBD3	330	400	98	No water	4.0	89.5	-18.7	95.1
				5:1	30	112.6	36.8	101.6
				20:1	39	114.7	43.2	101.8
NBD4	<300	373	95	No water	92	114.9	36.6	104.0
				5:1	279	117.5	36.2	106.7
				20:1	592	113.9	20.1	107.9
NBD5	316	390	98	No water	48	117.6	51.0	102.4
				5:1	140	94.4	-35.25	105.0
				20:1	462	115.7	26.2	107.9
NBD6	<300	397	99	No water	1.6	85.9	-27.4	94.1
				5:1	108	102.1	-7.73	104.4
				10:1	1790	148.0	123.2	111.3
				20:1	8762	185.5	230.8	116.7

[a] The λ_{max} were taken from UV/Vis absorption spectra measured in MeCN (0.1 mM).
[b] The λ_{onset} is defined as the wavelength where log ε = 2.
[c] The photoconversion yields from NBD to QC was obtained through integrating the peak areas of ^{1}H NMR spectra (Supplementary Information, Section 3.1).
[d] $t_{1/2}$ values are at 25 °C, which were calculated through Arrhenius plot based on the experimental kinetics data (Supplementary Information, Section 3.2).
[e] Those values were calculated through Eyring plot based on the experimental kinetics data (Supplementary Information, Section 3.2).

Table 1**.** Photoisomerization and thermal back isomerization properties of the NBD/QC pairs.

	λ_{max}[a] (nm)	λ_{onset}[b] (nm)	NBD to QC[c] (%)	Molar ratios of water:NBD	$t_{1/2}$[d] (d)	$\Delta H^{‡}$ [e] (kJ mol^{-1})
NBD6	<300	397	99	No water	1.6	85.9
				5:1	108	102.1
				10:1	1790	148.0
				20:1	8762	185.5

3.2.2 Selectively control thermal backconversion

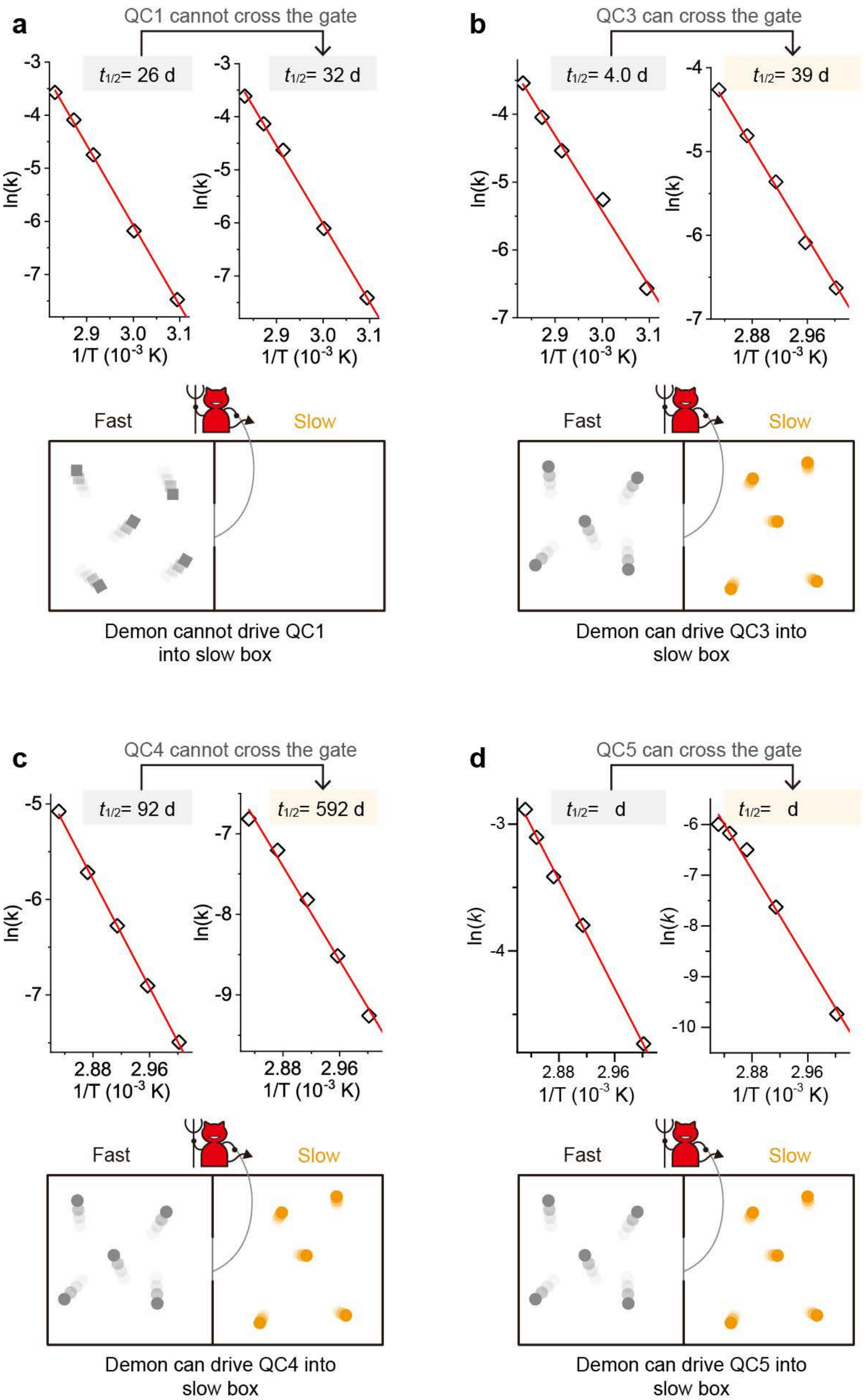

Fig. S68. Arrhenius plots of **a** QC1, **b** QC3, **c** QC4 and **d** QC5 in MeCN without water (left) and with water at a molar ratio of NBD:water = 1:20 (right). Cartoon of Maxwell's demon system illustrates that QC1 is unable to pass through the gate, whereas QC3-5 can.

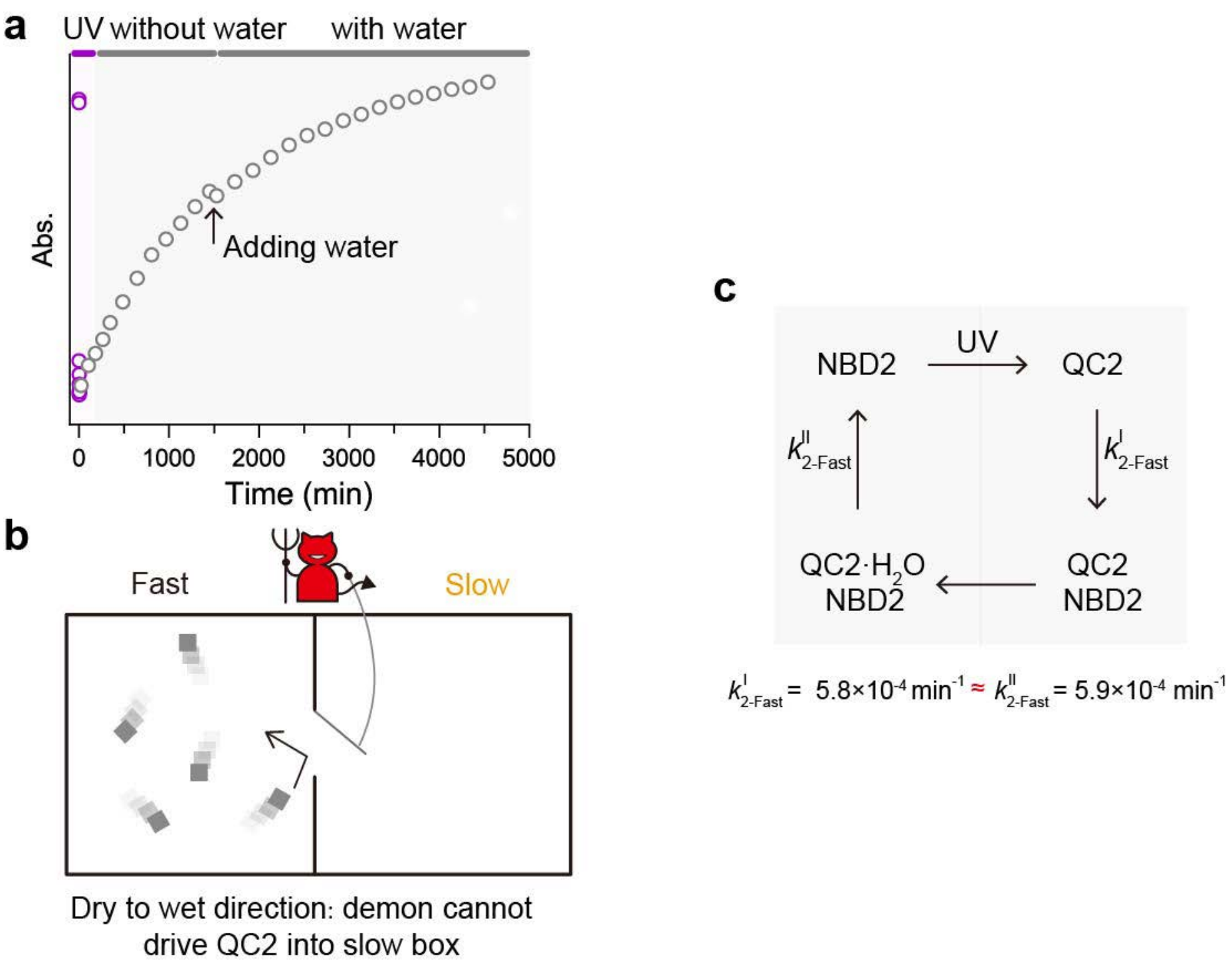

Fig. S69. **a**, Backcoversion plots of QC2 at 60 °C in MeCN from dry to wet. **b**, Cartoon of Maxwell's demon system represents the directional gate-opening control. **c**, Kinetic model of h dry to wet direction of QC2 solution

To further verify the selective regulation of backconversion dynamics in this system, two-way experiments of **QC2** between the Fast Box and Slow Box were performed. In the initial state, when the **QC2** solution contained no water, approximately 60% of **QC2** converted into **NBD6** within 1550 min, with a $k^{I}_{2\text{-Fast}}$ value of 5.8 $\times 10^{-4}$ min^{-1}. Upon the addition of water, there is nearly no change of the backconversion rate of **QC2**, with the $k^{II}_{6\text{-Slow}}$ value reducing to 5.9 $\times 10^{-4}$. In this scenario, the demon drives cannot transport from the Fast Box to the Slow Box.

3.2.4 Thermal back conversion of NBD6/QC6 pair in various solvents

NBD6 in anhydrous toluene (0.1 mM)

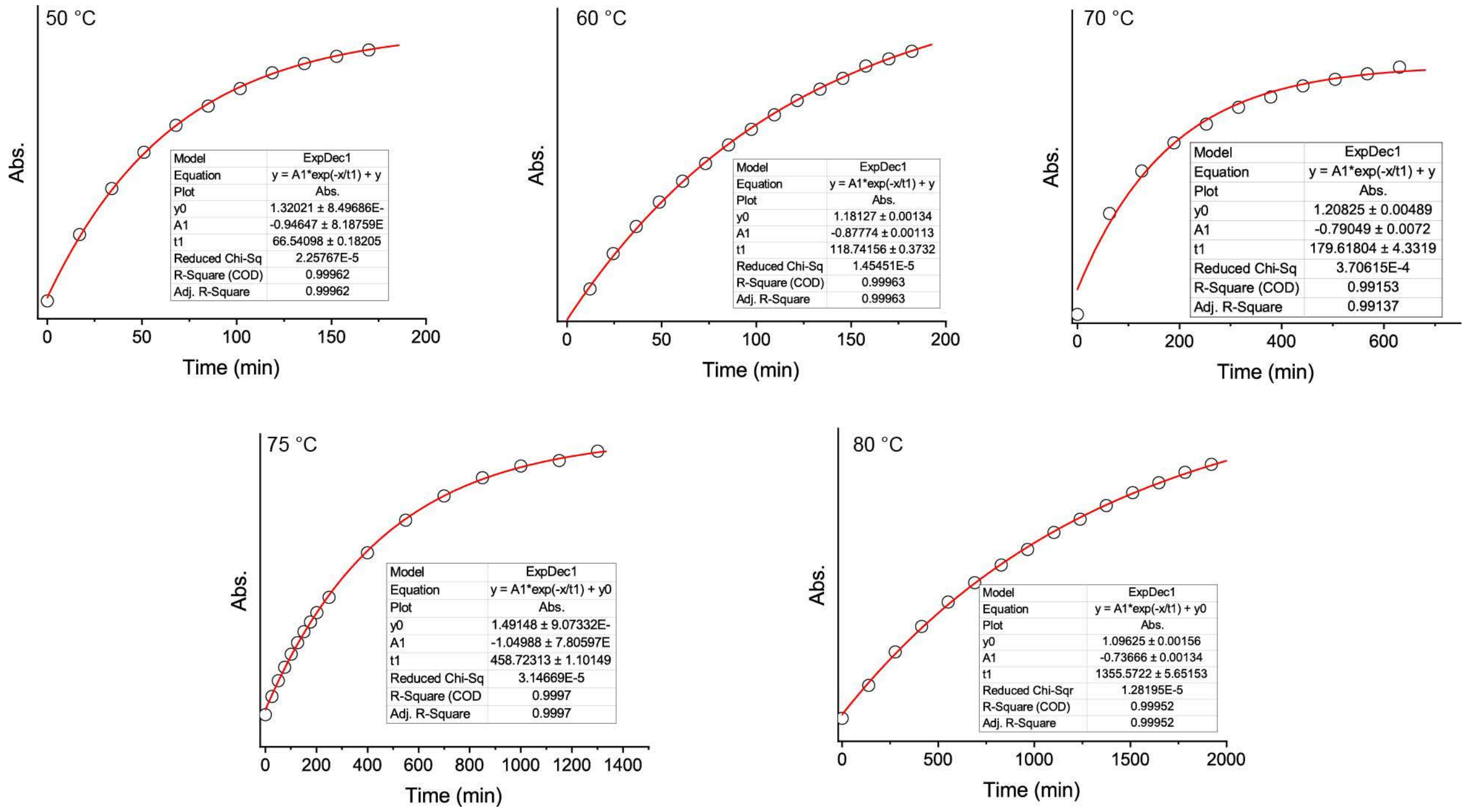

Fig. S70. Thermal back conversion of NBD6 in anhydrous toluene (0.1 mM) at 50, 60, 70, 75, 80 °C. All the curves are fitted by first-order kinetics equation.

Table S20. Thermal backconversion parameters of NBD6 in anhydrous toluene (0.1 mM).					
Backconversion rate	Temperature (°C)				
	50	60	70	75	80
k ($\times 10^{-3}$ min^{-1})	0.738	2.18	5.57	8.42	15.0

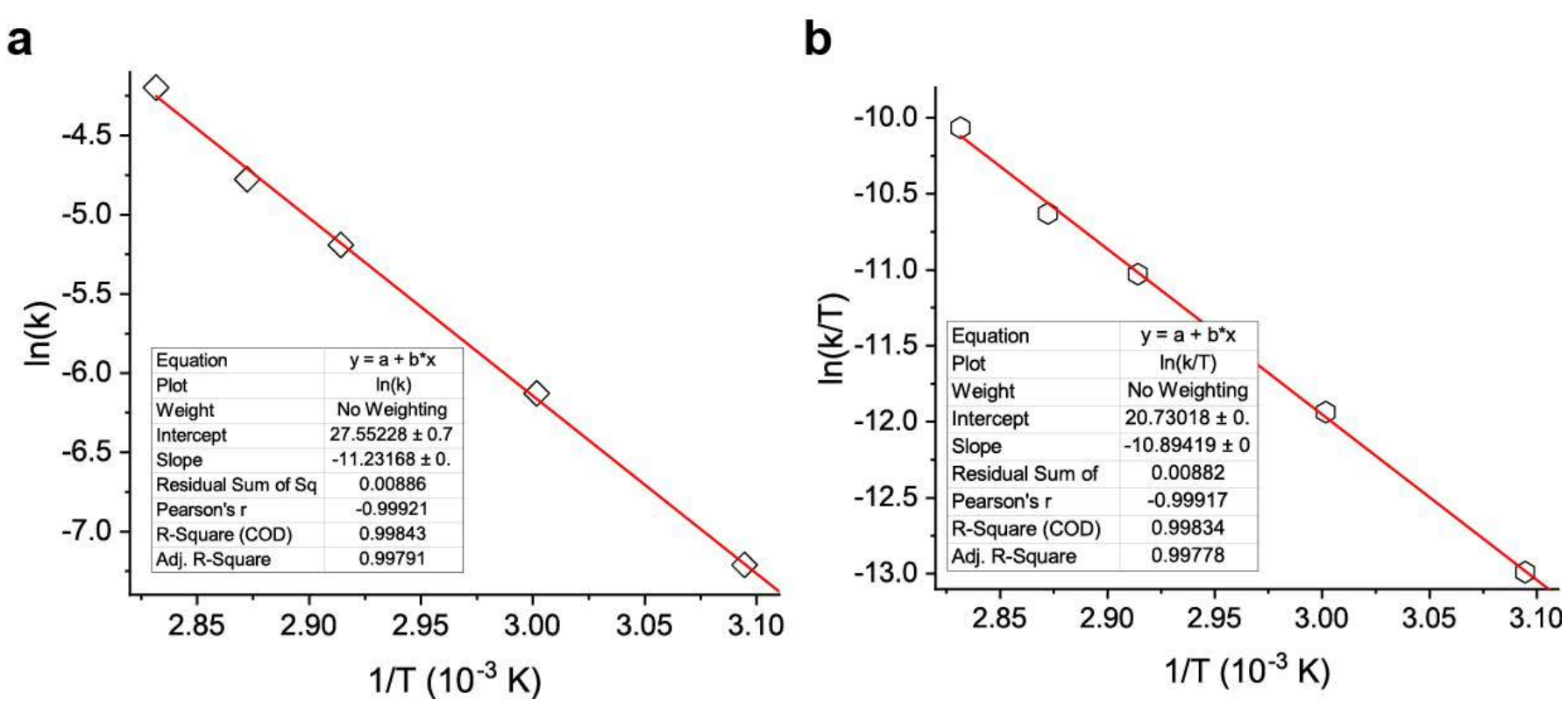

Fig. S71. **a**, Arrhenius plot of NBD6. **b**, Eyring plot of NBD6 in toluene.

NBD6 in toluene (0.1 mM) with NBD-to-water molar ratio of 1:20

(*The ratio of 1:20 indicates the amount of water added, rather than the actual content present in toluene.*)

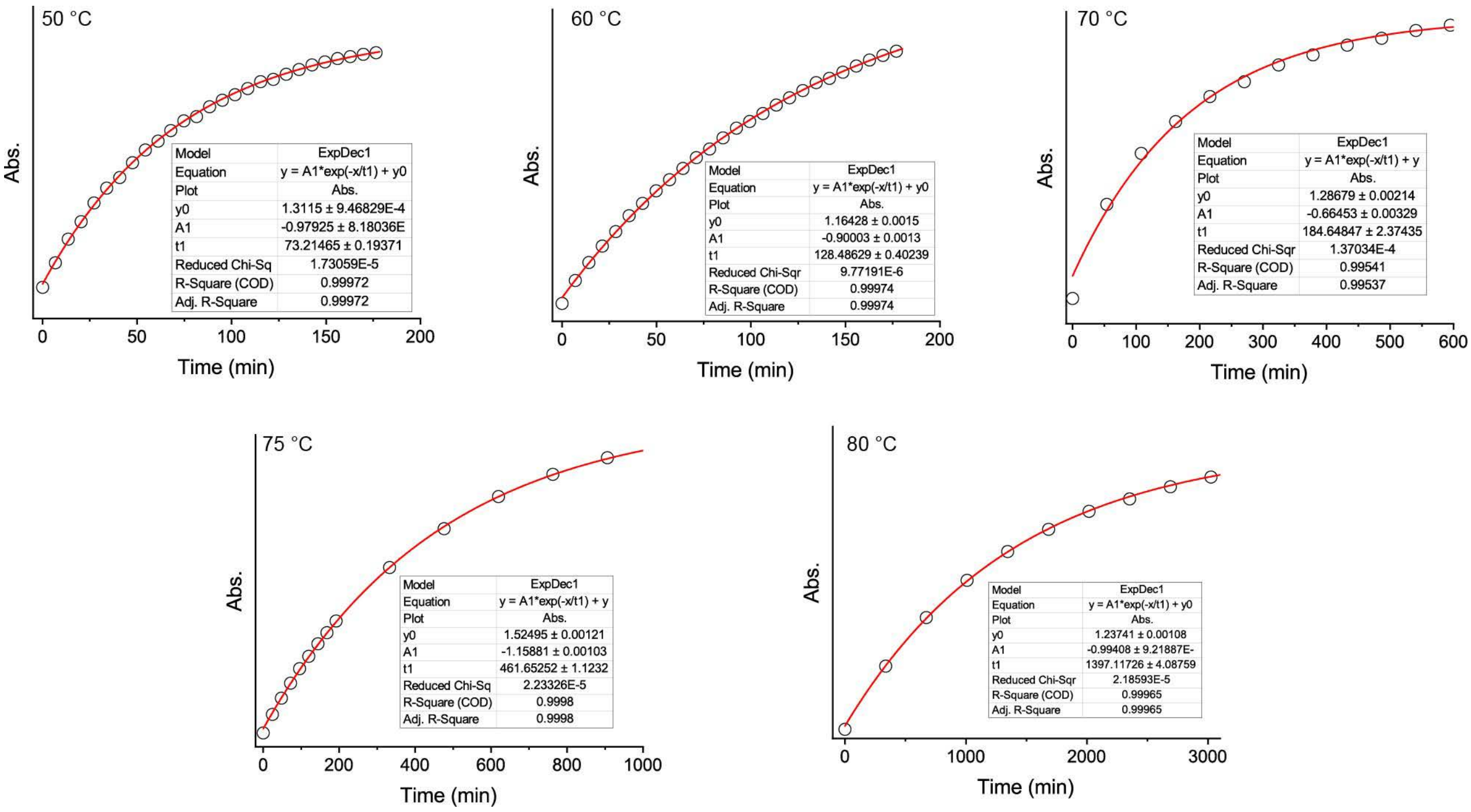

Fig. S72. Thermal back conversion of NBD6 in toluene (0.1 mM) with NBD-to-water molar ratio of 1:20 at 50, 60, 70, 75, 80 °C. All the curves are fitted by first-order kinetics equation.

Table S21. Thermal backconversion parameters of NBD6 in toluene (0.1 mM) with NBD-to-water molar ratio of 1:20.					
Backconversion rate	Temperature (°C)				
	50	60	70	75	80
k ($\times 10^{-3}$ min^{-1})	0.716	2.17	5.42	7.78	13.7

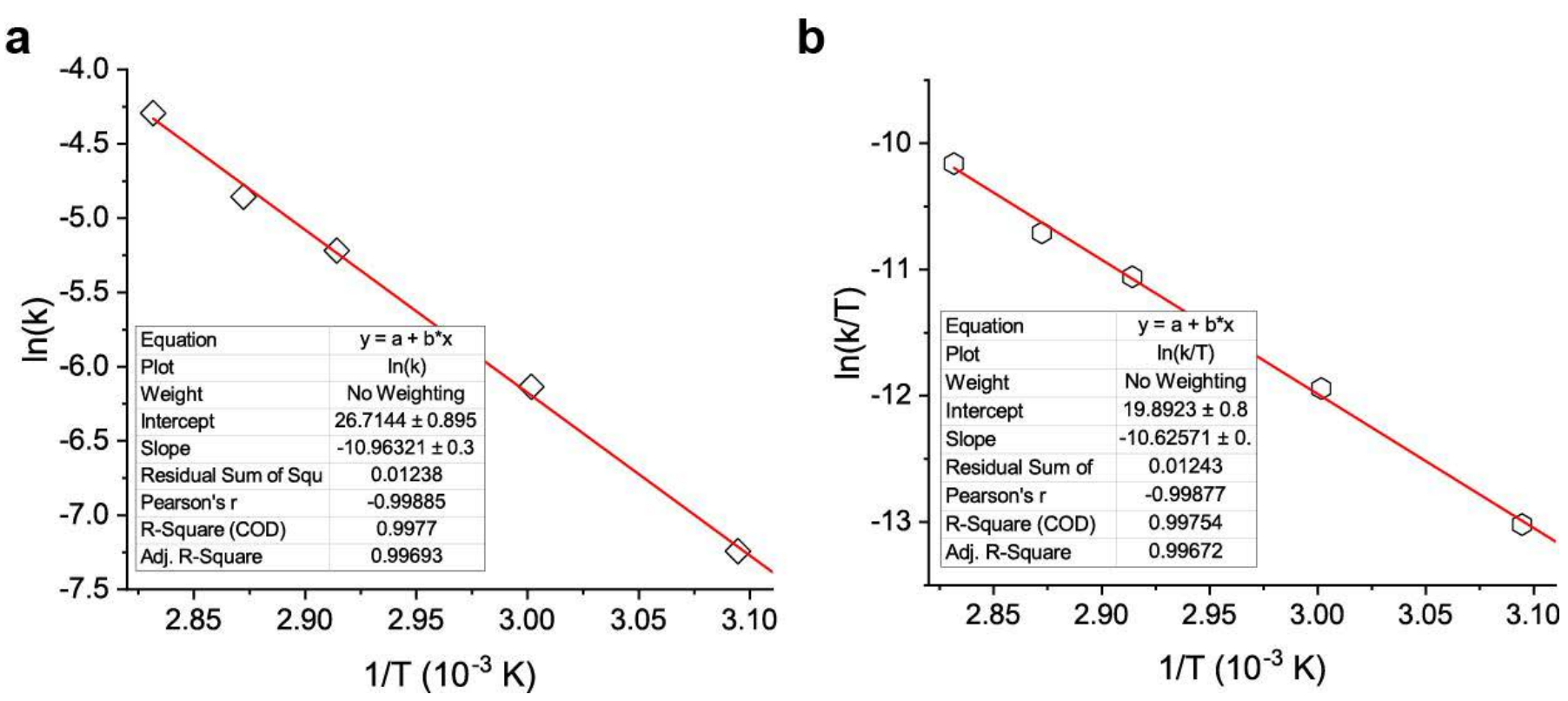

Fig. S73. **a**, Arrhenius plot of NBD6. **b**, Eyring plot of NBD6 in toluene.

NBD6 in anhydrous 1,4-dioxane (0.1 mM)

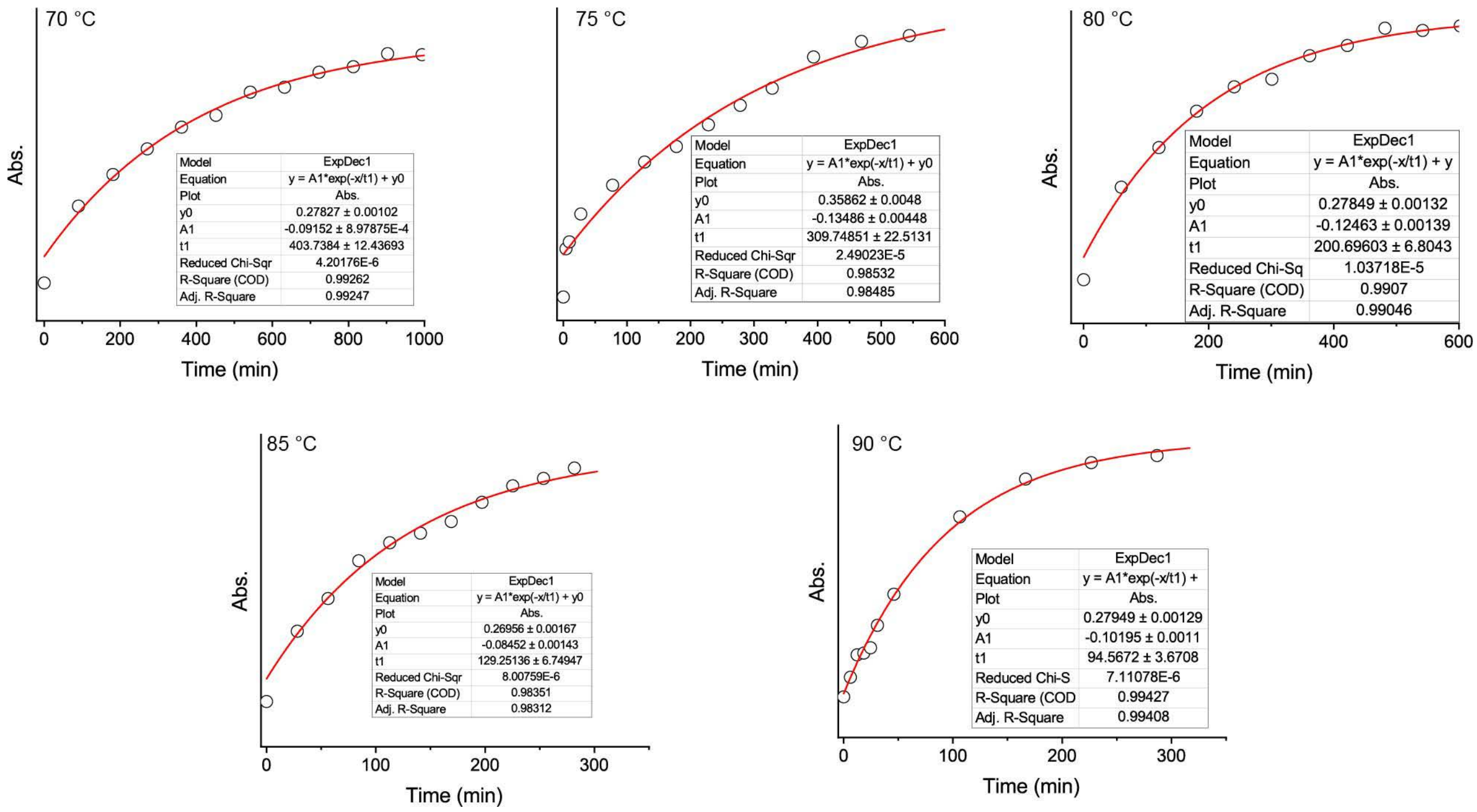

Fig. S74. Thermal back conversion of NBD6 in anhydrous 1,4-dioxane (0.1 mM) at 70, 75, 80, 85, 90 °C. All the curves are fitted by first-order kinetics equation.

Table S22. Thermal backconversion parameters of NBD6 in anhydrous 1,4-dioxane (0.1 mM).					
Backconversion rate	Temperature (°C)				
	70	75	80	85	90
k (×10^{-3} min^{-1})	2.48	3.23	4.98	7.74	10.6

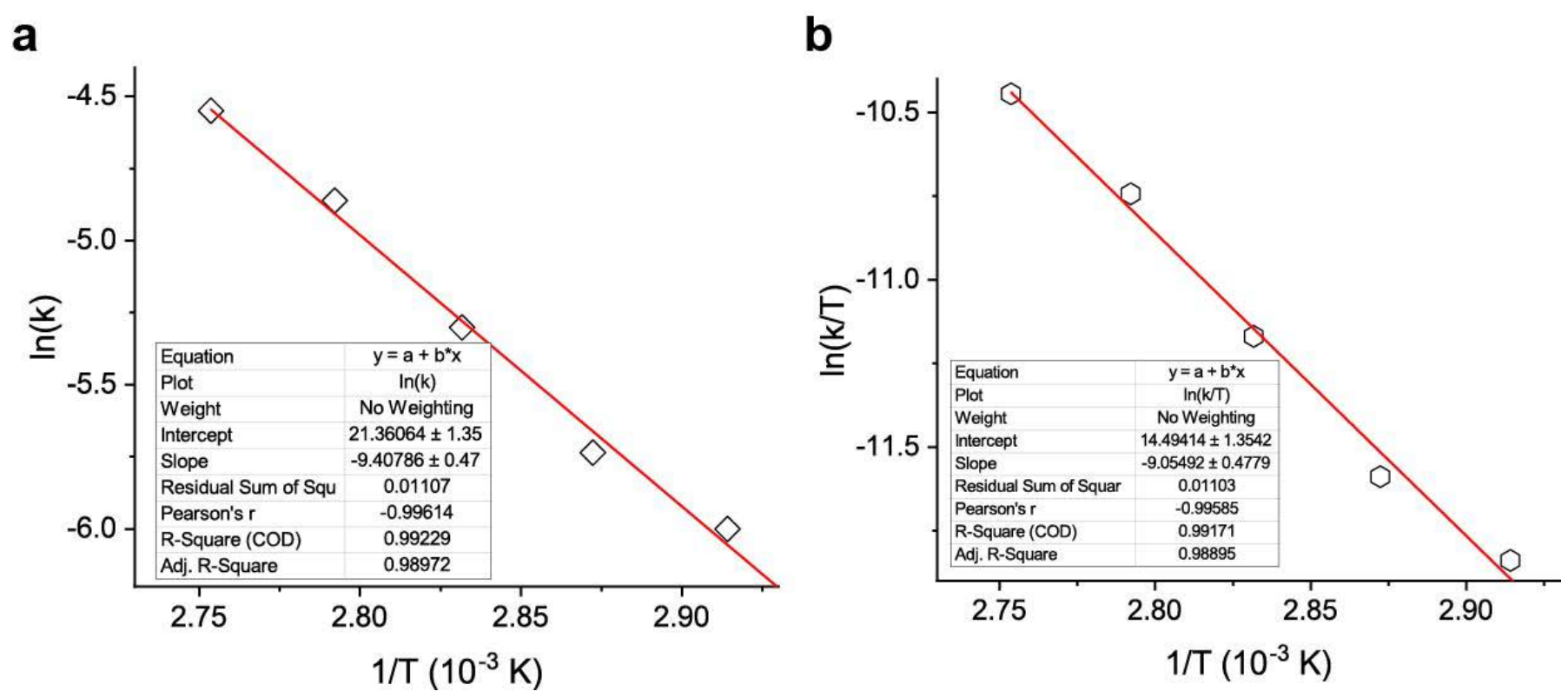

Fig. S75. **a**, Arrhenius plot of NBD6. **b**, Eyring plot of NBD6 in 1,4-dioxane.

NBD6 in 1,4-dioxane (0.1 mM) with NBD-to-water molar ratio of 1:20

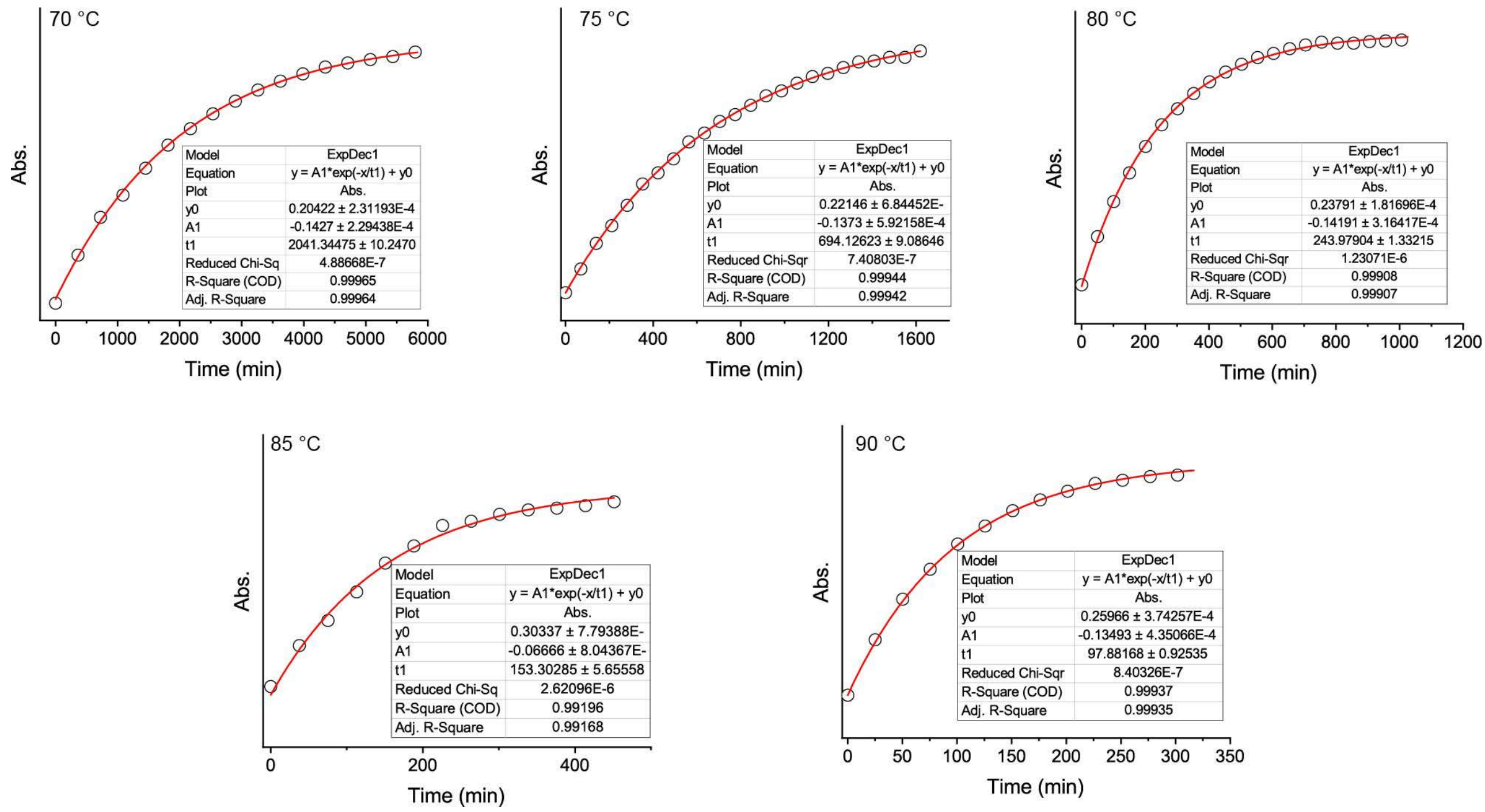

Fig. S76. Thermal back conversion of NBD6 in 1,4-dioxane (0.1 mM) with NBD-to-water molar ratio of 1:20 at 70, 75, 80, 85, 90 °C. All the curves are fitted by first-order kinetics equation.

Table S23. Thermal backconversion parameters of NBD6 in 1,4-dioxane (0.1 mM) with NBD-to-water molar ratio of 1:20.					
Backconversion rate	Temperature (°C)				
	70	75	80	85	90
k ($\times 10^{-3}$ min^{-1})	0.490	1.44	4.10	6.52	10.2

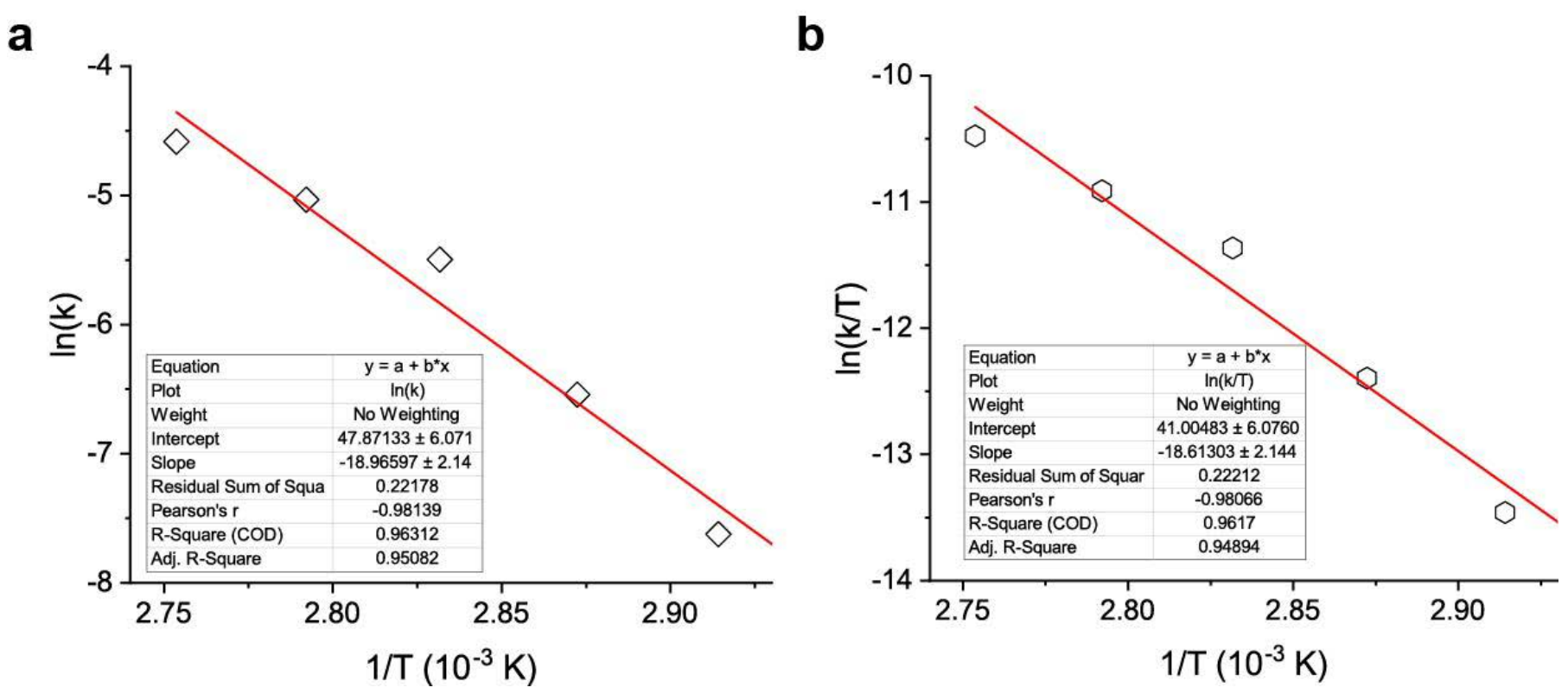

Fig. S77. **a**, Arrhenius plot of NBD6. **b**, Eyring plot of NBD6 in 1,4-dioxane.

NBD6 in anhydrous isopropanol (0.1 mM)

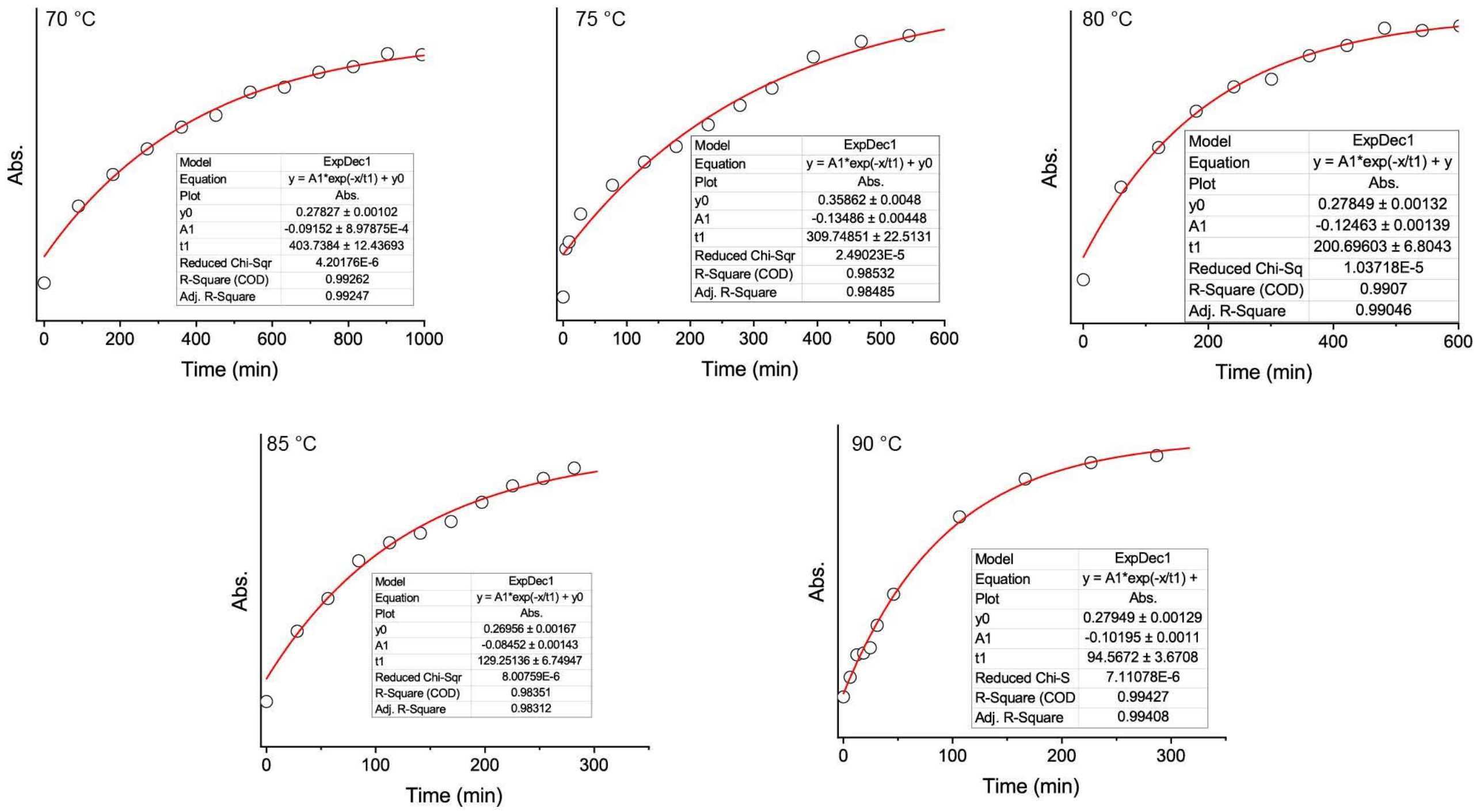

Fig. S78. Thermal back conversion of NBD6 in anhydrous isopropanol (0.1 mM) at 60, 70, 75, 78, 80 °C. All the curves are fitted by first-order kinetics equation.

Table S24. Thermal backconversion parameters of NBD6 in anhydrous isopropanol (0.1 mM).					
Backconversion rate	Temperature (°C)				
	60	70	75	78	80
k ($\times 10^{-3}$ min^{-1})	0.337	2.07	2.92	4.10	4.87

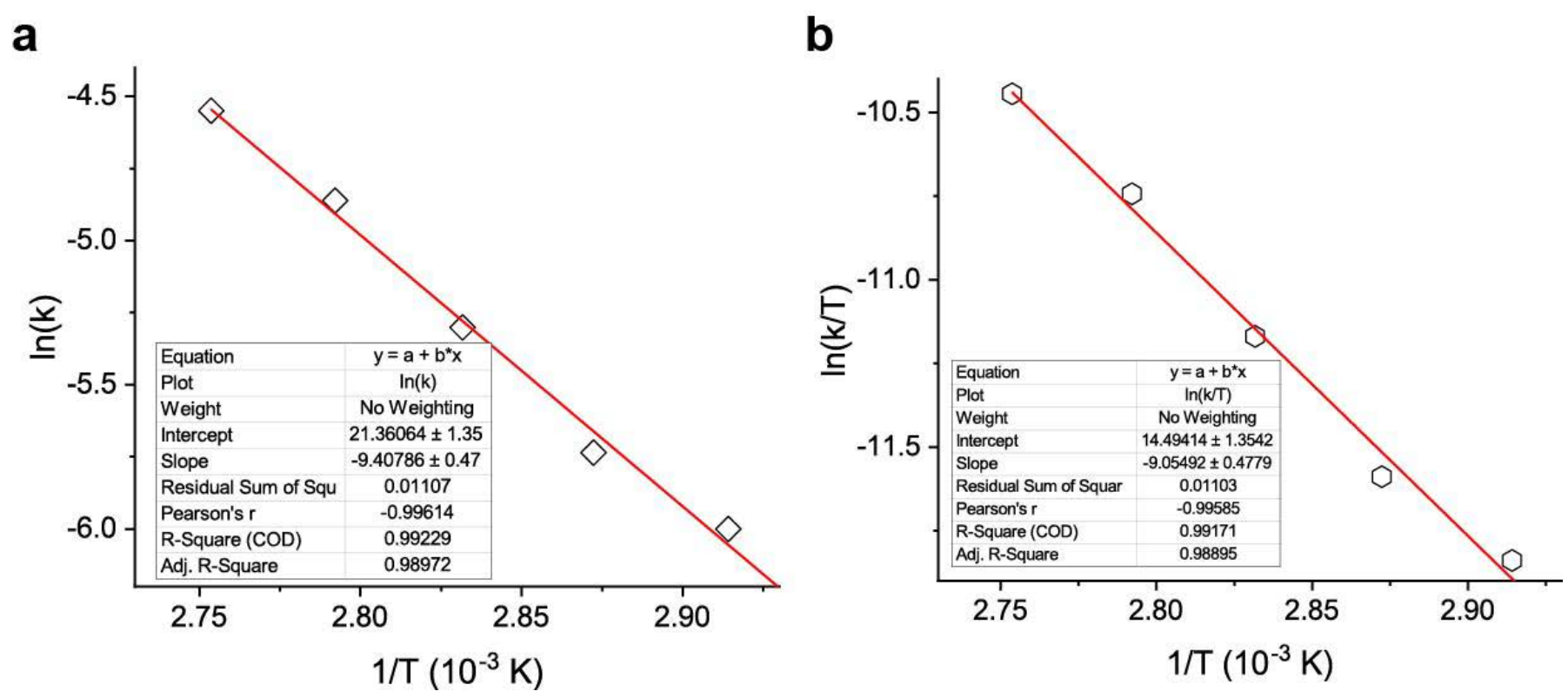

Fig. S79. **a**, Arrhenius plot of NBD6. **b**, Eyring plot of NBD6 in isopropanol.

NBD6 in isopropanol (0.1 mM) with NBD-to-water molar ratio of 1:20

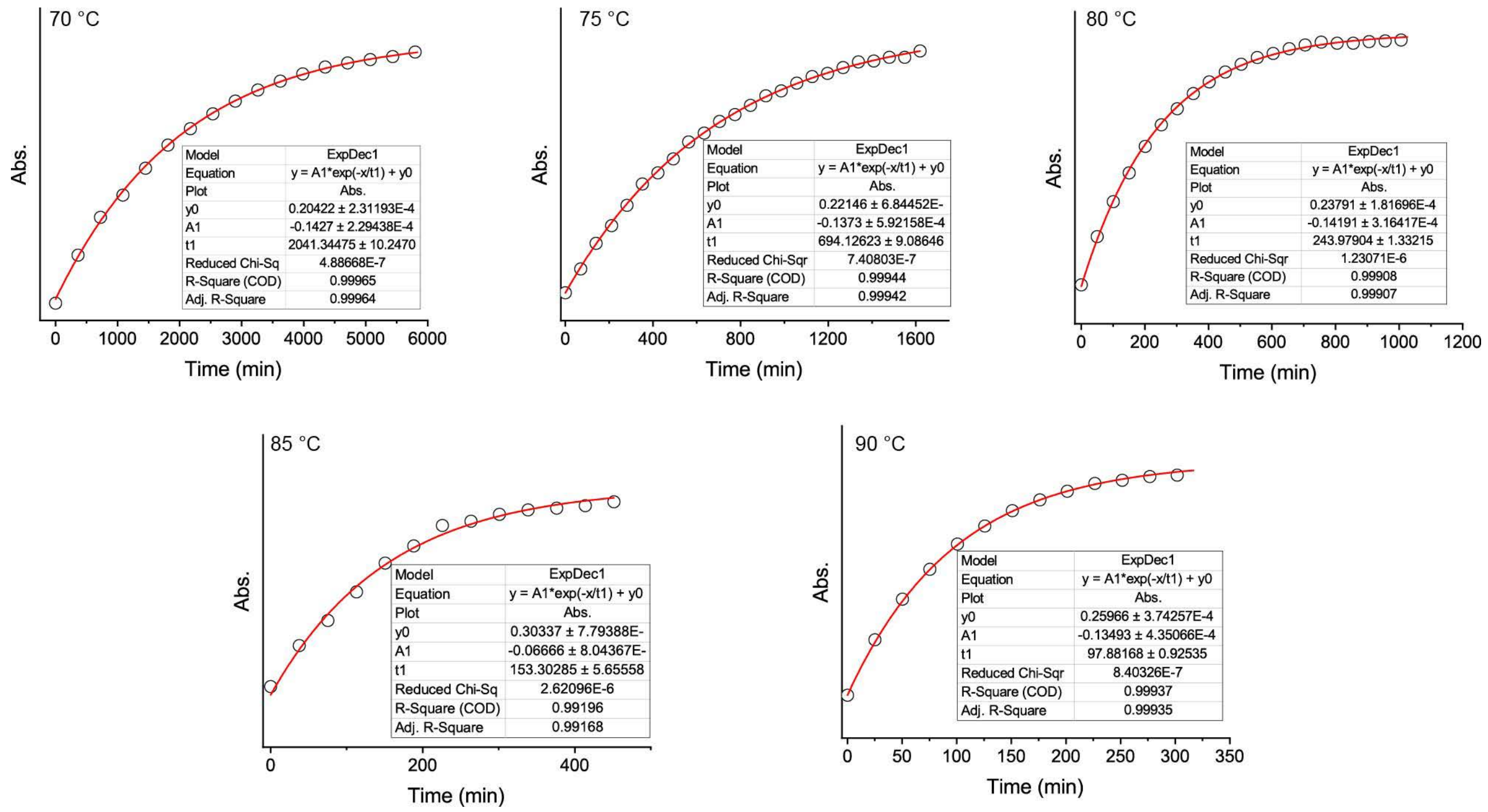

Fig. S80. Thermal back conversion of NBD6 in isopropanol (0.1 mM) with NBD-to-water molar ratio of 1:20 at 60, 70, 75, 78, 80 °C. All the curves are fitted by first-order kinetics equation.

Table S25. Thermal backconversion parameters of NBD6 in isopropanol (0.1 mM) with NBD-to-water molar ratio of 1:20.					
Backconversion rate	Temperature (°C)				
	60	70	75	78	80
k ($\times 10^{-3}$ min^{-1})	0.211	1.16	2.23	2.69	4.16

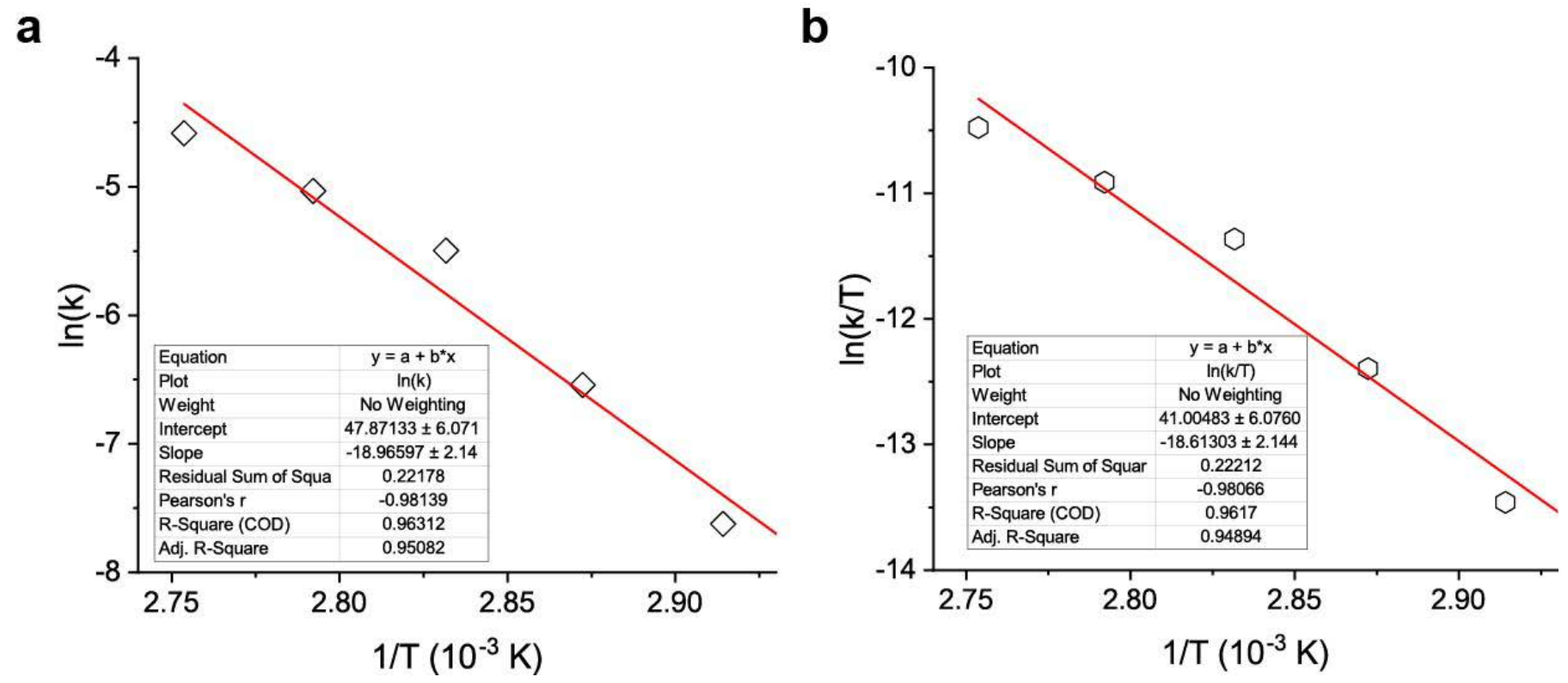

Fig. S81. **a**, Arrhenius plot of NBD6. **b**, Eyring plot of NBD6 in isopropanol.

Table S26**.** Thermal back isomerization properties of the NBD6/QC6 pair in various solvents.

Solvents [a]	Molar ratios of water:NBD	$t_{1/2}$ [b] (d)	$\Delta H^{‡}$ [c] (kJ mol^{-1})	$\Delta S^{‡}$ [c] (J mol^{-1} K^{-1})	$\Delta G^{‡}$ [d] (kJ mol^{-1})
Toluene	No water	8.6	90.5	-25.2	98.1
	20:1	8.0	88.4	-32.1	98.0
1,4-Dioxane	No water	9.3	97.3	-22.5	104.0
	20:1	2337	154.7	143.4	112.0
Isopropanol	No water	209	126.6	68.8	103.0
	20:1	633	139.1	102.1	103.0

[a] Concentration is 0.1 mM for all solvents.

[b] $t_{1/2}$ values are at 25 °C, which were calculated through Arrhenius plot based on the experimental kinetics data (Supplementary Information, Section 3.3).

[c] Those values were calculated through Eyring plot based on the experimental kinetics data (Supplementary Information, Section 3.3).

Table S27. Comparison of thermal half-life control in photoswitches achieved through solvent effects in literatures and the hydrogen-bonding strategy employed in this study.

Photoswitches	Minimum half-life [a]		Maximum half-life [b]		Changes [c]	Ref.
	Value	Solvent used	Value	Solvent used		
Norbornadiene derivatives with an asymmetric 1,2 substitution	17 h	Hexane	29 h	MeCN	Hour-scale regulation (~2 times)	([25])
	4.1 h	Hexane	8.8 h	MeCN		
Azobenzazoles	0.4 s	MeCN	84 s	Toluene	Second-scale regulation (~210 times)	([26])
Nitrogen Bridged Diazocines	27 min	Acetone	73 min	Water	Minute-scale regulation (~3 times)	([27])
Diazocine-Based Estrogen Receptor Agonists	17.2 h	DMSO	42.4 h	H2O/D2O	From seconds to minutes (~40 times)	([28])
	6.6 h	DMSO	40.7 h	H2O/D2O		
	3.6 s	DMSO	2.4 min	H2O/D2O		
	1.5 s	DMSO	19.9 s	H2O/D2O		
	4.0 h	DMSO	15.2 h	H2O/D2O		
	11.2 h	DMSO	26.1 h	H2O/D2O		
	11.4 h	DMSO	31.7 h	H2O/D2O		
Azothiophene-based molecular switches	8.6 s	Methanol	4.0 min	Isopropanol	From seconds to minutes (~28 times)	([29])
mexylaminotriazine-substituted merocyanine	30 s	DMF	6.8 min	n-Propanol	From seconds to minutes (~177 times)	([30])
	8.2 s	Toluene	24.2 min	Ethanol		
	19 s	THF	25.3 min	Ethanol		
Donor-acceptor Stenhouse adducts	21.7 s	Toluene	28.6 s	Chloroform	Second-scale regulation (~1.3 times)	([31])
Z-Tetra-ortho-fluoroazobenzene	14 h	DMSO-*d6*	48 h	Toluene-*d8*	Hour- or day- scale regulation (~4 times)	([32])
	193 d	Methanol-*d4*	503 d	DMSO-*d6*		
NBD1	26 d	MeCN [e]	32 d	MeCN [f]	**From hours to years (~5000 times)**	This work
NBD2	58 d	MeCN [e]	62 d	MeCN [f]		
NBD3	4.0 d	MeCN [e]	39 d	MeCN [f]		
NBD4	92 d	MeCN [e]	1.6 y	MeCN [f]		
NBD5	48 d	MeCN [e]	1.3 y	MeCN [f]		
NBD6	38.4 h	MeCN [e]	24 y	MeCN [f]		

[a] The minimum half-life and solvent used in the reported literature
[b] The maximum half-life and solvent used in the reported literature
[c] The Maximum change in half-life
[d] DMSO−water 1:1 + 25 μL 1,8-Diazabicyclo[5.4.0] undec-7-ene
[e] MeCN, extra dry, 99.9%, over molecular sieve, AcroSeal®
[f] MeCN with water (NBD-to-water molar ratio is 1:20)

3.3 The reversible hydrogen bonding systems

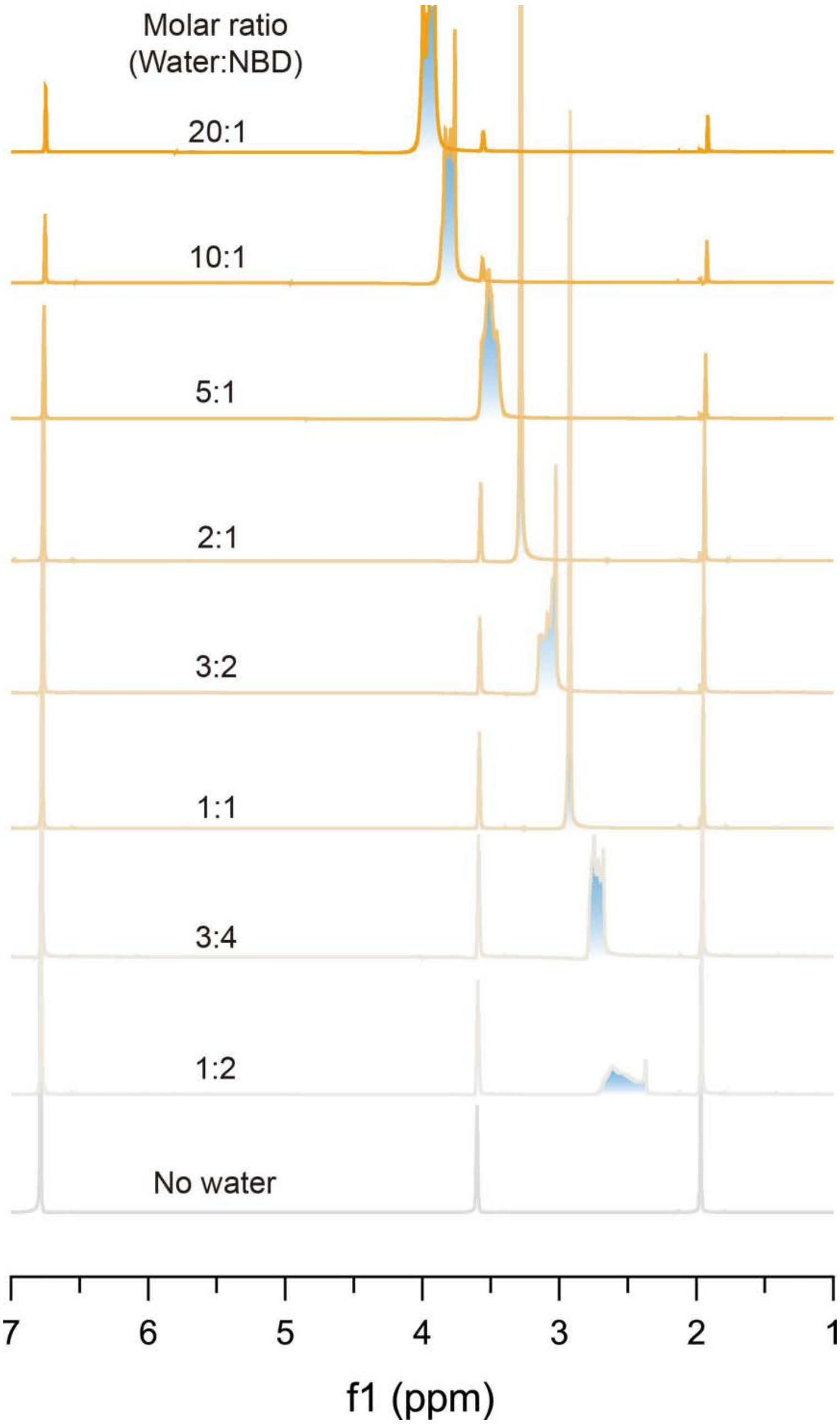

Fig. S82. ^{1}H NMR (400 MHz) of an **NBD/QC** pair with different ratios of water in CD_3CN at 25 °C.

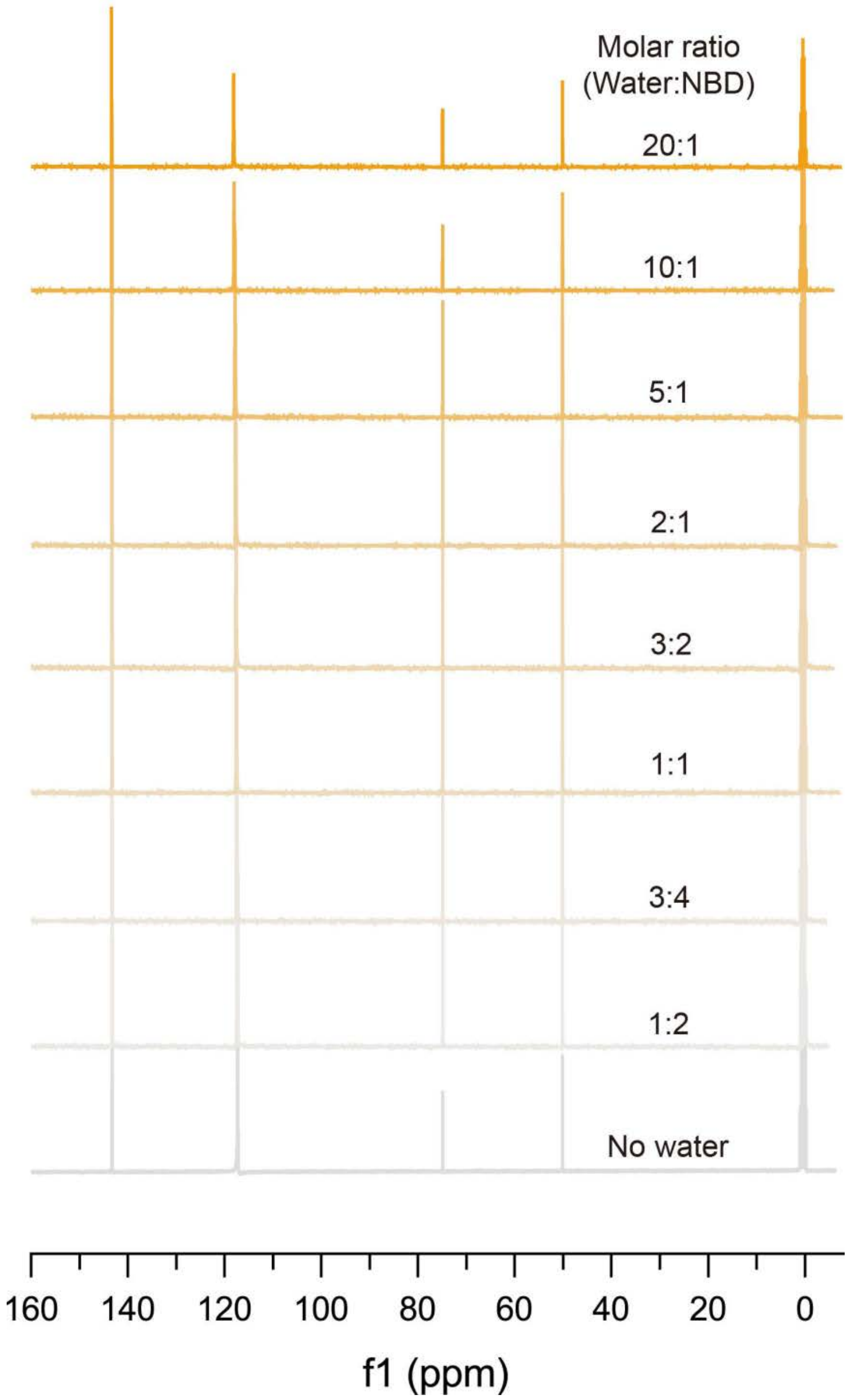

Fig. S83. ^{13}C NMR (101 MHz) of an **NBD/QC** pair with different ratios of water in CD_3CN at 25 °C.

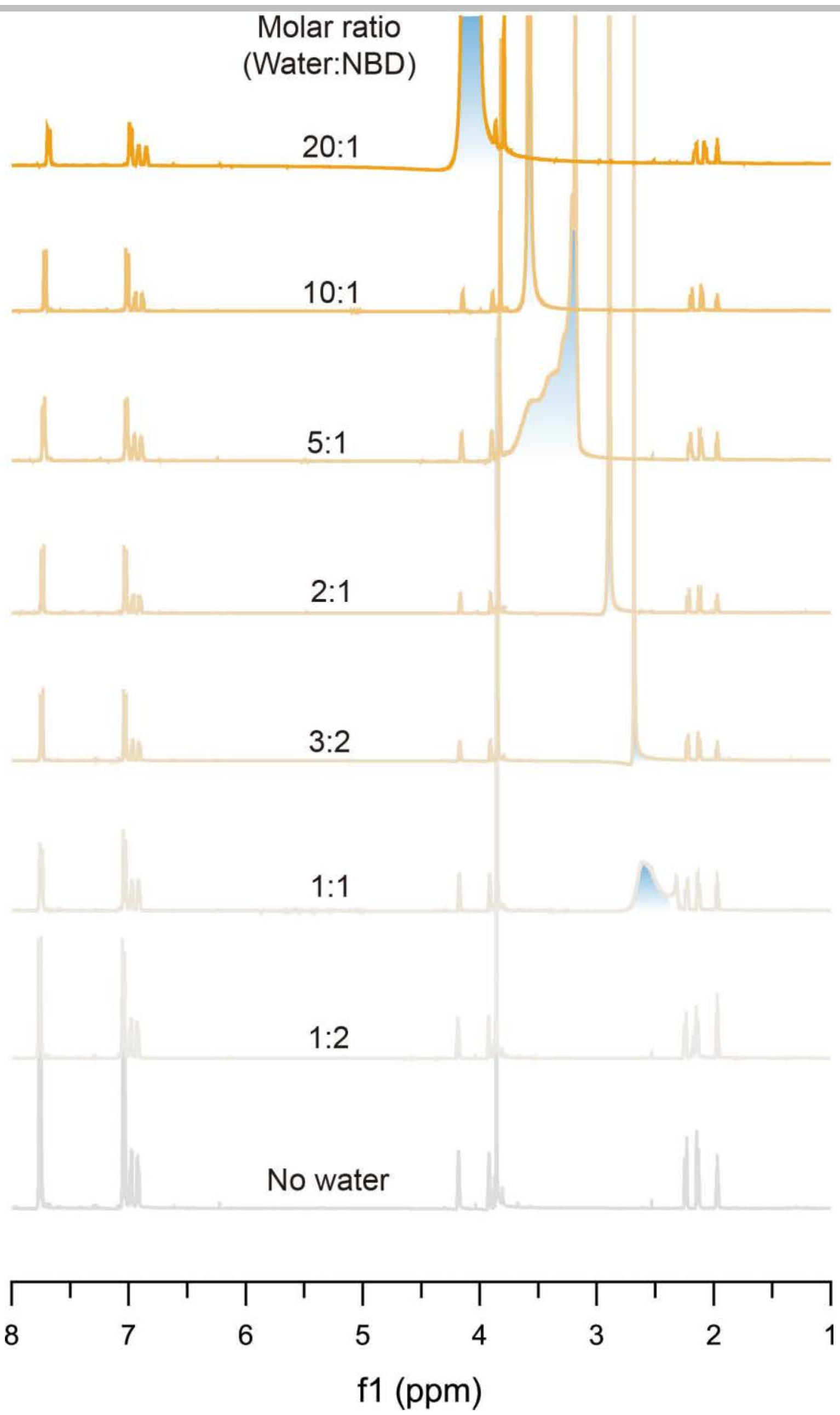

Fig. S84. ^{1}H NMR (400 MHz) of an **NBD2/QC2** pair with different ratios of water in CD_3CN at 25 °C.

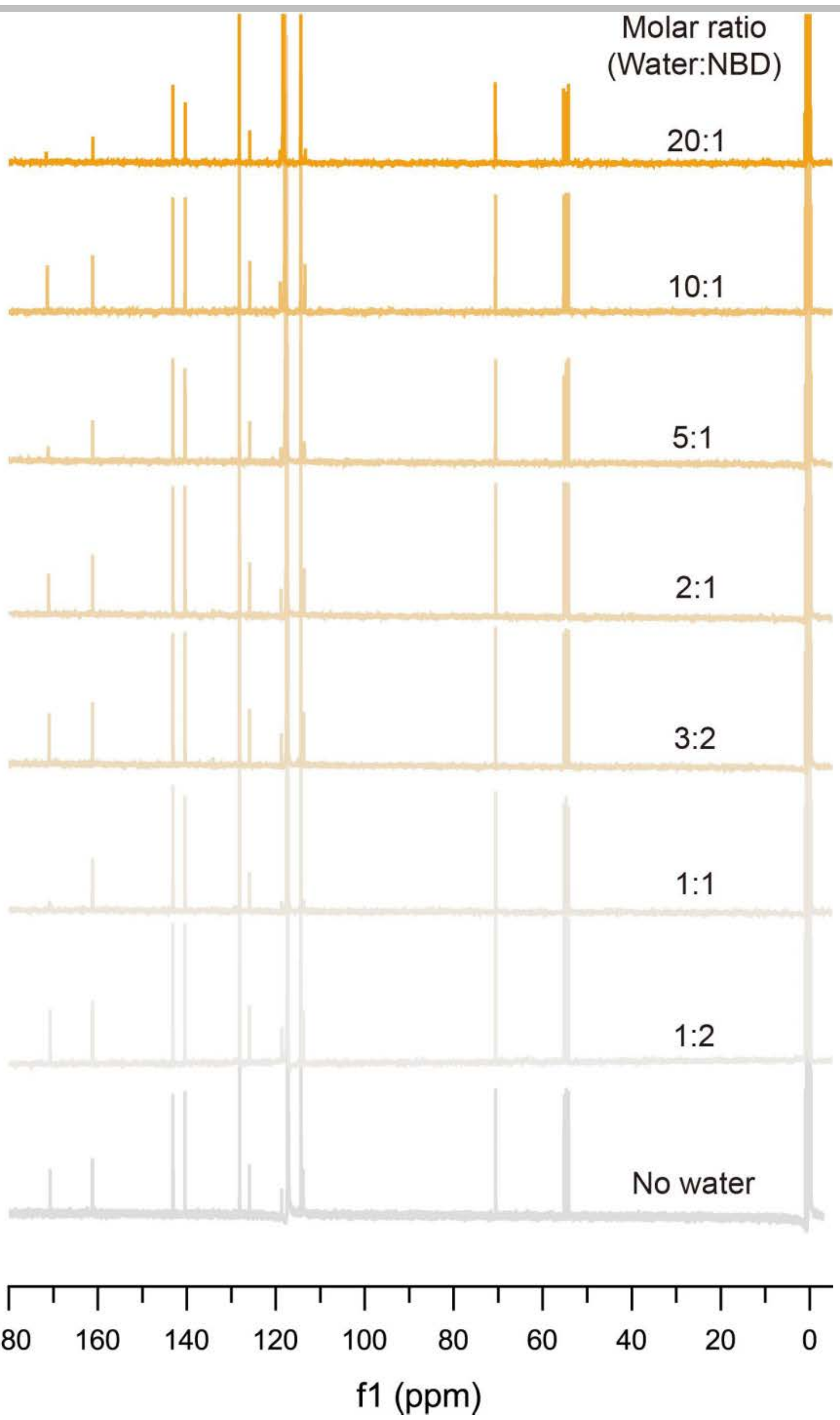

Fig. S85. ^{13}C NMR (101 MHz) of an **NBD2/QC2** pair with different ratios of water in CD_3CN at 25 °C.

4. Theoretical calculation

4.1 Global conformer searches (Suppl. Note 8)

Conformer searches with explicit solvent molecules characterized carbonyl dihedral angle distributions across all NBD and QC derivatives. Ensemble-averaged properties were computed for thermodynamically accessible conformers within 4 times RT (9.92 kJ/mol at 298.15 K). Population-weighted statistics (mean, median, standard deviation) quantified conformational preferences across vacuum, 6 acetonitrile molecules, and 6 water molecules. The dihedral angle coordinate defines two primary rotameric states. Low and high angles (roughly below 90° and above 270°) represent inward-pointing carbonyl oxygens. Intermediate angles (roughly 90-270°) represent outward-pointing carbonyl oxygens that minimize vacuum destabilization but sacrifice aqueous hydrogen-bonding stabilization (Fig. S92).

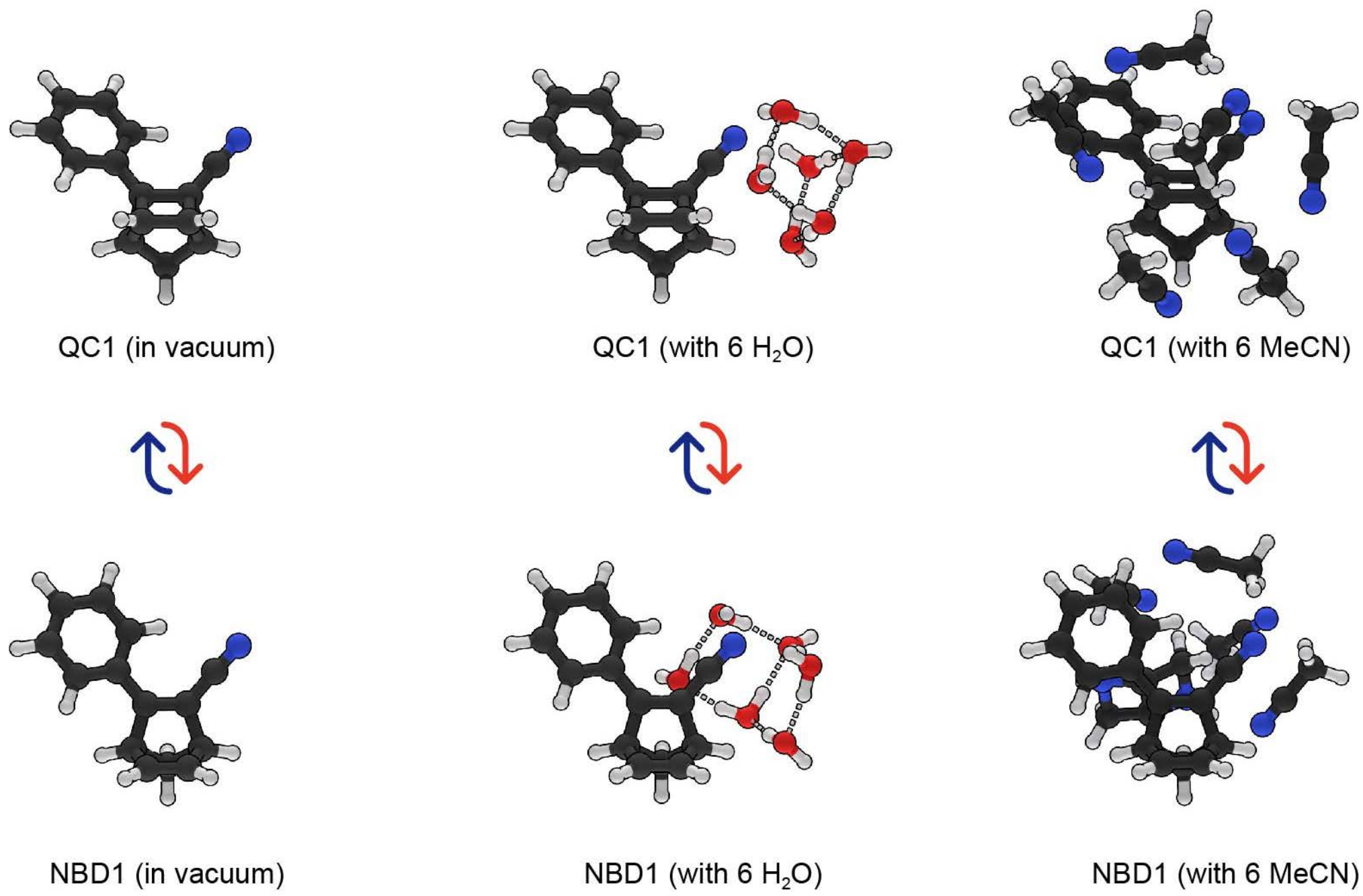

Fig. S86. Optimized structures of **NBD1** and **QC1** in vacuum, with six H_2O molecules, and with six MeCN molecules.

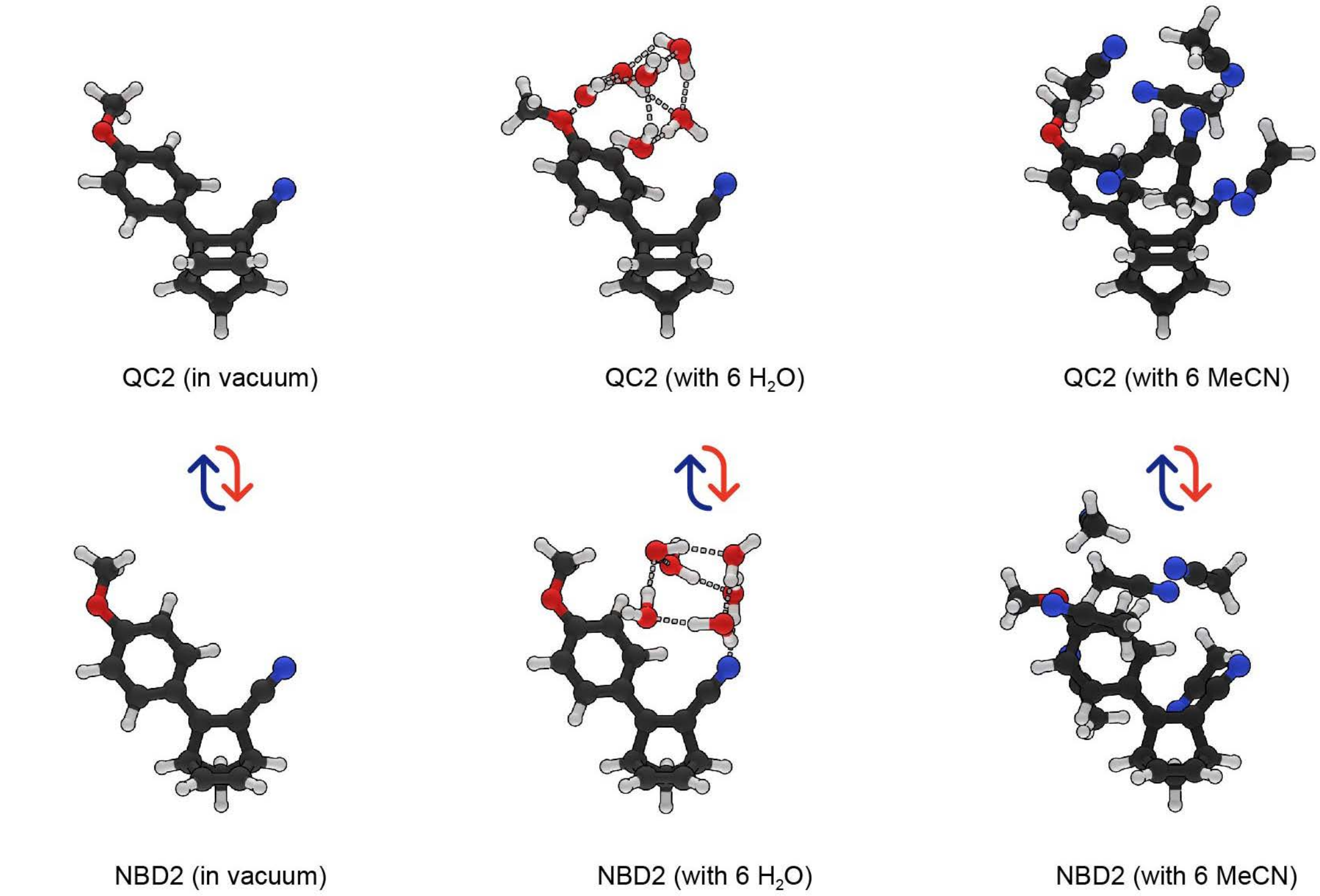

Fig. S87. Optimized structures of **NBD2** and **QC2** in vacuum, with six H_2O molecules, and with six MeCN molecules.

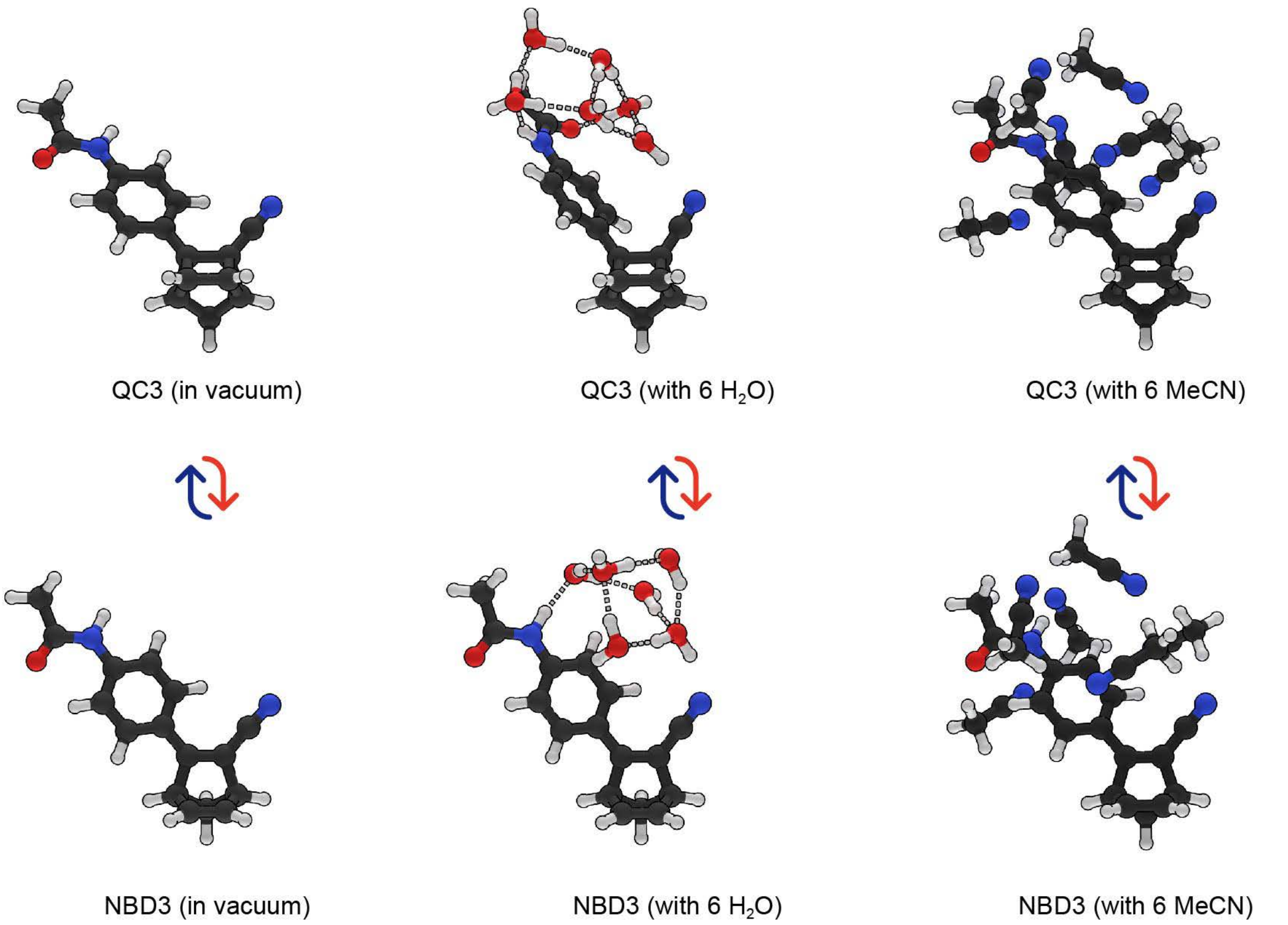

Fig. S88. Optimized structures of **NBD3** and **QC3** in vacuum, with six H_2O molecules, and with six MeCN molecules.

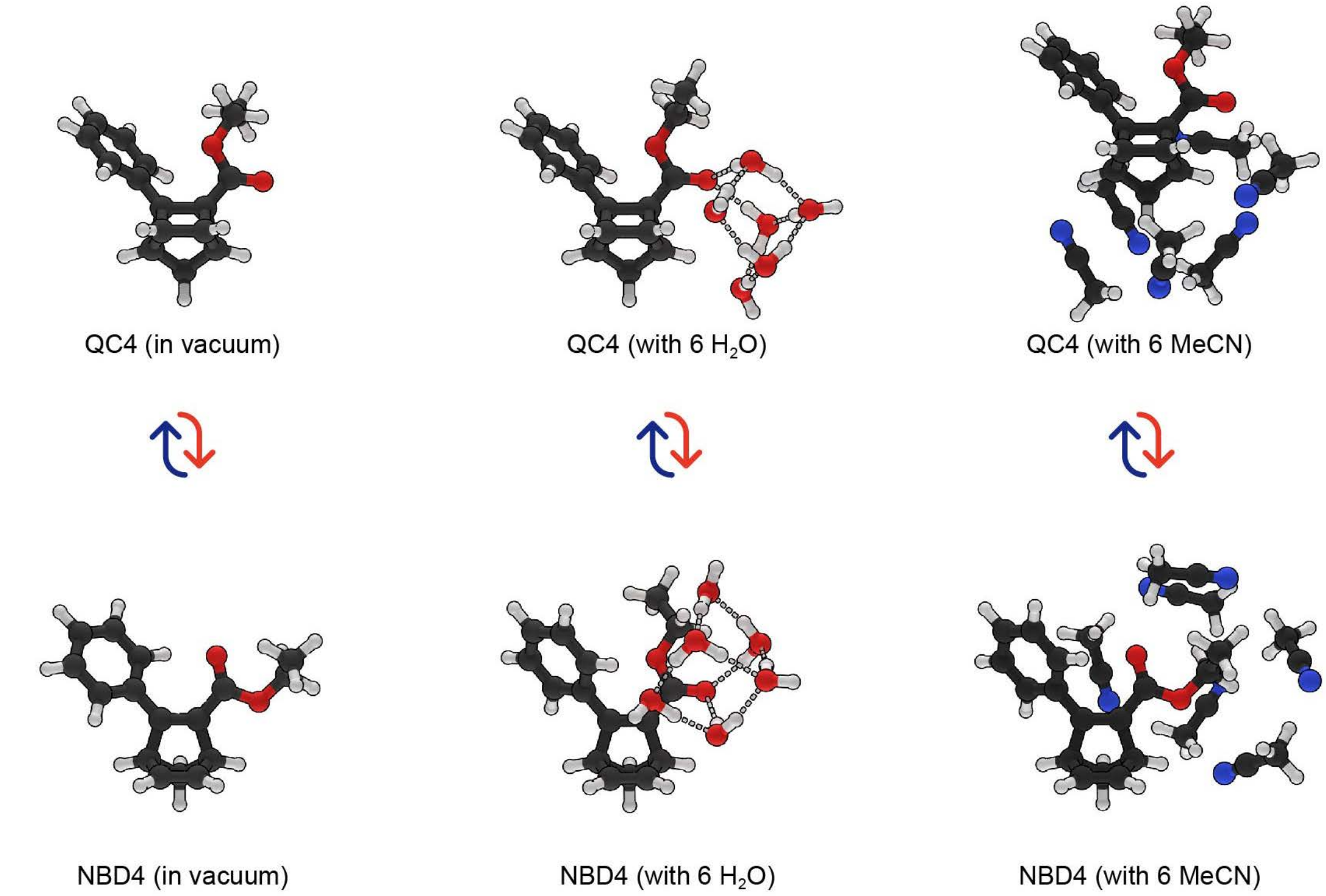

Fig. S89. Optimized structures of **NBD4** and **QC4** in vacuum, with six H_2O molecules, and with six MeCN molecules.

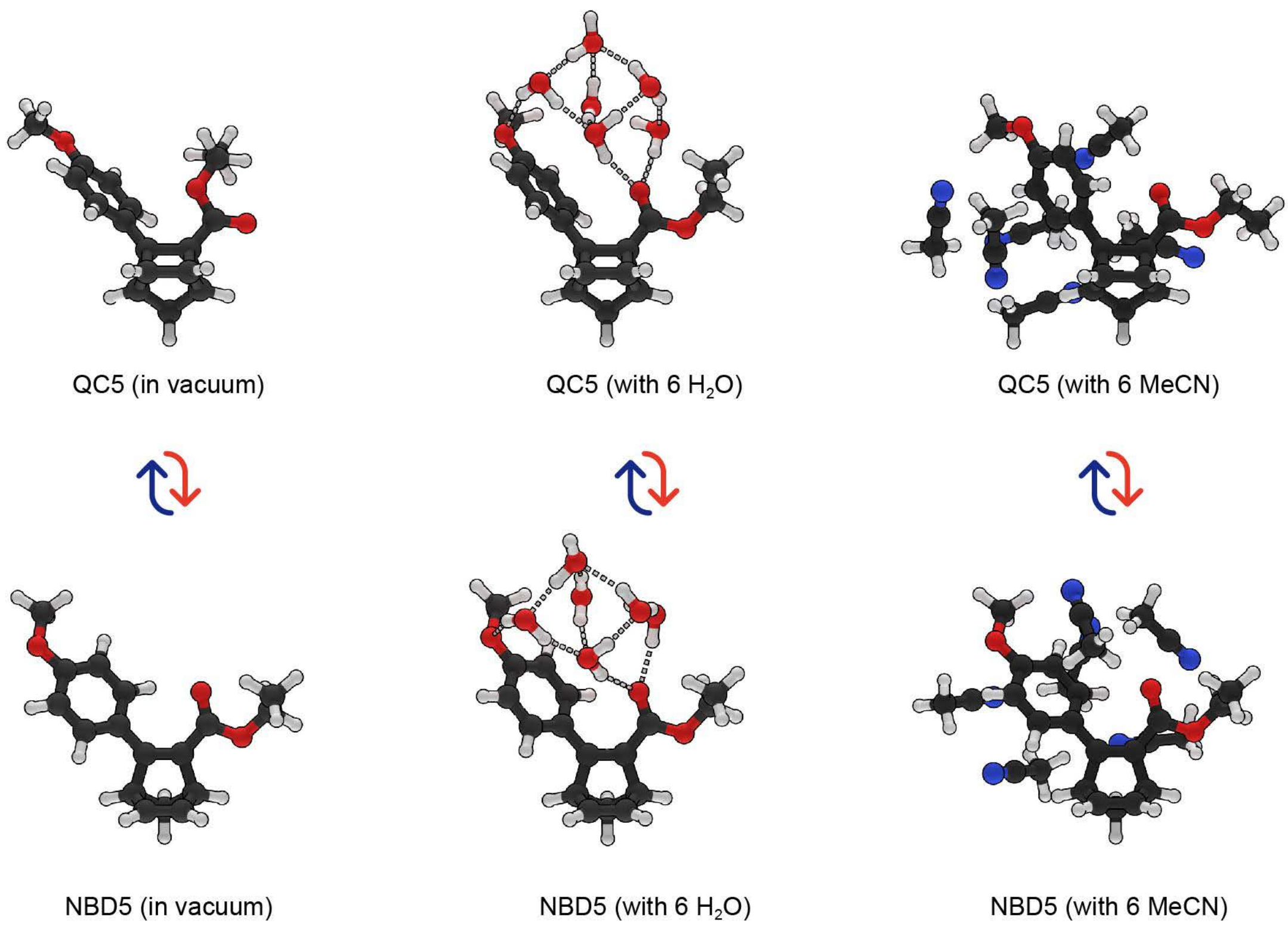

Fig. S90. Optimized structures of **NBD5** and **QC5** in vacuum, with six H_2O molecules, and with six MeCN molecules.

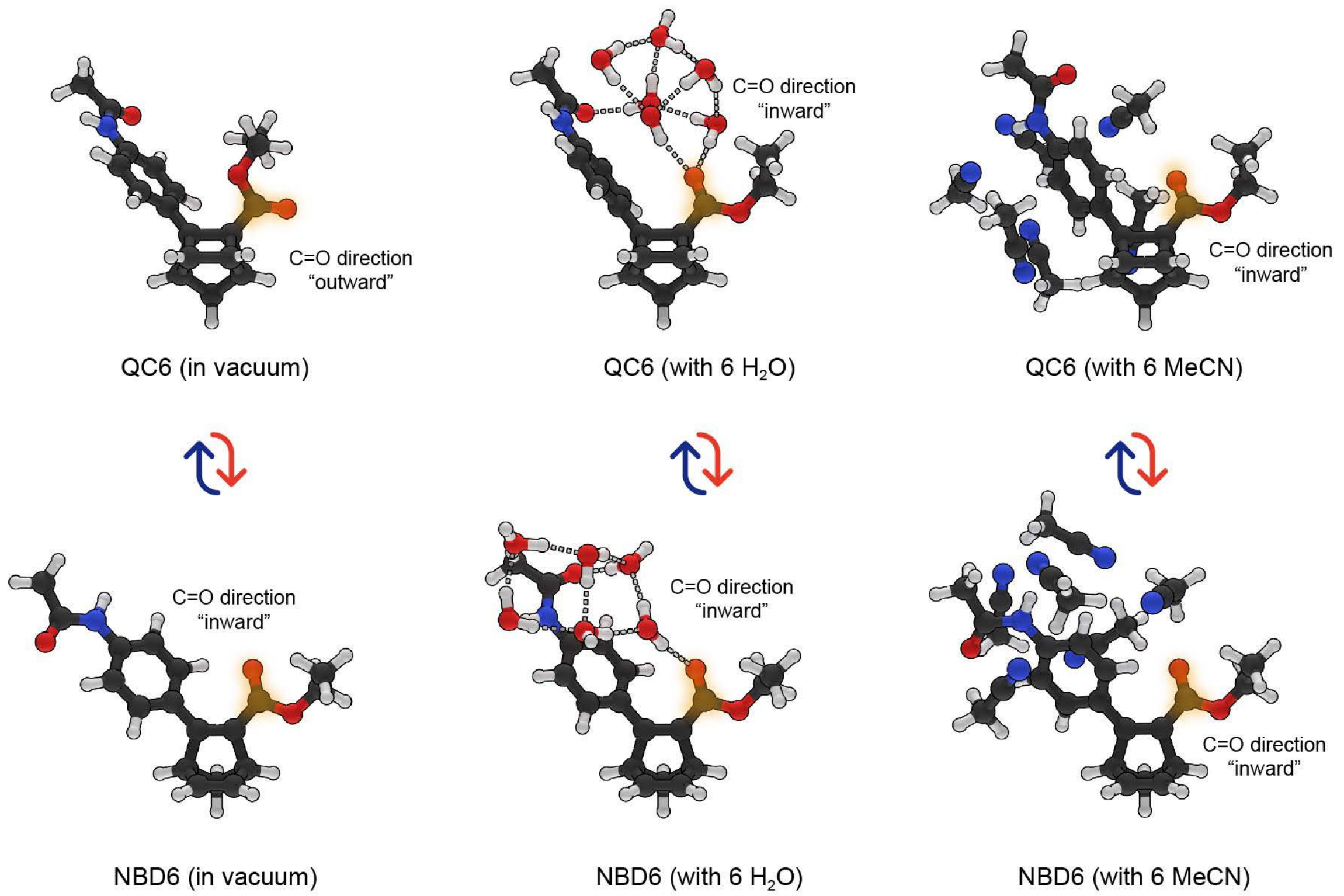

Fig. S91. Optimized structures of **NBD6** and **QC6** in vacuum, with six H2O molecules, and with six MeCN molecules.

Table S28. Energy storage and energy barrier of the NBD/QC pair.

NBD/QC	Energy storage density[a] (kJ mol^{-1})	Energy storage density[a] (MJ kg^{-1})	Backreaction barrier[b] (TS-QC, kJ mol^{-1})	Backreaction barrier[c] (TS-NBD, kJ mol^{-1})
NBD1/QC1	81.26	0.42	232.88	314.13
NBD2/QC2	84.73	0.38	231.79	316.52
NBD3/QC3	81.28	0.32	241.82	323.09
NBD4/QC4	69.06	0.29	239.10	308.17
NBD5/QC5	71.79	0.27	246.66	318.45
NBD6/QC6	71.26	0.24	224.45	295.72

[a] Energy storage density between NBD and QC.
[b] Energy barrier between transition state (TS) and QC.
[c] Energy barrier between transition state (TS) and NBD.

Full optimizations and frequency analyses of each system in vacuum, both minima and transition state, using ωB97X-D4/cc-pVTZ, predicted roughly similar backconversion barriers, with NBD/QC6 predicted to have the lowest energy barrier at 224.25 kJ/mol, calculated from the Gibbs free energy of each state. The backconversion of system 5 is predicted to be 246.66 kJ/mol in pure vacuum, thus further indicating the significantly higher impact of the demon on QC6, given the much higher measured thermal backconversion of QC6 in water containing solvent.

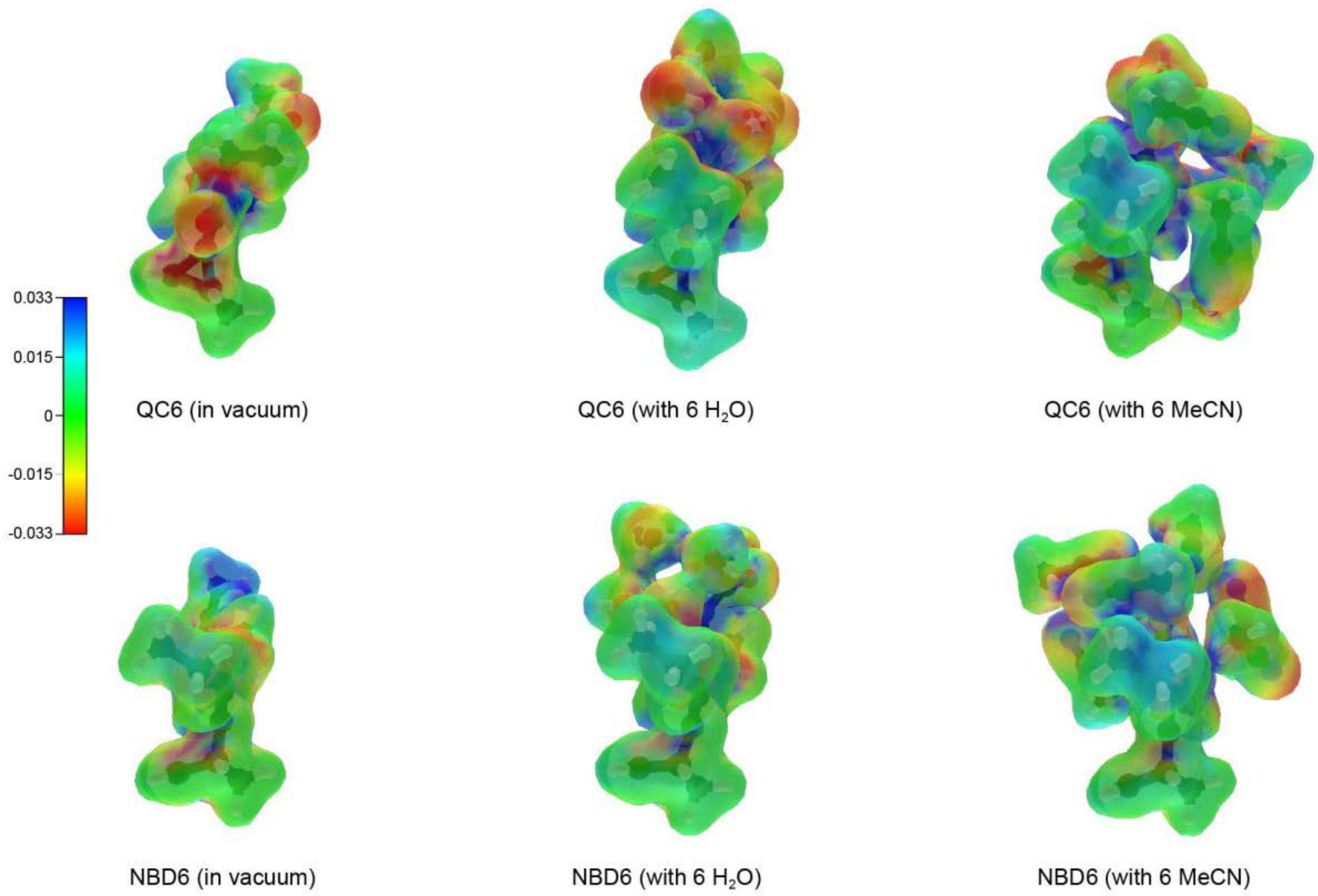

Fig. S92. Electrostatic potential of NBD6/QC6 pair (right view) in vacuum, with six H2O molecules, and with six MeCN molecules. ωB97X-D4/cc-pVTZ electron densities at 0.02 iso values, colored based on the electrostatic potential in a.u.

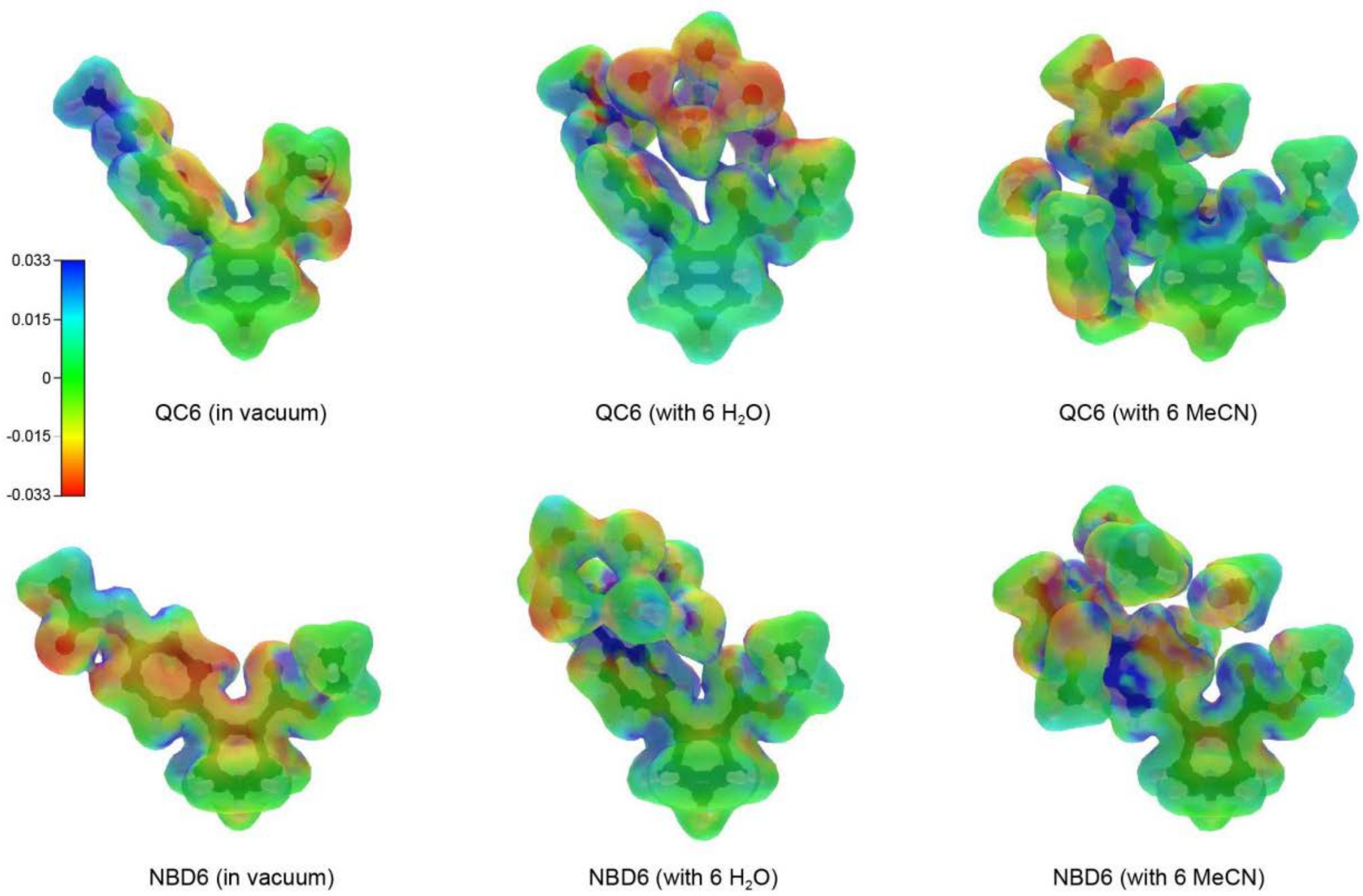

Fig. S93. Electrostatic potential of NBD6/QC6 pair (front view) in vacuum, with six H_2O molecules, and with six MeCN molecules. ωB97X-D4/cc-pVTZ electron densities at 0.02 iso values, colored based on the electrostatic potential in a.u.

4.2 Dihedral angle scan and energy landscape (Suppl. Note 9)

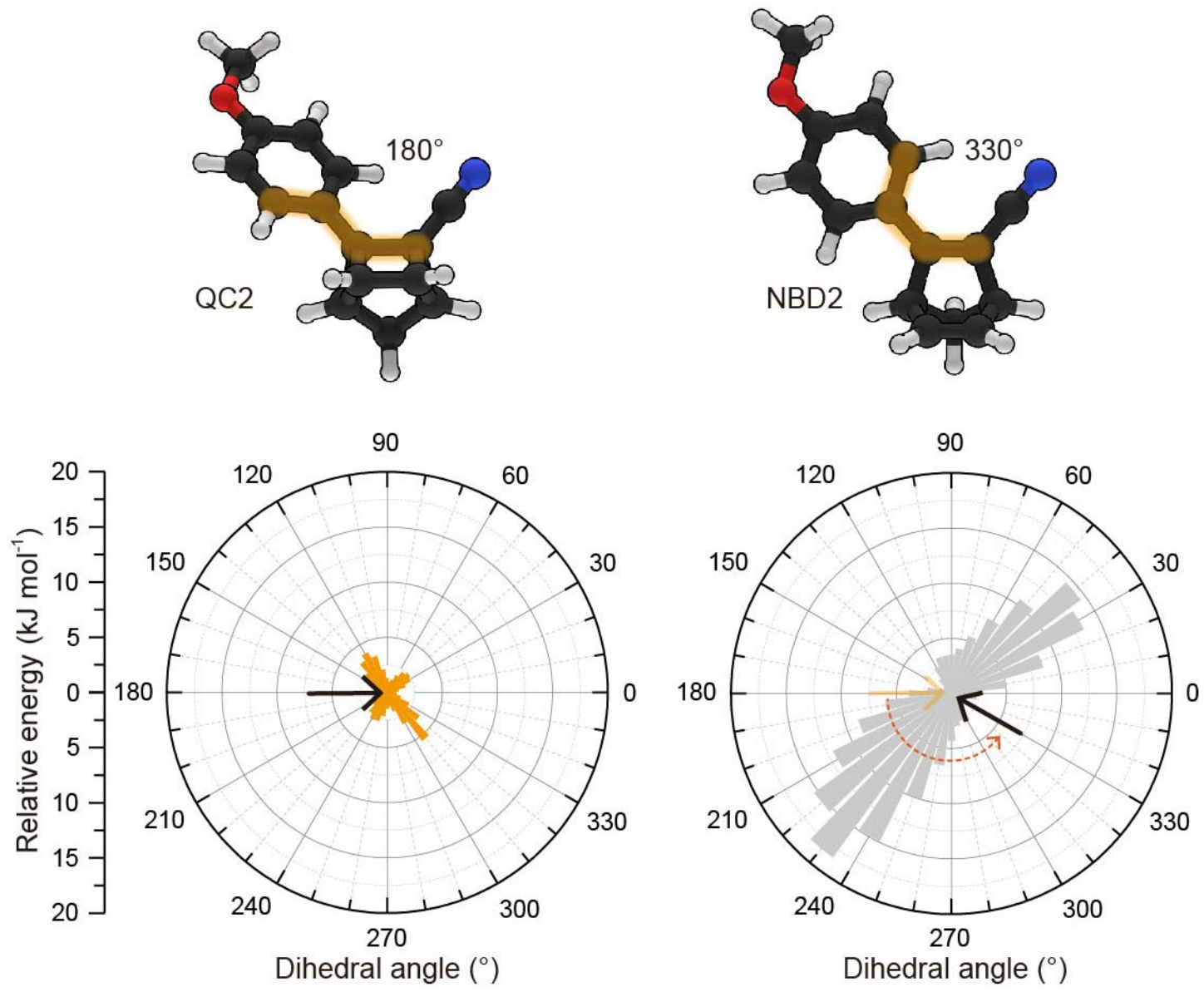

Fig. S94. Dihedral angles and energy landscapes in **NBD2/QC2**.

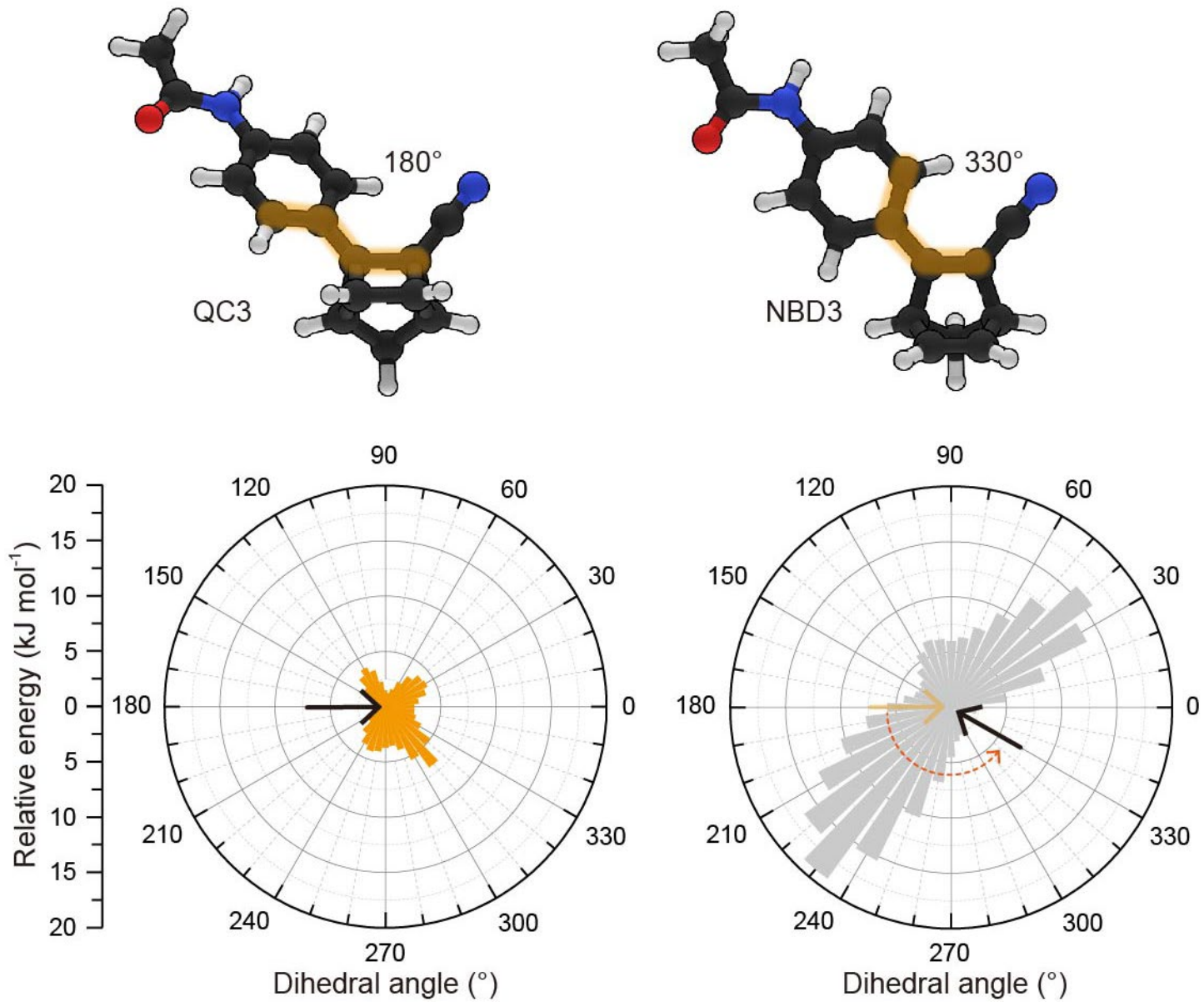

Fig. S95. Dihedral angles and energy landscapes in **NBD3/QC3**.

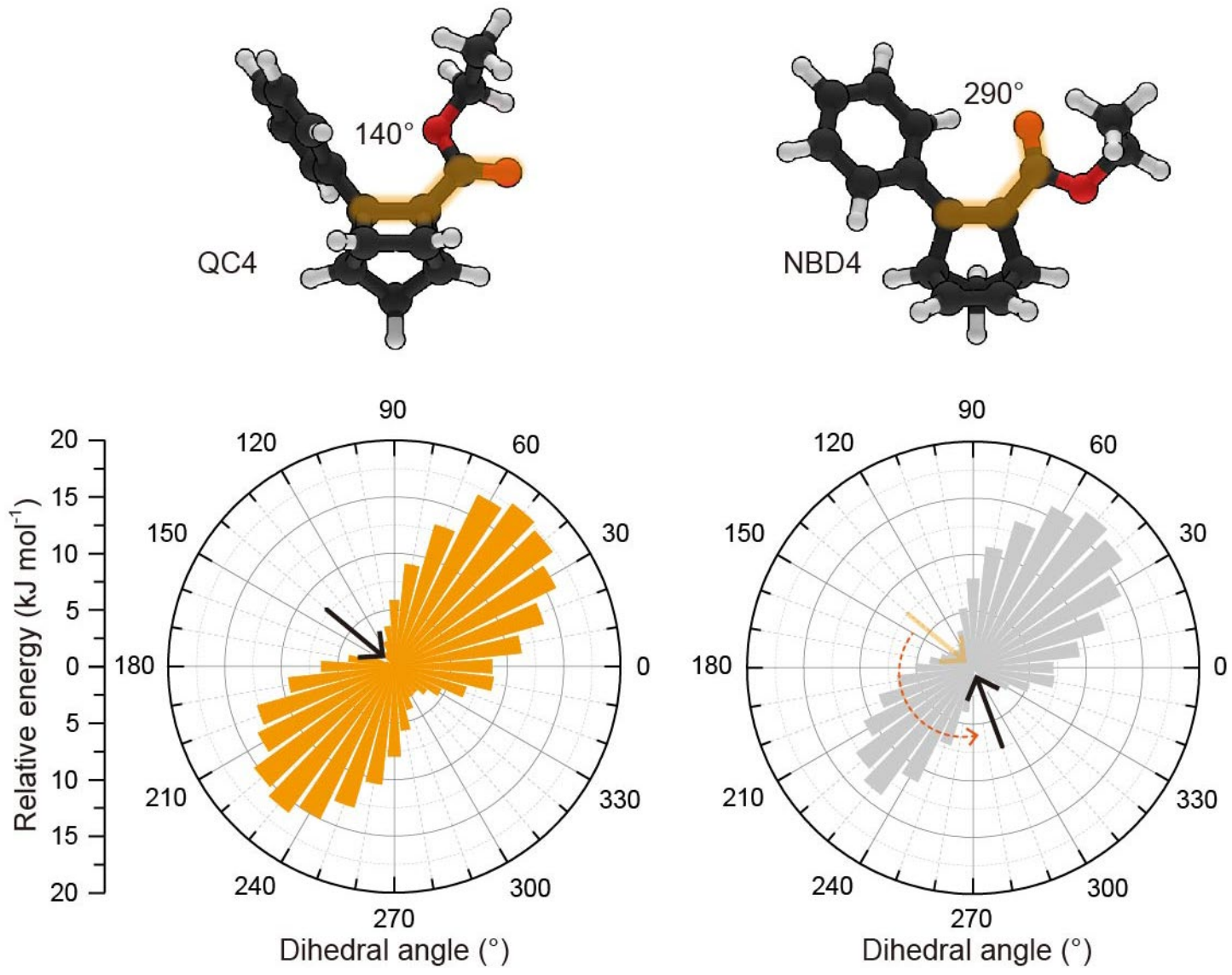

Fig. S96. Dihedral angles and energy landscapes in **NBD4/QC4**.

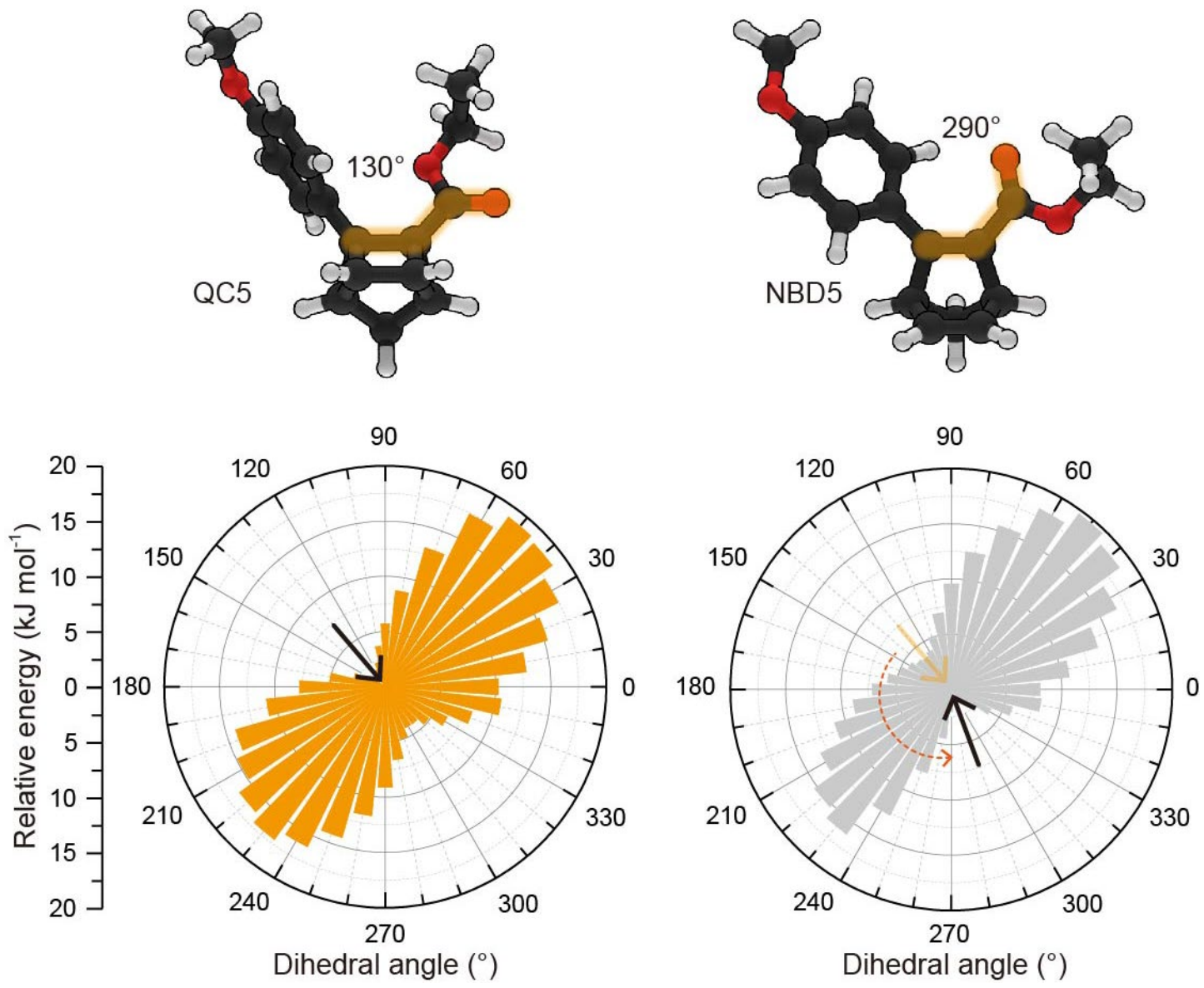

Fig. S97. Dihedral angles and energy landscapes in **NBD5/QC5**.

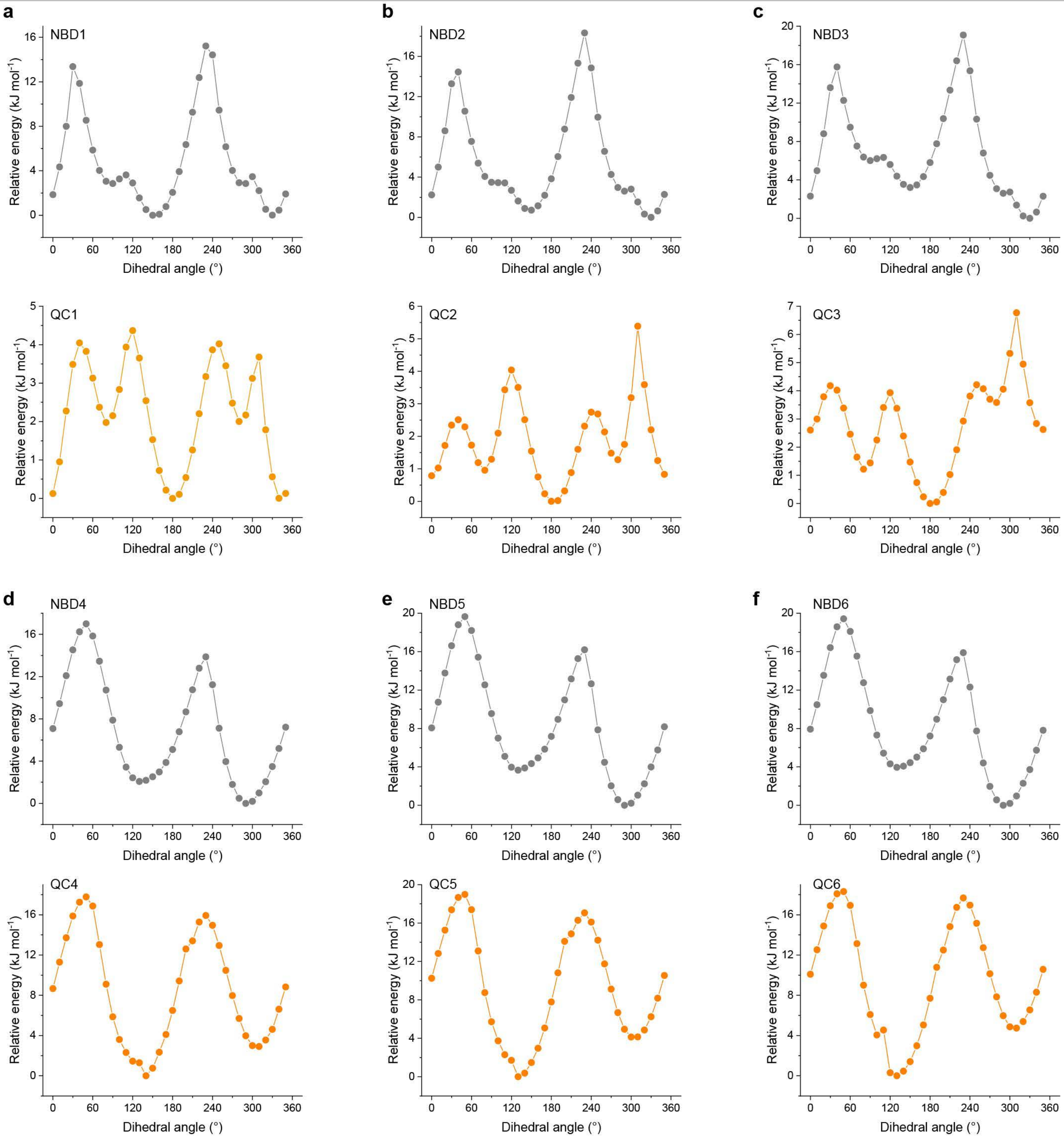

Fig. S98. Dihedral angles and energy landscapes of all NBD/QCs.

The lowest-energy dihedral angles for **QC5** and **QC6** correspond to the outward carbonyl orientation identified in the global optimizations, whereas the water-stabilized inward orientation aligns with the second-lowest minimum in the dihedral profile. In addition, **QC4–6** show markedly higher rotational barriers (~15–20 kJ/mol) than the other QC systems (~5 kJ/mol), reflecting increased conformational rigidity imparted by their larger, hydrogen-bonding side groups.

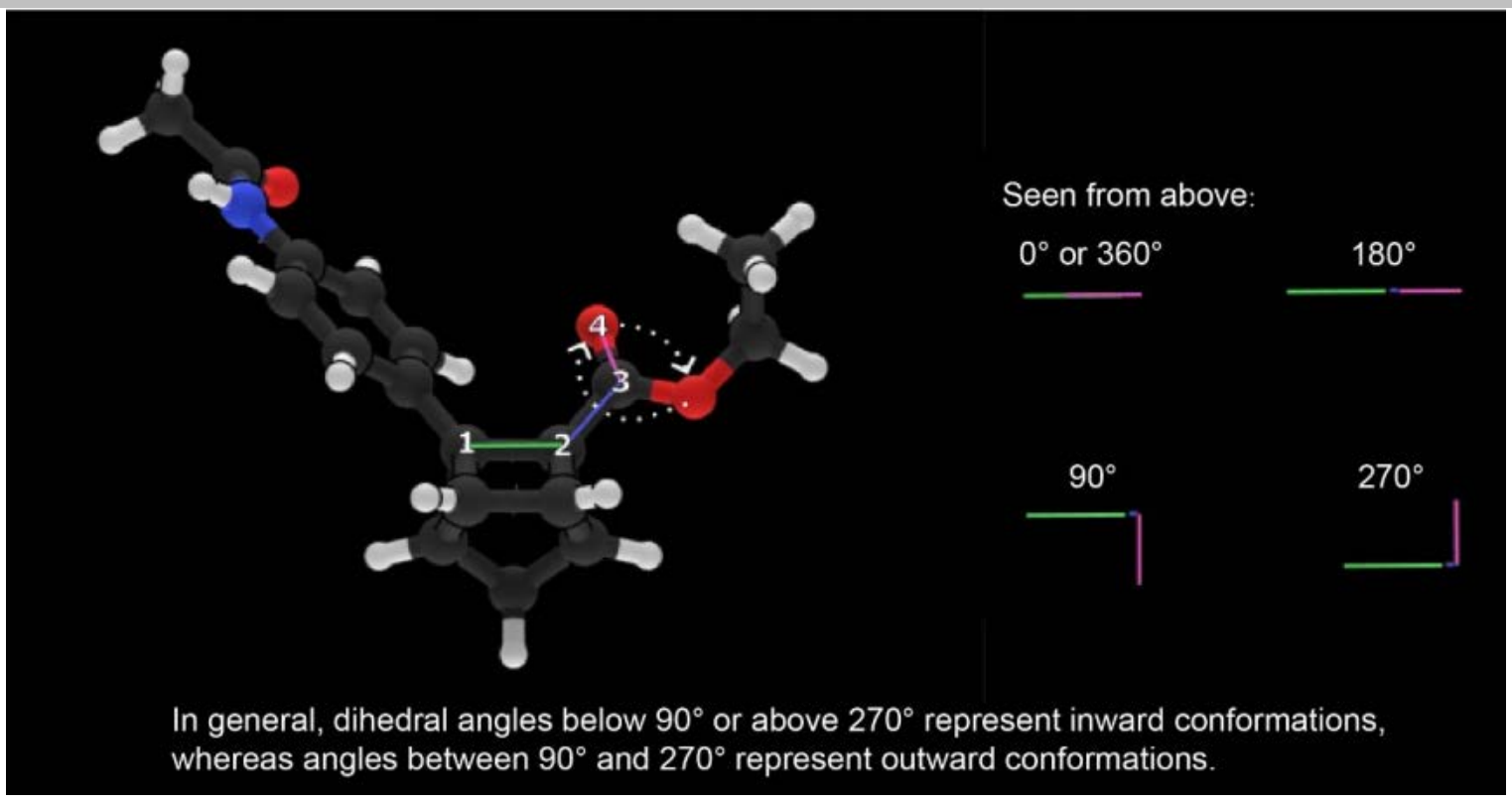

Fig. S99. Dihedral angles of the ester groups in **NBD/QC4-6**, and identification of the inward and outward orientations.

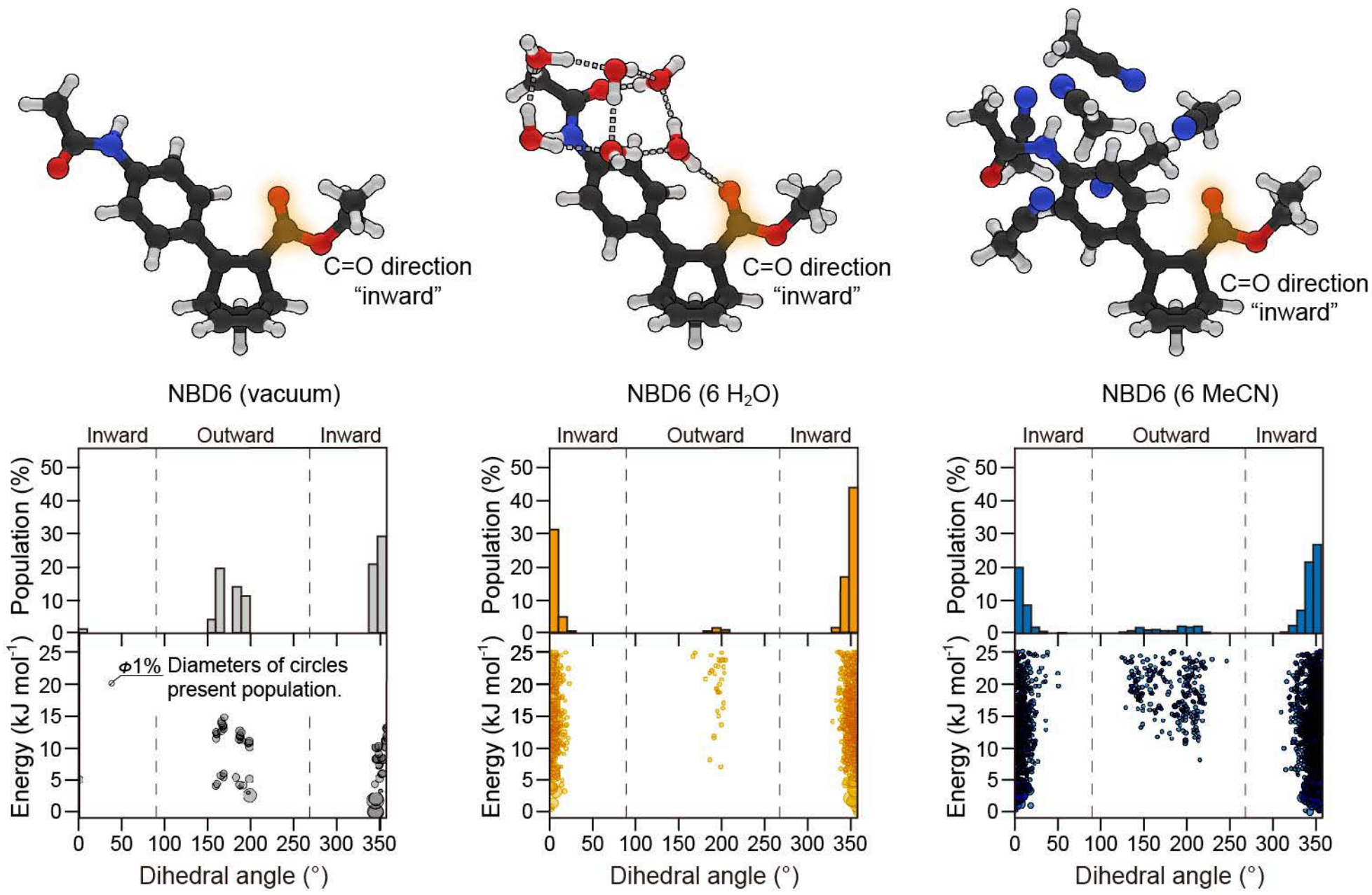

Fig. S100. Inward and outward orientation population and energy landscapes of **NBD6** in vacuum, with six H_2O molecules, and with six MeCN molecules.

The population histograms are made using 10° dihedral angle bins, starting with 0°-10°. In the scatter plots, conformers are grouped into 10° dihedral angle and 5 kJ/mol energy bins. For each conformer, the population percentage in its bin is calculated relative to total conformers. Dot sizes are scaled linearly according to equation as below, where p_{max} is the busiest bin in the dataset. This produces a range of 5-90 size units, allowing visual assessment of conformational population density across the dihedral-energy landscape.

$$s_i = s_{min} + \Delta s \cdot \frac{p_i}{p_{max}} \quad (S6)$$

where

s_i = dot size for conformer i

s_{min} = minimum dot size = 5

Δs = scaling amplitude = 85

p_i = population percentage in the bin containing conformer i

p_{max} = maximum population percentage across all bins

The change in lowest-energy conformation, a statistical shift is also seen across the Boltzmann-weighted population. Acetonitrile favors the outward pointing carbonyl oxygen in the lower-energy region of the population, while the inwards orientation is more pronounced across the entire population. Water induces a stark redistribution of the conformer ensemble almost entirely eliminating the presence of conformers with outwards pointing carbonyl oxygens.

All NBD4–6 derivatives showed broad, solvent-insensitive dihedral distributions (SD 76–173°). Mean dihedral angles spanned 160.6-191.8° for NBD4 (SD 53.5–100.0°), 99.8-299.6° for NBD5 (SD 76.3–173.3°), and 205.0-310.2° for NBD6 (SD 85.9-172.7°). Solvent-induced mean shifts were <50°, demonstrating that their conformational landscapes are largely invariant to hydrogen-bonding environments.

Similar dihedral-angle dependence appears along the minimum-energy paths for NBD-QC interconversion in vacuum. For systems 4, 5, and 6, the NBD→QC transition begins with roughly a half rotation of the carbonyl, from the inward-pointing orientation to an outward one, followed by bond reorientation within the NBD/QC core. A comparable half rotation precedes bond rearrangement in the QC→NBD direction. Thus, the transition either requires, or strongly favors, an out-of-plane carbonyl orientation close to that energetically preferred in the QC states of systems 4-6. Stabilizing the inward carbonyl geometry in the QC state would therefore hinder QC→NBD conversion, or at least increase its energetic cost, because the hydrogen-bonded carbonyl must rotate away from its inward position to permit core bond rearrangement.

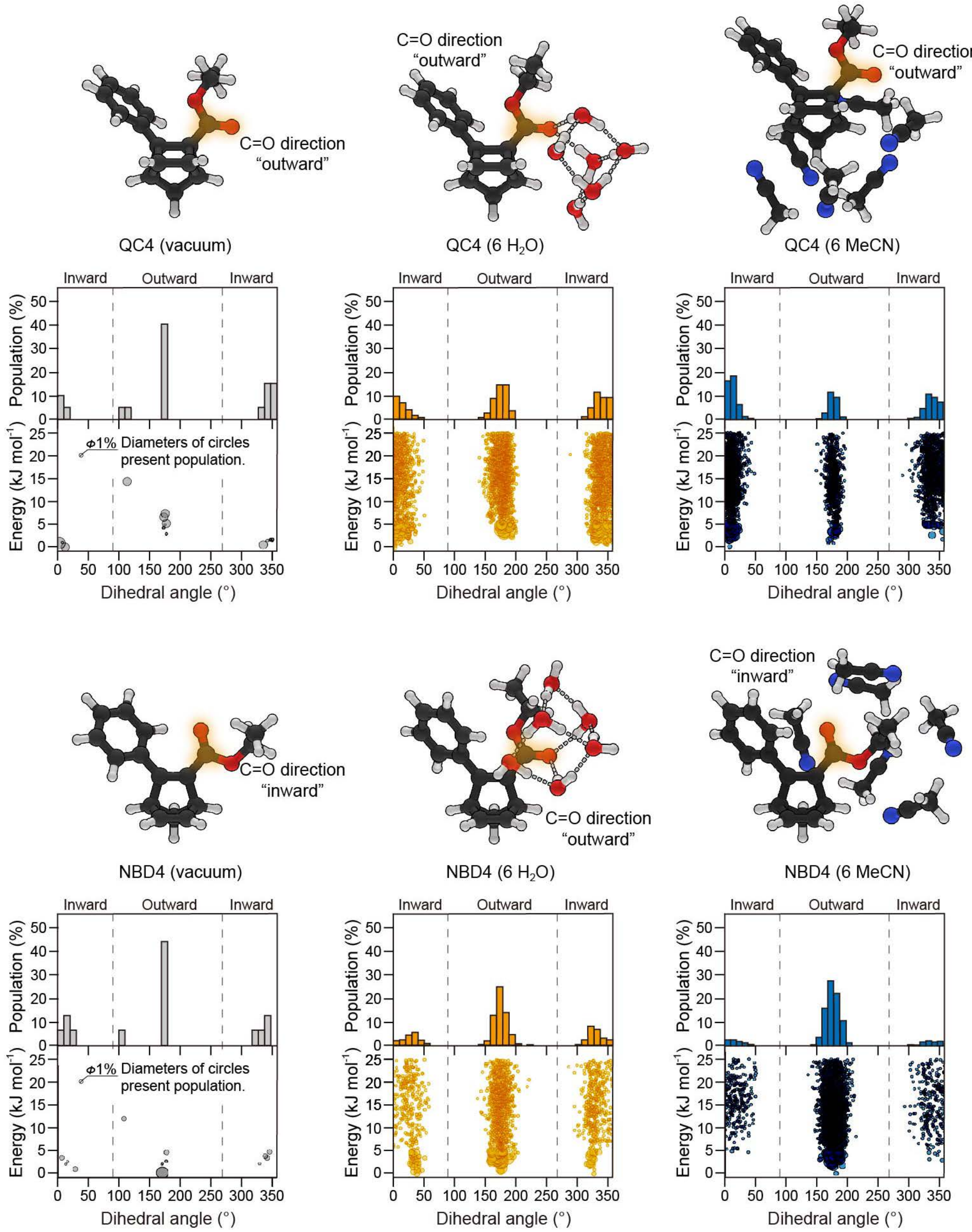

Fig. S101. Inward and outward orientation population and energy landscapes of **QC4** and **NBD4** in vacuum, with six H_2O molecules, and with six MeCN molecules.

QC4 showed moderate solvent sensitivity, exhibiting trimodal dihedral distributions. In vacuum, populations were centered near 10° (16%), 190° (17%), and 350° (27%). With six acetonitrile molecules (mean 148.9°, median 177.2°), a broad distribution persisted, with populations near 10° (5%), 170-210° (~37%), and 310-350° (~21%). Six water molecules (mean 186.2°, median 181.7°) broadened the central, outward-pointing region, distributing ~45% of the population across 150-310°, while maintaining substantial inward populations at 10-30° (~20%) and 330–350° (~19%). Molecular dynamics revealed a 24% reduction in flexibility, with standard deviations decreasing from 87.1° in vacuum to 66.4° in water.

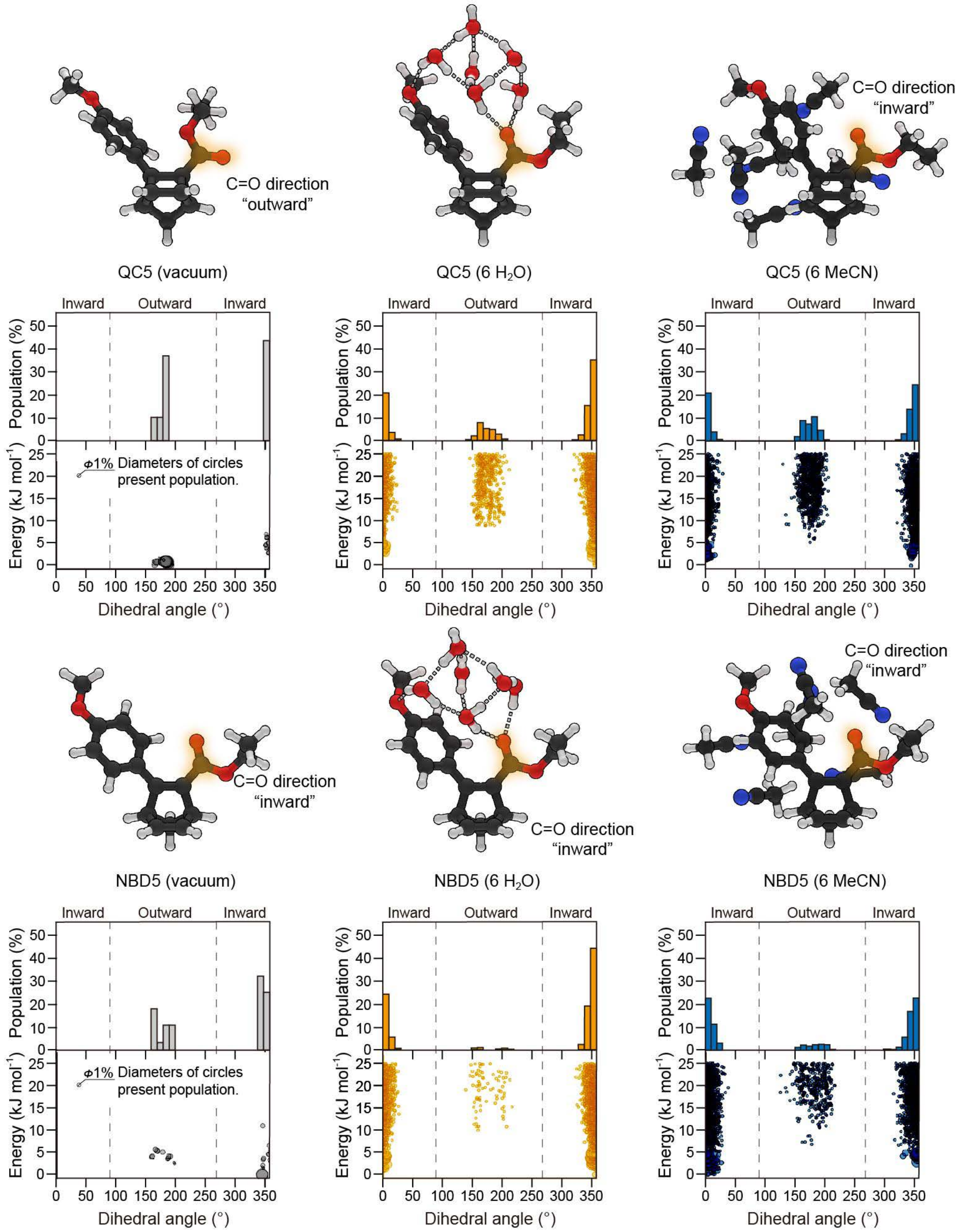

Fig. S102. Inward and outward orientation population and energy landscapes of **QC5** and **NBD5** in vacuum, with six H_2O molecules, and with six MeCN molecules.

QC5 exhibited pronounced solvent-induced reorganization with a clear bimodal character. In vacuum, populations were centered near 190° (56%) and 350° (16%). With six acetonitrile molecules (mean 197.1°, median 191.8°), broad distributions persisted, with ~28% in the 190-230° region and ~49% in the 310-350° region. Six water molecules (mean 266.0°, median 353.5°) induced a striking shift: ~69% of the population concentrated at 350° (inward conformation) and ~22% at 10-30°, effectively eliminating the vacuum-preferred outward orientation.

Molecular dynamics revealed a 36% reduction in flexibility (SD 93.9°→60.1°) and an ~100° shift in the mean dihedral angle (156.4°→256.8°), consistent with the outward-to-inward transition.

QC5 underwent the most significant transformation, with its standard deviation narrowing by 36% (93.9° to 60.1°) and a mean dihedral shift of approximately 100° from 156.4° to 256.8°. This reorientation directly corresponds to the transition from the vacuum-preferred outward carbonyl geometry to the water-stabilized inward orientation predicted by global conformer searches.

In short, water's strong donor-acceptor character enables structured bridges between side groups, selectively stabilizing inward-pointing geometries (~350° and 0-30°). Acetonitrile, a weaker acceptor, shows reduced but measurable inward preference, particularly for the longer derivatives (**QC5-6**). The systematic shifts in populations, from vacuum bimodal distributions to >70% inward conformations in water for **QC5** and **QC6**, demonstrate progressive solvent control. Mean–median divergences exceeding 100° further indicate dual-state populations consistent with substantial rotational barriers of 15-20 kJ mol^{-1}.

4.3 Molecular dynamics simulations (Suppl. Note 10)

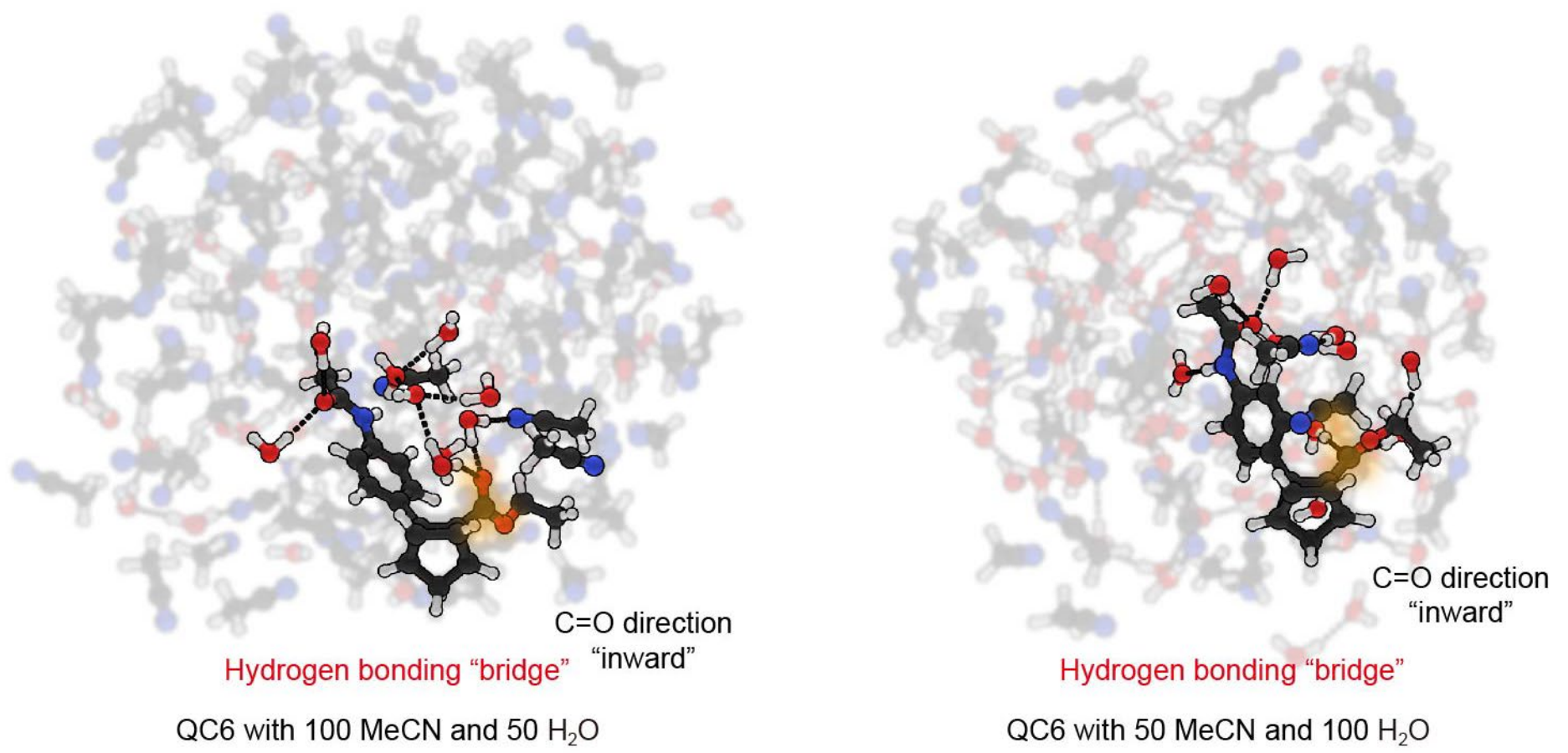

Fig. S103. 3D structures of **QC6** at 298.15 K. The carbonyl dihedral angle was monitored across 2.5 ns trajectories under two solvent conditions: mixed H_2O/MeCN solutions (50 H_2O/100 MeCN and 100 H_2O/50 MeCN).

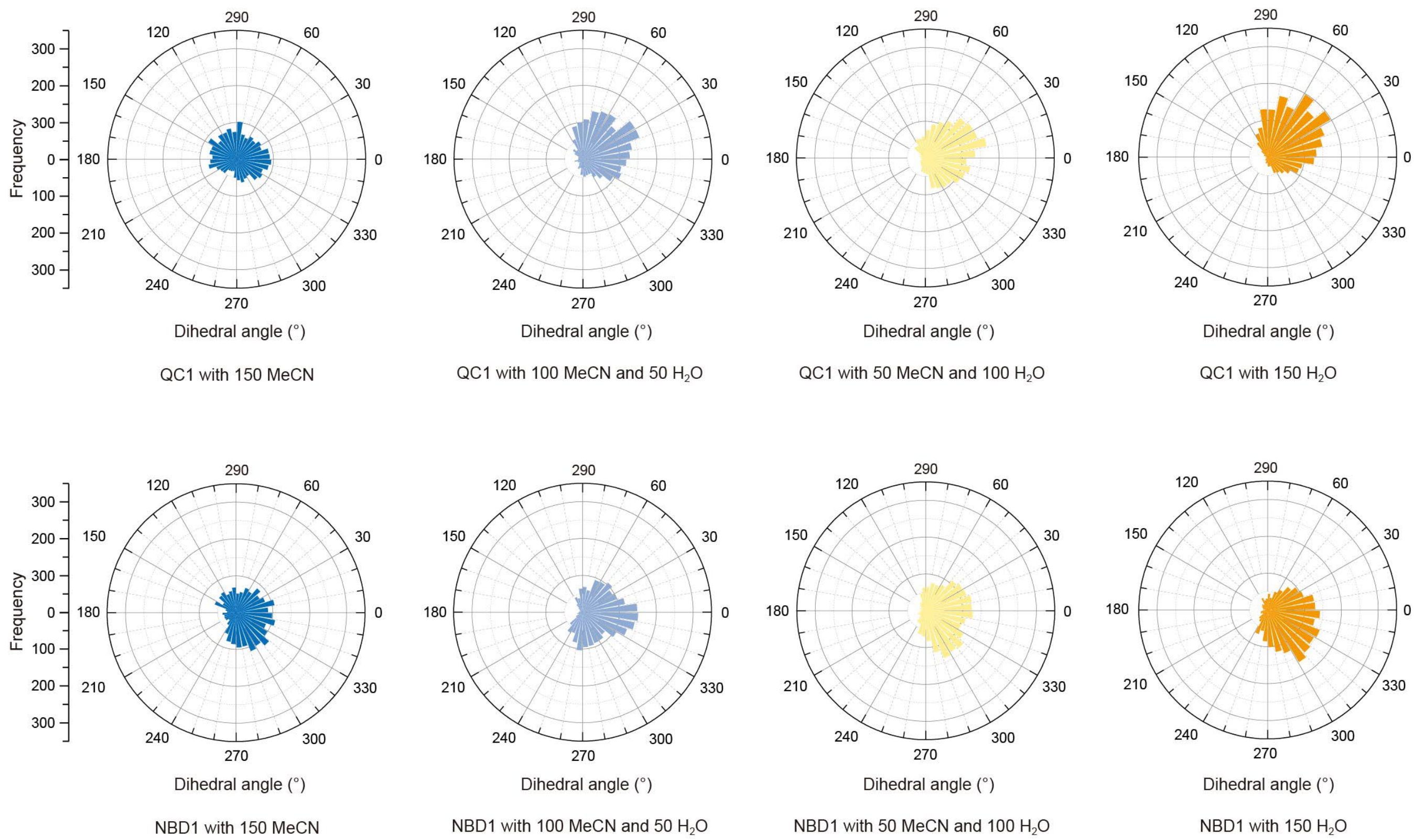

Fig. S104. Molecular dynamics and relative structures of **NBD1/QC1** at 298.15 K. The carbonyl dihedral angle was monitored across 2.5 ns trajectories under four solvent conditions: 150 explicit MeCN molecules, mixed H_2O/MeCN solutions (50 H_2O/100 MeCN and 100 H_2O/50 MeCN), and 150 explicit H_2O molecules.

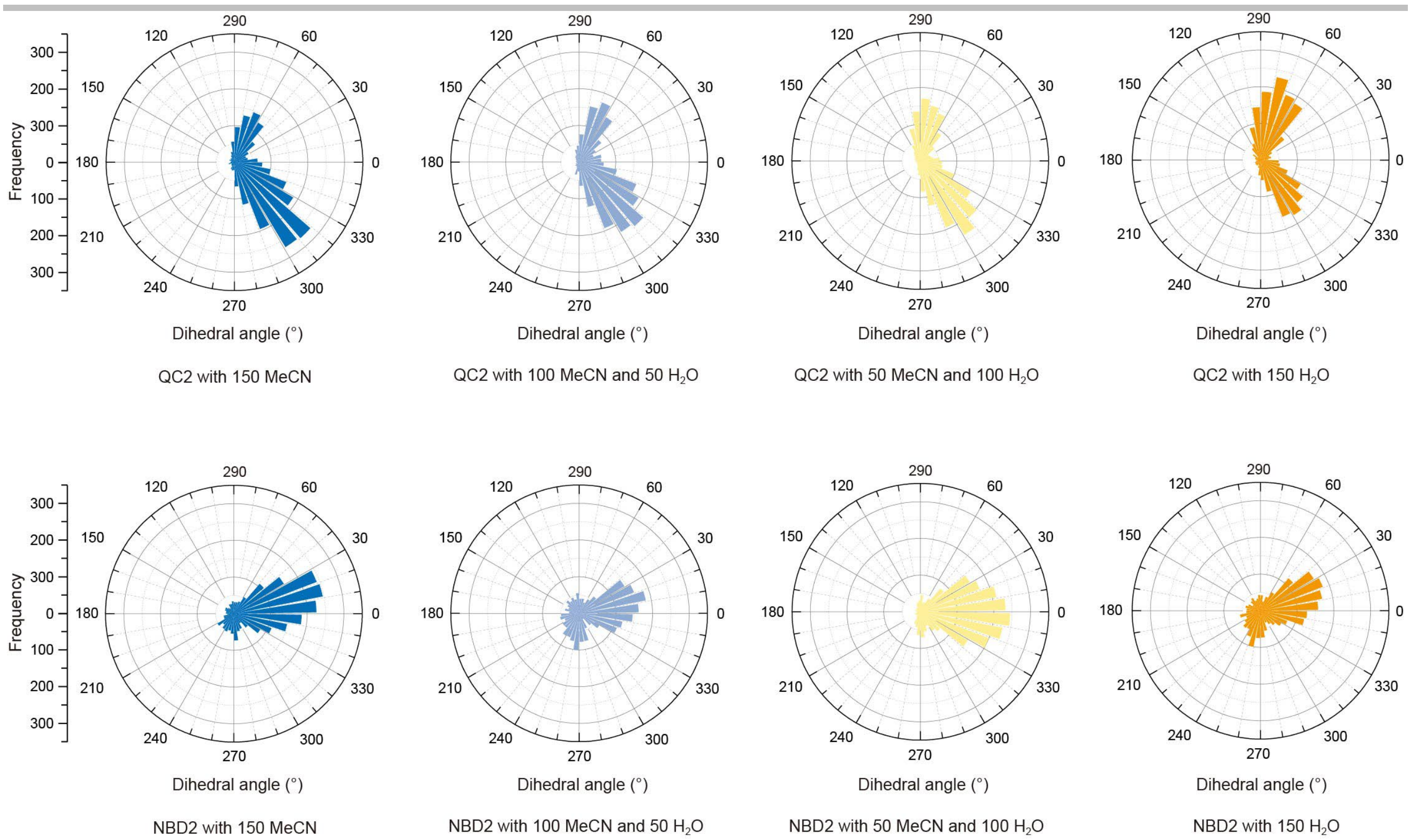

Fig. S105. Molecular dynamics and relative structures of **NBD2/QC2** at 298.15 K. The carbonyl dihedral angle was monitored across 2.5 ns trajectories under four solvent conditions: 150 explicit MeCN molecules, mixed H_2O/MeCN solutions (50 H_2O/100 MeCN and 100 H_2O/50 MeCN), and 150 explicit H_2O molecules.

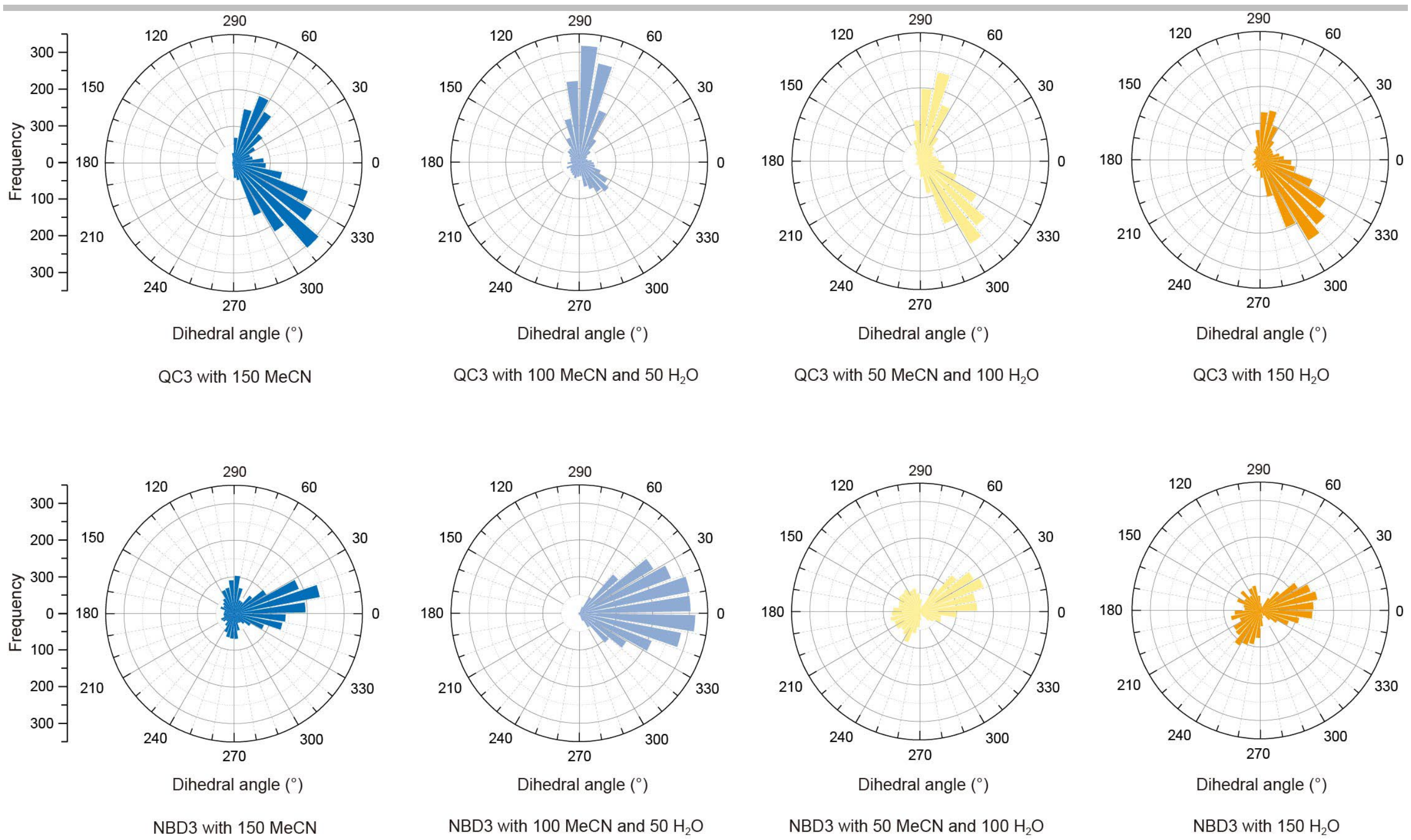

Fig. S106. Molecular dynamics and relative structures of **NBD3/QC3** at 298.15 K. The carbonyl dihedral angle was monitored across 2.5 ns trajectories under four solvent conditions: 150 explicit MeCN molecules, mixed H_2O/MeCN solutions (50 H_2O/100 MeCN and 100 H_2O/50 MeCN), and 150 explicit H_2O molecules.

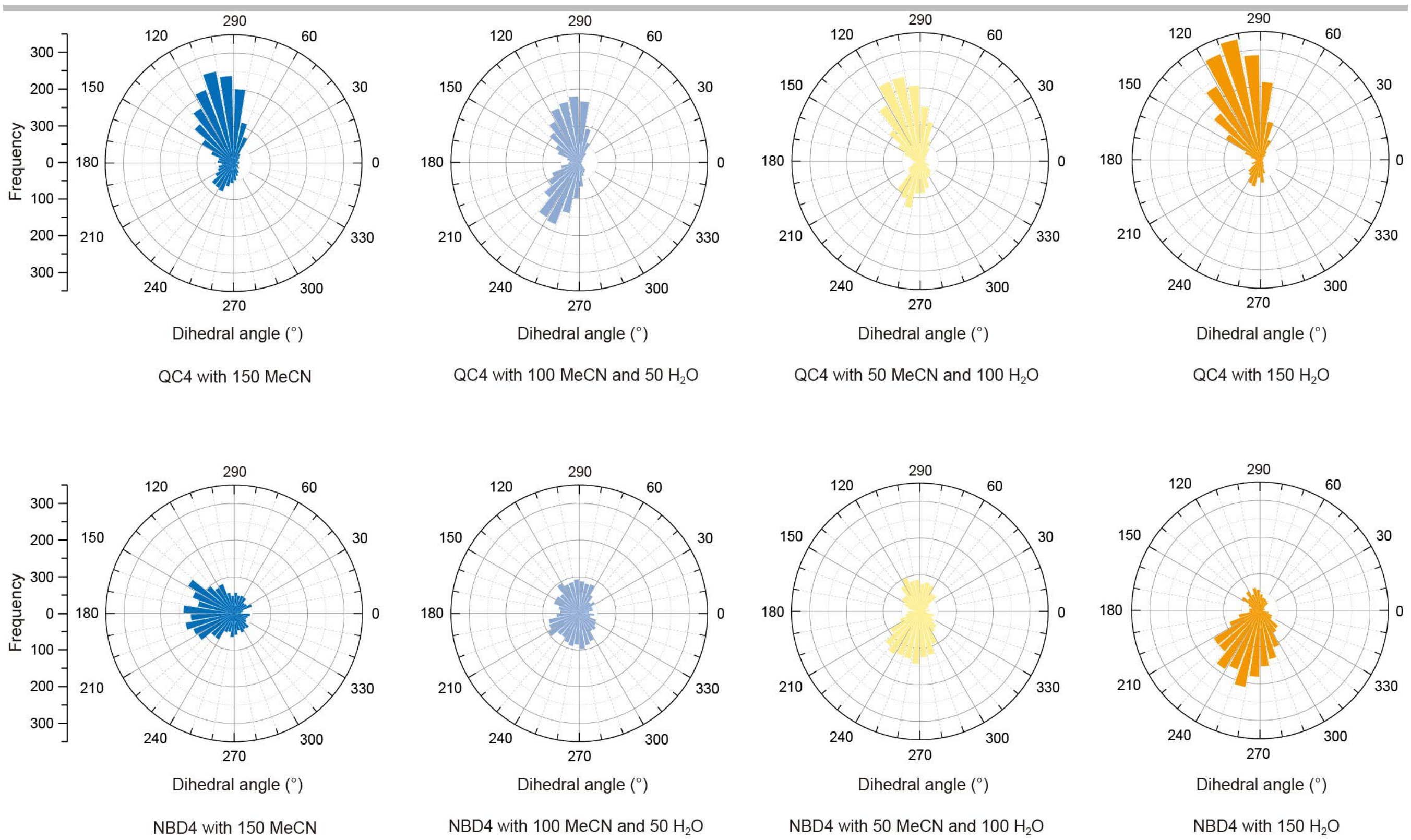

Fig. S107. Molecular dynamics and relative structures of **NBD4/QC4** at 298.15 K. The carbonyl dihedral angle was monitored across 2.5 ns trajectories under four solvent conditions: 150 explicit MeCN molecules, mixed H_2O/MeCN solutions (50 H_2O/100 MeCN and 100 H_2O/50 MeCN), and 150 explicit H_2O molecules.

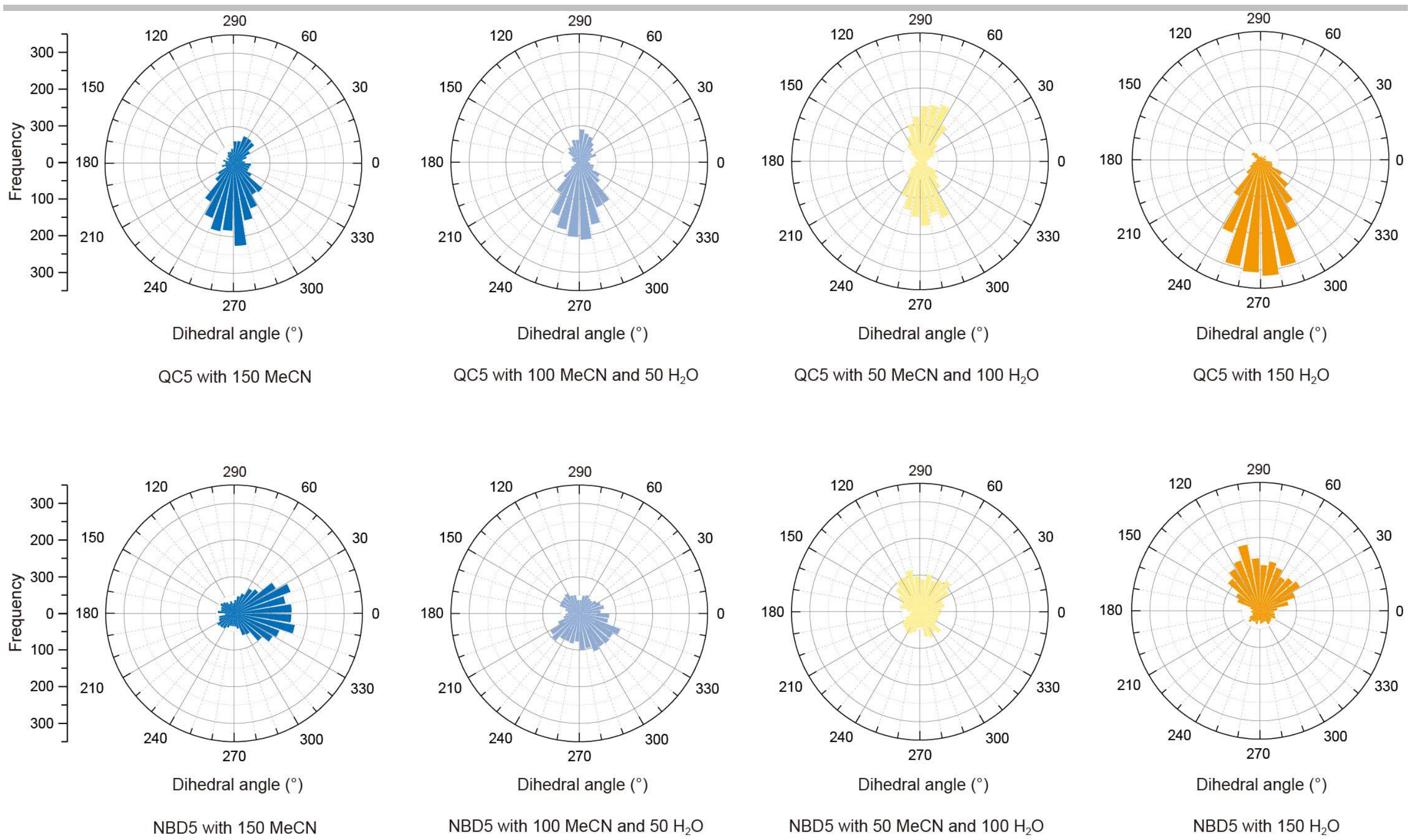

Fig. S108. Molecular dynamics and relative structures of **NBD5/QC5** at 298.15 K. The carbonyl dihedral angle was monitored across 2.5 ns trajectories under four solvent conditions: 150 explicit MeCN molecules, mixed H_2O/MeCN solutions (50 H_2O/100 MeCN and 100 H_2O/50 MeCN), and 150 explicit H_2O molecules.

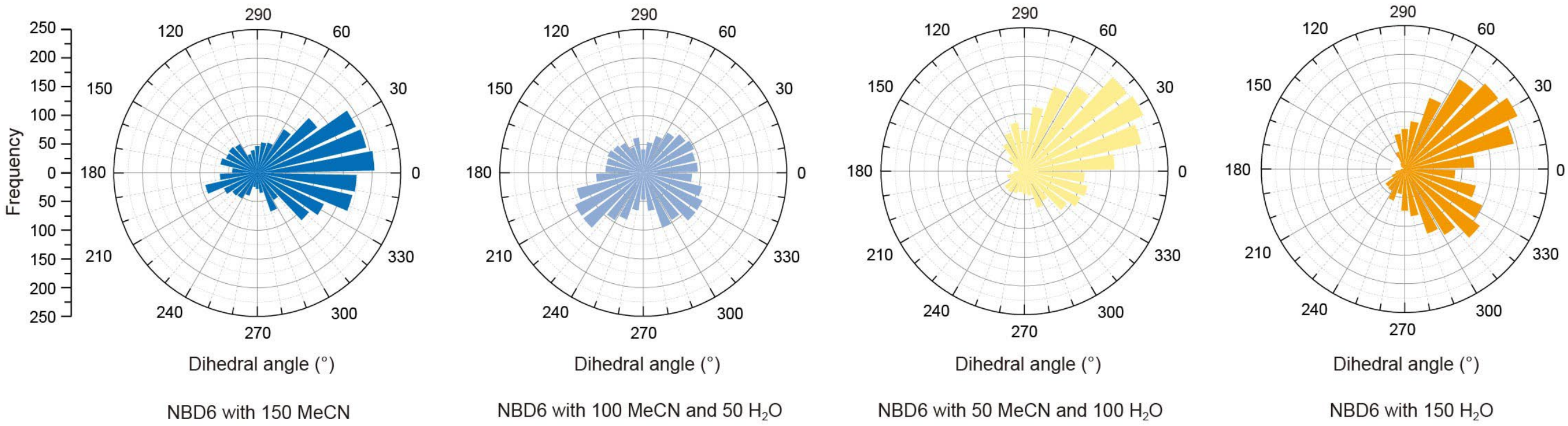

Fig. S109. Molecular dynamics and relative structures of **NBD6** at 298.15 K. The carbonyl dihedral angle was monitored across 2.5 ns trajectories under four solvent conditions: 150 explicit MeCN molecules, mixed H_2O/MeCN solutions (50 H_2O/100 MeCN and 100 H_2O/50 MeCN), and 150 explicit H_2O molecules.

References

1 Borsley, S., Leigh, D. A. & Roberts, B. M. W. Molecular Ratchets and Kinetic Asymmetry: Giving Chemistry Direction. *Angew. Chem. - Int. Ed.* **63**, e202400495 (2024).

2 Strasberg, P., Schaller, G., Brandes, T. & Esposito, M. Thermodynamics of a Physical Model Implementing a Maxwell Demon. *Phys. Rev. Lett.* **110**, 040601 (2013).

3 Minagawa, S., Mohammady, M. H., Sakai, K., Kato, K. & Buscemi, F. Universal Validity of the Second Law of Information Thermodynamics. *npj Quantum Inf.* **11**, 18 (2025).

4 Pruchyathamkorn, J. *et al.* Harnessing Maxwell's Demon to Establish a Macroscale Concentration Gradient. *Nat. Chem.* **16**, 1558-1564 (2024).

5 Cottet, N. *et al.* Observing a Quantum Maxwell Demon at Work. *Proc. Natl. Acad. Sci.* **114**, 7561-7564 (2017).

6 Sánchez, R., Splettstoesser, J. & Whitney, R. S. Nonequilibrium System as a Demon. *Phys. Rev. Lett.* **123**, 216801 (2019).

7 Jarvis, J. *et al.* Experimental Demonstration of Optical Stochastic Cooling. *Nature* **608**, 287-292 (2022).

8 Raizen, M. G. Comprehensive Control of Atomic Motion. *Science* **324**, 1403-1406 (2009).

9 Serreli, V., Lee, C.-F., Kay, E. R. & Leigh, D. A. A Molecular Information Ratchet. *Nature* **445**, 523-527 (2007).

10 Miyaura, N., Yamada, K. & Suzuki, A. A New Stereospecific Cross-Coupling by the Palladium-Catalyzed Reaction of 1-Alkenylboranes with 1-Alkenyl or 1-Alkynyl Halides. *Tetrahedron Lett.* **20**, 3437-3440 (1979).

11 Neese, F. Software Update: The Orca Program System-Version 6.0. *WIREs Comput. Mol. Sci.* **15** (2025).

12 Neese, F., Wennmohs, F., Becker, U. & Riplinger, C. The Orca Quantum Chemistry Program Package. *J. Chem. Phys.* **152** (2020).

13 Bannwarth, C. *et al.* Extendedtight-Bindingquantum Chemistry Methods. *WIREs Comput. Mol. Sci.* **11**, e1493 (2021).

14 Bannwarth, C., Ehlert, S. & Grimme, S. Gfn2-Xtb—an Accurate and Broadly Parametrized Self-Consistent Tight-Binding Quantum Chemical Method with Multipole Electrostatics and Density-Dependent Dispersion Contributions. *J. Chem. Theory Comput.* **15**, 1652-1671 (2019).

15 de Souza, B. Goat: A Global Optimization Algorithm for Molecules and Atomic Clusters. *Angew. Chem. - Int. Ed.* **64**, e202500393 (2025).

16 Henkelman, G., Uberuaga, B. P. & Jónsson, H. A Climbing Image Nudged Elastic Band Method for Finding Saddle Points and Minimum Energy Paths. *J. Chem. Phys.* **113**, 9901-9904 (2000).

17 Caldeweyher, E. *et al.* A Generally Applicable Atomic-Charge Dependent London Dispersion Correction. *J. Chem. Phys.* **150**, 154122 (2019).

18 Chai, J.-D. & Head-Gordon, M. Long-Range Corrected Hybrid Density Functionals with Damped Atom–Atom Dispersion Corrections. *Phys. Chem. Chem. Phys.* **10**, 6615-6620 (2008).

19 Dunning, T. H. Gaussian-Basis Sets for Use in Correlated Molecular Calculations .1. The Atoms Boron through Neon and Hydrogen. *J. Chem. Phys.* **90**, 1007-1023 (1989).

20 Ryckaert, J.-P., Ciccotti, G. & Berendsen, H. J. C. Numerical Integration of the Cartesian Equations of Motion of a System with Constraints: Molecular Dynamics of N-Alkanes. *J. Comput. Phys.* **23**, 327-341 (1977).

21 Elholm, J. L. *et al.* An Automated Photo-Isomerisation and Kinetics Characterisation System for Molecular Photoswitches. *Digit. Discov.* **4**, 2045-2051 (2025).

22 Gagliardi, L. G., Castells, C. B., Ràfols, C., Rosés, M. & Bosch, E. Static Dielectric Constants of Acetonitrile/Water Mixtures at Different Temperatures and Debye−Hückel a and A0b Parameters for Activity Coefficients. *J. Chem. Eng. Data* **52**, 1103-1107 (2007).

23 Рудаков, O., Rudakova, L. & Selemenev, V. Acetonitrile as Tops Solvent for Liquid Chromatography and Extraction. *J. Anal. Chromatogr. Spectrosc.* **1**, 1-19 (2018).

24 Jevric, M. *et al.* Norbornadiene-Based Photoswitches with Exceptional Combination of Solar Spectrum Match and Long-Term Energy Storage. *Chem. Eur. J.* **24**, 12767-12772 (2018).

25 Quant, M., Hamrin, A., Lennartson, A., Erhart, P. & Moth-Poulsen, K. Solvent Effects on the Absorption Profile, Kinetic Stability, and Photoisomerization Process of the Norbornadiene–Quadricyclanes System. *J. Phys. Chem. C* **123**, 7081-7087 (2019).

26 Kennedy, A. D. W., Sandler, I., Andréasson, J., Ho, J. & Beves, J. E. Visible-Light Photoswitching by Azobenzazoles. *Chem. Eur. J.* **26**, 1103-1110 (2020).

27 Lentes, P. *et al.* Nitrogen Bridged Diazocines: Photochromes Switching within the near-Infrared Region with High Quantum Yields in Organic Solvents and in Water. *J. Am. Chem. Soc.* **141**, 13592-13600 (2019).

28 Ewert, J. *et al.* Photoswitchable Diazocine-Based Estrogen Receptor Agonists: Stabilization of the Active Form inside the Receptor. *J. Am. Chem. Soc.* **144**, 15059-15071 (2022).

29 Zhang, X. *et al.* Azothiophene-Based Molecular Switches: Influence of Substituent Position and Solvent Environment on Photophysical Behavior. *Phys. Chem. Chem. Phys.* **28**, 1173-1188 (2026).

30 Piskorz, K., Dust, J. M., Buncel, E., Lebel, O. & Nunzi, J.-M. Deceleration of Thermal Ring Closure in a Glass-Forming Mexylaminotriazine-Substituted Merocyanine (Mc) Linked to Intramolecular Hydrogen Bonding. *New J. Chemistry* **41**, 940-947 (2017).

31 Seshadri, S. *et al.* Self-Regulating Photochemical Rayleigh-Bénard Convection Using a Highly-Absorbing Organic Photoswitch. *Nat. Commun.* **11**, 2599 (2020).

32 Brittain, W., Barrett, S., Mouer, L. & Sauceda, N. A Transient Phenolate Enables Relaxation of Z-Tetra-Ortho-Fluoroazobenzene to the Stable State in Seconds. *Angew. Chem. - Int. Ed.* **64**, e202513719 (2025).
