# Supplementary material for: Orchestrating photoswitch thermal backconversion using hydrogen bonds to reshape energy landscapes
Source: Nat Commun. 2026 Aug 22;17:10063. doi: 10.1038/s41467-026-76898-2 (PMC13594139; doi:10.1038/s41467-026-76898-2)
Supplement: Supplementary file 2 [file 41467_2026_76898_MOESM2_ESM.pdf]

Description of Additional Supplementary Files

File Name: Supplementary Movie 1
Description: Backconversion process from **QC6** to **NBD6**.

File Name: Supplementary Movie 2
Description: Dihedral angles and energy landscapes in **QC1**.

File Name: Supplementary Movie 3
Description: Dihedral angles and energy landscapes in **QC6**.
