# Supplementary material for: Orchestrating photoswitch thermal backconversion using hydrogen bonds to reshape energy landscapes
Source: Nat Commun. 2026 Aug 22;17:10063. doi: 10.1038/s41467-026-76898-2 (PMC13594139; doi:10.1038/s41467-026-76898-2)
Supplement: Supplementary file 3 [file 41467_2026_76898_MOESM6_ESM.pdf]

nature portfolio

Peer Review File

Orchestrating Photoswitch Thermal Backconversion using Hydrogen Bonds to Reshape Energy Landscapes

Corresponding Author: Professor Kasper Moth-Poulsen

This file contains all reviewer reports in order by version, followed by all author rebuttals in order by version.

Version 0:

Reviewer comments:

Reviewer #1

(Remarks to the Author)
The present work modifies the NBD scaffold with hydrogen-bond donor/acceptor motifs so that the photoisomerized product, QC, can interact with water clusters to form a more stabilized structure, thereby extending the thermal half-life of the metastable state. The molecules are novel, and the molecular design concept is interesting. The manuscript is generally well written, with a clear narrative flow and an engaging conceptual analogy to Maxwell's demon (thought it is not the same, strickly speaking). Overall, I recommend acceptance after minor revision.

Major:
1. To strengthen the perspective of this work, the conclusion can highlight broader implications and alternative stabilization strategies for the NBD/QC system. In addition to the intramolecular interactions explored here, other non covalent interactions, such as coordination with small anions (e.g., fluoride) or reversible covalent motifs (e.g., disulfide linkages), could, in principle, provide additional stabilization of the QC form and influence the NBD->QC equilibrium. While such approaches fall outside the scope of the present design, they illustrate that the concept of tuning the energy landscape through specific, switchable interactions may be generalizable. Looking beyond solution environments, embedding NBD/QC photoswitches in solid matrices or polymeric frameworks could further modulate both thermal and photochemical pathways, potentially enhancing energy storage lifetimes or enabling cooperative effects through the matrix. Similarly, excited state mechanisms distinct from those investigated here (e.g., involving triplet sensitization or vibronic coupling pathways) may offer alternative routes to improve photoisomerization efficiency. For the discussion, I encourage authors to cite the following reference, "doi.org/10.1038/s41467-022-28489-0". Brief consideration of these possibilities underscores the versatility of the current design concept and suggests avenues for future exploration.

2. The current presentation of Fig. 5c is somewhat vague. I suggest moving part of Fig. S84 into the main text to provide clearer structural insight. Ideally, the authors could present the key conformations of NBD/QC6 (in vacuum and/or in water), including inward and outward geometries. 8 structures, together with their relative energies (referenced to a single energy point) and the corresponding dihedral angles, would help readers better understand the energy landscape.

3. The involvement of water clusters sometimes suggests that quantum tunneling could influence the thermal back-conversion process. It may therefore be informative to test one representative case of QC recovery in D2O/ACN and compare the results with those obtained in H2O/ACN. Such an experiment could help evaluate whether a kinetic isotope effect (KIE) is present, which would provide additional insight.

Minor:
1. In the main text, "requiring rotations of up to 180° to escape this water-locked geometry before reaching the transition state (Fig. 1c, Table S25, Movie S2)." Should be Fig. 5c?

2. Is it possible to obtain a single crystal of QC6 from ACN/H_2O, for example by slow evaporation of ACN followed by gradual moisture diffusion? If feasible, such structural information could further support the proposed water-cluster interactions.

Reviewer #2

(Remarks to the Author)
The authors present a relevant study on the influence of hydrogen bonds – as established by gradual increase of water content in the reaction medium – on the back reaction of a photochromic equilibrium. For that purpose, they wisely chose the robust norbornadiene-quadricyclane system and attached varying substituents with different propensity to form hydrogen bonds. In extensive and systematic physico-chemical studies it is demonstrated convincingly that with hydrogen-bonding substituents the rate of conversion of quadricyclane is strongly decelerated with increasing content of water in the solution. In addition, it is shown by detailed spectroscopic and theoretical analysis that this effect is caused by hydrogen-bonded bridging water molecule between the substituents which imposes structural rigidity and thus slows down the reaction. This part of the study alone is already a highly relevant contribution to the field of functional photochromic systems as it provides a paradigm how to employ hydrogen bonds to control the reactivity of a metastable state.
In the second part of this project, it is shown that the influence of H-bonds on the reactivity of quadricyclanes may be employed in a setup to mimic Maxwell's Demon thought experiment. It is shown how the controlled gradual addition or removal of water molecules and the resulting increase or decrease of the reaction rate can be linked in an arrangement of "boxes" with different controllable states, thus providing a valid example for this theoretical model.
The manuscript is well written and the results and discussion are presented in a concise and convincing way. The experimental work is well conducted; and the resulting data are solid and justify the conclusion. The experimental material is sufficiently documented in the Supporting Information.
Overall, this is a highly relevant, high-quality contribution to a topical research field, which is also of very high interest for a broad scientific readership. Therefore, I recommend publication in Nature Communications. However, the following comments and suggestions should be considered in a minor revision:

1. The discussion of the effect of hydrogen bonding is mainly focused on the association of only one water molecule to the functionalities of the quadricyclane. Can it be excluded that the situation is a bit more complicated? In particular, more water molecules may be involved, not necessarily in the primary hydrogen bond, but potentially as a specifically structured second water shell around this hydrogen bonding unit, providing significant thermodynamic contribution to the system.

2. The addition of water to acetonitrile not only changes the H-bonding properties of the medium but also other solvent parameters, such as viscosity, density, refractive index, dielectric constant, etc. and the temperature dependence thereof, all of which may also have an influence on the reaction rate. This aspect should be considered and discussed in more detail, especially as water/acetonitrile mixtures have been investigated along these lines already.

3. It is known that H bonding at carbonyl functionalities may lower HOMO/LUMO energies. Does that also play a role in this case?

4. Experimental
– Compound characterization: Exact mass deviates in some cases quite a bit from the theoretical value. Please indicate the intrinsic error margin/standard deviation of the analysis and the acceptable difference between experimental and theoretical value.
– Please give solvent in caption of NMR spectra in SI.
– Would it be possible to determine water content of the solution in an independent analysis (e.g. by Karl-Fischer titration)?

5. Format
– In chemical structures, substituents R should not be assigned by subscript numbers, because this position is reserved for the number of the substituents (I know that this is a ChemDraw default, but it contradicts formalism in Organic Chemistry). Please place the index superscript instead.
– page 3: change "information carriers information carriers" to " information carriers"
– page 9, last line: change " experiemental" to " experimental"

Reviewer #3

(Remarks to the Author)
In this work, the authors present a supramolecular strategy to control the thermal back-conversion of the norbornadiene/quadricyclane (NBD/QC) photoswitch pair by using hydrogen-bond donors and acceptors that recruit water molecules as a reversible "bridge." The authors frame this as a molecular analogue of Maxwell's demon: the water-mediated hydrogen-bond network selectively locks certain QC conformations, reshapes the rotational energy landscape, raises the activation barrier for QC-to-NBD back-isomerization, and can even redirect the reaction pathway. Experimentally, it is shown that compounds with suitable hydrogen-bonding motifs, especially QC6, exhibit dramatically slowed thermal back-conversion, with the half-life extended by about 5000-fold upon aqueous solution conditions, while compounds lacking the necessary hydrogen-bonding architecture show little effect. NMR, Raman, DFT, and molecular dynamics analyses support the conclusion that reversible water-bridged hydrogen bonding is responsible for this tunable kinetic gating. Overall, the authors propose a method to control thermal isomerization kinetics in molecular photoswitches via hydrogen bond interactions with solvent.

However, in my view, the conceptual novelty of this work remains limited, as it primarily concerns solvent control of reaction barriers, which is not in itself a new concept. Solvent effects, particularly hydrogen-bonding interactions, have long been known to modulate reaction barriers. Accordingly, the observation of hydrogen-bond-controlled thermal isomerization rates in this study does not, by itself, constitute a fundamentally new finding. I also find the framing of this phenomenon as a realization of Maxwell's demon to be misleading. In the original thought experiment, Maxwell's demon appears to reduce

entropy without directly performing work on the system. That analogy does not convincingly apply here. In the present case, adding water to the solvent introduces isomer-dependent, species-specific hydrogen-bonding interactions that directly alter the enthalpy of the molecular system. The resulting energetic stabilization of certain QC derivatives by water is therefore the direct origin of the increased isomerization barrier. If an analogy has to be made to the Maxwell's demon in this case, then the demon actually slow down some of the moving particles in the though experiment, amounting to doing work to the system. In this sense, the phenomenon is more appropriately understood as a conventional solvation effect, namely a lowering of the reactant-state enthalpy through selective solute-solvent interactions, rather than as a genuine Maxwell's demon mechanism.

Overall, I find the Maxwell's demon interpretation superficial, as the central effect is essentially solvent-induced modulation of the reaction energy landscape. More broadly, a substantial body of prior work has already examined how the molecular environment influences the kinetics of photochemical and ground-state reactions of photoswitches, including phenomena such as aggregation-induced emission and turn-on fluorescence. Therefore, although this study presents a useful strategy for tuning the thermal half-lives of photoswitches without strongly perturbing their absorption wavelengths, I do not believe its conceptual novelty meets the level typically expected for publication in Nature Communications.

Version 1:

Reviewer comments:

Reviewer #1

(Remarks to the Author)
I have carefully examined the reply letter as well as the revised manuscript. The authors have addressed all of my concerns, and the manuscript has been revised appropriately. Therefore, I recommend "acceptance" in its current form.

Reviewer #2

(Remarks to the Author)
The revision led to a coherent and scientifically very sound manuscript. The authors provided a properly and thoroughly revised paper that is now in acceptable form to be published in Nature Communications. In the rebuttal, the authors discuss extensively the critical points raised by the reviewers, and they explain convincingly the resulting changes in the manuscript. The additional experimental and theoretical data as well as the clarification in the main text mostly toned down the concerns of the reviewers. At least for my part, I am fully satisfied with the response to the comments of my original evaluation report. My very positive assessment of the quality, relevance, and importance of this study has not changed, so that I recommend publication of this fine manuscript as it is.

Reviewer #3

(Remarks to the Author)
The authors have addressed all of my comments.

Open Access This Peer Review File is licensed under a Creative Commons Attribution 4.0 International License, which permits use, sharing, adaptation, distribution and reproduction in any medium or format, as long as you give appropriate credit to the original author(s) and the source, provide a link to the Creative Commons license, and indicate if changes were made.
In cases where reviewers are anonymous, credit should be given to 'Anonymous Referee' and the source.
The images or other third party material in this Peer Review File are included in the article's Creative Commons license, unless indicated otherwise in a credit line to the material. If material is not included in the article's Creative Commons license and your intended use is not permitted by statutory regulation or exceeds the permitted use, you will need to obtain permission directly from the copyright holder.
To view a copy of this license, visit https://creativecommons.org/licenses/by/4.0/

Reply to Reviewer: 1

Reviewer 1: The present work modifies the NBD scaffold with hydrogen-bond donor/acceptor motifs so that the photoisomerized product, QC, can interact with water clusters to form a more stabilized structure, thereby extending the thermal half-life of the metastable state. The molecules are novel, and the molecular design concept is interesting. The manuscript is generally well written, with a clear narrative flow and an engaging conceptual analogy to Maxwell's demon (thought it is not the same, strickly speaking). Overall, I recommend acceptance after minor revision.

We thank the reviewer for improving the manuscript and valuing our work in thermal dynamic control by hydrogen-bond system. The answers to the comments are revised below.

1. To strengthen the perspective of this work, the conclusion can highlight broader implications and alternative stabilization strategies for the NBD/QC system. In addition to the intramolecular interactions explored here, other non covalent interactions, such as coordination with small anions (e.g., fluoride) or reversible covalent motifs (e.g., disulfide linkages), could, in principle, provide additional stabilization of the QC form and influence the NBD->QC equilibrium. While such approaches fall outside the scope of the present design, they illustrate that the concept of tuning the energy landscape through specific, switchable interactions may be generalizable. Looking beyond solution environments, embedding NBD/QC photoswitches in solid matrices or polymeric frameworks could further modulate both thermal and photochemical pathways, potentially enhancing energy storage lifetimes or enabling cooperative effects through the matrix. Similarly, excited state mechanisms distinct from those investigated here (e.g., involving triplet sensitization or vibronic coupling pathways) may offer alternative routes to improve photoisomerization efficiency. For the discussion, I encourage authors to cite the following reference, "doi.org/10.1038/s41467-022-28489-0". Brief consideration of these possibilities underscores the versatility of the current design concept and suggests avenues for future exploration.

Response 1: Thank you for your valuable advice. We have revised the conclusion to emphasize broader implications and potential alternative stabilization strategies for the NBD/QC system. Also, the reference has been cited, see in ref.24.

To clarify this, we have revised the conclusion as follows.

In Conclusion

Remarkably, **QC6**-water interactions prolong the thermal half-life by over 5000-fold compared to the anhydrous form, an effect attributed to multiple hydrogen bonds imposing steric and enthalpic constraints at the transition state. This strategy could be extended beyond intramolecular hydrogen bonding to other switchable interactions, such as small-anion coordination or reversible covalent motifs, offering new ways to tune the energy landscape. Complementary to such ground-state stabilization strategies, excited-state approaches, including triplet sensitization or vibronic-coupling pathways,[24] may provide orthogonal means to enhance photoisomerization efficiency and further optimize photoswitch performance. Beyond solution-phase systems,

embedding such photoswitches in solid matrices or supramolecular frameworks could further enhance thermal stability and enable cooperative effects.

2. The current presentation of Fig. 5c is somewhat vague. I suggest moving part of Fig. S84 into the main text to provide clearer structural insight. Ideally, the authors could present the key conformations of NBD/QC6 (in vacuum and/or in water), including inward and outward geometries. 8 structures, together with their relative energies (referenced to a single energy point) and the corresponding dihedral angles, would help readers better understand the energy landscape.

Response 2: We agree with your suggestions. Figure S86 has been moved to the main text as Extended Data Fig. 3, where the inward and outward geometries of NBD6 and QC6 are presented in vacuum, in the presence of six H_2O molecules, and with six MeCN molecules. In addition, Fig. 5c has been updated to include both the rotational angles and the corresponding energies along the QC-to-NBD pathway, highlighting that the conversion requires an approximately 180° rotation.

To further improve clarity, we have also supplemented Fig. S91 with a single-energy-point representation, enabling a more direct comparison between the relative energies and their corresponding dihedral angles.

To clarify this, we have rewritten the sentence and revised the Fig. 5c, Extended Data Fig. 3, and Fig. S91 as follows.

Page 12

Electrostatic potential maps reveal that the **NBD6**/**QC6** pair localizes stronger negative charge on the ester group and stronger positive charge on the amide group (Extended Data **Fig. 4**, **Fig. S86**), creating favorable sites for water-bridge formation. Water selectively stabilizes inward conformers (~350° and 0-30°), such that escape from this water-locked geometry requires rotational reorganization of up to 180° prior to reaching the transition state. (**Fig. 5**c, **S91**, **Table S28**, **Movie S2**).

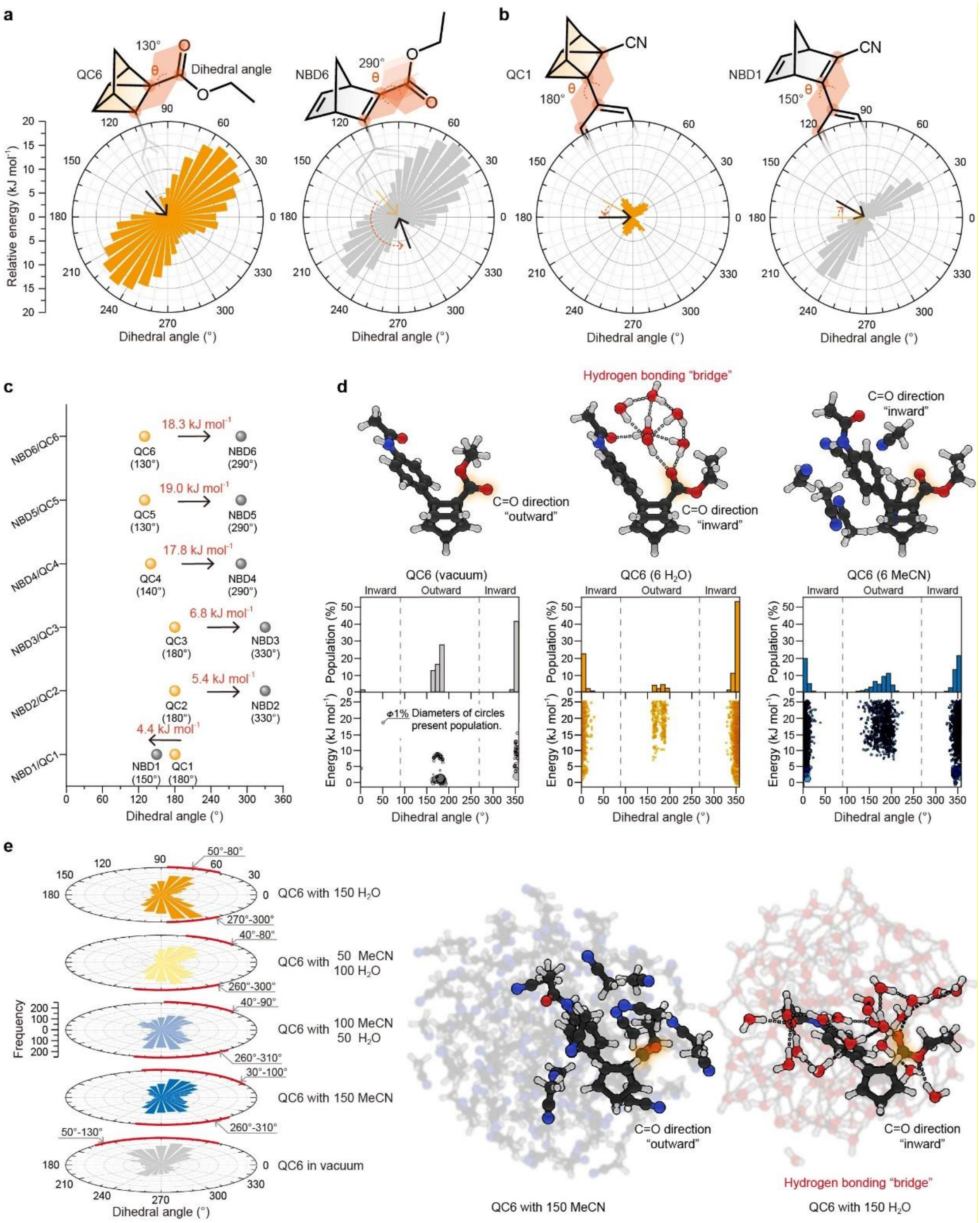

Fig. 5. Simulations of NBD/QC pairs. Dihedral angles and energy landscapes in **a NBD6/QC6** and **b NBD1/QC1** pairs. **c,** Rotational angles and the corresponding energies along the dihedral angle scans. The annotated angles and energy values correspond to the minimum- and maximum-energy points identified during the dihedral angle scanning process, respectively. **d,** Inward and outward orientation population and energy landscapes of **QC6** in vacuum, with six H_2O molecules, and with six MeCN molecules. **e,** Molecular dynamics and relative structures of **QC6** at 298.15 K. The carbonyl dihedral angle was monitored across 2.5 ns trajectories under five solvent conditions: pure vacuum, 150 explicit MeCN molecules, mixed H_2O/MeCN solutions (50 H_2O/100 MeCN and 100 H_2O/50 MeCN), and 150 explicit H_2O molecules.

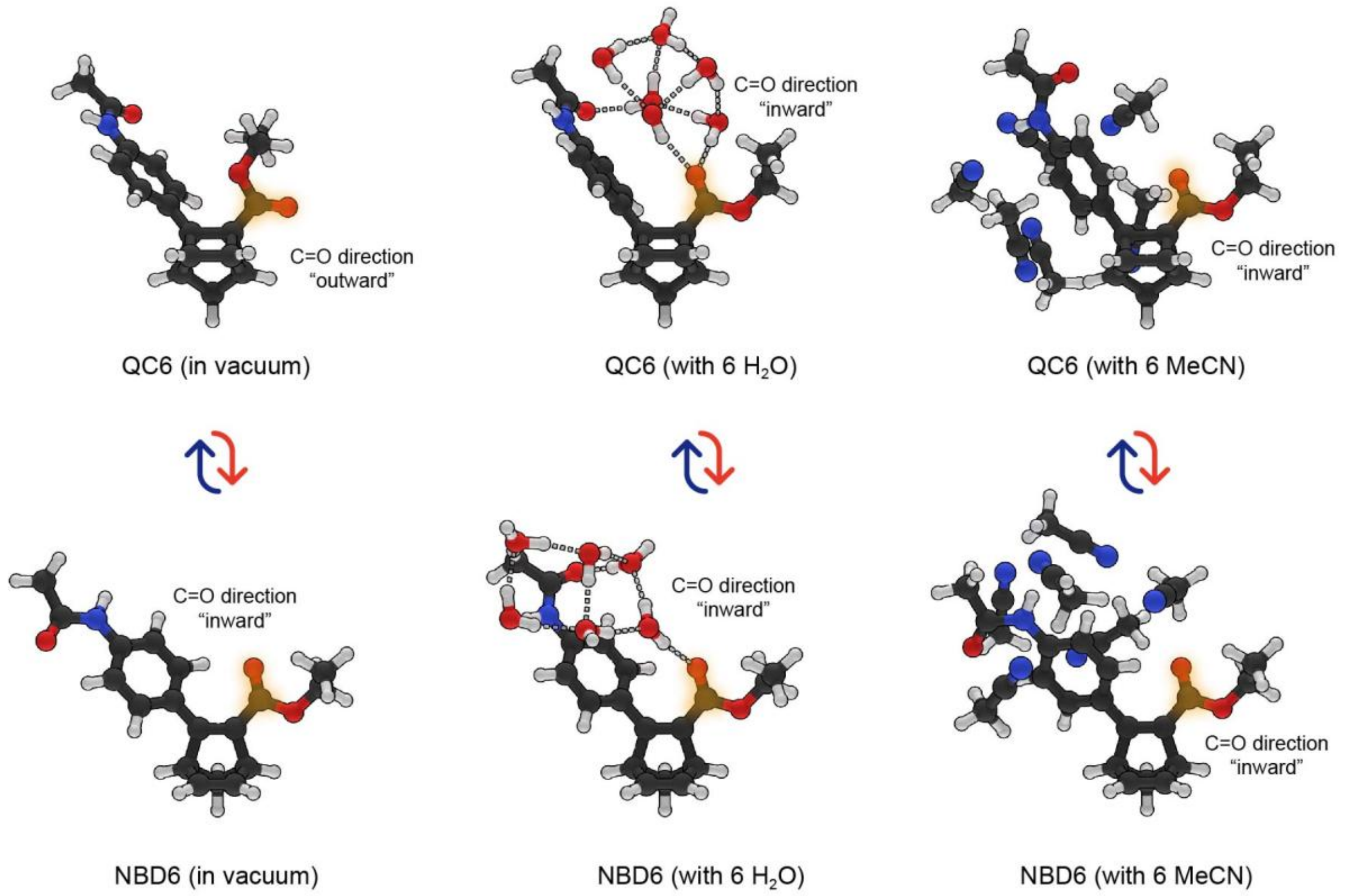

Extended Data Fig. 3. Optimized structures of **NBD6** and **QC6** in vacuum, with six H_2O molecules, and with six MeCN molecules.

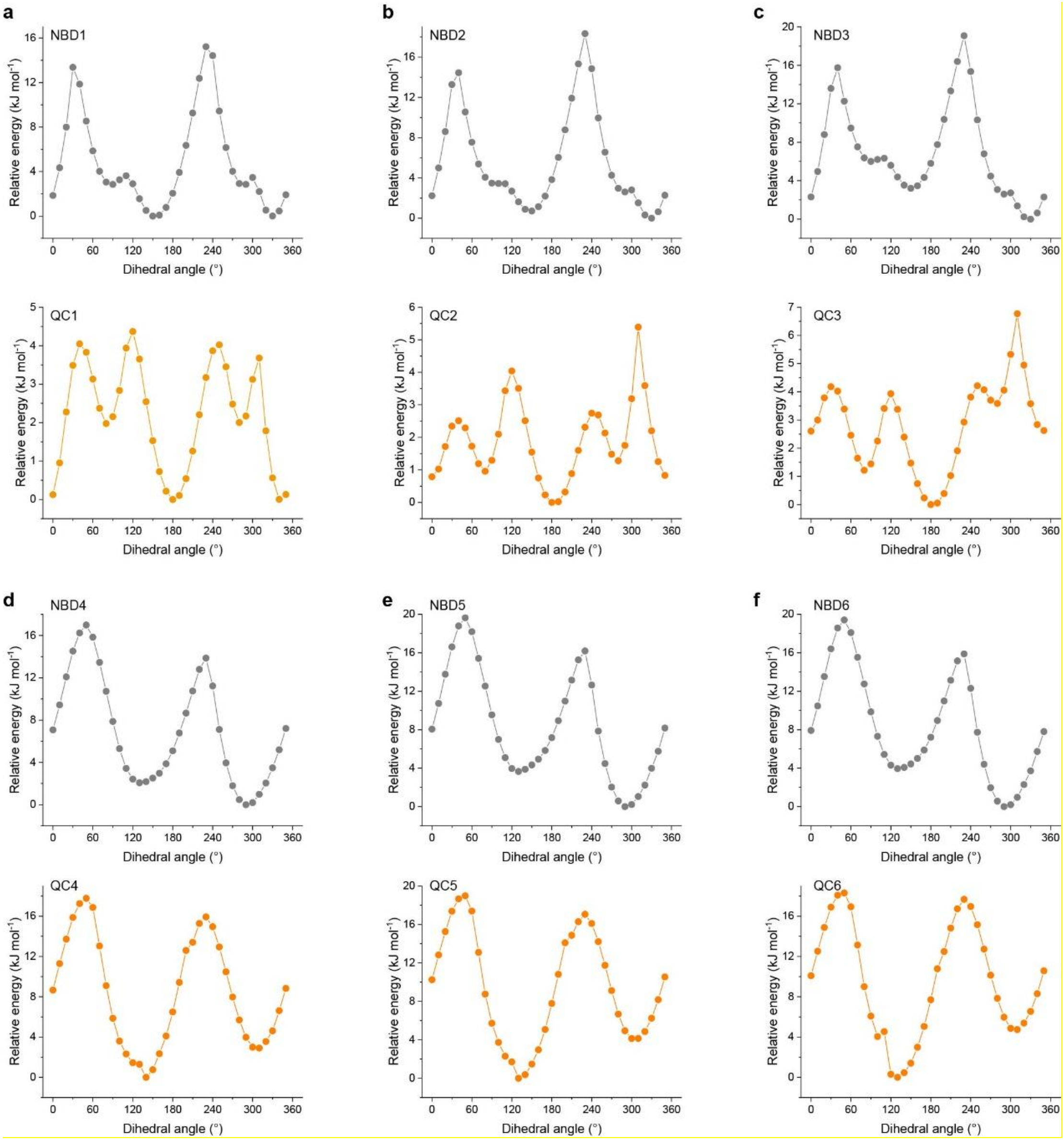

Fig. S91. Dihedral angles and energy landscapes of all NBD/QCs.

3. The involvement of water clusters sometimes suggests that quantum tunneling could influence the thermal back-conversion process. It may therefore be informative to test one representative case of QC recovery in D2O/ACN and compare the results with those obtained in H2O/ACN. Such an experiment could help evaluate whether a kinetic isotope effect (KIE) is present, which would provide additional insight.

Response 3: Thank you for this valuable suggestion. We performed additional control experiments by comparing the thermal back-conversion behavior of QC6 in H_2O/ACN and D_2O/ACN mixtures (1:20). As shown in the Figs. R1, R2 and Table R1 as below, the back-conversion kinetics (half-life at 25 °C is 8699 days) in D_2O/ACN were nearly identical to those observed in H_2O/ACN, and no significant kinetic isotope effect was detected within the experimental uncertainty. These results suggest that proton transfer or quantum tunneling processes are unlikely to play a dominant role in the thermal back-conversion mechanism. Instead, the observed kinetic control is more consistent with a hydrogen-bond effect, rather than a tunneling-controlled process.

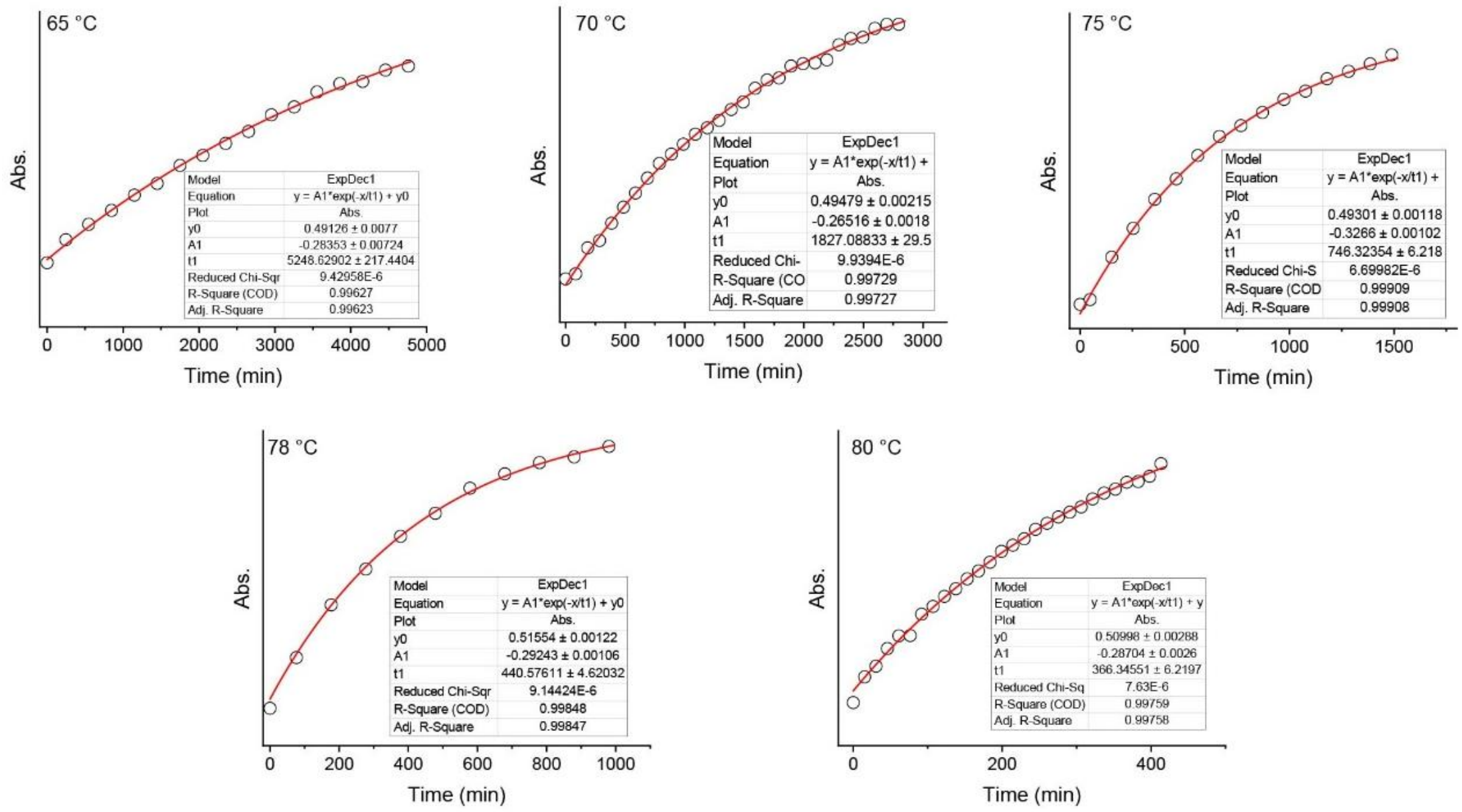

Fig. R1. Thermal back conversion of NBD6 in MeCN (0.1 mM) with NBD-to-D_2O molar ratio of 1:20 at 65, 70, 75, 78, 80 °C. All the curves are fitted by first-order kinetics equation.

Table R1. Thermal backconversion parameters of NBD6 in MeCN (0.1 mM) with NBD-to-D_2O molar ratio of 1:20.					
Backconversion rate	Temperature (°C)				
	65	70	75	78	80
k ($\times 10^{-3}$ min^{-1})	0.191	0.547	1.34	2.27	2.72

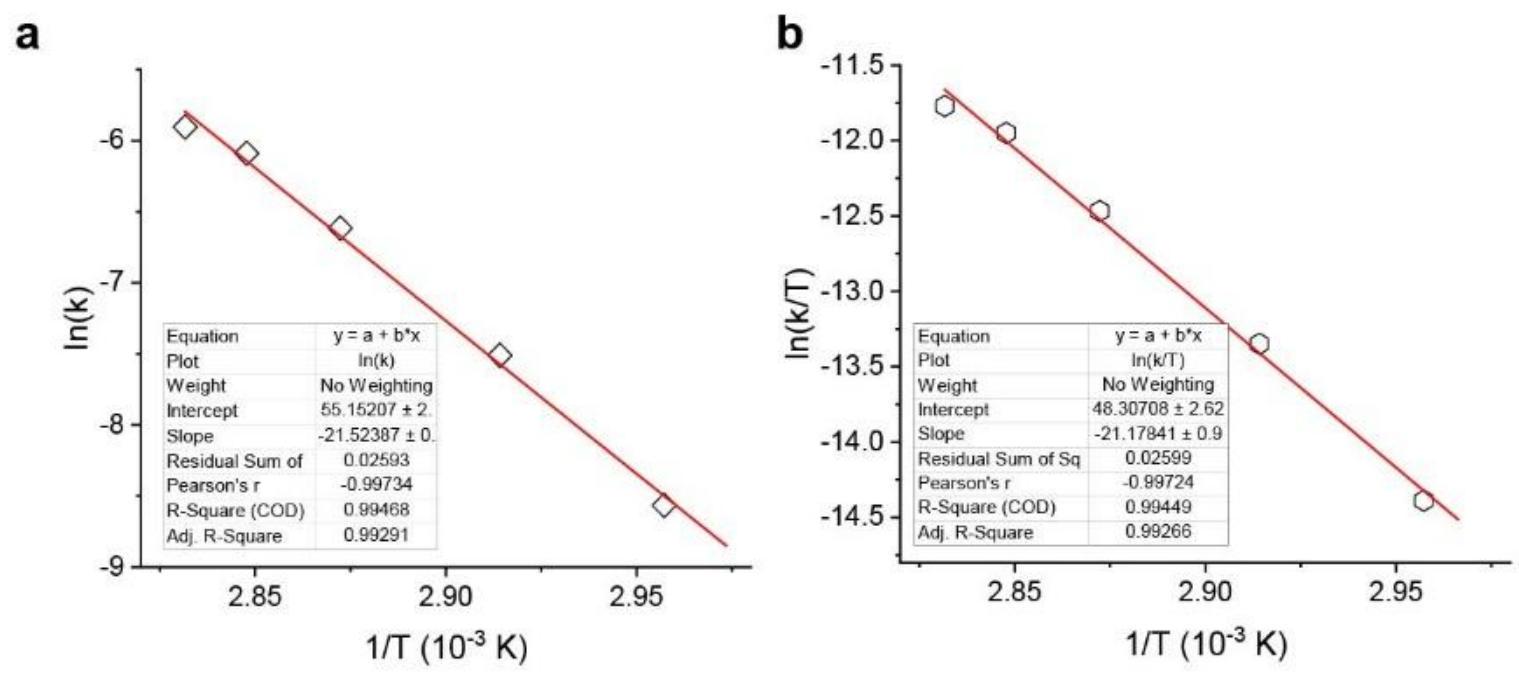

Fig. R2. **a**, Arrhenius plot of NBD6. **b**, Eyring plot of NBD6.

4. In the main text, “requiring rotations of up to 180° to escape this water-locked geometry before reaching the transition state (Fig. 1c, Table S25, Movie S2).” Should be Fig. 5c?

Response 4: We have revised this mistake in the main text.

5. Is it possible to obtain a single crystal of QC6 from ACN/H_2O, for example by slow evaporation of ACN followed by gradual moisture diffusion? If feasible, such structural information could further support the proposed water-cluster interactions.

Response 5: That's a good suggestion. We attempted to obtain single crystals of QC6 from ACN/H_2O mixtures by slow evaporation of ACN followed by gradual moisture diffusion. However, no single crystals were obtained, likely due to the nonplanar and asymmetric structure of QC (see Figure R1 below). Slow evaporation of ACN resulted in a liquid sample, while slow evaporation of a 1:15 ethyl acetate/hexane mixture produced only solid powders rather than single crystals.

Method for single-crystal preparation.

Attempts to grow single crystals of QC6 were carried out by slow evaporation of QC6 solutions in 1:15 (v/v) ethyl acetate/hexane or pure ACN at room temperature. In addition, QC6 samples with different water ratios (ACN/H_2O = 1:1, 1:5, and 1:20) were prepared and subjected to slow evaporation at room temperature.

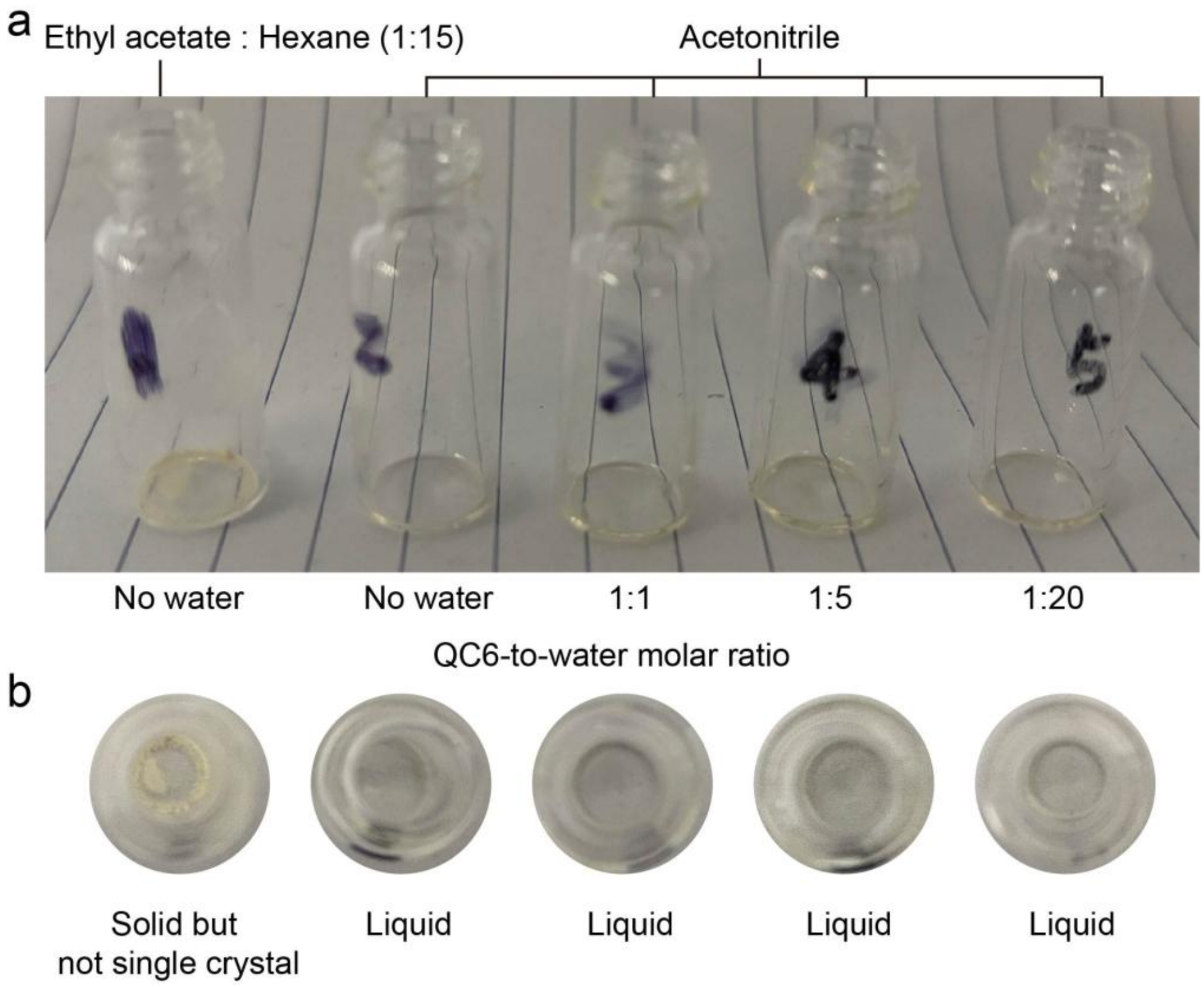

Figure R1. **a**, Front view and **b**, top view of the vials showing the phase states obtained following the single-crystal growth procedure.

Reply to Reviewer: 2

The authors present a relevant study on the influence of hydrogen bonds – as established by gradual increase of water content in the reaction medium – on the back reaction of a photochromic equilibrium. For that purpose, they wisely chose the robust norbornadiene-quadricyclane system and attached varying substituents with different propensity to form hydrogen bonds. In extensive and systematic physico-chemical studies it is demonstrated convincingly that with hydrogen-bonding substituents the rate of conversion of quadricyclane is strongly decelerated with increasing content of water in the solution. In addition, it is shown by detailed spectroscopic and theoretical analysis that this effect is caused by hydrogen-bonded bridging water molecule between the substituents which imposes structural rigidity and thus slows down the reaction. This part of the study alone is already a highly relevant contribution to the field of functional photochromic systems as it provides a paradigm how to employ hydrogen bonds to control the reactivity of a metastable state.

In the second part of this project, it is shown that the influence of H-bonds on the reactivity of quadricyclanes may be employed in a setup to mimic Maxwell's Demon thought experiment. It is shown how the controlled gradual addition or removal of water molecules and the resulting increase or decrease of the reaction rate can be linked in an arrangement of "boxes" with different controllable states, thus providing a valid example for this theoretical model.

The manuscript is well written and the results and discussion are presented in a concise and convincing way. The experimental work is well conducted; and the resulting data are solid and justify the conclusion. The experimental material is sufficiently documented in the Supporting Information.

Overall, this is a highly relevant, high-quality contribution to a topical research field, which is also of very high interest for a broad scientific readership. Therefore, I recommend publication in Nature Communications. However, the following comments and suggestions should be considered in a minor revision:

We thank him/her for the many valuable suggestions to improve the quality of the manuscript. The answers to the comments are revised below.

1. The discussion of the effect of hydrogen bonding is mainly focused on the association of only one water molecule to the functionalities of the quadricyclane. Can it be excluded that the situation is a bit more complicated? In particular, more water molecules may be involved, not necessarily in the primary hydrogen bond, but potentially as a specifically structured second water shell around this hydrogen bonding unit, providing significant thermodynamic contribution to the system.

Response 1: We agree with the reviewer's comment. A series of experiments with varying NBD-to-water molar ratios (e.g., 2:1, 1:1, 2:3, 1:2, 1:5, 1:10, and 1:20) were conducted to probe the thermodynamic behavior. For instance, QC3-6 exhibited a gradual increase in $t_{1/2}$ with increasing water:NBD ratios (see Fig. 3c). In addition, we supplemented an experiment with QC6 at a higher water ratio of 1:200, where the back-conversion rate showed no further change compared to that at 1:20 (see Figs. S61, 62, and Table S18). These observations suggest that more than a single water molecule interacts with the functional groups of the quadricyclane. Instead, water may

form clusters or a more structured secondary solvation shell around the hydrogen-bonding sites as reviewer mentioned. To further investigate this, we carried out molecular dynamics simulations under different conditions: vacuum, 150 explicit MeCN molecules, mixed H_2O/MeCN systems (50 H_2O /100 MeCN and 100 H_2O/50 MeCN), and 150 explicit H_2O molecules (see Fig. 5e and Figs. S96-102), mimicking various solvation-shell environments. In these cases, the bimodal distribution becomes significantly narrower, indicating stabilization of two distinct water-locked rotameric states.

To clarify this, we have rewritten the sentence and added the figures as follows.

Page 6

In the initial state, when the **QC6** solution contained no water, approximately 30% of **QC6** converted into **NBD6** within 250 minutes, with a $k^{I}_{6\text{-Fast}}$ value of 1.1 ×10^{-3} min^{-1} (**Figs. 2**d and h). Upon the addition of water, the backconversion rate of **QC6** decreased dramatically, with the $k^{II}_{6\text{-Slow}}$ value reducing to 9.8 ×10^{-5} min^{-1}. When the water ratio reached 20, the back-conversion rate remained nearly constant, even upon further increasing the water ratio to 200 (**Figs. S61, 62** and **Table S18**). In this scenario, the system enables a preferential redistribution of species from the Fast Box to the Slow Box.

NBD6 in MeCN (0.1 mM) with NBD-to-water molar ratio of 1:200

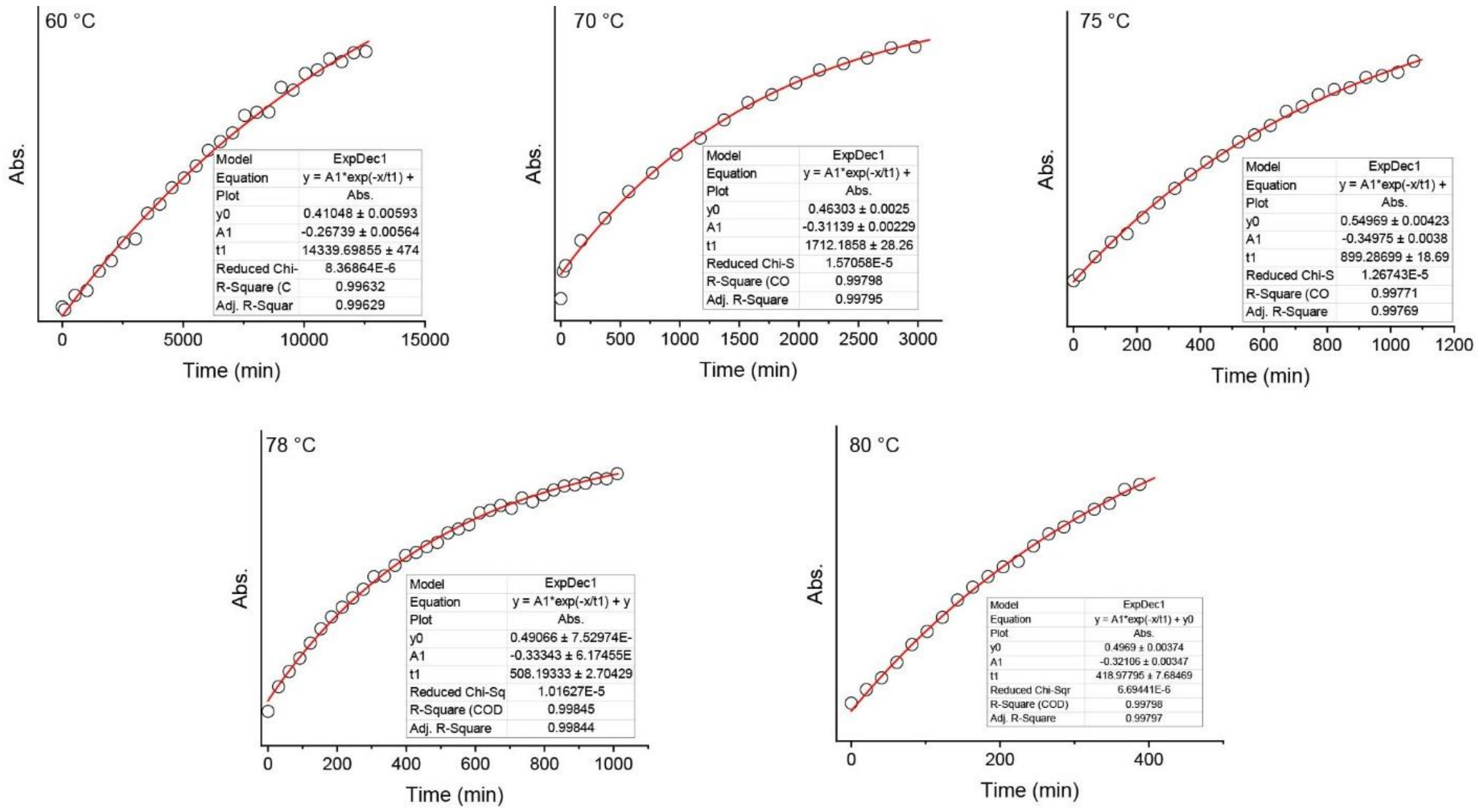

Fig. S61. Thermal back conversion of NBD6 in MeCN (0.1 mM) with NBD-to-water molar ratio of 1:200 at 60, 70, 75, 78, 80 °C. All the curves are fitted by first-order kinetics equation.

Table S18. Thermal backconversion parameters of NBD6 in MeCN (0.1 mM) with NBD-to-water molar ratio of 1:200.					
Backconversion rate	Temperature (°C)				
	60	70	75	78	80
k ($\times10^{-3}$ min^{-1})	0.0697	0.584	1.11	1.97	2.39

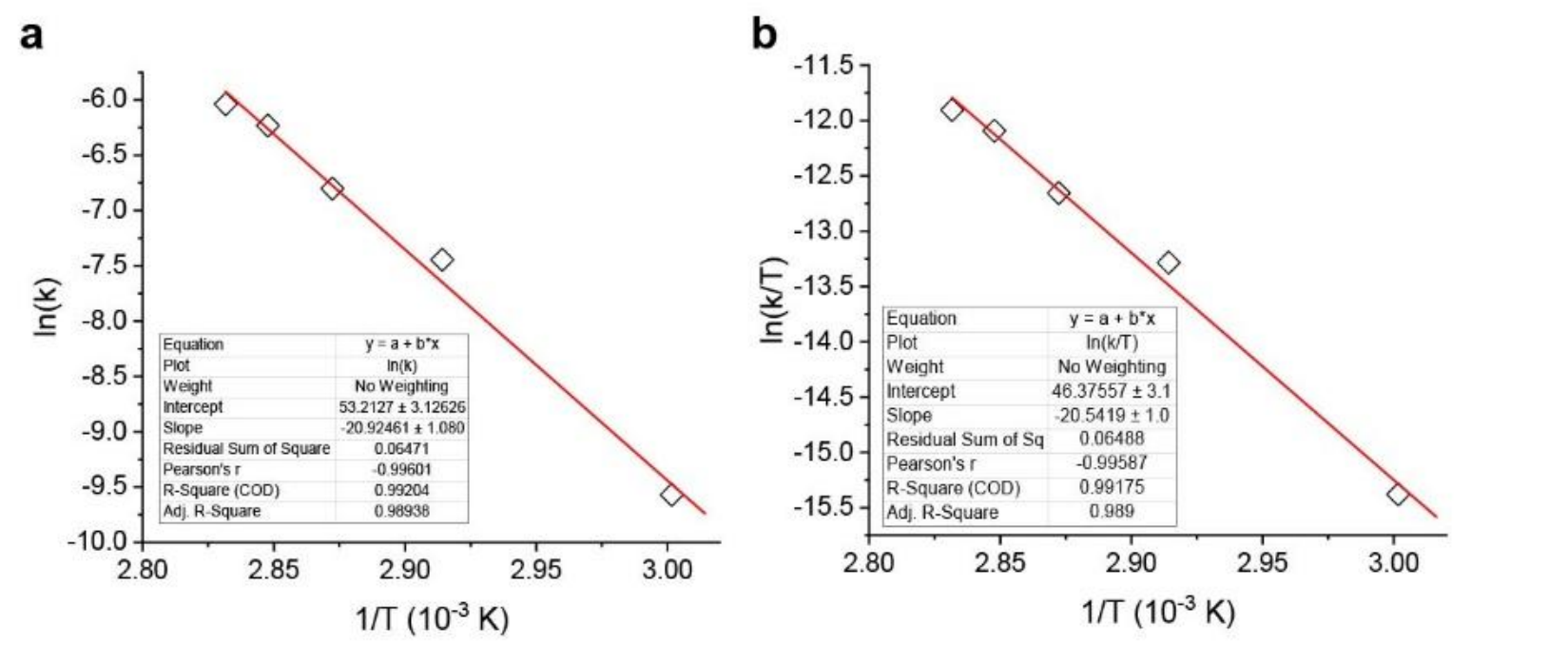

Fig. S62. **a**, Arrhenius plot of NBD6. **b**, Eyring plot of NBD6.

2. The addition of water to acetonitrile not only changes the H-bonding properties of the medium but also other solvent parameters, such as viscosity, density, refractive index, dielectric constant, etc. and the temperature dependence thereof, all of which may also have an influence on the reaction rate. This aspect should be considered and discussed in more detail, especially as water/acetonitrile mixtures have been investigated along these lines already.

Response 2: Thank you for your valuable suggestion. In this study, the amount of added water is very small. For example, even at the highest NBD-to-water molar ratio of 1:20, the water concentration in acetonitrile is only 20 mM (0.0046 wt%) for UV-Vis measurements and 600 mM (1.38 wt%) for NMR measurements. According to the literature (*J. Chem. Eng. Data 52, 1103-1107*), both the density and dielectric constant of acetonitrile change only slightly at low water contents (≤2 wt%) at 25 °C. For instance, the density increases negatively from 0.777 to 0.781 kg dm^{-3} when the water content increases from 0 to 2 wt%. Similarly, the dielectric constant shows only a moderate increase, from 35.9 (pure acetonitrile) to 39.3 at 9.9 wt% water, while pure acetonitrile itself exhibits a slight decrease from 35.9 to 31.1 over the temperature range considered (25 to 60 °C). The viscosity and refractive index change only marginally below 10 wt% water, increasing from 0.35 to 0.40 mPa·s and from 1.342 to 1.343, respectively. (*J. Anal. Chromatogr. Spectrosc. 1, 1-19*) Therefore, the macroscopic changes in solvent properties are minimal under our experimental conditions. In contrast, the local organization of water molecules, such as the formation of hydrogen-bonded clusters around the solute, is expected to have a much more pronounced effect than the small variation in the bulk dielectric constant.

To clarify this, we have rewritten the sentence as follows.

Page 9

These findings are consistent with the corresponding changes observed in the thermal backconversion rate constants (**Table S1-18**). Additionally, even at the highest NBD-

to-water molar ratio (1:20), the water content in acetonitrile remains as low as 20 mM (0.0046 wt%), such that changes in bulk solvent properties are negligible (see Supporting Information Section 2.4.4).

Supporting Information Section 2.4.4

Solvent change effect

In our experiments, the water content is very low. Even at the highest NBD-to-water molar ratio (1:20), the water concentration in acetonitrile is only 20 mM (0.0046 wt%) for UV–Vis and 600 mM (1.38 wt%) for NMR measurements.

Literature data show that such low water contents (≤2 wt%) induce only minor changes in bulk solvent properties.[22] For example, the density increases slightly from 0.777 to 0.781 kg dm^{-3} (0-2 wt% H_2O), while the dielectric constant changes modestly from 35.9 (pure MeCN) to 39.3 even at 9.9 wt% H_2O. The viscosity and refractive index change only marginally below 10 wt% water, increasing from 0.35 to 0.40 mPa·s and from 1.342 to 1.343, respectively.[23]

Therefore, under our conditions, macroscopic solvent effects are negligible. Instead, the observed behavior is more reasonably attributed to local hydrogen-bonding interactions, such as the formation of water clusters or structured solvation shells around the solute, which are expected to dominate over small changes in the bulk dielectric constant.

22 Gagliardi, L. G., Castells, C. B., Ràfols, C., Rosés, M. & Bosch, E. Static Dielectric Constants of Acetonitrile/Water Mixtures at Different Temperatures and Debye−Hückel a and A0b Parameters for Activity Coefficients. *J. Chem. Eng. Data* **52**, 1103-1107 (2007).

23 Рудаков, O., Rudakova, L. & Selemenev, V. Acetonitrile as Tops Solvent for Liquid Chromatography and Extraction. *J. Anal. Chromatogr. Spectrosc.* **1**, 1-19 (2018).

3. It is known that H bonding at carbonyl functionalities may lower HOMO/LUMO energies. Does that also play a role in this case?

Response 3: Thanks for your suggestion. We acknowledge that hydrogen bonding can perturb frontier-orbital energies, but two pieces of existing evidence indicate this is not the operative effect here. First, the rate-determining step is a ground-state structural rearrangement. Our DFT backconversion barriers (Table S28) and the dihedral-scan landscapes (Fig. 5c, Fig. S91) show the barrier change tracks conformational reorganization, not an electronic descriptor. Second, the new D_2O/ACN control shows no kinetic isotope effect (as below), arguing against an electronically/proton-transfer-dominated pathway. Any frontier-orbital stabilization is therefore assumed secondary to the conformational (water-bridge) effect.

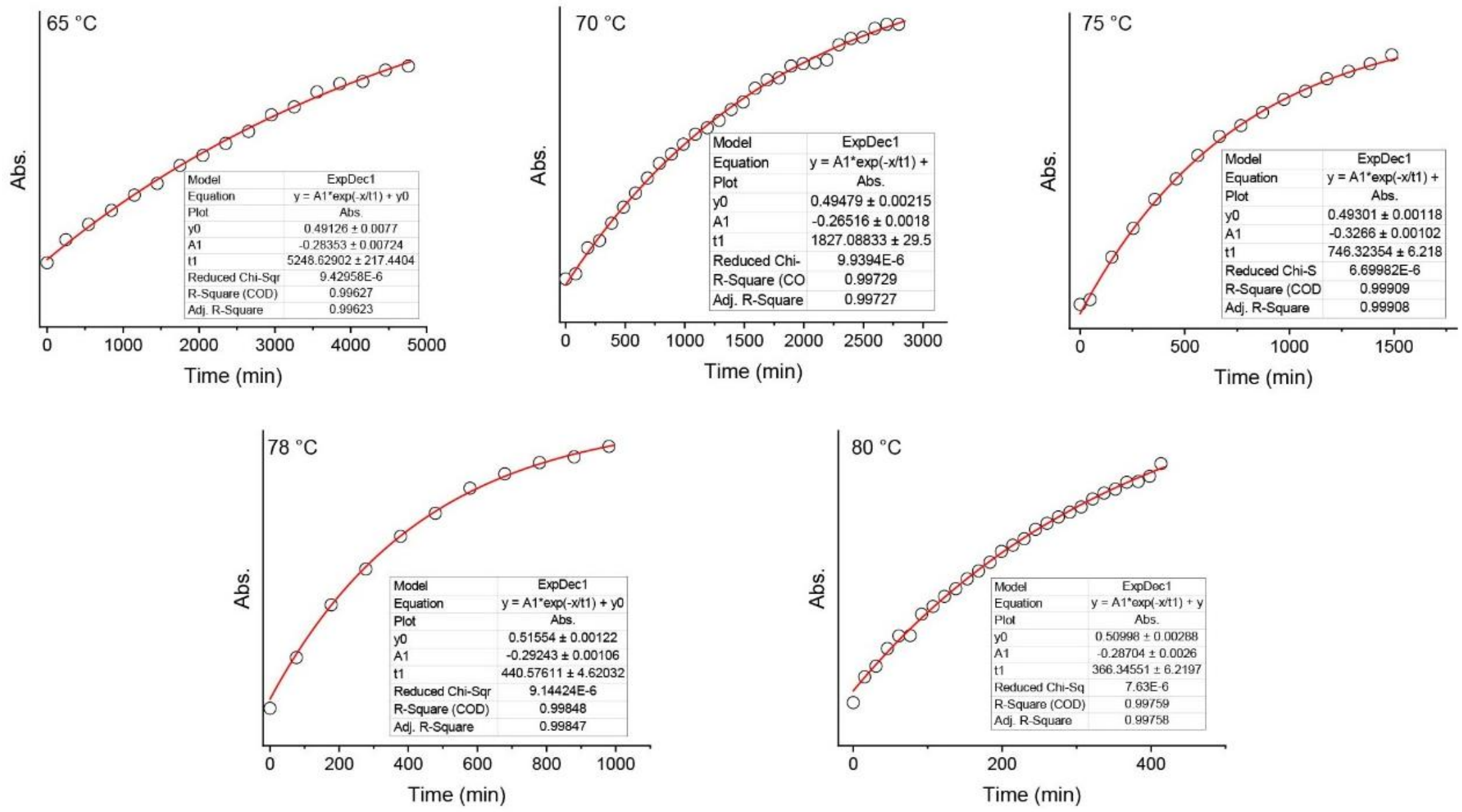

Fig. R1. Thermal back conversion of NBD6 in MeCN (0.1 mM) with NBD-to-D_2O molar ratio of 1:20 at 65, 70, 75, 78, 80 °C. All the curves are fitted by first-order kinetics equation.

Table R1. Thermal backconversion parameters of NBD6 in MeCN (0.1 mM) with NBD-to-D_2O molar ratio of 1:20.					
Backconversion rate	Temperature (°C)				
	65	70	75	78	80
k ($\times10^{-3}$ min^{-1})	0.191	0.547	1.34	2.27	2.72

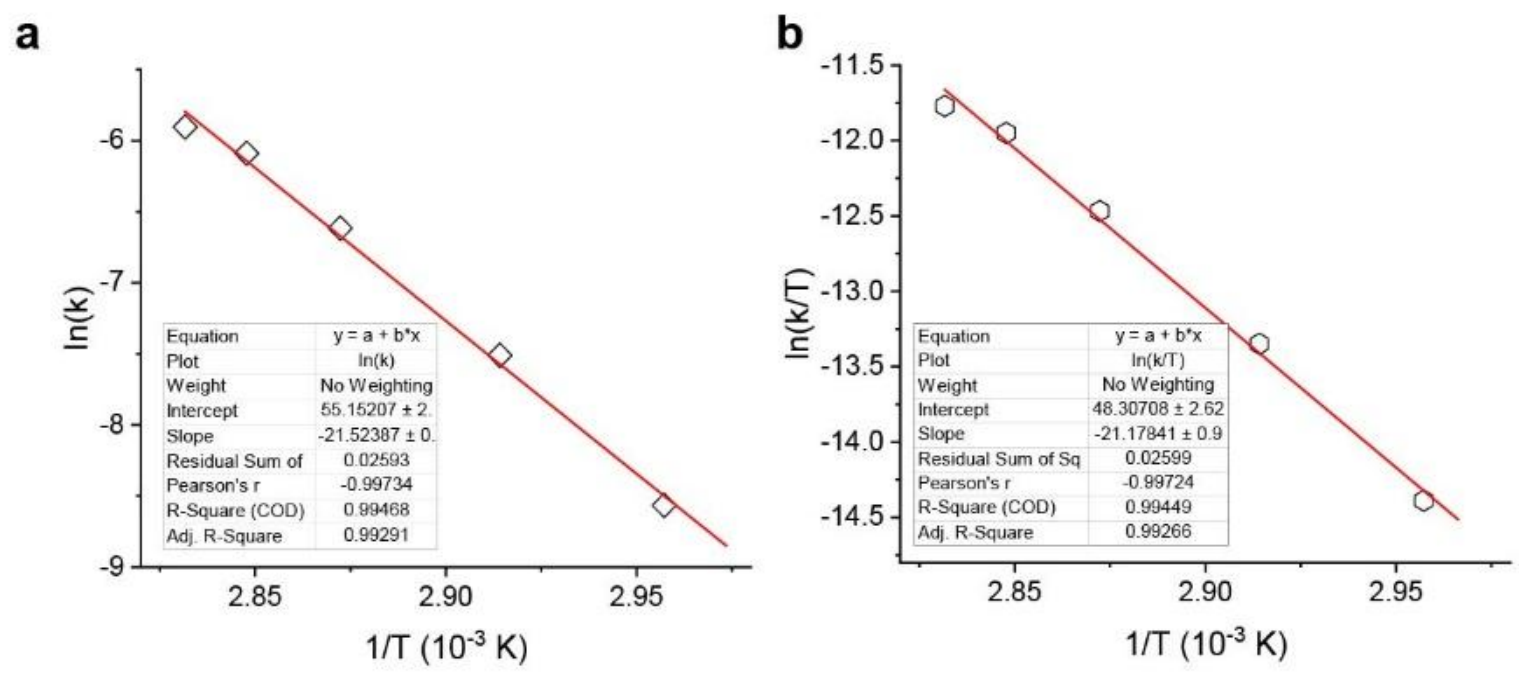

Fig. R2. **a**, Arrhenius plot of NBD6. **b**, Eyring plot of NBD6.

As shown in the Figs. R1, R2 and Table R1 as below, the back-conversion kinetics (half-life at 25 °C is 8699 days) in D_2O/ACN were nearly identical to those observed in H_2O/ACN, and no significant kinetic isotope effect was detected within the experimental uncertainty.

4. Experimental

– 4.1 Compound characterization: Exact mass deviates in some cases quite a bit from the theoretical value. Please indicate the intrinsic error margin/standard deviation of the analysis and the acceptable difference between experimental and theoretical value.

Response 4.1: Thank you for your suggestion. The mass errors were evaluated using the standard equation, and relatively large deviations (>10 ppm) were observed for NBD3 and NBD5 (see Table R2 below). Such deviations can arise from several experimental factors, including minor calibration drift, limited signal-to-noise ratios, and the presence of sodium/potassium adducts or noncovalent solvent-associated species under ESI conditions. Accordingly, the HRMS measurements for NBD3 and NBD5 were repeated, affording accurate mass data in good agreement with the calculated values.

$$error\ (ppm) = \frac{m_{exp} - m_{cal}}{m_{cal}} \times 10^6$$

Table R2. Mass errors of NBDs

	NBDs					
	1	2	3	4	5	6
Mass error before (ppm)	6.18	4.02	25.88	2.07	11.80	3.02
Mass error corrected (ppm)	-	-	0.4	-	2.2	-

The ESI-MS measurements were performed in positive-ion mode using a Waters Synapt G2-Si mass spectrometer with direct infusion. The elemental composition analysis is provided in the screenshots below.

In addition, we summarized the results in a table. Both the protonated molecular ion ($M+H^+$) and the sodium adduct ($M+Na^+$) were observed in the spectra, and elemental composition analysis confirmed that both species matched the expected molecular formula with low mass error.

Re-examination of the MS data

NBD3

Calculated mass	Measured mass	Chemical formula		Error (ppm)
251.1184	251.1183	$C_{16}H_{15}N_2O$	$M + H^+$	0.4
273.1004	273.1003	$C_{16}H_{14}N_2ONa$	$M + Na^+$	0.4

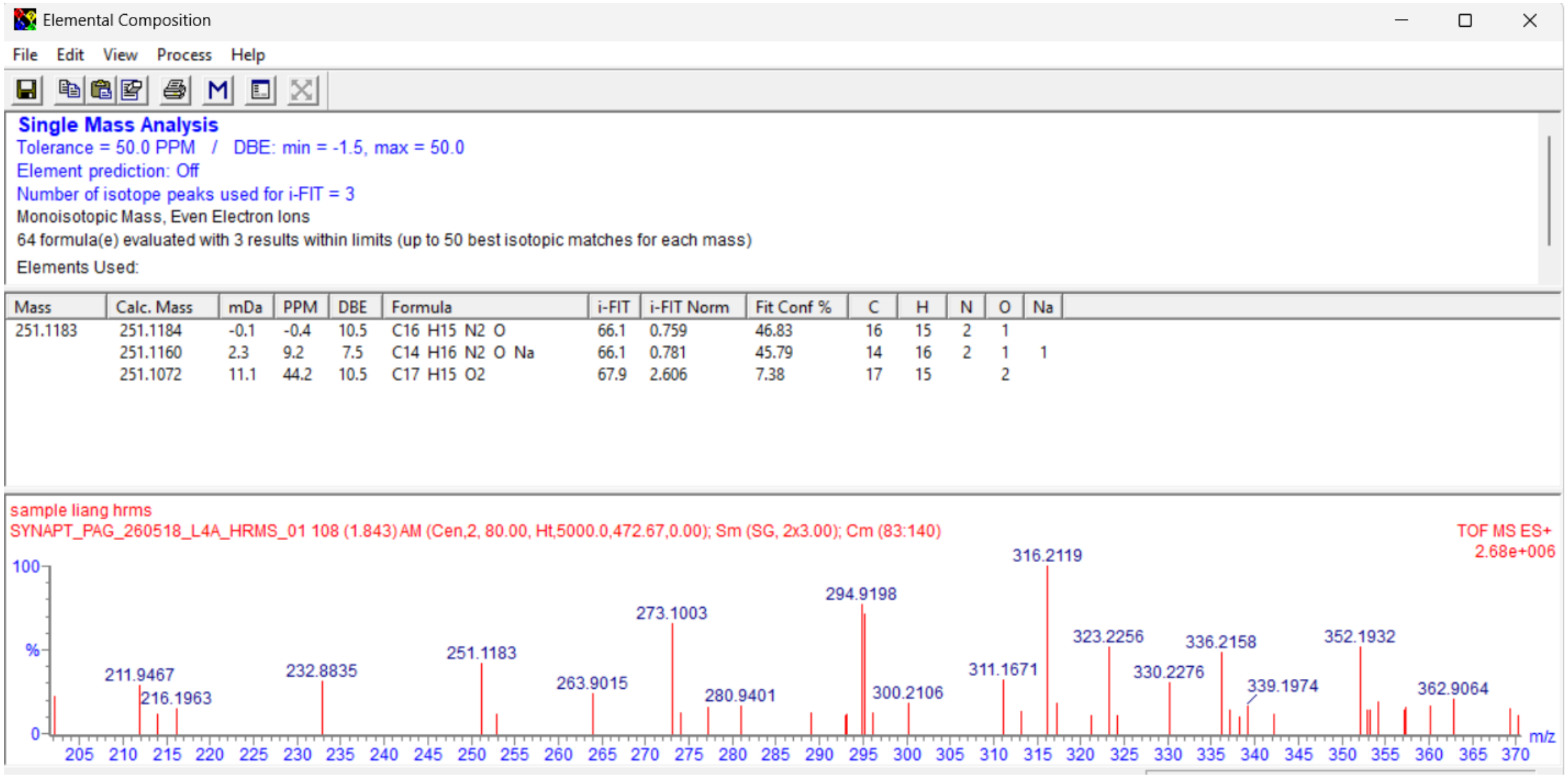

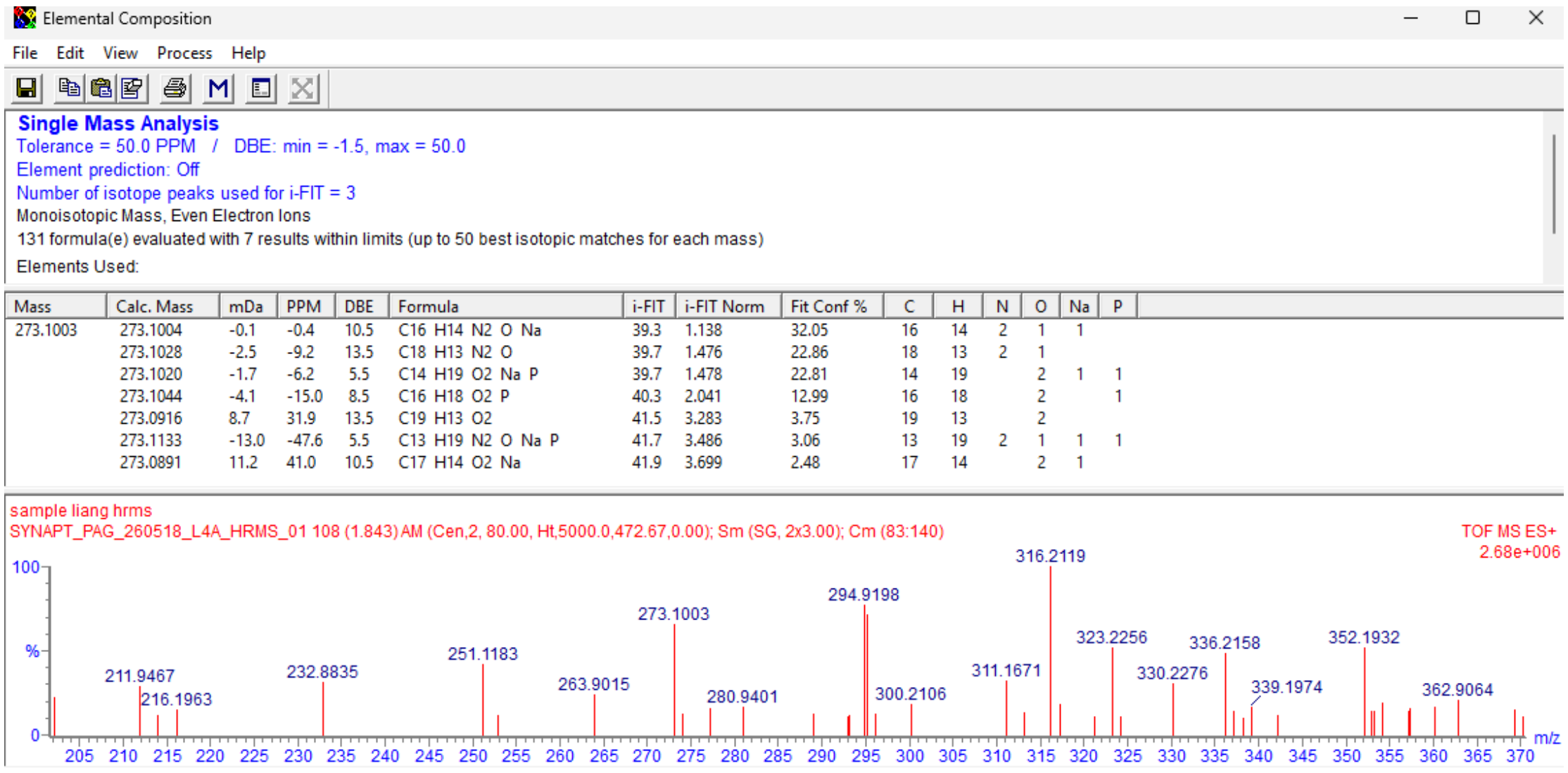

NBD5

Calculated mass	Measured mass	Chemical formula		Error (ppm)
271.1334	271.1328	$C_{17}H_{19}O_3$	M + H^+	2.2
293.1154	293.1154	$C_{17}H_{18}O_3Na$	M + Na^+	0.0

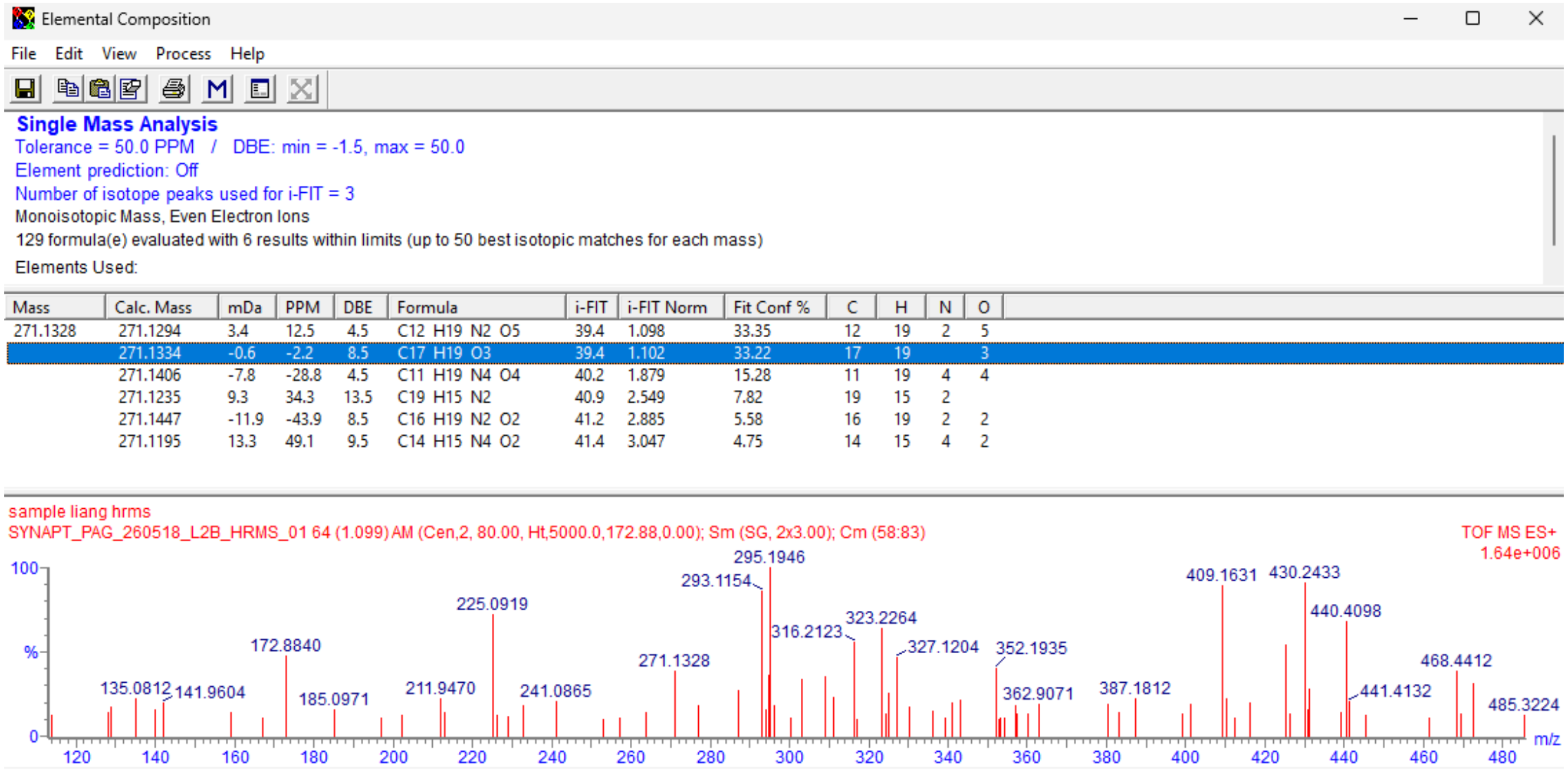

Elemental Composition

File Edit View Process Help

Single Mass Analysis
Tolerance = 50.0 PPM / DBE: min = -1.5, max = 50.0
Element prediction: Off
Number of isotope peaks used for i-FIT = 3
Monoisotopic Mass, Even Electron Ions
129 formula(e) evaluated with 6 results within limits (up to 50 best isotopic matches for each mass)
Elements Used:

Mass	Calc. Mass	mDa	PPM	DBE	Formula	i-FIT	i-FIT Norm	Fit Conf %	C	H	N	O
271.1328	271.1294	3.4	12.5	4.5	C12 H19 N2 O5	39.4	1.098	33.35	12	19	2	5
	271.1334	-0.6	-2.2	8.5	C17 H19 O3	39.4	1.102	33.22	17	19		3
	271.1406	-7.8	-28.8	4.5	C11 H19 N4 O4	40.2	1.879	15.28	11	19	4	4
	271.1235	9.3	34.3	13.5	C19 H15 N2	40.9	2.549	7.82	19	15	2	
	271.1447	-11.9	-43.9	8.5	C16 H19 N2 O2	41.2	2.885	5.58	16	19	2	2
	271.1195	13.3	49.1	9.5	C14 H15 N4 O2	41.4	3.047	4.75	14	15	4	2

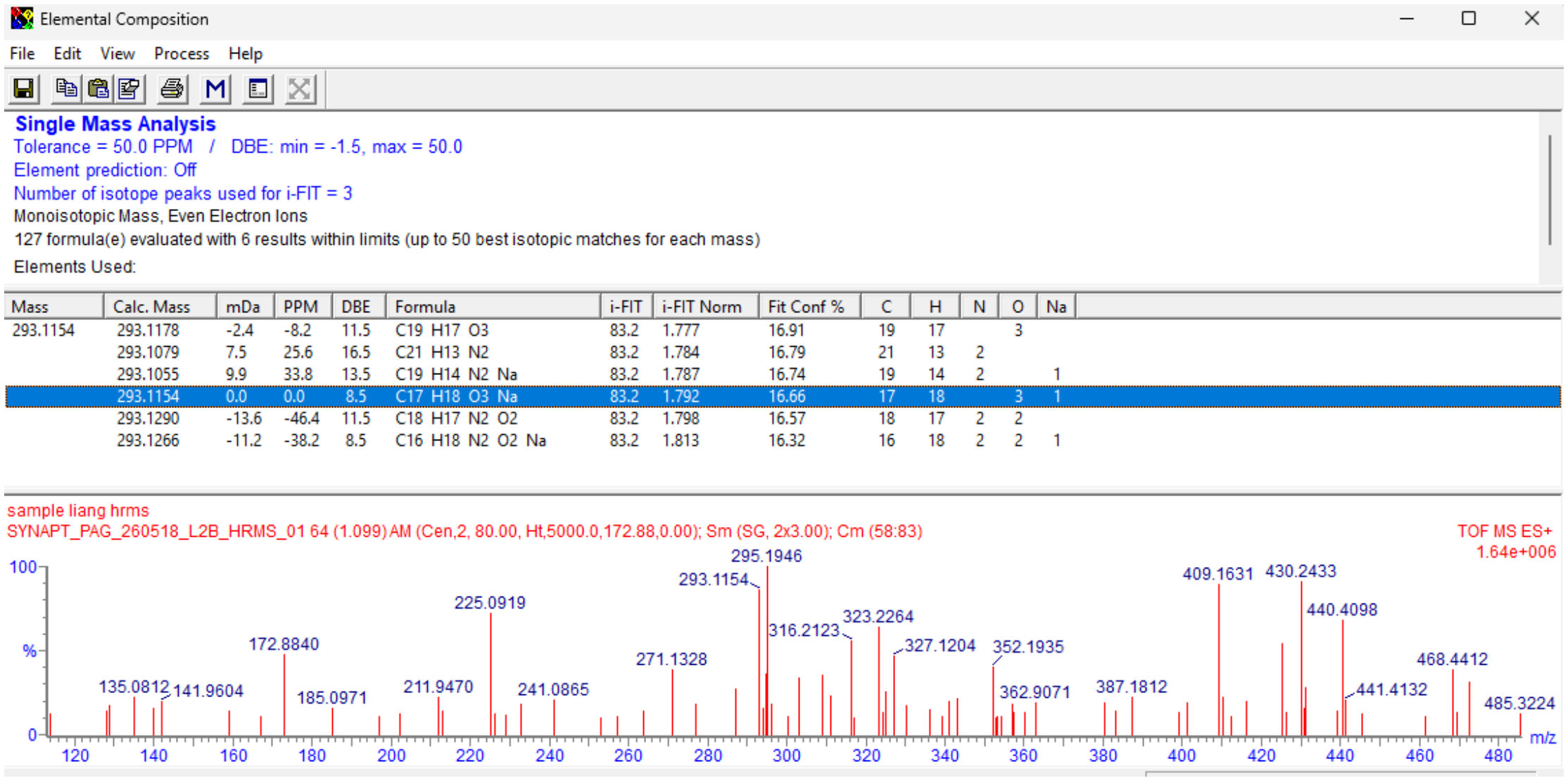

Elemental Composition

File Edit View Process Help

Single Mass Analysis
Tolerance = 50.0 PPM / DBE: min = -1.5, max = 50.0
Element prediction: Off
Number of isotope peaks used for i-FIT = 3
Monoisotopic Mass, Even Electron Ions
127 formula(e) evaluated with 6 results within limits (up to 50 best isotopic matches for each mass)
Elements Used:

Mass	Calc. Mass	mDa	PPM	DBE	Formula	i-FIT	i-FIT Norm	Fit Conf %	C	H	N	O	Na
293.1154	293.1178	-2.4	-8.2	11.5	C19 H17 O3	83.2	1.777	16.91	19	17		3	
	293.1079	7.5	25.6	16.5	C21 H13 N2	83.2	1.784	16.79	21	13	2		
	293.1055	9.9	33.8	13.5	C19 H14 N2 Na	83.2	1.787	16.74	19	14	2		1
	293.1154	0.0	0.0	8.5	C17 H18 O3 Na	83.2	1.792	16.66	17	18		3	1
	293.1290	-13.6	-46.4	11.5	C18 H17 N2 O2	83.2	1.798	16.57	18	17	2	2	
	293.1266	-11.2	-38.2	8.5	C16 H18 N2 O2 Na	83.2	1.813	16.32	16	18	2	2	1

P8

The product, **NBD3**, was obtained as a pure, yellow solid in 23% yield. ^{1}H NMR (400 MHz, $CDCl_3$) δ 7.74 (d, *J* = 8.8 Hz, 2H), 7.63 (d, *J* = 8.8 Hz, 2H), 7.53 (s, 1H), 6.96 (dd, *J* = 5.2, 2.9 Hz, 1H), 6.86 (dd, *J* = 5.4, 2.8 Hz, 1H), 4.14 (s, 1H), 3.95 (s, 1H), 2.33 – 2.26 (m, 1H), 2.26 – 2.17 (m, 4H). ^{13}C NMR (101 MHz, $CDCl_3$) δ 170.28, 168.45, 143.17, 140.13, 139.69, 128.83, 127.48, 119.51, 115.18, 114.71, 70.97, 54.85, 54.00, 29.72. HRMS (ESI) m/z: $[M + H]^+$ calcd for $C_{16}H_{15}N_2O$, 251.1184; found, 251.1083. $[M + Na]^+$ calcd for $C_{16}H_{14}N_2ONa$, 273.1004; found, 273.1003.

P9

The product, **NBD5**, was obtained as a pure, light yellow solid in 46% yield. ^{1}H NMR (400 MHz, Acetonitrile-d_3) δ 7.56 (d, *J* = 8.9 Hz, 2H), 6.95 (t, *J* = 1.9 Hz, 1H), 6.92 (d, *J* = 8.9 Hz, 2H), 4.15 – 4.06 (m, 2H), 3.99 (h, *J* = 1.7 Hz, 1H), 3.88 (h, *J* = 1.7 Hz, 1H), 3.81 (s, 3H), 2.18 (t, *J* = 1.7 Hz, 1H), 2.03 – 1.96 (m, 1H), 1.20 (t, *J* = 7.1 Hz, 3H). ^{13}C NMR (101 MHz, Acetonitrile-d_3) δ 167.15, 160.74, 144.04, 141.53, 137.76, 130.35,

128.57, 113.72, 70.46, 60.44, 58.74, 55.63, 53.68, 14.18. HRMS (ESI) m/z: $[M + H]^+$ calcd for $C_{17}H_{19}O_3$, 271.1334; found, 271.1328. $[M + Na]^+$ calcd for $C_{17}H_{18}O_3Na$, 293.1154; found, 293.1154.

– 4.2 Please give solvent in caption of NMR spectra in SI.

Response 4.2: Thank you for your suggestion. We have checked and included the solvent information in all captions of the NMR spectra in both the main text and SI.

To clarify this, we have rewritten the sentence as follows.

Fig. 4. Evidence for the hydrogen bonding system. a, ^1H NMR and ^{13}C NMR, **b** peaks of NH, and **c** peaks of CO of **NBD6/QC6** pair with different ratios of water in CD_3CN at 25 °C. **d**, Chemical shifts of protons and carbons of **NBD6** in ^1H-^{13}C HMBC spectra with and without water in CD_3CN at 25 °C. **e**, Raman spectra of **QC6** solution, MeCN, water. **f**, ^1H NMR of **NBD6/QC6** with and without water under various temperatures in CD_3CN at 25 °C. **g**, Cycles of hydrogen bonding forming in **QC6** CD_3CN solution.

Fig. S3.^1H NMR of NBD-CN,Br in $CDCl_3$ at 25 °C.
Fig. S4.^{13}C NMR of NBD-CN,Br in $CDCl_3$ at 25 °C.
Fig. S5. **^1H NMR of NBD1 in $CDCl_3$ at 25 °C.**
Fig. S6. **^{13}C NMR of NBD1 in $CDCl_3$ at 25 °C.**
Fig. S7. **^1H NMR of NBD2 in CD_3CN at 25 °C.**
Fig. S8. **^{13}C NMR of NBD2 in CD_3CN at 25 °C.**
Fig. S9. **^1H NMR of NBD3 in $CDCl_3$ at 25 °C.**
Fig. S10. **^{13}C NMR of NBD3 in $CDCl_3$ at 25 °C.**
Fig. S11. **^1H NMR of ethyl 3-bromopropiolate in $CDCl_3$ at 25 °C.**
Fig. S12. **^{13}C NMR of ethyl 3-bromopropiolate in $CDCl_3$ at 25 °C.**
Fig. S13. **^1H NMR of NBD-Ester,Br in $CDCl_3$ at 25 °C.**
Fig. S14. **^{13}C NMR of NBD-Ester,Br in $CDCl_3$ at 25 °C.**
Fig. S15. **^1H NMR of NBD4 in $CDCl_3$ at 25 °C.**
Fig. S16. **^{13}C NMR of NBD4 in $CDCl_3$ at 25 °C.**
Fig. S17. **^1H NMR of NBD5 in CD_3CN at 25 °C.**
Fig. S18. **^{13}C NMR of NBD5 in CD_3CN at 25 °C.**
Fig. S19. **^1H NMR of NBD6 in $CDCl_3$ at 25 °C.**
Fig. S20. **^{13}C NMR of NBD6 in $CDCl_3$ at 25 °C.**

5. Format

–5.1 In chemical structures, substituents R should not be assigned by subscript numbers, because this position is reserved for the number of the substituents (I know that this is a ChemDraw default, but it contradicts formalism in Organic Chemistry). Please place the index superscript instead.

Response 5.1: Thank you for your suggestion. We have revised the chemical structures accordingly.

–5.2 page 3: change "information carriers information carriers" to " information carriers"

Response 5.2: Thank you for pointing this out. We have corrected it.

–5.3 page 9, last line: change " experiemental" to " experimental"

Response 5.3: Thank you for pointing this out. We have corrected it.

Reply to Reviewer: 3

Reviewer 3: In this work, the authors present a supramolecular strategy to control the thermal back-conversion of the norbornadiene/quadricyclane (NBD/QC) photoswitch pair by using hydrogen-bond donors and acceptors that recruit water molecules as a reversible "bridge." The authors frame this as a molecular analogue of Maxwell's demon: the water-mediated hydrogen-bond network selectively locks certain QC conformations, reshapes the rotational energy landscape, raises the activation barrier for QC-to-NBD back-isomerization, and can even redirect the reaction pathway. Experimentally, it is shown that compounds with suitable hydrogen-bonding motifs, especially QC6, exhibit dramatically slowed thermal back-conversion, with the half-life extended by about 5000-fold upon aqueous solution conditions, while compounds lacking the necessary hydrogen-bonding architecture show little effect. NMR, Raman, DFT, and molecular dynamics analyses support the conclusion that reversible water-bridged hydrogen bonding is responsible for this tunable kinetic gating. Overall, the authors propose a method to control thermal isomerization kinetics in molecular photoswitches via hydrogen bond interactions with solvent.

However, in my view, the conceptual novelty of this work remains limited, as it primarily concerns solvent control of reaction barriers, which is not in itself a new concept. Solvent effects, particularly hydrogen-bonding interactions, have long been known to modulate reaction barriers. Accordingly, the observation of hydrogen-bond-controlled thermal isomerization rates in this study does not, by itself, constitute a fundamentally new finding. I also find the framing of this phenomenon as a realization of Maxwell's demon to be misleading. In the original thought experiment, Maxwell's demon appears to reduce entropy without directly performing work on the system. That analogy does not convincingly apply here. In the present case, adding water to the solvent introduces isomer-dependent, species-specific hydrogen-bonding interactions that directly alter the enthalpy of the molecular system. The resulting energetic stabilization of certain QC derivatives by water is therefore the direct origin of the increased isomerization barrier. If an analogy has to be made to the Maxwell's demon in this case, then the demon actually slow down some of the moving particles in the though experiment, amounting to doing work to the system. In this sense, the phenomenon is more appropriately understood as a conventional solvation effect, namely a lowering of the reactant-state enthalpy through selective solute-solvent interactions, rather than as a genuine Maxwell's demon mechanism.

Overall, I find the Maxwell's demon interpretation superficial, as the central effect is essentially solvent-induced modulation of the reaction energy landscape. More broadly, a substantial body of prior work has already examined how the molecular environment influences the kinetics of photochemical and ground-state reactions of photoswitches, including phenomena such as aggregation-induced emission and turn-on fluorescence. Therefore, although this study presents a useful strategy for tuning the thermal half-lives of photoswitches without strongly perturbing their absorption wavelengths, I do not believe its conceptual novelty meets the level typically expected for publication in Nature Communications.

We highly appreciate the reviewer's careful consideration of the conceptual framework of our work and valuing our work in control of reaction barriers by hydrogen bonding system. The comments have helped us clarify both the novelty and the intended

interpretation of the “Maxwell’s demon” analogy. The reviewer’s main concerns focus on the limited conceptual novelty of the work and the validity of the “Maxwell’s demon” interpretation. Below, we respond point-by-point.

1. The conceptual novelty is limited because solvent-controlled reaction barriers and hydrogen-bond-mediated kinetic modulation are already well known.

Response 1: We agree with the reviewer that solvent effects and hydrogen-bond-mediated modulation of reaction barriers are well-established concepts in chemistry. However, we want to point out here the major key findings of our studies.

First of all, the conceptual advance of our work does not lie in the solvent effects, but rather focuses on the water-locked rotameric effect.

In this study, the amount of added water is very small. For example, even at the highest NBD-to-water molar ratio of 1:20, the water concentration in acetonitrile is only 20 mM (0.0046 wt%) for UV-Vis measurements and 600 mM (1.38 wt%) for NMR measurements. According to the literature (*J. Chem. Eng. Data 52, 1103-1107*), both the density and dielectric constant of acetonitrile change only slightly at low water contents (≤2 wt%) at 25 °C. For instance, the density increases negatively from 0.777 to 0.781 kg dm^{-3} when the water content increases from 0 to 2 wt%. Similarly, the dielectric constant shows only a moderate increase, from 35.9 (pure acetonitrile) to 39.3 at 9.9 wt% water, while pure acetonitrile itself exhibits a slight decrease from 35.9 to 31.1 over the temperature range considered (25 to 60 °C). The viscosity and refractive index change only marginally below 10 wt% water, increasing from 0.35 to 0.40 mPa·s and from 1.342 to 1.343, respectively. (*J. Anal. Chromatogr. Spectrosc. 1, 1-19*) Therefore, the macroscopic changes in solvent properties are minimal under our experimental conditions. In contrast, the local organization of water molecules, such as the formation of hydrogen-bonded clusters around the solute, is expected to have a much more pronounced effect than the small variation in the bulk dielectric constant. In addition, we supplemented an experiment with QC6 at a higher water ratio of 1:200, where the back-conversion rate showed no further change compared to that at 1:20. These observations suggest that water molecules interact with the functional groups of the quadricyclane, rather than the solvent effect.

We also acknowledge the substantial literature on environment-controlled photoswitch kinetics, including aggregation- and turn-on-fluorescence systems. Our advance is not a generic medium effect but a geometry-encoded, reversible water-bridge that restricts a specific rotational coordinate and reroutes the back-conversion pathway. An effect that is switched on and off by low water content and is absent in some compounds (QC1-2) lacking the bridging geometry.

To clarify this, we have rewritten the sentence as follows.

Page 9

These findings are consistent with the corresponding changes observed in the thermal backconversion rate constants (**Table S1-18**). Additionally, even at the highest NBD-to-water molar ratio (1:20), the water content in acetonitrile remains as low as 20 mM (0.0046 wt%), such that changes in bulk solvent properties are negligible (see Supporting Information Section 2.4.4).

Supporting Information Section 2.4.4

Solvent change effect

In our experiments, the water content is very low. Even at the highest NBD-to-water molar ratio (1:20), the water concentration in acetonitrile is only 20 mM (0.0046 wt%) for UV–Vis and 600 mM (1.38 wt%) for NMR measurements.

Literature data show that such low water contents (≤2 wt%) induce only minor changes in bulk solvent properties.[22] For example, the density increases slightly from 0.777 to 0.781 kg dm^{-3} (0-2 wt% H_2O), while the dielectric constant changes modestly from 35.9 (pure MeCN) to 39.3 even at 9.9 wt% H_2O. The viscosity and refractive index change only marginally below 10 wt% water, increasing from 0.35 to 0.40 mPa·s and from 1.342 to 1.343, respectively.[23]

Therefore, under our conditions, macroscopic solvent effects are negligible. Instead, the observed behavior is more reasonably attributed to local hydrogen-bonding interactions, such as the formation of water clusters or structured solvation shells around the solute, which are expected to dominate over small changes in the bulk dielectric constant.

22 Gagliardi, L. G., Castells, C. B., Ràfols, C., Rosés, M. & Bosch, E. Static Dielectric Constants of Acetonitrile/Water Mixtures at Different Temperatures and Debye−Hückel a and A0b Parameters for Activity Coefficients. *J. Chem. Eng. Data* **52**, 1103-1107 (2007).

23 Рудаков, O., Rudakova, L. & Selemenev, V. Acetonitrile as Tops Solvent for Liquid Chromatography and Extraction. *J. Anal. Chromatogr. Spectrosc.* **1**, 1-19 (2018).

Page 6

In the initial state, when the **QC6** solution contained no water, approximately 30% of **QC6** converted into **NBD6** within 250 minutes, with a $k^{I}_{6\text{-Fast}}$ value of 1.1 ×10^{-3} min^{-1} (**Figs. 2**d and h). Upon the addition of water, the backconversion rate of **QC6** decreased dramatically, with the $k^{II}_{6\text{-Slow}}$ value reducing to 9.8 ×10^{-5} min^{-1}. When the water ratio reached 20, the back-conversion rate remained nearly constant, even upon further increasing the water ratio to 200 (**Figs. S61, 62** and **Table S18**). In this scenario, the system enables a preferential redistribution of species from the Fast Box to the Slow Box.

NBD6 in MeCN (0.1 mM) with NBD-to-water molar ratio of 1:200

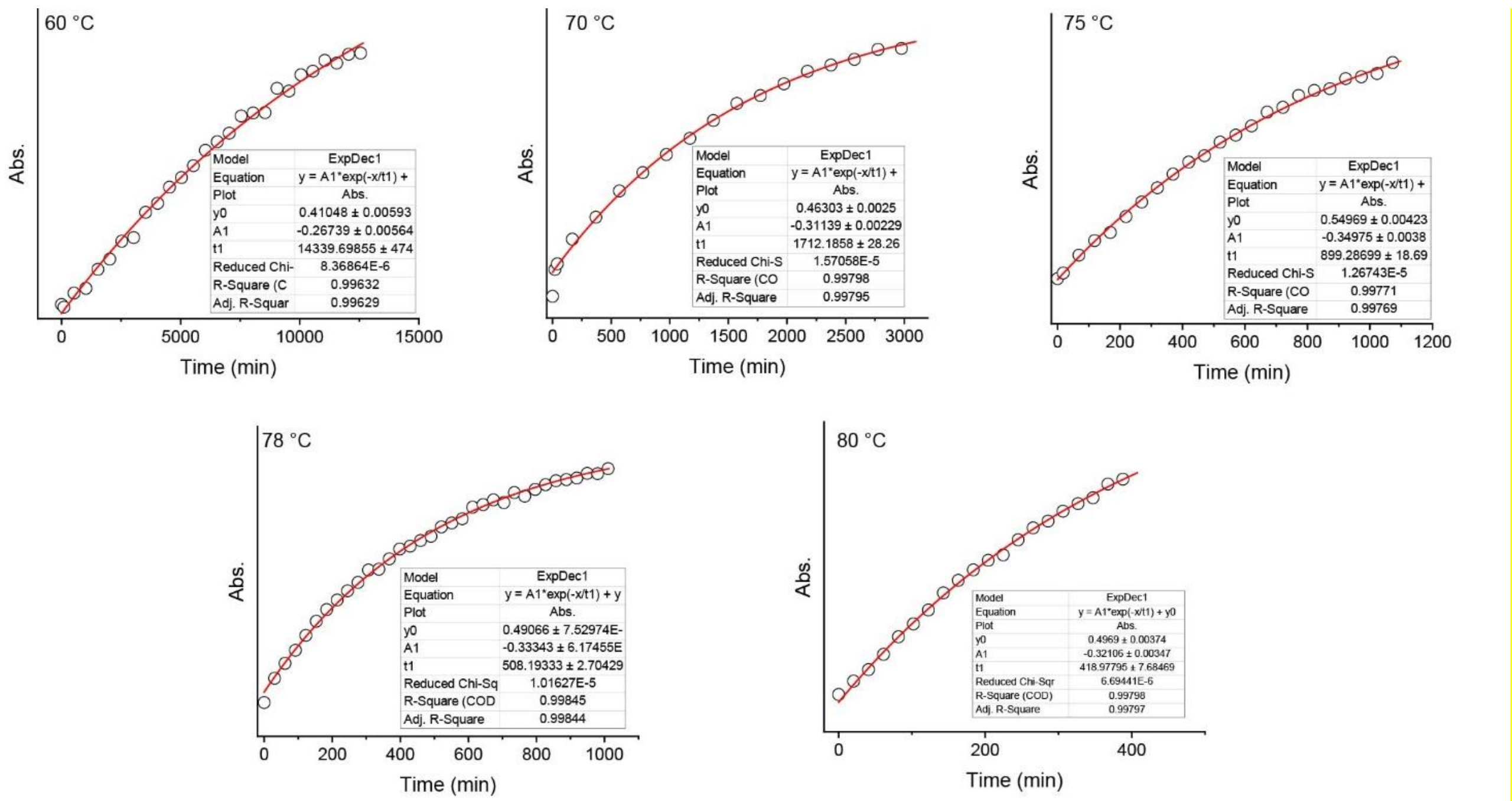

Fig. S61. Thermal back conversion of NBD6 in MeCN (0.1 mM) with NBD-to-water molar ratio of 1:200 at 60, 70, 75, 78, 80 °C. All the curves are fitted by first-order kinetics equation.

Table S18. Thermal backconversion parameters of NBD6 in MeCN (0.1 mM) with NBD-to-water molar ratio of 1:200.

Backconversion rate	Temperature (°C)				
	60	70	75	78	80
k ($\times 10^{-3}$ min^{-1})	0.0697	0.584	1.11	1.97	2.39

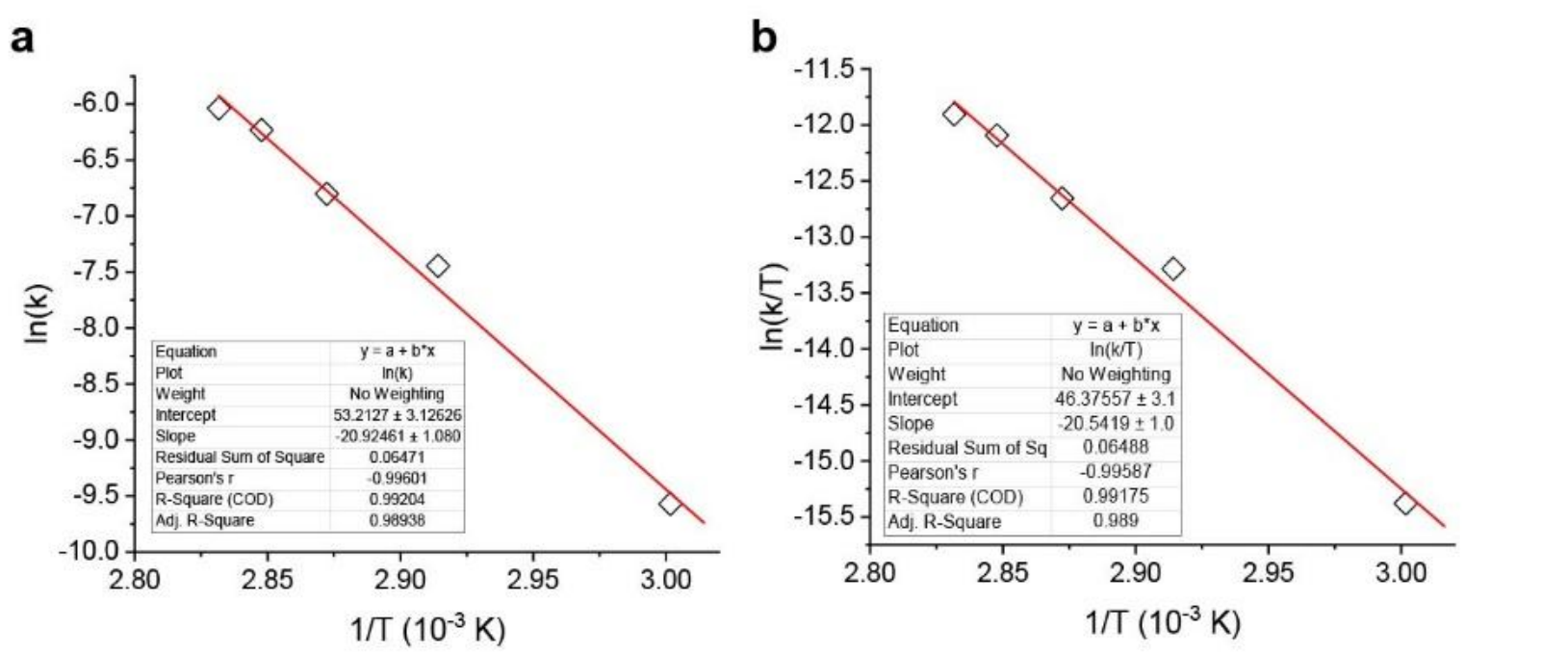

Fig. S62. **a**, Arrhenius plot of NBD6. **b**, Eyring plot of NBD6.

Secondly, large kinetic modulation by the hydrogen bonding system.

A key outcome of our system is that the thermal half-life can be dramatically tuned without changing the absorption wavelength. This decoupling between thermal backconversion kinetics and optical properties is important in molecular photoswitches, particularly for information storage and energy storage applications, where maintaining

red-shifted absorption is highly desirable. In addition, the magnitude of half-life modulation achieved through this hydrogen-bonding strategy exceeds that reported for conventional solvent-effect-based approaches (see Table S27), with the thermal half-life tunable over a remarkably broad timescale, from hours to years.

To clarify this, we have supplemented a comparison table and relative sentence as follows.

Page 9

Compared to previously reported NBD/QC pairs,[19,31,59-62] QC3-6 demonstrated significantly extended t1/2 values, attributed to the formation of hydrogen bonding networks, without compromising blue-shifted absorption or introducing excessive steric bulk (Fig. 3d). In addition, the magnitude of half-life modulation achieved through this hydrogen-bonding strategy exceeds that reported for conventional solvent-effect-based approaches (see Table S27), with the thermal half-life tunable over a remarkably broad timescale, from hours to years.

Table S27. Comparison of thermal half-life control in photoswitches achieved through solvent effects in literatures and the hydrogen-bonding strategy employed in this study.

Photoswitches	Minimum half-life [a]		Maximum half-life [b]		Changes [c]	Ref.
	Value	Solvent used	Value	Solvent used		
Norbornadiene derivatives with an asymmetric 1,2 substitution	17 h	Hexane	29 h	MeCN	Hour-scale regulation (~2 times)	(24)
	4.1 h	Hexane	8.8 h	MeCN		
Azobenzazoles	0.4 s	MeCN	84 s	Toluene	Second-scale regulation (~210 times)	(25)
Nitrogen Bridged Diazocines	27 min	Acetone	73 min	Water	Minute-scale regulation (~3 times)	(26)
Diazocine-Based Estrogen Receptor Agonists	17.2 h	DMSO	42.4 h	H_2O/D_2O	From seconds to minutes (~40 times)	(27)
	6.6 h	DMSO	40.7 h	H_2O/D_2O		
	3.6 s	DMSO	2.4 min	H_2O/D_2O		
	1.5 s	DMSO	19.9 s	H_2O/D_2O		
	4.0 h	DMSO	15.2 h	H_2O/D_2O		
	11.2 h	DMSO	26.1 h	H_2O/D_2O		
	11.4 h	DMSO	31.7 h	H_2O/D_2O		
Azothiophene-based molecular switches	8.6 s	Methanol	4.0 min	Isopropanol	From seconds to minutes (~28 times)	(28)
	30 s	DMF	6.8 min	n-Propanol		(29)

mexylaminotriazine-substituted merocyanine	8.2 s<br>19 s	Toluene<br>THF	24.2 min<br>25.3 min	Ethanol<br>Ethanol	From seconds to minutes (~177 times)	
Donor-acceptor Stenhouse adducts	21.7 s	Toluene	28.6 s	Chloroform	Second-scale regulation (~1.3 times)	(30)
Z-Tetra-ortho-fluoroazobenzene	14 h<br>193 d	DMSO-*d6*<br>Methanol-*d4*	48 h<br>503 d	Toluene-*d8*<br>DMSO-*d6*	Hour- or day-scale regulation (~4 times)	(31)
NBD1	26 d	MeCN [e]	32 d	MeCN [f]	**From hours to years (~5000 times)**	This work
NBD2	58 d	MeCN [e]	62 d	MeCN [f]		
NBD3	4.0 d	MeCN [e]	39 d	MeCN [f]		
NBD4	92 d	MeCN [e]	1.6 y	MeCN [f]		
NBD5	48 d	MeCN [e]	1.3 y	MeCN [f]		
NBD6	38.4 h	MeCN [e]	24 y	MeCN [f]		

[a] The minimum half-life and solvent used in the reported literature
[b] The maximum half-life and solvent used in the reported literature
[c] The Maximum change in half-life
[d] DMSO−water 1:1 + 25 μL 1,8-Diazabicyclo[5.4.0] undec-7-ene
[e] MeCN, extra dry, 99.9%, over molecular sieve, AcroSeal®
[f] MeCN with water (NBD-to-water molar ratio is 1:20)

24 Quant, M., Hamrin, A., Lennartson, A., Erhart, P. & Moth-Poulsen, K. Solvent Effects on the Absorption Profile, Kinetic Stability, and Photoisomerization Process of the Norbornadiene–Quadricyclanes System. *J. Phys. Chem. C* **123**, 7081-7087 (2019).

25 Kennedy, A. D. W., Sandler, I., Andréasson, J., Ho, J. & Beves, J. E. Visible-Light Photoswitching by Azobenzazoles. *Chem. Eur. J.* **26**, 1103-1110 (2020).

26 Lentes, P. *et al.* Nitrogen Bridged Diazocines: Photochromes Switching within the near-Infrared Region with High Quantum Yields in Organic Solvents and in Water. *J. Am. Chem. Soc.* **141**, 13592-13600 (2019).

27 Ewert, J. *et al.* Photoswitchable Diazocine-Based Estrogen Receptor Agonists: Stabilization of the Active Form inside the Receptor. *J. Am. Chem. Soc.* **144**, 15059-15071 (2022).

28 Zhang, X. *et al.* Azothiophene-Based Molecular Switches: Influence of Substituent Position and Solvent Environment on Photophysical Behavior. *Phys. Chem. Chem. Phys.* **28**, 1173-1188 (2026).

29 Piskorz, K., Dust, J. M., Buncel, E., Lebel, O. & Nunzi, J.-M. Deceleration of Thermal Ring Closure in a Glass-Forming Mexylaminotriazine-Substituted Merocyanine (MC) Linked to Intramolecular Hydrogen Bonding. *New J. Chemistry* **41**, 940-947 (2017).

30 Seshadri, S. *et al.* Self-Regulating Photochemical Rayleigh-Bénard Convection Using a Highly-Absorbing Organic Photoswitch. *Nat. Commun.* **11**, 2599 (2020).

31 Brittain, W., Barrett, S., Mouer, L. & Sauceda, N. A Transient Phenolate Enables Relaxation of Z-Tetra-Ortho-Fluoroazobenzene to the Stable State in Seconds. *Angew. Chem. - Int. Ed.* **64**, e202513719 (2025).

Thirdly, selective and two-way thermal dynamic control

Water selectively interacts with specific QC isomers through structurally encoded hydrogen-bonding motifs, leading to a disproportionately large modulation of the thermal back-conversion barrier. Furthermore, we demonstrated reversible directional regulation of the back-conversion kinetics in Fig. 2c–f. These results show that hydrogen bonding and de-bonding can reversibly switch the kinetics between two distinct states in the **NBD6/QC6** system, strongly supporting that the observed dynamic behavior originates from the hydrogen-bonding interactions. Beyond the directional transport of backconversion dynamics, the demon can selectively control that some of QC cross the gate, while others cannot cross. We gave an example of QC6 and QC2. Upon the addition of water, only **QC6** can be slower $k^{II}_{6\text{-Slow}}$, while the $k^{II}_{2\text{-Fast}}$ kept the same values. Due to that $k^{II}_{6\text{-Slow}}$ is significantly slower than $k^{II}_{2\text{-Fast}}$, nearly all of **QC2** converted into **NBD2** in 1000 min, while the **QC6** remained unconverted.

Beyond the hydrogen-bonding interactions investigated here, other reversible and selective noncovalent interactions may also provide viable strategies for regulating the NBD/QC energy landscape. These may include coordination with small anions, supramolecular recognition motifs, or dynamic covalent interactions capable of selectively stabilizing the QC state. In addition, we now highlight the possibility of extending this strategy from solution systems to confined or solid-state environments, such as polymer matrices, supramolecular assemblies, or framework materials, where collective or matrix-assisted effects may further influence thermal stability and photochemical behavior. To strengthen the perspective of this work, the conclusion can highlight broader implications and alternative stabilization strategies for the NBD/QC system.

To clarify this, we have revised the conclusion as follows.

In Conclusion

Remarkably, **QC6**-water interactions prolong the thermal half-life by over 5000-fold compared to the anhydrous form, an effect attributed to multiple hydrogen bonds imposing steric and enthalpic constraints at the transition state. This strategy could be extended beyond intramolecular hydrogen bonding to other switchable interactions, such as small-anion coordination or reversible covalent motifs, offering new ways to tune the energy landscape. Complementary to such ground-state stabilization strategies, excited-state approaches, including triplet sensitization or vibronic-coupling pathways,[24] may provide orthogonal means to enhance photoisomerization efficiency and further optimize photoswitch performance. Beyond solution-phase systems, embedding such photoswitches in solid matrices or supramolecular frameworks could further enhance thermal stability and enable cooperative effects.

2. “The Maxwell’s demon analogy is misleading because water directly alters molecular enthalpy rather than reducing entropy without doing work.”

Response 2: We sincerely appreciate this important conceptual point and agree that the original Maxwell’s demon thought experiment should not be interpreted literally in the context of our molecular system. Our intention was not to claim that the present

system constitutes a rigorous thermodynamic realization of Maxwell's demon. Rather, we used the analogy in a conceptual sense to describe how environmental information (hydrogen bonding) can selectively discriminate between molecular states and thereby regulate kinetic behavior.

We agree with the reviewer that water directly interacts with the QC derivatives through hydrogen bonding and therefore contributes to enthalpic stabilization. We also agree that this differs fundamentally from the idealized Maxwell's demon framework in statistical mechanics, where entropy reduction is achieved without explicit energetic intervention.

Nevertheless, we believe that Maxwell's demon analogy remains conceptually valuable and scientifically instructive for the present work. First, Maxwell's demon thought experiment has long served as an important source of inspiration across chemistry, physics, and information science, not necessarily because experimental systems strictly reproduce the original entropy-defying scenario, but because the underlying concept of state-selective regulation provides a powerful framework for understanding how information, recognition, and environmental interactions can influence molecular behavior. In this sense, we use the analogy as a conceptual guide rather than as a literal thermodynamic equivalence.

More specifically, the central idea of Maxwell's demon, that a system can selectively distinguish between different states and thereby direct dynamic behavior, closely aligns with the mechanism observed in our study. In our system, the water selectively "recognizes" different QC isomeric states through hydrogen-bonding interactions. This selective recognition produces asymmetric modulation of the thermal back-conversion kinetics, where certain molecular states are preferentially stabilized relative to others. Importantly, this kinetic discrimination is not imposed through permanent covalent redesign or continuous external manipulation, but instead emerges adaptively from state-dependent interactions between the molecule and its environment. In this regard, Maxwell's demon analogy helps illustrate the concept of selective kinetic gating and environment-dependent state sorting that underlies our observations.

We also note that many modern studies (examples as below) invoking Maxwell's demon do not represent strict realizations of the original entropy-reduction thought experiment or testing the second law by attempting to reduce entropy, but rather adopt the concept in a broader phenomenological or information-guided context. For example, Maxwell's demon-inspired ideas have been widely used in molecular machines, Brownian ratchets, chemical transport, and chemically driven self-assembly systems, where selective transport or directional regulation arises from asymmetric interactions or information-like discrimination mechanisms, even though these systems still obey conventional thermodynamics and involve energetic inputs.

Example 1- Brownian ratchets

Brownian ratchets are connected to Maxwell's demon concept because both describe how random thermal motion can be directionally biased through selective gating or asymmetric regulation. In a Brownian ratchet system, stochastic molecular fluctuations are not eliminated; instead, the surrounding environment or structural asymmetry selectively rectifies these fluctuations into preferential motion or kinetic bias. Importantly, modern Brownian ratchet models are generally not interpreted as literal violations of the second law of thermodynamics, nor as strict realizations of entropy reduction without energetic input. Rather, they serve as conceptual frameworks for understanding how nonequilibrium interactions, kinetic asymmetry, and environment-

dependent selection can generate directional behavior in molecular systems. In this sense, the connection to Maxwell's demon is primarily phenomenological and conceptual, emphasizing selective kinetic discrimination rather than a rigorous thermodynamic equivalence.

Similarly, in our system, the hydrogen-bonding environment selectively stabilizes specific QC states and biases the thermal back-conversion pathway, making the "Maxwell's demon-inspired" analogy more appropriately understood in the context of kinetic rectification and state-selective regulation rather than a literal entropy-defying process.

Reference of brownian ratchets

1. Chatterjee, M. N., Kay, E. R. & Leigh, D. A. Beyond Switches: Ratcheting a Particle Energetically Uphill with a Compartmentalized Molecular Machine. *J. Am. Chem. Soc.* **128**, 4058-4073 (2006).
2. Serreli, V., Lee, C.-F., Kay, E. R. & Leigh, D. A. A Molecular Information Ratchet. *Nature* **445**, 523-527 (2007).
3. Kay, E. R., Leigh, D. A. & Zerbetto, F. Synthetic Molecular Motors and Mechanical Machines. *Angew. Chem. - Int. Ed.* **46**, 72-191 (2007).
4. Borsley, S., Leigh, D. A. & Roberts, B. M. W. Molecular Ratchets and Kinetic Asymmetry: Giving Chemistry Direction. *Angew. Chem. - Int. Ed.* **63**, e202400495 (2024).
5. Binks, L. *et al.* The Role of Kinetic Asymmetry and Power Strokes in an Information Ratchet. *Chem* **9**, 2902-2917 (2023).
6. Amano, S. *et al.* Insights from an Information Thermodynamics Analysis of a Synthetic Molecular Motor. *Nat. Chem.* **14**, 530-537 (2022).
7. Erbas-Cakmak, S., Leigh, D. A., McTernan, C. T. & Nussbaumer, A. L. Artificial Molecular Machines. *Chem. Rev.* **115**, 10081-10206 (2015).

Example 2- Chemical transport

The recent study (*Nat. Chem. 2024, 16, 1558-1564*) provides an important precedent for the modern use of Maxwell's demon concept in molecular systems. In that work, the authors employed selective host–guest recognition and light-driven isomerization to directionally transport molecules across compartments and establish concentration gradients far from equilibrium. Importantly, the system does not represent a literal realization of the original Maxwell's demon thought experiment in the strict thermodynamic sense of entropy reduction without energetic input. Instead, the process is explicitly driven by external light energy, selective molecular recognition, and asymmetric kinetic transport processes. As stated by the authors, the "demon" functions conceptually as a molecular gate that selectively discriminates between molecular states and biases stochastic transport pathways, while fully remaining consistent with the second law of thermodynamics. In this context, the term "Maxwell's demon" is used phenomenologically to describe state-selective kinetic regulation and directional bias rather than a rigorous entropy-defying mechanism.

Reference of chemical transport

1. Pruchyathamkorn, J. *et al.* Harnessing Maxwell's Demon to Establish a Macroscale Concentration Gradient. *Nat. Chem.* **16**, 1558-1564 (2024).

Example 3- Membrane transport

The recent review (*ChemSystemsChem 2024, 6, e202400004*) provides an important conceptual framework for understanding how Maxwell's demon principles are employed in modern molecular systems. In this work, the authors explicitly describe a wide range of molecular machines, membrane pumps, information ratchets, and transport systems using the language of Maxwell's demon, not as literal entropy-defying systems, but as kinetic and informational frameworks for understanding directional molecular processes. Importantly, the review emphasizes that these systems operate through kinetic asymmetry, state-dependent interactions, allosteric modulation, and selective gating mechanisms, often driven by external energy input such as light or chemical fuels. For example, ATP-driven transporters, rotaxane pumps, rotary motors, and photoresponsive membrane transport systems are all discussed as "Maxwell's demon-like" systems because they selectively discriminate molecular states and bias stochastic motion toward nonequilibrium behavior. However, these systems fully remain consistent with the second law of thermodynamics and are not intended as strict realizations of entropy reduction without energetic intervention. As discussed in the review, the "demon" terminology is therefore used primarily as a conceptual and phenomenological description of selective kinetic regulation and information-ratchet behavior.

Reference of membrane transport

1. Borsley, S. Membrane Transport, Molecular Machines, and Maxwell's Demon. *ChemSystemsChem* **6**, e202400004 (2024).

We appreciate the reviewer's concern that the original wording may overstate the connection to Maxwell's demon concept. To avoid possible misunderstanding, we have revised the manuscript and supporting information to substantially soften the language and now describe the phenomenon as follows:

Title:

Orchestrating Photoswitch Thermal Backconversion: Hydrogen Bonds Reshape Energy Landscapes

Abstract:

Thermodynamic control of molecular photoswitches remains a fundamental challenge, centered on the stabilization of high-energy metastable states. Inspired by concepts of selective kinetic regulation found in Maxwell's demon-like systems, we explore hydrogen bonding as a molecular-level selector for modulating the thermal back-conversion of quadricyclane (QC) to norbornadiene (NBD). By strategically incorporating hydrogen-bond donors and acceptors, we show that these interactions build a water "bridge" that restricts rotational degrees of freedom. This bridge significantly elevates the kinetic barrier and redirects the isomerization along an alternative reaction coordinate. In this manner, the hydrogen-bonding system selectively regulates the thermal back-conversion dynamics and enables directional control over the isomerization kinetics, achieving up to a 5000-fold extension of the thermal half-life. This work illustrates how hydrogen bonds can facilitate precise control over back-conversion dynamics and molecular thermal stability in molecular photoswitches.

Introduction:

The spontaneous decay of high-energy states toward thermodynamic equilibrium is a fundamental law of nature. Yet, the pursuit of strategies to sustain non-equilibrium states has remained a cross-disciplinary challenge for over a century. This inquiry traces its intellectual lineage to the seminal thought experiments of Maxwell's paradox and Landauer's principle (**Figs. 1**a, b),[1-2] which conceptualize a "demon" capable of selectively partitioning particles to reduce a system's entropy.[3-5] In current molecular science, Maxwell's demon-inspired systems are generally not viewed as strict realizations of the original entropy-reduction thought experiment or as attempts to violate the second law of thermodynamics. Rather, the concept has evolved into a broader phenomenological and information-guided framework for describing selective kinetic regulation, directional transport, and nonequilibrium molecular processes, including molecular machines and membrane transport systems (**Fig. 1**c).[6-11] However, while significant progress has been made in driving molecular systems away from equilibrium, the challenge of stabilizing these transient states through autonomous, selective regulation remains virtually unexplored.

Results and Discussion

Page 3:

Molecular design for a Maxwell's demon-inspired system

A Maxwell's demon-inspired framework is used to describe a selective molecular gating behavior that biases transport directionality within a multi-box system (**Fig. 1**d). Our system comprises two compartments (the Slow Box and Fast Box), representing distinct thermal back-conversion rates from QC to NBD, separated by dynamic hydrogen-bonding networks. These hydrogen-bonding interactions stabilize the designed QC derivatives and increase the thermal back-conversion barrier. Importantly, water molecules can modulate these interactions by selectively interacting with the QC derivatives, thereby acting as a dynamic regulatory mediator of the local hydrogen-bonding environment. Initially, QCs distribute between the Slow and Fast Boxes depending on the presence of hydrogen bonds. The gating direction is tunable: introducing water promotes hydrogen bonding, shifting QCs from the Fast Box to the Slow Box, whereas removing water reverses this flow (detailed see in supplement **Section 2.5**, **Figs. S1**, and **2**).

Page 6

Thermal backconversion dynamics control

To further verify the direct control of backconversion dynamics in this selective gating system, two-way experiments of **QC6** between the Fast Box and Slow Box were performed. In the initial state, when the **QC6** solution contained no water, approximately 30% of **QC6** converted into **NBD6** within 250 minutes, with a $k^{I}_{6\text{-Fast}}$ value of 1.1 ×10^{-3} min^{-1} (**Figs. 2**d and h). Upon the addition of water, the backconversion rate of **QC6** decreased dramatically, with the $k^{II}_{6\text{-Slow}}$ value reducing to 9.8 ×10^{-5}. In this scenario, the system enables a preferential redistribution of species from the Fast Box to the Slow Box. Conversely, when the **QC6** solution initially contained water, the backconversion rate accelerated again after the water was removed, corresponding to a transition from the Slow Box back to the Fast Box (**Figs. 2**e and f). To confirm the selectivity of this gating behavior, a control experiment was performed using **QC2** (**Fig. S64**).

Page 9

Compared to traditional Maxwell's demon-inspired systems where molecular transport is typically considered between two compartments, here we demonstrate a multi-state system regulated through hydrogen-bonding interactions. Changes in the NBD-to-water molar ratio provide a direct way to control the backconversion dynamics, which can be regarded as analogous to infinitely splitting the boxes (**Fig. 3**a).

Page 14

These findings provide insight into the mechanism underlying the selective regulation of thermal backconversion. Water channels the solute into preferred conformations, reducing rotational entropy in exchange for enthalpic stabilization through hydrogen bonding.

Methods:

Page 17

In order to demonstrate selective transport behavior, we mixed two types of NBD/QC pairs in one solution. Solutions of **NBD6**/**QC6** and NBD2/QC2 pairs were prepared in anhydrous MeCN (0.1 mM) and heated to 60 °C.

Compared to traditional selective transport systems involving two compartments, we demonstrate a multi-compartment system regulated by hydrogen-bonding interactions. The NBD-to-water molar ratio change is a direct way to control the backconversion dynamics, which can be seen as like an infinitely box splitting. To illustrate this, we designed an experiment with various NBD-to-water molar ratios as described below.

Supporting information

Table of Contents

1. Maxwell's demon-inspired system .... **Error! Bookmark not defined.**
1.1 What is Maxwell's Demon .... **Error! Bookmark not defined.**
1.2 How does it inspire in this study .... **Error! Bookmark not defined.**
2. Experimental Procedures .... **Error! Bookmark not defined.**
2.1 Materials .... **Error! Bookmark not defined.**
2.2 Molecular synthesis .... **Error! Bookmark not defined.**
2.3 Computational Modelling Approach .... **Error! Bookmark not defined.**
2.4 Characterization and Methods .... **Error! Bookmark not defined.**
2.4.1 Structural characterization. .... **Error! Bookmark not defined.**
2.4.2 Photostationary state measurement. .... **Error! Bookmark not defined.**
2.4.3 Hydrogen bonds characterization. .... **Error! Bookmark not defined.**
2.4.4 Thermal back-conversion kinetics .... **Error! Bookmark not defined.**
2.5 Maxwell's demon experiments .... **Error! Bookmark not defined.**
2.6 NMR spectra .... **Error! Bookmark not defined.**
2.6.1 NMR of NBD-CN,Br .... **Error! Bookmark not defined.**
2.6.2 NMR of NBD1 .... **Error! Bookmark not defined.**
2.6.3 NMR of NBD2 .... **Error! Bookmark not defined.**
2.6.4 NMR of NBD3 .... **Error! Bookmark not defined.**
2.6.5 NMR of ethyl 3-bromopropiolate .... **Error! Bookmark not defined.**
2.6.6 NMR of NBD-Ester,Br .... **Error! Bookmark not defined.**
2.6.7 NMR of NBD4 .... **Error! Bookmark not defined.**

2.6.8 NMR of NBD5 ....Error! Bookmark not defined.
2.6.9 NMR of NBD6 ....Error! Bookmark not defined.
3. Results and Discussion ....Error! Bookmark not defined.
3.1 Molecular design ....Error! Bookmark not defined.
3.2 Thermal backconversion dynamics........................Error! Bookmark not defined.
3.2.1 Thermal back conversion of NBD/QC pairs in MeCN Error! Bookmark not defined.
3.2.2 Selectively control thermal backconversion .Error! Bookmark not defined.
3.2.4 Thermal back conversion of NBD6/QC6 pair in various solvents ....Error! Bookmark not defined.
3.3 The reversible hydrogen bonding systems ....Error! Bookmark not defined.
4. Theoretical calculation...Error! Bookmark not defined.
4.1 Global conformer searches ....Error! Bookmark not defined.
4.2 Dihedral angle scan and energy landscape...........Error! Bookmark not defined.
4.3 Molecular dynamics simulations ....Error! Bookmark not defined.
4.4 Cartesian Coordinates..Error! Bookmark not defined.
Captions for Movies...Error! Bookmark not defined.
References..Error! Bookmark not defined.

Page 2

While NBD/QC chemistry has been central to molecular solar energy research, the same design principles, including the strategic encoding of information through hydrogen bonds, and substituent effects, extend naturally to other classes of photoswitches. This perspective highlights how hydrogen-bonding-mediated regulation can be integrated with modern supramolecular chemistry, opening pathways toward adaptive and programmable photoresponsive materials that combine energy storage, actuation, and sensing within a single molecular platform.

Page 48

To further verify the selective regulation of backconversion dynamics in this system, two-way experiments of **QC2** between the Fast Box and Slow Box were performed. In the initial state, when the **QC2** solution contained no water, approximately 60% of **QC2** converted into **NBD6** within 1550 min, with a $k_{2\text{-Fast}}$ value of 5.8 ×10^{-4} min^{-1}.

Description of Additional Supplementary Files

Movie S1. Backconversion process from **QC6** to **NBD6**.

Movie S2. Dihedral angles and energy landscapes in **QC1**.

Movie S3. Dihedral angles and energy landscapes in **QC6**.
